# Supplementary material for: A cysteine selenosulfide redox switch for protein chemical synthesis
Source: Nat Commun. 2020 May 22;11:2558. doi: 10.1038/s41467-020-16359-6 (PMC7244499; doi:10.1038/s41467-020-16359-6)
Supplement: Supplementary file 1 — Supplementary Information [file 41467_2020_16359_MOESM1_ESM.pdf]

Supplementary Information for

A cysteine selenosulfide redox switch for protein chemical synthesis

Vincent Diemer, Nathalie Ollivier, Bérénice Leclercq, Hervé Drobecq, Jérôme Vicogne, Vangelis Agouridas*, Oleg Melnyk*

Univ. Lille, CNRS, Inserm, CHU Lille, Institut Pasteur de Lille, U1019 - UMR 9017 - CIIL - Center for Infection and Immunity of Lille, F-59000 Lille, France

Corresponding authors:

Dr Oleg Melnyk, E-mail : oleg.melnyk@ibl.cnrs.fr

Dr Vangelis Agouridas, E-mail: vangelis.agouridas@ibl.cnrs.fr

Website: http://olegmelnyk.cnrs.fr

Table of content

Supplementary Methods 2

Supplementary Figures 30

Supplementary Tables 130

Supplementary References 132

Supplementary Methods

Synthesis of the Fmoc-protected SetCys amino acid SI8 (see Supplementary Figure 1)

*Synthesis of methyl S-trityl-L-cysteinate **SI2**.*

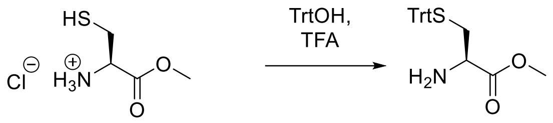

SI1 **SI2**

To a solution of cysteine methyl ester hydrochloride **SI1** (856 mg, 5.00 mmol, 1.0 equiv) in TFA (5 mL) was added triphenylmethanol (1.43 g, 5.49 mmol, 1.1 equiv) and the mixture was stirred at rt for 5 h. After evaporation of the TFA, the residue was dissolved in methanol and the mixture was stirred at rt until the yellow color disappeared. The methanol was then evaporated under reduced pressure and the residue was suspended in 0.25 M K_2CO_3 (30 mL). The obtained aqueous layer was extracted with Et_2O (2 × 20 mL) and the combined organic layers were dried over $MgSO_4$. After evaporation of the solvent, purification of the crude by column chromatography (CH_2Cl_2/MeOH 98:2) provided the *S*-trityl protected amino acid **SI2** (1.73 g) as a viscous oil with 91% yield.

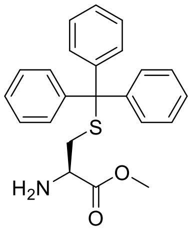

SI2

Chemical Formula: $C_{23}H_{23}NO_2S$
Molecular Weight: 377,5020

NMR data for compound **SI2** are in agreement with the literature.[1]

^{1}H NMR (300 MHz, $CDCl_3$, Supplementary Figure 10) δ 7.41-7.44 (m, 6H), 7.18-7.31 (m, 9H), 3.65 (s, 3H), 3.20 (dd, J = 4.8 and 7.8 Hz, 1H), 2.59 (dd, J = 4.8 and 12.4 Hz, 1H), 2.46 (d, J = 7.8 and 12.4 Hz, 1H) ppm.

^{13}C NMR (75 MHz, $CDCl_3$, Supplementary Figure 11) δ 174.3 (C), 144.6 (3 × CH), 129.7 (6 × CH), 128.1 (6 × CH), 126.9 (3 × CH), 66.9 (C), 53.9 (CH), 52.3 (CH_3), 37.2 (CH_2) ppm.

Synthesis of methyl N-(2-((4-methoxybenzyl)selanyl)ethyl)-S-trityl-L-cysteinate ***SI5****.*

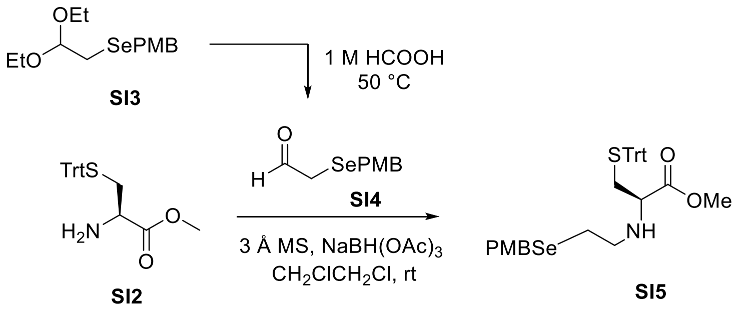

(2,2-Diethoxyethyl)(4-methoxybenzyl)selenide **SI3** was prepared according to Cargoët *et al.*[2]

(2,2-Diethoxyethyl)(4-methoxybenzyl)selenide **SI3** (1.18 g, 3.72 mmol, 1.0 equiv) was suspended in 1 M formic acid (15 mL) and the mixture was heated overnight at 50 °C. Water (50 mL) was added and the aqueous layer was extracted with Et_2O (2 × 50 mL). The combined organic extracts were washed with water (50 mL) and dried over $MgSO_4$. After evaporation of the solvent under reduced pressure, crude aldehyde **SI4** was dissolved in anhydrous 1,2-dichloroethane (50 mL) under argon. Activated powdered 3 Å molecular sieves (4 g), sodium triacetoxyborohydride (1.10 g, 5.19 mmol, 1.4 equiv) and a solution of compound **SI2** (1.41 g, 3.73 mmol, 1.0 equiv) in DCE (35 mL) were successively added to the solution of aldehyde. After 18 h stirring at rt, the reaction mixture was filtered on a Büchner funnel and the solid was washed with additional portions of CH_2Cl_2. The filtrate was evaporated under reduced pressure. Then 0.5 M K_2CO_3 (50 mL) was added to the residue and the aqueous layer was extracted with CH_2Cl_2 (3 × 50 mL). The combined organic layers were dried over $MgSO_4$ and the solvent was evaporated under reduced pressure. Purification of the crude mixture by column chromatography (cyclohexane/ethyl acetate 80:20) afforded the *N*-alkylated amino acid **SI5** as a clear yellow oil (1.94 g, 86%).

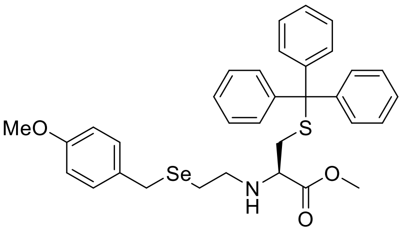

SI5

Chemical Formula: $C_{33}H_{35}NO_3SSe$
Molecular Weight: 604,6670

^{1}H NMR (300 MHz, $CDCl_3$, Supplementary Figure 12) δ 7.41 (d, *J* = 7.4 Hz, 6H), 7.19-7.32 (m, 9H), 7.17 (d, *J* = 8.6 Hz, 2H), 6.79 (d, *J* = 8.6 Hz, 2H), 3.77 (s, 3H), 3.71 (s, 2H), 3.66 (s, 3H), 2.99 (t, *J* = 6.5 Hz, 1H), 2.37-2.74 (m, 6H), 1.44-1.91 (m, 1H) ppm.

^{13}C NMR (75 MHz, $CDCl_3$, Supplementary Figure 13) δ 173.5 (C), 158.4 (C), 144.6 (3 × C), 131.1 (C), 129.9 (2 × CH), 129.6 (6 × CH), 127.9 (6 × CH), 126.7 (3 × CH), 113.9 (2 × CH), 66.8 (C), 60.2 (CH), 55.2 (CH_3), 51.9 (CH_3), 47.3 (CH_2), 34.6 (CH_2), 26.3 (CH_2), 23.9 (CH_2) ppm.

IR (ATR, cm^{-1}) 3029, 2949, 1736, 1698, 1243, 1172, 738.

MALDI-TOF (matrix 2,5-dihydroxybenzoic acid, positive detection mode, Supplementary Figure 14) Calcd. for $[M+Na]^+$ (monoisotopic) 628.14, found 628.06.

HRMS (ES^+): Calcd. for $C_{33}H_{35}NO_3SNaSe$: 628.1401, found: 628.1378.

$[\alpha]_D^{20}$ -19 ° (*c* 1.0, chloroform).

Synthesis of methyl N-(((9H-fluoren-9-yl)methoxy)carbonyl)-N-(2-((4-methoxybenzyl)selanyl)ethyl)-S-trityl-L-cysteinate ***SI6****.*

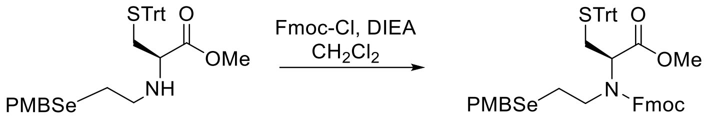

SI5 **SI6**

To a solution of compound **SI5** (2.12 g, 3.51 mmol, 1.00 equiv) in CH_2Cl_2 (20 mL) were successively added DIEA (0.638 mL, 3.67 mmol, 1.05 equiv) and Fmoc-Cl (950 mg, 3.67 mmol, 1.05 equiv). The reaction mixture was stirred at rt for 24 h and was then diluted with CH_2Cl_2. The obtained organic layer was washed with water and was dried over $MgSO_4$. After evaporation of the solvent under reduced pressure, purification of the crude by column chromatography (cyclohexane/EtOAc 7:3) provided the expected compound **SI6** as a white solid (2.87 g, 99%).

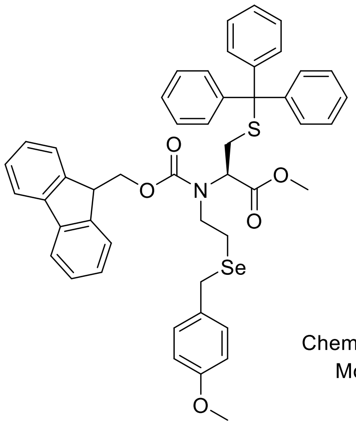

SI6

Chemical Formula: $C_{48}H_{45}NO_5SSe$
Molecular Weight: 826,9100

Compound **SI6** was characterized by ^{1}H and ^{13}C NMR (Supplementary Figure 15, Supplementary Figure 16, Supplementary Figure 17) as a mixture of two conformers A and B (A/B = 37/63, as determined from integration of the CH signals of the Fmoc group by ^{1}H NMR).

^{1}H NMR (300 MHz, $CDCl_3$, Supplementary Figure 15) δ 7.80 (d, J = 9.0 Hz, 0.64H, $2H_A$), 7.74 (d, J = 7.6 Hz, $2H_B$), 7.49-7.59 (m, $2H_A$ + $2H_B$), 7.12-7.49 (m, $21H_A$ + $19H_B$ + residual $CDCl_3$), 7.08 (d, J = 8.3 Hz, $2H_B$), 6.89 (d, J = 8.3 Hz, $2H_A$), 6.71 (d, J = 8.4 Hz, $2H_B$), 4.34-4.60 (m, $2H_A$ + $2H_B$), 4.20 (t, J = 5.9 Hz, H_B), 4.13 (t, J = 5.2 Hz, H_A), 3.75 (s, $3H_A$), 3.73 (s, $2H_A$), 3.68 (s, $3H_B$) 3.40-3.63 (m, H_A + $5H_B$), 3.02-3.40 (m, $4H_A$ + $2H_B$), 2.45-3.02 (m, $3H_A$ + $3H_B$), 2.17-2.45 (m, $2H_A$ + $2H_B$) ppm.

^{13}C NMR (75 MHz, $CDCl_3$, Supplementary Figure 16) δ 170.0 (C, B), 169.7 (C, A), 158.4 (C, A +B), 155.2 (C, A + B), 144.5 (C, A + B), 143.7 (C, A + B), 141.4 (C, A), 141.3 (C, B), 131.3

(C, A), 130.9 (C, B), 129.8 (CH, A + B), 129.6 (CH, A + B), 128.0 (CH, A + B), 127.7 (CH, A + B), 127.1 (CH, A + B), 126.8 (CH, A + B), 124.3-125.3 (m, CH, A + B), 120.0 (CH, A + B), 113.9 (A + B), 67.2 (CH_2, A), 67.0 (CH_2, B), 60.6 (CH, B), 59.8 (CH, A), 55.1 (CH_3, A+ B), 52.3 (CH_3, B), 52.2 (CH_3, A), 49.6 (CH_2, A), 49.2 (CH_2, B), 47.2 (CH, B), 47.1 (CH, A), 31.5 (CH_2, A), 31.2 (CH_2, B), 26.5 (CH_2, A + B), 21.3 (CH_2, B), 20.8 (CH_2, A) ppm.

IR (ATR, cm^{-1}) 3054, 2949, 1742, 1699, 1509, 1445, 1283, 1245, 741, 701.

HRMS (ES^+): Calcd. for $C_{48}H_{45}NO_5SNaSe$: 850.2081, found: 850.2089.

MALDI-TOF (matrix 2,5-dihydroxybenzoic acid, positive detection mode, Supplementary Figure 18) Calcd. for $[M+Na]^+$ (monoisotopic) 850.2, found 849.9.

$[\alpha]_D^{20}$ (c 1.0, $CHCl_3$): -38 °.

*Synthesis of 5-((9H-fluoren-9-yl)methyl) 6-methyl (R)-1,2,5-thiaselenazepane-5,6-dicarboxylate **SI7***

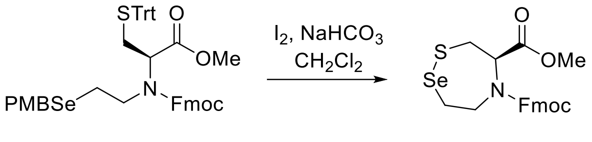

SI6 **SI7**

To a solution of compound **SI6** (1.67 g, 2.02 mmol, 1 equiv) in CH_2Cl_2 (50 mL) was added $NaHCO_3$ (508 mg, 6.05 mmol, 3. equiv). The mixture was cooled to 0 °C and I_2 (1.54 g, 6.06 mmol, 3 equiv) dissolved in CH_2Cl_2 (50 mL) was added dropwise. The reaction mixture was stirred at rt for 30 min and was washed 1 M $Na_2S_2O_3$ (50 mL) to remove the excess of I_2. The organic layer was separated and the aqueous layer was extracted with CH_2Cl_2 (2 × 50 mL). Note that the separation between the aqueous and organic layers was improved by addition of brine (50 mL). All the organic layers were then combined, washed with brine (50 mL) and dried over $MgSO_4$. After evaporation of the solvent, purification of the crude by column chromatography (cyclohexane/EtOAc 8:2) provided the expected compound **SI7** as a glass (780 mg, 83%).

LC-MS analysis of compound **SI7** can be found in Supplementary Figure 19.

Compound **SI7** was characterized by ^1H and ^{13}C NMR (Supplementary Figure 20, Supplementary Figure 21, Supplementary Figure 22) as a mixture of two main conformers A and B (A/B = 45:55, as determined from the integration of aromatic CH signals of the Fmoc group). Due to overlapping signals, no clear description of the ^1H NMR spectrum can be provided.

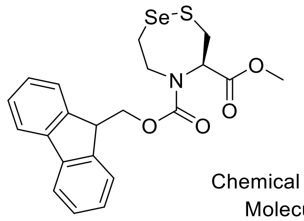

SI7

Chemical Formula: $C_{21}H_{21}NO_4SSe$

Molecular Weight: 462,4220

^{13}C NMR (75 MHz, $CDCl_3$, Supplementary Figure 21) δ 170.0 (C), 169.7 (C), 155.6 (C), 155.2 (C), 143.90 (C), 143.88 (C), 143.84 (C), 143.6 (C), 141.6 (C), 141.53 (C), 141.51 (C), 127.91 (CH), 127.88 (CH), 127.86 (CH), 127.7 (CH), 127.34 (CH), 127.30 (CH), 127.25 (CH), 127.1 (CH), 124.8 (CH), 124.4 (CH), 124.3 (CH), 67.1 (CH_2), 66.8 (CH_2), 62.7 (CH), 52.7 (CH_3), 52.4 (CH_3), 49.8 (CH_2), 47.4 (CH), 47.2 (CH), 38.6 (CH_2), 29.3 (CH_2), 29.1 (CH_2) ppm.

IR (ATR, cm^{-1}) 2948, 1740, 1697, 1474, 1449, 1417, 1278, 738.

MALDI-TOF (matrix 2,5-dihydroxybenzoic acid, positive detection mode, Supplementary Figure 23) Calcd. for $[M+Na]^+$ (monoisotopic) 486.0, found 486.2.

HRMS (ES^+) Calcd. for $C_{21}H_{21}NO_4NaSSe$: 486.0254, found: 486.0246.

$[\alpha]_D^{20}$ (c 1.0, $CHCl_3$): -29 °.

*Synthesis of (R)-5-(((9H-fluoren-9-yl)methoxy)carbonyl)-1,2,5-thiaselenazepane-6-carboxylic acid **SI8***

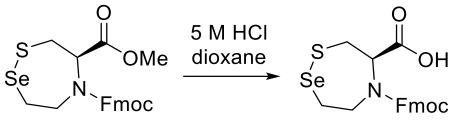

SI7 **SI8**

To a solution of compound **SI7** (411 mg, 0.889 mmol) in dioxane (19 mL) was added 5 M HCl (9 mL) and the mixture was refluxed for 6 h. The reaction mixture was cooled to rt and 5% K_2CO_3 (200 mL) was added to raise the pH above 9. The aqueous layer was then washed with E_2O (2 × 100 mL), acidified with concentrated HCl until pH ≈ 2-3 and extracted with CH_2Cl_2 (3 × 100 mL). The organic layers (CH_2Cl_2) were combined and dried over $MgSO_4$. Evaporation of the solvent under reduced pressure provided the expected amino acid **SI8** (345 mg, 86%) as a white solid which was used in the next step without further purification.

LC-MS analysis of compound **SI8** can be found in Supplementary Figure 24.

Compound **SI8** was characterized by ^{1}H and ^{13}C NMR as a mixture of conformers (Supplementary Figure 25, Supplementary Figure 26). Due to overlapping signals, no clear description of the ^{1}H NMR spectrum can be provided.

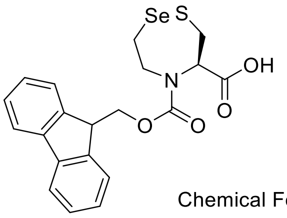

SI8

Chemical Formula: $C_{20}H_{19}NO_4SSe$
Molecular Weight: 448,3950

IR (ATR, cm^{-1}) 3000 (broad signal), 1699 (broad), 1476, 1450, 1419, 1286, 1190, 740.

HRMS (ES^+): Calcd. for $C_{20}H_{19}NO_4NaSSe$: 472.0098, found: 472.0116.

MALDI-TOF (matrix 2,5-dihydroxybenzoic acid, positive detection mode, Supplementary Figure 27) $[M+Na]^+$ calcd. (monoisotopic) 472.01, found 471.95.

$[\alpha]_D^{20}$ (c 1.0, $CHCl_3$): -32 °.

Synthesis of the PMB-protected Fmoc-SetAla-OH amino acid SI2 (see Supplementary Figure 2)

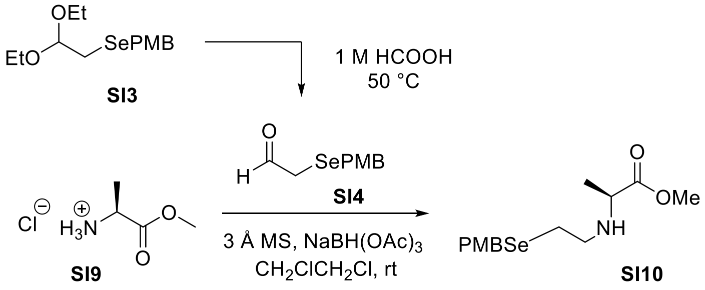

(2,2-Diethoxyethyl)(4-methoxybenzyl)selenide **SI3** was prepared according to Cargoët et al.[2]

(2,2-Diethoxyethyl)(4-methoxybenzyl)selenide **SI3** (230 mg) was suspended in 1 M formic acid (6 mL) and the mixture was heated overnight at 50 °C. Water (50 mL) was added and the aqueous layer was extracted with Et_2O (2 × 50 mL). The combined organic extracts were washed with water (50 mL) and dried over $MgSO_4$. After evaporation of the solvent under reduced pressure, aldehyde **SI4** (184 mg, 0.757 mmol, 1 equiv) was dissolved in anhydrous 1,2-dichloroethane (15 mL) under argon. Alanine methyl ester hydrochloride **SI9** (106 mg, 0.759 mmol, 1 equiv), DIEA (146 µL, 0.840 mmol, 1.1 equiv), activated powdered 3 Å molecular sieves (760 mg) and sodium triacetoxyborohydride (208 mg, 0.981 mmol, 1.3 equiv) were successively added to the solution of aldehyde. After 20 h stirring at rt, the reaction mixture was filtered on a Büchner funnel and the solid was washed with additional portions of CH_2Cl_2. The filtrate was evaporated under reduced pressure. Then 0.5 M K_2CO_3 (30 mL) and saturated NaCl (30 mL) were added to the residue and the aqueous layer was extracted with CH_2Cl_2 (3 × 50 mL). The combined organic layers were dried over $MgSO_4$ and the solvent was evaporated under reduced pressure to produce the crude *N*-alkylated derivative **SI10**.

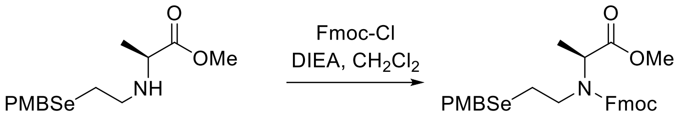

SI10 **SI11**

The crude *N*-alkylated derivative **SI10** (272 mg, 0.823 mmol, 1 equiv) was dissolved in CH_2Cl_2 (4 mL). DIEA (142 µL, 0.820 mmol, 1 equiv) and Fmoc-Cl (212 mg, 0.819 mmol, 1 equiv) were added to the solution of **SI10**. After 24 h stirring at rt, the reaction mixture was diluted with an additional portion of CH_2Cl_2. The organic layer was washed with water and was dried over $MgSO_4$. After evaporation of the solvent under reduced pressure, purification of the crude by column chromatography (cyclohexane/EtOAc 7:3) provided the Fmoc-protected derivative **SI11**, in mixture with an impurity that resulted from Fmoc-Cl decomposition (310 mg).

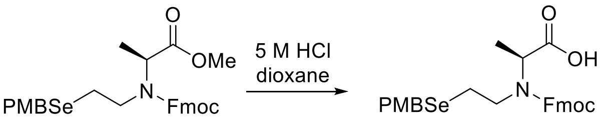

SI11 **SI12**

To the a portion (140 mg) of impure compound **SI11** isolated by column chromatography were added dioxane (6 mL) and 5 M HCl (3 mL) and the mixture was refluxed for 6 h. The reaction mixture was then diluted with 1 M HCl (40 mL) and the aqueous layer was extracted with CH_2Cl_2 (3 × 40 mL). The combined organic layers were dried over $MgSO_4$ and the solvent was evaporated under reduced pressure. Purification of the crude by column chromatography (CH_2Cl_2/MeOH 99:1 → 95:5) provided the expected amino acid **SI12** as an amorphous solid (55 mg, 30% from **SI9**).

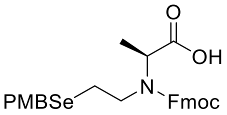

Chemical Formula: $C_{28}H_{29}NO_5Se$
Molecular Weight: 538.50
SI12

Compound **SI12** was analyzed by LC-MS (Supplementary Figure 28).

Compound **SI12** was characterized by ^{1}H (Supplementary Figure 29) and ^{13}C NMR (Supplementary Figure 30) as a mixture of conformers.

^{1}H NMR (300 MHz, $CDCl_3$, Supplementary Figure 29) δ 7.75 (d, *J* = 6.1 Hz, 2H), 7.55 (d, *J* = 7.1 Hz, 2H), 7.07-7.45 (m, 6H), 6.70-6.86 (m, 2H), 4.55 (br s, 2H), 3.97-4.39 (m, 2H), 3.59-3.84 (m, 5H), 2.88-3.59 (m, 2H), 2.18-2.83 (m, 2H), 1.19-1.43 (m, 3H) ppm.

^{13}C NMR (75 MHz, $CDCl_3$, Supplementary Figure 30) δ 176.58 (C), 158.47 (C), 155.95 (C), 143.71 (C), 141.27 (C), 130.82 (C), 129.91 (CH), 127.71 (CH), 127.10 (CH), 124.71 (CH),120.01 (CH), 113.93 (CH), 67.42 (CH_2), 67.16 (CH_2), 55.62 (CH), 55.20 (CH_3), 54.99 (CH), 47.71 (CH_2), 47.35 (CH_2), 47.27 (CH), 26.59 (CH_2), 21.83 (CH_2), 15.74 (CH_3), 15.16 (CH_3) ppm.

ESI (positive detection mode, Supplementary Figure 28): $[M+Na]^+$ m/z calcd. (monoisotopic mass) 562.11, found 562.33.

IR (ATR, cm^{-1}) 3065, 2995, 2932, 1739, 1696, 1509, 1284, 1244, 1172, 1101, 726.

$[\alpha]_D^{20}$ (c 1.0, $CHCl_3$): -9 °.

Peptide synthesis

Peptide amides (see Supplementary Figure 3)

Model cysteinyl peptide CILKEPVHGV-NH_2 has already been synthesized and characterized elsewhere.[3]

The biotinylated cysteinyl peptide segment **14** (K1[177-209]-K(Biot)-NH_2) was synthesized as described elsewhere.[4]

*Synthesis of SetCys-ALKEPVHGV-NH_2 peptide **1***

SetCys-ALKEPVHGV-NH_2 peptide **1** was synthesized on 0.1 mmol scale as described in the general procedure presented in the Methods section. TFA/TIS/H_2O/EDT 92.5/2.5/2.5/2.5 v/v/v/v (12 mL) was used as cleavage cocktail. Peptide **1** which was recovered by precipitation from Et_2O/heptane was used in the next step without further purification (48 mg, 32%).

Peptide **1** was analyzed by LC-MS (Supplementary Figure 31) and MALDI-TOF MS (Supplementary Figure 32).

ESI (positive detection mode, Supplementary Figure 31) m/z calcd. for $[M+H]^+$ (monoisotopic mass): 1157.5, found: 1157.6.

MALDI-TOF (matrix α-cyano-4-hydroxycinnamic acid, positive detection mode, Supplementary Figure 32) calcd. for $[M+H]^+$ (monoisotopic): 1157.5, found: 1157.8.

Synthesis of the SetAla peptide precursor

The diselenide (SetAla-ALKEPVHGV-NH_2)$_2$ used as SetAla peptide precursor was synthesized from the PMB-protected SetAla peptide PMB-SeCH_2CH_2-Ala-ALKEPVHGV-NH_2.

PMB-SeCH_2CH_2-Ala-ALKEPVHGV-NH_2 peptide was synthesized on 0.05 mmol scale. The peptide was elongated according to general procedure presented in the Methods section. The PMB-protected Fmoc-SetAla-OH amino acid was coupled as described for Fmoc-SetCys-OH and TFA/H_2O/TIS 95/2.5/2.5 v/v/v (5 mL) was used as cleavage cocktail. PMB-SeCH_2CH_2-Ala-ALKEPVHGV-NH_2 peptide was recovered by precipitation from Et_2O/heptane, solubilized in water and lyophilized (43.0 mg).

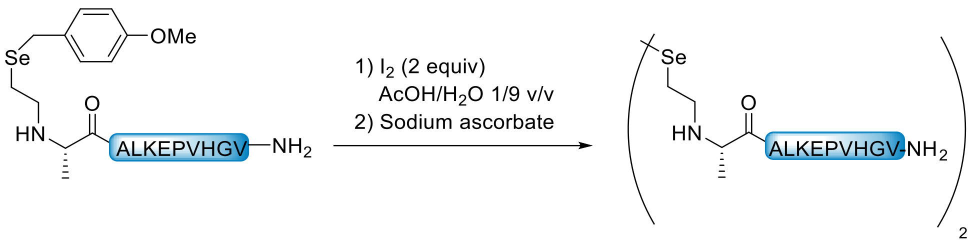

Crude PMB-SeCH$_2$CH$_2$-Ala-ALKEPVHGV-NH$_2$ peptide was converted into the final diselenide by oxidation with iodine using the following procedure. The crude PMB-SeCH$_2$CH$_2$-Ala-ALKEPVHGV-NH$_2$ peptide (21.0 mg, 1 equiv) was solubilized in AcOH/water 1/4 v/v (13.2 mL) and a solution of I_2 in DMSO (109 µL, 61.4 mg mL^{-1}, 2 equiv) was added dropwise. After 30 min of stirring at rt, a solution of sodium ascorbate in water (9.9 mg mL^{-1}) was added dropwise until the complete disappearance of I_2 yellow color. Purification of the crude by preparative HPLC (eluent A 0.1% TFA in water, eluent B 0.1% TFA in CH$_3$CN, 20 mL min^{-1}, 0-10% eluent B in 4 min then 10-30% eluent B in 29 min, C18 XBridge 5 µm (19 × 150 mm) column, detection at 215 nm, rt) provided the expected diselenide as a white solid after lyophilisation (8.7 mg, 24% overall starting from solid support).

The diselenide (SetAla-ALKEPVHGV-NH$_2$)$_2$ was analyzed by LC-MS (Supplementary Figure 33) and MALDI-TOF MS (Supplementary Figure 34).

ESI (positive detection mode, Supplementary Figure 33): m/z = 1126.6 ([M+2H]$^{2+}$), 750.9 ([M+3H]$^{3+}$), 563.6 ([M+4H]$^{4+}$); calcd. for M (average): 2250.4, found 2250.4.

MALDI-TOF (matrix α-cyano-4-hydroxycinnamic acid, positive detection mode, Supplementary Figure 34) Calcd. for [M+H]$^+$ (selenol): 1127.55, found: 1127.53; calcd for [M+H]$^+$ (diselenide): 2252.07, found: 2252.05.

Synthesis of CHOCVCKNTC-NH$_2$

CHOCVCKNTC-NH$_2$ peptide was synthesized on 0.1 mmol scale as described in the general procedure and was cleaved from the solid support using TFA/TIS/H$_2$O/thioanisol/thiophenol 92.5/5/2.5/2.5/2.5 v/v/v/v/v (10 mL). The solid that was recovered by precipitation from Et$_2$O/heptane was solubilized in water and lyophilized. Purification of the crude by HPLC (eluent A 0.1% TFA in water, eluent B 0.1% TFA in CH$_3$CN, 20 mL min^{-1}, 0-5% eluent B in 10 min then 5-25% in 25 min, C18 column, detection at 215 nm, rt) provided the desired peptide as a white powder after lyophilization (61.3 mg, 42%).

Peptide CHOCVCKNTC-NH$_2$ was characterized by LC-MS (Supplementary Figure 35).

ESI (positive detection mode, Supplementary Figure 35): Calcd. for [M+H]$^+$ (monoisotopic): 1122.42, found: 1122.42.

SEA peptides (see Supplementary Figure 4)

AcA-K1[128-148]-SEA^{off} and K1[149-176]-SEA^{off} peptide segments needed for the assembly of peptide **9b** have already been synthesized and characterized elsewhere.[5]

The SEA peptide segment **12** (K1[125-148]-SEA^{off}) needed for the assembly of linear K1 polypeptide **15** was synthesized as described elsewhere.[6]

*Synthesis of peptide **9a***

Peptide **9a** was synthesized on a 0.2 mmol scale as described in the general procedure presented in the Methods section. TFA/H_2O/TIS/thiophenol/thioanisole 87.5/2.5/5/2.5/2.5 v/v/v/v/v (20 mL) was used as the cleavage cocktail. Purification of the crude by preparative HPLC (eluent A 0.1% TFA in water, eluent B 0.1% TFA in CH_3CN/water 4/1 v/v, 0-10% eluent B in 2 min then 10-30% in 40 min, 25 °C, detection at 215 nm, 51 mL min^{-1}, C18 Xbridge 5 µm (19 × 100 mm) column) provided the titled peptide as a white solid after lyophilisation (146 mg, 30%).

Peptide **9a** was analyzed by LC-MS (Supplementary Figure 36) and MALDI-TOF MS (Supplementary Figure 37).

ESI (positive detection mode, Supplementary Figure 36) m/z = 1062.5 ($[M+2H]^{2+}$), 708.7 ($[M+3H]^{3+}$). Calcd. for M (average) 2124.5, found: 2123.5.

MALDI-TOF (matrix α-cyano-4-hydroxycinnamic acid, positive detection mode, Supplementary Figure 37) calcd. for $[M+H]^+$ (monoisotopic): 2123.9, found: 2124.1.

Synthesis of K1[177-205]-SEA^{off}

K1[177-205]-SEA^{off} (C(S*t*Bu)RNPRGEEGGPWC(S*t*Bu)FTSNPEVRYEVC(S*t*Bu)DIPQ-SEA^{off}) was synthesized on a 1.80 mmol scale as described in the general procedure presented in the Methods section. TFA/H_2O/TIS/thioanisole/EDT 90/1.67/5/1.67/1.67 v/v/v/v/v was used as cleavage cocktail. Purification of the crude by preparative HPLC (eluent A 0.1% TFA in water, eluent B 0.1% TFA in CH_3CN/water 4/1 v/v, 25 °C, detection at 215 nm, 51 mL min^{-1}, 0-28% eluent B in 5 min then 28-42% in 25 min, C18 Xbridge 5 µm (19 × 100 mm) column) provided the titled peptide as a white solid after lyophilisation (404 mg, 5%).

K1[177-205]-SEA^{off} was analyzed by LC-MS (Supplementary Figure 38) and MALDI-TOF MS (Supplementary Figure 39).

ESI (positive detection mode, Supplementary Figure 38) m/z = 1860.8 ($[M+2H]^{2+}$), 1241.8 ($[M+3H]^{3+}$); calcd. for M: 3721.4, found: 3721.0.

MALDI-TOF (matrix α-cyano-4-hydroxycinnamic acid, positive detection mode, Supplementary Figure 39) calcd. for $[M+H]^+$ (monoisotopic) 3719.6, found 3720.0.

Assembly of the linear K1 precursor ***9b***

The linear K1 precursor **9b** used for the synthesis of K1 cyclic derivatives was prepared as described in Supplementary Figure 7. Its sequence corresponds to HGF 128-205 using Uniprot numbering of amino acid residues (P14210).

Preparation of peptide thioester AcA-K1[128-148]-MPA (step 1 in Supplementary Figure 7)

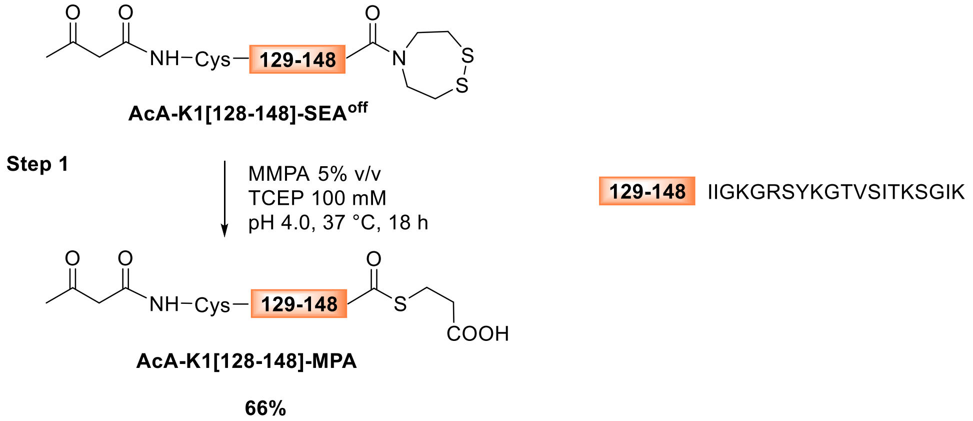

TCEP·HCl (286 mg) was dissolved in a mixture of MPA (0.5 mL) and H_2O (9.5 mL) and the pH was adjusted to 4.0 by addition of 6 M NaOH. AcA-K1[128-148]-SEA^{off} (23.1 mg, 7.79 µmol) was added to the solution (7.78 mL) and the mixture was stirred at 37 °C for 18 h. Note that a white solid precipitated during the reaction. The addition of Gn·HCl (7.35 g, final concentration: 6 M) and MPA (0.255 mL, final concentration: 5% v/v) did not allow the dissolution of the precipitate. The reaction mixture was acidified with AcOH (1.2 mL) and extracted with Et_2O (3 ×) to remove the MPA. The crude was purified by HPLC (eluent A 0.1% TFA in water, eluent B 0.1% TFA in acetonitrile/water 4/1 v/v, 30°C, detection at 215 nm, 6 mL min^{-1}, 0-15% eluent B in 5 min, then 15-35% eluent B in 60 min, C18 column) to give AcA-K1[128-148]-MPA as a white solid (15.1 mg, 66%).

AcA-K1[128-148]-MPA was analyzed by LC-MS (Supplementary Figure 40) and MALDI-TOF MS (Supplementary Figure 41).

ESI (positive detection mode, Supplementary Figure 40) m/z = 1185.0 ($[M+2H]^{2+}$), 790.2 ($[M+3H]^{3+}$), 593.1 ($[M+4H]^{4+}$); calcd. for M (average): 2368.8, found: 2368.2.

MALDI-TOF (matrix 2,5-dihydroxybenzoic acid, positive detection mode, Supplementary Figure 41) calcd. for $[M+H]^+$ (monoisotopic) 2368.3, found 2368.6.

Assembly of peptide thioester AcA-K1[128-176]-MPA (steps 2 & 3 in Supplementary Figure 7)

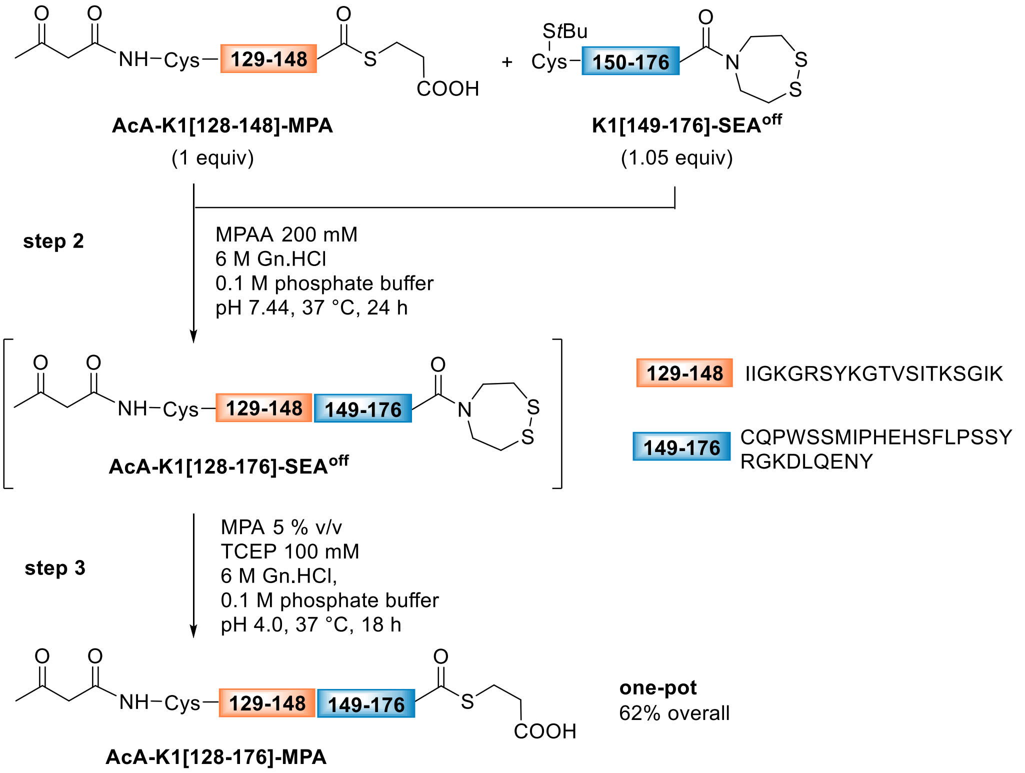

To a solution of Gn·HCl (574 mg) in 0.1 M, pH 7.4 phosphate buffer (600 µL) was added MPAA (33.4 mg, 200 mM) and the pH of the mixture was adjusted to 7.44 by addition of 6 M NaOH (80 µL). K1[149-176]-SEA^{off} (21.5 mg, 5.22 µmol, 1.05 equiv) and AcA-K1[128-148]-MPA (14.6 mg, 4.97 µmol, 1 equiv) were then successively dissolved in the MPAA solution (710 µL) and the mixture was stirred at 37 °C. The progress of the reaction leading to peptide AcA-K1[128-176]-SEA^{off} was followed by HPLC.

After completion of the NCL ligation (24h), SEA peptide AcA-K1[128-176]-SEA^{off} was directly converted into the corresponding MPA thioester by addition MPA (5% v/v) and TCEP (100 mM). To perform this reaction, TCEP (222 mg, 111 mM) and MPA (388 µL, 5.55 % v/v) were added to the solution of Gn·HCl (4.01 g) in 0.1 M, pH 7.4 phosphate buffer (3.81 mL) and the pH of the mixture was adjusted to 4.00 by addition of 6 M NaOH (600 µL). The previous ligation mixture containing AcA-K1[128-176]-SEA^{off} was then diluted with the MPA/TCEP solution (6.38 mL) and the pH was readjusted to 4.00 by addition of 6 N HCl (25 µL). In these conditions ([TCEP] = 100 mM, 5% MPA v/v), the reaction mixture was stirred at 37 °C for 18 h. After completion of the reaction, the mixture was acidified with AcOH (0.7 mL) and extracted with Et_2O (5 ×) to remove the MPA and the MPAA. The crude was purified by HPLC (eluent A 0.1% TFA in water, eluent B 0.1% TFA in acetonitrile/water 4/1 v/v, 50°C, detection

at 215 nm, 6 mL min^{-1}, 0-20% eluent B in 5 min, then 20-40% eluent B in 60 min, C18 column) to give AcA-K1[128-176]-MPA as a white solid after lyophilisation (20.8 mg, 62%).

AcA-K1[128-176]-MPA was analyzed by LC-MS (Supplementary Figure 42) and MALDI-TOF MS (Supplementary Figure 43).

ESI (positive detection mode, Supplementary Figure 42) m/z = 1422.8 ($[M+4H]^{4+}$), 1138.4 ($[M+5H]^{5+}$), 948.7 ($[M+6H]^{6+}$), 813.4 ($[M+7H]^{7+}$), 711.9 ($[M+8H]^{8+}$); calcd. for M: 5688.5, found: 5687.0.

MALDI-TOF (matrix 2,5-dihydroxybenzoic acid (DHB), positive detection mode, Supplementary Figure 43) calcd. for $[M+H]^+$ (average) 5689.5, found 5689.5.

Assembly of peptide **9b** (steps 4 & 5 in Supplementary Figure 7)

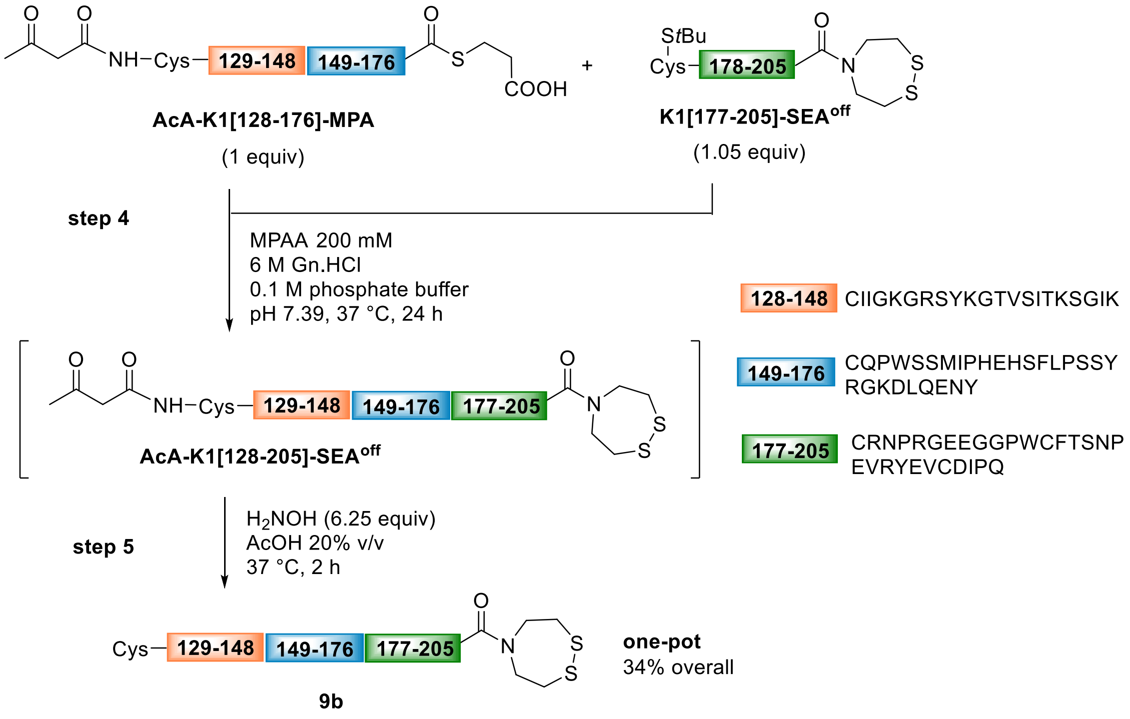

To a solution of Gn·HCl (574 mg) in 0.1 M, pH 7.4 phosphate buffer (600 µL) was added MPAA (33.7 mg) and the pH of the mixture was adjusted to 7.39 by addition of 6 M NaOH (75 µL). K1[177-205]-SEA^{off} (13.5 mg, 3.25 µmol, 1.05 equiv) and AcA-K1[128-176]-MPA (20.8 mg, 3.10 µmol, 1 equiv) were then successively dissolved in the MPAA solution (442 µL) and the mixture was stirred at 37 °C. The progress of the reaction was followed by HPLC.

After completion of the NCL ligation (24 h), the AcA protecting group of AcA-K1[128-205]-SEA^{off} was removed by reaction with NH_2OH. To perform this deprotection, the reaction mixture was acidified with AcOH (110 µL) and treated with a 0.71 M solution of NH_2OH·HCl in H_2O (27.3 µL, 19.4 µmol, 6.25 equiv). After 2 h stirring at 37 °C, the reaction mixture was diluted with H_2O (570 µL) and extracted with Et_2O (5 ×) to remove the MPAA. The crude was

further diluted with H_2O (1.14 mL, [AcOH] ≈ 5% v/v) and purified by HPLC (eluent A 0.1% TFA in water, eluent B 0.1% TFA in acetonitrile/water 4/1 v/v, 6 mL min^{-1}, 0-25% eluent B in 5 min, then 25-75% eluent B in 75 min, C18 column, 50°C, detection at 215 nm) to give the K1 analogue **9b** as a white solid after lyophilisation (10.9 mg, 34%).

Peptide **9b** was analyzed by LC-MS (Supplementary Figure 44) and MALDI-TOF MS (Supplementary Figure 45).

ESI (positive detection mode, Supplementary Figure 44) m/z = 1493.42 ($[M+6H]^{6+}$), 1280.25 ($[M+7H]^{7+}$), 1120.42 ($[M+8H]^{8+}$), 996.08 ($[M+9H]^{9+}$), 896.75 ($[M+10H]^{10+}$), 815.25 ($[M+11H]^{11+}$), 747.33 ($[M+12H]^{12+}$), 689.92 ($[M+13H]^{13+}$); calcd. for M (average): 8955.26, found: 8955.81.

MALDI-TOF (matrix 2,5-dihydroxybenzoic acid, positive detection mode, Supplementary Figure 45) calcd. for $[M+H]^+$ (average) 8956.2, found: 8957.4.

Peptide thioesters (see Supplementary Figure 4)

*Synthesis of peptide **8a***

Peptide **8a** was synthesized as described in the Methods section and analyzed by LC-MS (Supplementary Figure 46).

ESI (positive detection mode, Supplementary Figure 46) m/z calcd. for $[M+H]^+$ (monoisotopic mass): 1218.4, found: 1218.5.

*Synthesis of SetCys-(GS)$_2$-K(Biot)-(GS)$_2$A-MPA **8b***

SetCys-(GS)$_2$-K(Biot)-(GS)$_2$A-SEA^{on} was synthesized on 0.05 mmol scale as described in the general procedure presented in the Methods section. TFA/H_2O/TIS/thiophenol 92.5/2.5/2.5/2.5 v/v/v/v (5 mL) was used as cleavage cocktail. The SEA^{on} peptide which was recovered by precipitation from Et_2O/heptane was immediately converted into the corresponding MPA thioester using the following procedure without TCEP and Gn·HCl additives.

MPA (620 µL) was dissolved in water (11.9 mL) and the pH of the solution was adjusted to 4.0 by addition of 6 M NaOH. The SEA^{on} peptide was dissolved in this solution and the reaction mixture was stirred under inert atmosphere at 37 °C for 16 h. The reaction mixture was then acidified with 1.5% TFA in water (9 mL) and was extracted with Et_2O (5 ×) to remove the excess of MPA. Purification of the crude by semi-preparative HPLC (eluent A 0.1% TFA in water, eluent B 0.1% TFA in CH_3CN/water 4/1 v/v, 6 mL min^{-1}, 0-5% eluent B in 3 min then 5-30% eluent B in 32 min, C18 XBridge 5 µm (10 × 250 mm) column, detection at 215 nm, 30 °C) provided SetCys-(GS)$_2$-K(Biot)-(GS)$_2$A-MPA peptide **8b** as a white solid after lyophilisation (5.48 mg, 8%).

SetCys-$(GS)_2$-K(Biot)-$(GS)_2$A-MPA peptide **8b** was analyzed by LC-MS (Supplementary Figure 47) and MALDI-TOF MS (Supplementary Figure 48).

ESI (positive detection mode, Supplementary Figure 47) m/z calcd. for $[M+H]^+$ (monoisotopic mass): 1317.4, found: 1317.5.

MALDI-TOF (matrix α-cyano-4-hydroxycinnamic acid, positive detection mode, Supplementary Figure 48) calcd. for $[M+Na]^+$ (monoisotopic): 1339.4, found: 1339.2.

*Synthesis of SetCys-$(GS)_3$-K(Biot)-$(GS)_3$A-MPA **8c***

SetCys-$(GS)_3$-K(Biot)-$(GS)_3$A-SEA peptide **8c** was synthesized on 0.05 mmol scale as described in the general procedure presented in the Methods section. TFA/H_2O/TIS/thiophenol 92.5/2.5/2.5/2.5 v/v/v/v (5 mL) was used as cleavage cocktail. The SEA^{on} peptide which was recovered by precipitation from Et_2O/heptane was immediately converted into the corresponding MPA thioester using the following procedure without TCEP and Gn·HCl additives.

MPA (1.25 mL) was dissolved in water (23.75 mL) and the pH of the solution was adjusted to 4.0 by addition of 6 M NaOH. The SEA^{on} peptide was dissolved in this solution and the reaction mixture was stirred at 37 °C for 5.5 h. The reaction was monitored by HPLC and was stopped before completion to limit the formation of the side product which resulted from the slow cyclisation of SetCys-$(GS)_3$-K(Biot)-$(GS)_3$A-MPA peptide **8c**. The reaction mixture was then acidified with 10% TFA in water (until pH 3.2) and was extracted with Et_2O (5 ×) to remove the excess of MPA. Purification of the crude by semi-preparative HPLC (eluent A 0.1% TFA in water, eluent B 0.1% TFA in CH_3CN/water 4/1 v/v, 6 mL min^{-1}, 0-5% eluent B in 3 min then 5-35% eluent B in 32 min, C18 XBridge 5 μm (10 × 250 mm) column, detection at 215 nm, 30 °C) provided SetCys-$(GS)_3$-K(Biot)-$(GS)_3$A-MPA peptide **8c** as a white solid after lyophilisation (16.6 mg, 19%).

SetCys-$(GS)_3$-K(Biot)-$(GS)_3$A-MPA peptide **8c** was analyzed by LC-MS (Supplementary Figure 49) and MALDI-TOF MS (Supplementary Figure 50).

ESI (positive detection mode, Supplementary Figure 49) m/z calcd. for $[M+H]^+$ (monoisotopic mass): 1605.5, found: 1605.6.

MALDI-TOF (matrix α-cyano-4-hydroxycinnamic acid, positive detection mode, Supplementary Figure 50) calcd. for $[M+Na]^+$ (monoisotopic): 1627.5, found: 1627.5.

Synthesis of peptide thioester SetCys-CGVONAA-MPA

SetCys-CGVONAA-MPA peptide was synthesized on 0.05 mmol scale as described in the general procedure. TFA/H_2O/TIS/thiophenol/thioanisol 87.5/2.5/5/2.5/2.5 v/v/v/v/v (8 mL) was used as the cleavage cocktail. The SEA^{on} peptide which was recovered by precipitation from Et_2O/heptane was immediately converted into the corresponding MPA thioester using the following procedure.

MPA (0.625 mL) was dissolved in 11.9 mL of water and the pH of the solution was adjusted to 4.0 by addition of 6 M NaOH. The SEA^{on} peptide recovered after acidic cleavage was added to this solution of MPA. Due to the low solubility of the peptide, solid Gn·HCl (1.99 g) was added to the reaction mixture. Once the pH was readjusted to 4.0 by addition of 6 M NaOH, the reaction was stirred under inert atmosphere at 37 °C for 7 h. The reaction mixture was then acidified with AcOH (1 mL) and extracted with Et_2O (5 ×) to remove the MPA. Purification of the crude by semi-preparative HPLC (eluent A 0.1% TFA in water, eluent B 0.1% TFA in CH_3CN, 20 mL min^{-1}, 0-10% eluent B in 5 min then 10-25% in 30 min, C18 column, detection at 215 nm, 30 °C) provided SetCys-CGVONAA-MPA peptide as a white solid after lyophilisation (7.22 mg, 14% overall yield).

SetCys-CGVONAA-MPA peptide was analyzed by LC-MS (Supplementary Figure 51).

ESI (positive detection mode, Supplementary Figure 51) Calcd. for $[M+H]^+$ (monoisotopic): 944.21, found: 944.08.

Synthesis of ILKEPWHGA-MPAA peptide

ILKEPWHGA-MPAA peptide was synthesized from the corresponding peptide hydrazide using Liu's method.[7] The peptide was analyzed by LC-MS (Supplementary Figure 52).

ESI (positive detection mode, Supplementary Figure 52) Calcd. for [M+H]+ (monoisotopic): 1200.58, found: 1200.67.

*Synthesis of SetCys-K1[150-176]-MPA peptide **13***

The synthesis of SetCys-K1[150-176]-MPA peptide **13** is described in the Methods section.

SetCys-K1[150-176]-MPA peptide **13** was analyzed by LC-MS (Supplementary Figure 53).

ESI (positive detection mode, Supplementary Figure 53) m/z = 1766.25 ($[M+2H]^{2+}$), 1177.75 ($[M+3H]^{3+}$), 883.50 ($[M+4H]^{4+}$); calcd. for M (average): 3530.25, found: 3530.82.

Reactivity studies (Figure 2a)

Property 2. Ligation of SetCys peptide under low reducing conditions. Synthesis of SetCys amide peptide.

SetCys amide peptide RLKEPVHGA-SetCys-ALKEPVHGV-NH_2 was synthesized as described in the Methods section and characterized by LC-MS (Supplementary Figure 59) and MALDI-TOF MS (Supplementary Figure 60). The insertion of the SetCys amino acid into the peptide sequence was confirmed by MALDI-TOF MS-MS analysis (Supplementary Figure 61).

ESI (positive detection mode, Supplementary Figure 59) m/z = 1073.7 ([M+2H]2+), 716.0 ([M+3H]3+), 537.2 ([M+4H]4+); calcd. for M (average): 2144.4, found: 2145.1.

MALDI-TOF (matrix α-cyano-4-hydroxycinnamic acid, positive detection mode, Supplementary Figure 60) calcd. for $[M+H]^+$ (monoisotopic): 2145.1, found: 2145.2.

Property 4. Stability of SetCys under strong reducing conditions. Importance of ascorbate (see Supplementary Figure 69)

To a solution of Gn·HCl (287 mg) in pH 7.4 0.1 M phosphate buffer (300 μL) were added TCEP·HCl (14.3 mg) and MPAA (16.8 mg) and the pH of the mixture was adjusted to 6.0 by addition of 6 M NaOH. SetCys peptide **1** (0.48 mg, 0.32 μmol) was then dissolved in the buffered solution (320 μL) and the reaction mixture was stirred at 37 °C. The progress of the reaction was monitored by HPLC. For each point, an 8 μL aliquot was taken from the reaction mixture and quenched by adding 10% AcOH in water (100 μL). The sample was then extracted with Et_2O to remove MPAA prior to HPLC analysis.

At pH 6.0 and at the beginning of the reaction, the deselenization of SetCys peptide **1** into the corresponding *N*-ethyl cysteinyl derivative (Et-CALKEPVHGV-NH_2) dominates in the mixture (Supplementary Figure 70a). At longer reaction times, the SetCys peptide **1** also yields the Cys peptide by losing its selenoethyl arm (Supplementary Figure 70b). An increase of the reaction mixture pH (from 6.0 to 7.2) favors the deselenization process and leads nearly exclusively to the formation of the *N*-ethylcysteinyl peptide product (Supplementary Figure 71).

The Et-CALKEPVHGV-NH_2 peptide produced by deselenization of SetCys peptide **1** under the above conditions was isolated by HPLC (Supplementary Figure 72) and its sequence was confirmed by MALDI MS-MS analysis (Supplementary Figure 73, Supplementary Figure 74).

Property 5.

NCL under strong reducing conditions (peptide alkyl thioester, TCEP/ascorbate, MPAA)

When SetCys peptide **1** and peptide thioester **4** were reacted in the presence of the TCEP/MPAA, native peptide **6** was produced with 64% yield (Supplementary Figure 75).

Peptide **6** was synthesized as described in the Methods section and characterized by LC-MS (Supplementary Figure 77) and MALDI-TOF MS (Supplementary Figure 78). Furthermore, the replacement of the SetCys residue by a Cys residue during the ligation was confirmed by MALDI-TOF-TOF sequencing of peptide **6** (Supplementary Figure 79). Finally, the determination of the optical purity for the Ala residue involved in the newly formed junction was performed by chiral GC-MS analysis after acid hydrolysis of peptide **6** in deuterated acid. This analysis indicated that the Ala residue did not epimerize to a significant extent during the NCL: 1.69% D-Ala content was measured in the isolated peptide **6**.

ESI (positive detection mode, Supplementary Figure 77) m/z = 1020.4 ($[M+2H]^{2+}$), 680.7 ($[M+3H]^{3+}$), 510.7 ($[M+4H]^{4+}$); calcd. for M (average): 2039.4, found 2039.2.

MALDI-TOF (matrix α-cyano-4-hydroxycinnamic acid, positive detection mode, Supplementary Figure 78) calcd. for $[M+H]^+$ (monoisotopic): 2039.1, found: 2039.2.

Besides peptide **6**, RLKEPVHGA-OH (0.97 mg, Supplementary Figure 80) and CALKEPVHGV-NH_2 (1.12 mg, Supplementary Figure 81) were isolated as side products from the reaction mixture. Peptide RLKEPVHGA-OH results from the partial hydrolysis of the peptidyl MPA-thioester **4** whereas the cysteinyl peptide CALKEPVHGV-NH_2 results from the decomposition of the unreacted SetCys peptide **1**.

NCL under strong reducing conditions (peptide aryl thioester, TCEP/ascorbate, no added MPAA)

When SetCys peptide **1** and peptidyl MPAA-thioester ILKEPWHGA-MPAA were reacted in the presence of the TCEP, peptide ILKEPWHGA-CALKEPVHGV-NH_2 was produced with 40% isolated yield (Supplementary Figure 82). Monitoring of the reaction by UPLC-MS (Supplementary Figure 83) shows the transient accumulation of the mono- and di-ligated species A and B, the latter being quickly converted into the mono-ligated compounds A. The intermediate A is then slowly converted into the final peptide ILKEPWHGACALKEPVHGV-NH_2 featuring a cysteine as a junction residue.

The following procedure was used to ligate SetCys peptide **1** and peptidyl MPAA-thioester ILKEPWHGA-MPAA. To a solution of Gn·HCl (287 mg) in 0.1 M, pH 7.4 phosphate buffer (300 μL) were added sodium ascorbate (9.9 mg) and TCEP·HCl (14.3 mg) and the pH of the mixture was adjusted to 7.27 by addition of 6 M NaOH. SetCys peptide **1** (2.00 mg, 1.33 μmol, 1.2 equiv) and peptidyl MPAA-thioester ILKEPWHGA-MPAA (1.71 mg, 1.10 μmol, 1 equiv) were then successively dissolved in this solution (139 μL) and the reaction mixture was stirred at 37 °C. The mixture was then acidified with 7.5% AcOH in water (8 mL) and extracted with Et_2O (5 ×). Purification of the crude by HPLC (eluent A 0.1% TFA in water, eluent B 0.1% TFA in acetonitrile, gradient: 0-10% eluent B in 5 min then 10-25% in 25 min, 6 mL min^{-1},

C18 column, rt, detection at 215 nm,) provided ILKEPWHGA-CALKEPVHGV-NH_2 peptide as a white solid after lyophilisation (1.17 mg, 40%).

ILKEPWHGA-CALKEPVHGV-NH_2 peptide was analyzed by LC-MS (Supplementary Figure 84).

ESI (positive detection mode, Supplementary Figure 84) MS trace: m/z = 1853.42 ([M+2H]2+), 927.58 ([M+3H]3+), 618.83 ([M+4H]4+). Calcd. for M (average): 2083.49, found: 2082.99.

NCL under strong reducing conditions (peptide alkyl thioester, DTT, MPAA)

When SetCys peptide **1** and peptide thioester **4** were reacted in the presence of the DTT, native peptide **6** was produced with 55% yield (Supplementary Figure 85). The following procedure was used to synthesize peptide **6** in these reductive conditions. To a solution of Gn·HCl (574 mg) in pH 7.4 0.1 M phosphate buffer (600 µL) were added DTT (15.4 mg) and MPAA (33.6 mg) and the pH of the mixture was adjusted to 7.24 by addition of 6 M NaOH. Peptide thioester **4** (6.04 mg, 3.89 µmol, 1 equiv) and SetCys peptide **1** (7.00 mg, 4.67 µmol, 1.2 equiv) were then successively dissolved in this solution (487 µL) and the reaction mixture was stirred at 37 °C. The reaction was monitored by HPLC (Supplementary Figure 86). After completion of the reaction (4 days), the mixture was then acidified with 10% AcOH in water (8 mL) and extracted with Et_2O (5 ×) to remove the MPAA. Purification of the crude by HPLC (eluent A: 0.1% TFA in water, eluent B: 0.1% TFA in acetonitrile, gradient: 0-10% eluent B in 5 min then 10-30% eluent B in 30 min, 6 mL min^{-1}, C18 column, rt, detection at 215 nm) provided peptide **6** as a white solid after lyophilisation (5.83 mg, 55%).

Reactivity studies (Figure 2b)

The SutCys-ALKEPVHGV-NH_2 peptide used as model compound in this study was synthesized according to Ruff *et al*.[8]

Stability of SutCys-ALKEPVHGV-NH_2 peptide under strong reducing conditions

The following procedure was used to investigate the stability of the SutCys-ALKEPVHGV-NH_2 peptide under strong reducing conditions (Supplementary Figure 87). To a solution of Gn·HCl (287 mg) in 0.1 M phosphate buffer (300 µL) were added TCEP·HCl (14.3 mg), sodium ascorbate (9.9 mg) and MPAA (16.8 mg) and the pH of the mixture was adjusted to 6.95 by addition of 6 M NaOH. SutCys-ALKEPVHGV-NH_2 peptide (0.23 mg, 0.16 µmol) was then dissolved in this solution (160 µL) and the reaction mixture was stirred at 37 °C.The reaction was monitored by HPLC (Supplementary Figure 88). For each point, an 8 µL aliquot was taken from the reaction mixture and quenched by adding 10% AcOH in water (100 µL). The sample was then extracted with Et_2O to remove MPAA prior to HPLC analysis.

The HPLC monitoring showed that the SutCys residue didn't lose its sulfanylethyl arm, even after several days of incubation in presence of TCEP. Neither increasing (from 6.95 to 7.92) nor decreasing (from 6.95 to 6.06) the pH of the reaction mixture changed the outcome of the reaction.

NCL under low reducing conditions: synthesis of SutCys amide peptide

As described in Supplementary Figure 89, the SutCys amide peptide RLKEPVHGA-SutCys-ALKEPVHGV-NH_2 was synthesized by ligating peptidyl MPA-thioester **4** and SutCys-ALKEPVHGV-NH_2 peptide under low reducing conditions. The final peptide featuring the SutCys unit in the form of a cyclic disulfide at the ligation junction was recovered as the only product of the ligation (Supplementary Figure 90) and was isolated with 53% yield using the following procedure. To a solution of Gn·HCl (1.03 g) in 0.1 M, pH 7.4 phosphate buffer (1.08 mL) was added MPAA (60.6 mg) and the pH of the mixture was adjusted to 7.21 by addition of 6 M NaOH. SutCys-ALKEPVHGV-NH_2 peptide (22.4 mg, 15.4 µmol, 1.2 equiv) and RLKEPVHGA-MPA peptide **4** (20.0 mg, 12.9 µmol, 1 equiv) were then successively dissolved in this solution (1.61 mL) and the reaction mixture was stirred at 37 °C for 24 h. The reaction was monitored by LC-MS (Supplementary Figure 90). The mixture was then acidified with 10% AcOH in water (23 mL) and extracted with Et_2O (5 ×) to remove the MPAA. Purification of the crude by HPLC (eluent A: 0.1% TFA in water, eluent B: 0.1% TFA in acetonitrile/water 4/1 v/v, 50°C, detection at 215 nm, 6 mL min^{-1}, 0-10% eluent B in 5 min, then 10-35% eluent B in 35 min, C18XBridge column) provided RLKEPVHGA-SutCys-ALKEPVHGV-NH_2 peptide as a white solid after lyophilisation (19.2 mg, 53%).

Peptide RLKEPVHGA-SutCys-ALKEPVHGV-NH_2 was analyzed by LC-MS (Supplementary Figure 91) and MALDI-TOF (Supplementary Figure 92).

ESI (positive detection mode, Supplementary Figure 91) m/z 1049.6 ([M+2H]2^{+}), 700.0 ([M+3H]$^{3+}$); calcd. for M (average): 2097.5, found: 2097.2.

MALDI-TOF (matrix α-cyano-4-hydroxycinnamic acid, positive detection mode, Supplementary Figure 92) calcd. for [M+H]$^{+}$ (monoisotopic): 2097.1, found: 2097.5.

NCL under strong reducing conditions

The ligation of peptidyl MPA-thioester **4** and SutCys-ALKEPVHGV-NH_2 peptide under strong reducing conditions is described in Supplementary Figure 93 and the following procedure was used to perform this reaction. To a solution of Gn·HCl (573 mg) in 0.1 M phosphate buffer (600 µL) were successively added TCEP (53.7 mg) and MPAA (33.6 mg) and the pH of the mixture was adjusted to 7.2 by addition of 6 M NaOH. SutCys-ALKEPVHGV-NH_2 (8.00 mg, 5.51 µmol, 1.25 equiv) and peptide thioester **4** (6.83 mg, 4.40 µmol, 1 equiv) were then successively dissolved in this solution (550 µL) and the reaction mixture was maintained at 37 °C.

After 48 h reaction, a 2 µL aliquot was taken from the reaction mixture and quenched by adding 10% AcOH in water (100 µL). The sample was then extracted with Et_2O to remove MPAA prior to LC-MS analysis (Supplementary Figure 94). The ligation product (t_R = 11.48 min) featuring the SutCys unit in its reduced, ring-opened form was detected in the crude, in mixture with the unreacted SutCys-ALKEPVHGV-NH_2 peptide (t_R = 10.10 min) and with the RLKEPVHGA-OH peptide (t_R = 8.37 min) resulting from the hydrolysis of the starting peptidyl thioester.

The ligation product was isolated in the form of the cyclic disulfide after oxidation with iodine. Experimentally, the ligation mixture was acidified with 10% AcOH in water (3.5 mL) and extracted with Et_2O (5 ×) to remove the MPAA. A solution of iodine in DMSO (79 mg mL^{-1}) was then added dropwise until persistency of the yellow color of iodine. The excess of iodine was then quenched with a solution of DTT in 20% AcOH (10 mg mL^{-1}) and the mixture was diluted with water (final volume: 10 mL). Purification of the crude by HPLC (eluent A: 0.1% TFA in water, eluent B: 0.1% TFA in acetonitrile/water 4/1 v/v, 50°C, detection at 215 nm, 6 mL min^{-1}, 0-10% eluent B in 5 min, then 10-30% eluent B in 30 min, C18XBridge column) provided RLKEPVHGA-SutCys-ALKEPVHGV-NH_2 as a white solide after lyophilisation (3.70 mg, 30%).

Mechanistic studies (Figure 3)

Participation of Cys thiol in the ease of 2-selenoethyl arm removal. Control experiment with the SetAla peptide (Figure 3d)

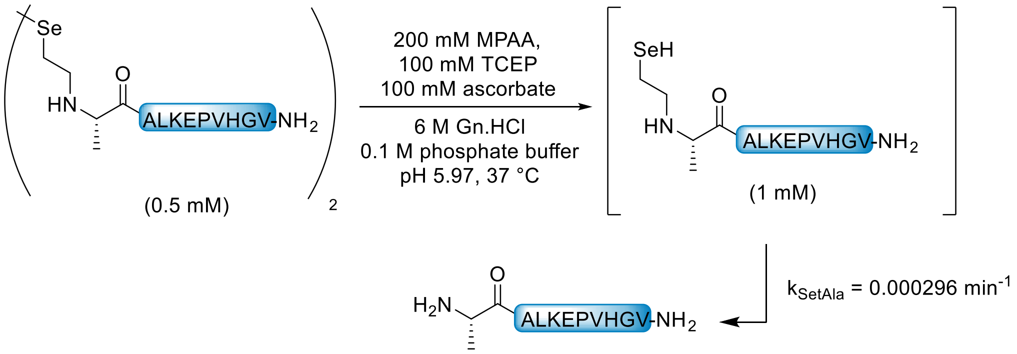

To a solution of Gn·HCl (287 mg) in 0.1 M pH 7.4 phosphate buffer (300 µL) were added TCEP·HCl (14.3 mg), sodium ascorbate (9.9 mg) and MPAA (16.8 mg) and the pH of the mixture was adjusted to 5.97 by addition of 6 M NaOH. Diselenide precursor of SetAla peptide (SetAla-ALKEPVHGV-NH_2)$_2$ (0.47 mg, 0.16 µmol) was then dissolved in the buffered solution (320 µL) and the reaction mixture was stirred at 37 °C. The progress of the reaction was monitored by HPLC (Supplementary Figure 96). For each point, an 8 µL aliquot was taken from the reaction mixture and quenched by adding 10% AcOH in water (100 µL). The sample was then extracted with Et_2O to remove MPAA prior to HPLC analysis. The kinetic data were then fitted to a pseudo-first order kinetic law using KinteK Explorer Software*TM* (Supplementary Figure 97). The AALKEPVHGV-NH_2 peptide produced by decomposition of SetAla peptide

during the reaction was purified by HPLC and its sequence was confirmed by MS-MS analysis (Supplementary Figure 98 and Supplementary Figure 99).

Ligation with SetAla peptide

The reaction described in Supplementary Figure 100 was tested to investigate the ligation of SetAla peptide and peptidyl PMA-thioester **4** under strong reducing conditions (TCEP + MPAA). The following procedure in which the SetAla peptide was generated *in situ* by reduction of the corresponding diselenide was used to perform this reaction. To 500 µL of a 6 M solution of Gn·HCl in 0.1 M pH 7.4 phosphate buffer were successively added MPAA (16.8 mg), TCEP·HCl (14.3 mg) and sodium ascorbate (9.9 mg). The pH of the mixture was adjusted to 7.28 by addition of 6 M NaOH (61,5 µL). In 140 µL of the previous solution were dissolved the diselenide precursor of SetAla peptide (SetAla-ALKEPVHGV-NH_2)$_2$ (1.97 mg, 0.671 µmol, 0.6 equiv) and peptidyl MPA-thioester **4** (1.73 mg, 1.12 µmol, 1 equiv). The reaction mixture was stirred at 37 °C and monitored by HPLC (Supplementary Figure 101). For each point, a 2 µL aliquot was taken from the reaction mixture and quenched by adding 10% AcOH in water (100 µL). The sample was then extracted with Et_2O to remove MPAA prior to HPLC analysis. The HPLC monitoring of the reaction showed a complete decomposition of the starting material: conversion of SetAla peptide into the Ala peptide and hydrolysis of the MPA-thioester **4**. No native ligated product (RLKEPVHGAAALKEPVHGV-NH_2) was observed although a ligated seleno peptide (RLKEPVHGA-SetAla-ALKEPVHGV-NH_2) was transiently produced during the course of the reaction.

Kinetic model of SetCys-mediated NCL under strong reducing conditions (Figure 4)

Generalities

Kintek Global Kinetic Explorer Version 8.0.190823 was used for kinetic modelization.

The standard deviation of each experimental point was estimated based upon fitting the experimental dataset with an analytical function so as to determine an average sigma value further used for numerical data fitting.

Fitting to a given model is achieved by non linear regression analysis based upon an iterative search to find a set of reaction parameters that gives a minimum χ^2. Standard errors produced by non linear regression fitting are estimated based upon the covariance matrix.

A one-tailed *p*-value test is made to estimate the probability for observed χ^2 of being within a range attributable to random errors expected from the measured sigma value. The process is completed by careful visual examination of the fit and critical evaluation of the model.

Peptide and protein assemblies

Assembly of conotoxin OIVA sequence (see Supplementary Figure 5)

To a solution of Gn·HCl (287 mg) in 0.1 M, pH 7.4 phosphate buffer (300 µL) was added MPAA (16.8 mg) and the pH of the mixture was adjusted to 7.25 by addition of 6 M NaOH. Peptide CHOCVCKNTC-NH_2 (2.50 mg, 1.70 µmol, 1 equiv) and peptide SetCys-CGVONAA-MPA (1.80 mg, 1.70 µmol, 1 equiv) were successively dissolved in the MPAA solution (472 µL) and the mixture was stirred at 37 °C. The progress of the ligation leading to the elongated SeCys peptide intermediate was followed by HPLC (Supplementary Figure 105a).

After completion of the NCL ligation (2 h), the second step of the process leading to the conversion of the SetCys residue into Cys residue was induced by addition of TCEP. To a solution of Gn·HCl (287 mg) in 0.1 M, pH 7.4 phosphate buffer (300 µL) were added TCEP·HCl (28.6 mg), sodium ascorbate (9.9 mg) and MPAA (16.8 mg) and the pH of the mixture was adjusted to 5.85 by addition of 6 M NaOH. The previous ligation mixture containing the elongated SetCys peptide intermediate was then diluted with the solution of TCEP (472 µL) and the pH of the new mixture was readjusted to 5.98 by addition of 6 M HCl. The reaction mixture was then stirred at 37 °C for 24 h. After completion of the reaction (Supplementary Figure 105b), the mixture was acidified with 7.5 % AcOH in water (8 mL) and extracted with Et_2O (5 ×) to remove the MPAA. The crude was purified by HPLC (eluent A: 0.1% TFA in water, eluent B 0.1% TFA in acetonitrile, 0-5% eluent B in 5 min then 5-20% eluent B in 30 min, 6 mL min^{-1}, C18 column, rt, detection at 215 nm) to give the linear sequence of conotoxin OVIA as a white solid after lyophilization (1.48 mg, 40%).

Conotoxin OVIA linear peptide was analyzed by LC-MS (Supplementary Figure 106a and b) and MALDI-TOF MS (Supplementary Figure 106c). The MALDI-TOF MS-MS sequencing of the ion at *m/z* 1853.65 (Supplementary Figure 106d) confirms the Ala-Cys junction formation by NCL as well as the TCEP-induced conversion of the N-terminal SetCys residue into Cys.

ESI (positive detection mode, Supplementary Figure 106b) Calcd. for $[M+H]^+$ (monoisotopic): 1853.70, found: 1853.42.

MALDI-TOF (matrix α-cyano-4-hydroxycinnamic acid, positive detection mode, Supplementary Figure 106) calcd. for $[M+H]^+$ (monoisotopic): 1853.70, found: 1853.65.

Synthesis of cyclic peptides (see Supplementary Figure 6)

Synthesis of cyclic peptide 10. To a solution of Gn·HCl (287 mg) in 0.1 M, pH 7.4 phosphate buffer (300 µL) was added MPAA (16.8 mg) and the pH of the mixture was adjusted to 7.20 by addition of 6 M NaOH. Peptide **9a** (4.50 mg, 1.82 µmol, 1 equiv) and peptide **8a** (2.84 mg, 1.82 µmol, 1 equiv) were then successively dissolved in the MPAA solution (228 µL) and the mixture was stirred at 37 °C. The progress of the reaction leading to the bifunctional peptide intermediate was followed by HPLC (Supplementary Figure 107a).

After completion of the NCL ligation (3 h), the second step of the process leading to the cyclisation of the peptide was induced by addition of TCEP. To a solution of Gn·HCl (1.32 g) in 0.1 M, pH 7.4 phosphate buffer (1.38 mL) were added TCEP·HCl (87.7 mg), sodium ascorbate (60.6 mg) and MPAA (77.4 mg) and the pH of the mixture was adjusted to 5.51 by addition of 6 M NaOH. The previous ligation mixture containing the bifunctional peptide intermediate was then diluted with the solution of TCEP (2.05 mL) and the mixture was stirred at 37 °C for 22 h. After completion of the reaction (Supplementary Figure 107c), the mixture was acidified with 10 % AcOH in water (5 mL) and diluted with water (2.5 mL). The mixture was then extracted with Et_2O (5 ×) to remove the MPAA and purified by HPLC (eluent A 0.1% TFA in water, eluent B 0.1% TFA in acetonitrile/water 4/1 v/v, 50°C, detection at 215 nm, 6 mL min^{-1}, 0-10% eluent B in 5 min, then 10-35% eluent B in 30 min, C18XBridge column) to give the cyclic peptide **10** as a white solid after lyophilization (1.65 mg, 27%).

Peptide **10** was analyzed by LC-MS (Supplementary Figure 108) and MALDI-TOF (Supplementary Figure 109).

ESI (positive detection mode, Supplementary Figure 108) m/z 1455.3 ($[M+2H]^{2+}$), 970.0 ($[M+3H]^{3+}$), 727.8 ($[M+4H]^{4+}$); calcd. for M (average): 2907.3, found: 2907.1.

MALDI-TOF (matrix α-cyano-4-hydroxycinnamic acid, positive detection mode, Supplementary Figure 109) calcd. for $[M+H]^+$ (monoisotopic): 2906.4, found: 2906.1.

Synthesis of cyclic peptide 11. To a solution of Gn·HCl (287 mg) in 0.1 M, pH 7.4 phosphate buffer (300 μL) was added MPAA (16.8 mg) and the pH of the mixture was adjusted to 7.20 by addition of 6 M NaOH. Peptide **9a** (4.50 mg, 1.82 μmol, 1 equiv) and peptide **8b** (2.61 mg, 1.82 μmol, 1 equiv) were then successively dissolved in the MPAA solution (228 μL) and the mixture was stirred at 37 °C. The reaction yielding the bifunctional peptide intermediate was monitored by HPLC (Supplementary Figure 110a).

After completion of the NCL ligation (3.5 h), the second step of the process leading to the cyclisation of the peptide was induced by addition of TCEP. To a solution of Gn·HCl (1.32 g) in 0.1 M, pH 7.4 phosphate buffer (1.38 mL) were added TCEP·HCl (87.7 mg), sodium ascorbate (60.6 mg) and MPAA (77.4 mg) and the pH of the mixture was adjusted to 5.50 by addition of 6 M NaOH. The previous ligation mixture containing the bifunctional peptide intermediate was then diluted with the solution of TCEP (2.05 mL) and the mixture was stirred at 37 °C for 4 days. After completion of the reaction (Supplementary Figure 110b), the mixture was acidified with 10 % AcOH in water (7.5 mL) and diluted with water (10 mL). The crude was then extracted with Et_2O (5 ×) to remove the MPAA and purified by HPLC (eluent A 0.1% TFA in water, eluent B 0.1% TFA in acetonitrile/water 4/1 v/v, 50°C, detection at 215 nm, 6 mL min^{-1}, 0-5% eluent B in 3 min, then 5-35% eluent B in 32 min, C18XBridge column) to give the cyclic peptide **11** as a white solid after lyophilization (1.65 mg, 28%).

Peptide **11** was analyzed by LC-MS (Supplementary Figure 111) and MALDI-TOF (Supplementary Figure 112).

ESI (positive detection mode, Supplementary Figure 111) m/z = 1503.7 ($[M+2H]^{2+}$), 1002.9 ($[M+3H]^{3+}$); calcd for M 3006.3, found 3005.9.

MALDI-TOF analysis of cyclic peptide **11** (matrix α-cyano-4-hydroxycinnamic acid, positive detection mode, Supplementary Figure 112) calcd. for $[M+H]^+$ (monoisotopic): 3005.3, found: 3005.7.

Synthesis of cK1-1. The synthesis of **cK1-1** is described in the Methods section. The assembly was monitored by LC-MS (Supplementary Figure 113). **cK1-1** was analyzed by LC-MS (Supplementary Figure 114) and proteomic analysis (Supplementary Figure 115, Supplementary Figure 116).

ESI (positive detection mode, Supplementary Figure 114) m/z = 1655.25 ($[M+6H]^{6+}$), 1419.00 ($[M+7H]^{7+}$), 1241.83 ($[M+8H]^{8+}$), 1104.00 ($[M+10H]^{10+}$); calcd. for M (average): 9925.19, found: 9926.28.

The cyclic structure of **cK1-1** was verified by proteomic analysis (Supplementary Figure 115, Supplementary Figure 116). In this procedure, the free Cys residues of **cK1-1** had to be masked by alkylation before performing the trypsin digestion of the polypeptide.

Alkylation step. **cK1-1** (50 µg) was treated with a solution of iodoacetamide in 0.025 M ammonium bicarbonate (10 mg mL^{-1}, 50 µL) for 15 min. The alkylation step was monitored by MALDI-TOF MS (Supplementary Figure 115).

Trypsin digestion. Trypsin (0.1 mg mL^{-1}, 0.5 µL) was added to the mixture to cleave the alkylated cyclic peptide. The fragments resulting from the enzymatic cleavage were identified by MALDI-TOF MS (Supplementary Figure 116a) and the MS-MS sequencing of the ion at m/z 2756.36 confirmed the Gln-Cys and Ala-Cys junctions formed during the cyclisation process (Supplementary Figure 116b).

Synthesis of cK1-2. To a solution of Gn·HCl (287 mg) in 0.1 M, pH 7.4 phosphate buffer (300 µL) was added MPAA (16.8 mg) and the pH of the mixture was adjusted to 7.26 by addition of 6 M NaOH (31 µL). Peptide **9b** (5.00 mg, 0.479 µmol, 1 equiv) and peptide **8c** (0.822 mg, 0.479 µmol, 1 equiv) were successively dissolved in the MPAA solution (120 µL) and the mixture was stirred at 37 °C. The progress of the ligation leading to the bifunctional peptide intermediate was followed by HPLC (Supplementary Figure 117a).

After completion of the NCL ligation (2 h), the second step of the process leading to the cyclisation of the peptide was induced by addition of TCEP in the reaction mixture. To a solution of Gn·HCl (1.72 g) in 0.1 M, pH 7.4 phosphate buffer (1.80 mL) were added TCEP·HCl (90.1 mg), sodium ascorbate (62.4 mg) and MPAA (101 mg) and the pH of the mixture was adjusted to 5.52 by addition of 6 M NaOH (260 µL). The previous ligation mixture containing the bifunctional peptide intermediate was then diluted with this solution of TCEP

(2.28 mL) and the reaction mixture was stirred at 37 °C for 40 h. After completion of the reaction (Supplementary Figure 117b), the mixture was acidified with AcOH (0.30 mL) and extracted with Et_2O (5 ×) to remove the MPAA. The crude was further diluted with water (9 mL) and purified by HPLC (eluent A 0.1% TFA in water, eluent B 0.1% TFA in acetonitrile/water 4/1 v/v, 50°C, detection at 215 nm, 6 mL min^{-1}, 0-20% eluent B in 5 min, then 20-45% eluent B in 60 min, C18XBridge column) to give the cyclic peptide **cK1-2** as a white solid after lyophilization (2.04 mg, 37%).

cK1-2 was analyzed by LC-MS (Supplementary Figure 118) and proteomic analysis (Supplementary Figure 119, Supplementary Figure 120).

ESI (positive detection mode, Supplementary Figure 118) m/z = 1702.83 ($[M+6H]^{6+}$), 1459.92 ($[M+7H]^{7+}$), 1277.67 ($[M+8H]^{8+}$), 1135.83 ($[M+9H]^{9+}$), 1022.33 ($[M+10H]^{10+}$); calcd. for M: 10213.45, found: 10212.71.

The cyclic structure and sequence of **cK1-2** were verified by proteomic analysis (Supplementary Figure 119, Supplementary Figure 120). In this procedure, the free Cys residues of **cK1-2** had to be masked by alkylation before performing the trypsin digestion of the polypeptide.

Alkylation step. **cK1-2** (20 µg) was dissolved in a solution of dithiothreitol in 0.025 M ammonium bicarbonate (1 mg mL^{-1}, 20 µL) and the mixture was treated with iodoacetamide (10 mg mL^{-1} in 0.025 M ammonium bicarbonate, 20 µL) for 15 min. The alkylation step was monitored by MALDI-TOF mass spectrometry (Supplementary Figure 119).

Trypsin digestion. Trypsin (0.1 mg mL^{-1}, 1 µL) was added to the mixture to cleave the alkylated cyclic peptide. The fragments resulting from the enzymatic cleavage were identified by MALDI-TOF MS (Supplementary Figure 120a) and the MS-MS sequencing of the ion at m/z 3044.05 confirmed the Gln-Cys and Ala-Cys junctions formed during the cyclisation process (Supplementary Figure 120b).

Synthesis of Linear K1 polypeptide 15. Linear K1 polypeptide **15** was synthesized following the procedures A and B described in the Methods section.

Linear K1 polypeptide **15** was analyzed by LC-MS (Supplementary Figure 123) and MALDI-TOF (Supplementary Figure 124).

ESI (positive detection mode, Supplementary Figure 123) m/z = 1666.17 ($[M+6H]^{6+}$), 1428.42 ($[M+7H]^{7+}$), 1250.08 ($[M+8H]^{8+}$), 1111.25 ($[M+9H]^{9+}$), 1000.25 ($[M+10H]^{10+}$), 909.42 ($[M+11H]^{11+}$), 833.75 ($[M+12H]^{12+}$), 769.58 ($[M+13H]^{13+}$), 714.67 ($[M+14H]^{14+}$), 667.17 ($[M+15H]^{15+}$). Calcd. for M (average): 9993.40, found: 9992.14.

MALDI-TOF (matrix: 2,5-dihydroxybenzoic acid, positive detection mode, Supplementary Figure 124) calcd. for $[M+H]^+$ (average): 9994.4, found: 9993.1.

The sequence of polypeptide **15** was verified by proteomic analysis (Supplementary Figure 125, Supplementary Figure 126). In this procedure, the free Cys residues of polypeptide **15** had to be masked by alkylation before performing the trypsin digestion of the peptide sequence.

Alkylation step. Synthetic K1 polypeptide **15** (20 µg) was treated with a solution of iodoacetamide in 0.025 M ammonium bicarbonate (1 mg mL^{-1}, 20 µL) for 15 min. The alkylation step was monitored by MALDI-TOF MS (Supplementary Figure 125).

Trypsin digestion. Trypsin (0.5 mg mL^{-1}, 2 µL) was added to the mixture to cleave the alkylated linear polypeptide. Analysis of the fragments by MALDI-TOF MS confirms the Tyr-Cys junction formed by NCL and the conversion of the SetCys residue into Cys residue during the second ligation step (Supplementary Figure 126).

Protein folding

cK1-1 and **cK1-2** were folded following the procedure described in the Methods section.

The folded protein **cK1-1f** was analyzed by LC-MS (Supplementary Figure 129).

ESI (positive detection mode, Supplementary Figure 129) m/z = 1654.17 ($[M+6H]^{6+}$), 1418.17 ($[M+7H]^{7+}$), 1241.08 ($[M+8H]^{8+}$), calcd for M (average): 9919.13, found: 9919.95.

The folded protein **cK1-2f** was analyzed by LC-MS (Supplementary Figure 130).

ESI (positive detection mode, Supplementary Figure 130) m/z = 1702.33 ($[M+6H]^{6+}$), 1459.25 ($[M+7H]^{7+}$), 1277.08 ($[M+8H]^{8+}$), 1135.33 ($[M+9H]^{9+}$); calcd for M (average): 10207.39, found 10208.34.

The characterization of disulfide bridge patterns of **cK1-1f** and **cK1-2f** is given in Supplementary Figure 131. These results show that the disulfide bridge pattern of the K1 domain (Cys^{149}-Cys^{189}, Cys^{128}-Cys^{206}, Cys^{177}-Cys^{201}) is not altered by the biotinylated linkers introduced to cyclize the K1 domain.

Experimental determination of disulfide bridge patterns was achieved by enzymatic digestion of **cK1-1f** and **cK1-2f** domains and identification of the peptide fragments by mass spectrometry using non reducing conditions. Fragments obtained by digestion with trypsin permitted the direct assignment of Cys^{149}-Cys^{189} disulfide bond whereas further digestion with the endoproteinase Asp-N was required to establish the formation of Cys^{128}-Cys^{206} and Cys^{177}-Cys^{201} disulfide bonds.

Trypsin digestion. Prior to use, **cK1-1f** and **cK1-2f** were purified by HPLC (eluent A 0.1% TFA in water, eluent B 0.1% TFA in CH_3CN/water 4/1 by v/v, C18 XBridge BEH 300Å 3.5 µm 2.1×150 mm column, 50 °C, gradient 0-10% B in 5 min then 10-80% in 5 min, 0.4 mL min^{-1}, detection at 215 nm). For each protein, the HPLC fractions containing the folded structure were collected and dried under vacuum and the residue was then dissolved in a solution of trypsin (5 µL, 50 ng/µL) in 20 mM NH_4HCO_3. The digestion was carried out at 37 °C and the progress of

the enzymatic reaction was monitored by MALDI-TOF MS. Two main fragments were detected by HPLC after 5 h reaction (Supplementary Figure 132). The analysis of these fragments by mass spectrometry permitted the assignment of Cys^{149}-Cys^{189} disulfide bond in both **cK1-1f** and **cK1-2f** proteins (Supplementary Figure 133).

Asp-N digestion. Each trypsic digest was diluted with 0.1% TFA in water and the resulting mixture was purified by HPLC in order to isolate the fragment containing in its structure cysteines 128, 177, 201 and 206. The HPLC fractions in which this fragment was detected were concentrated and dried under vacuum and the obtained residue was then dissolved in a solution of endoproteinase *N*-Asp (3 μL, 0.01 mg mL^{-1}) in 20 mM NH_4HCO_3. The digestion was carried out at 37 °C and was run for 2 min. The analysis of the new fragments by mass spectrometry permitted the assignment of Cys^{126}-Cys^{206} and Cys^{177}-Cys^{201} disulfide bonds for both **cK1-1f** and **cK1-2f** proteins (Supplementary Figure 134).

Supplementary Figures

General figures

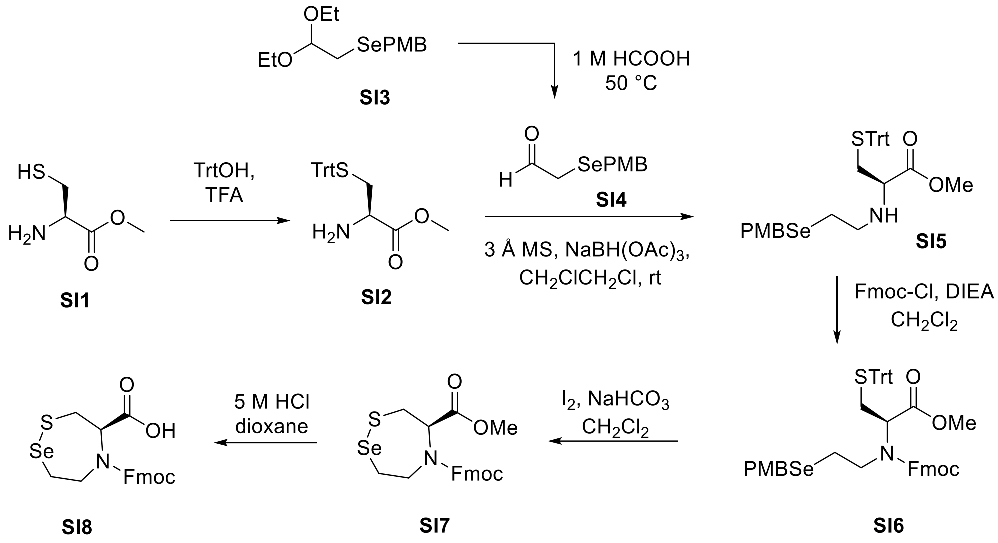

Supplementary Figure 1. Synthesis of the Fmoc-protected SetCys amino acid **SI8**.

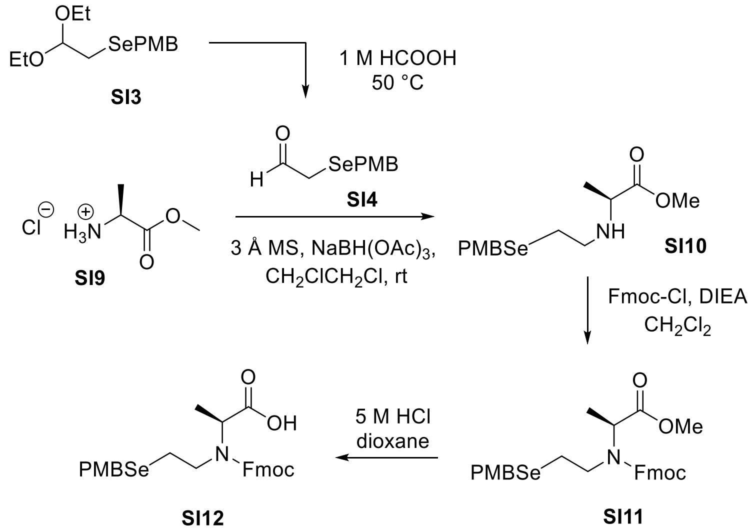

Supplementary Figure 2. Synthesis of the PMB-protected Fmoc-SetAla-OH amino acid **SI12**.

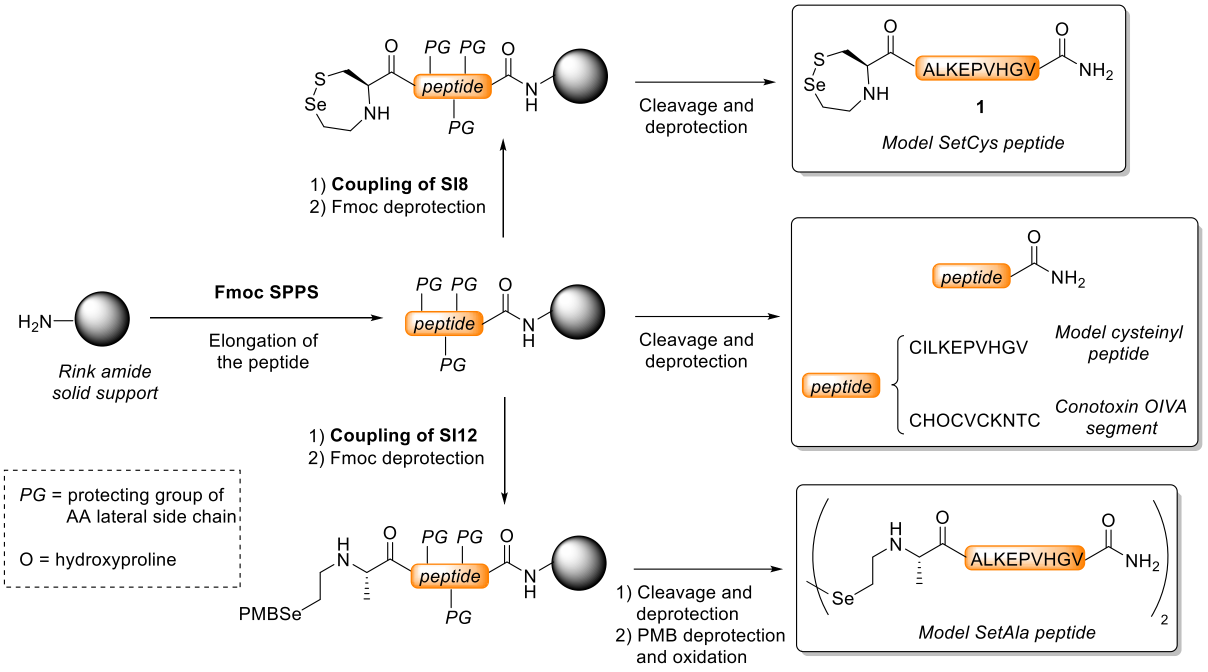

Supplementary Figure 3. General approach for the preparation of peptide amides.

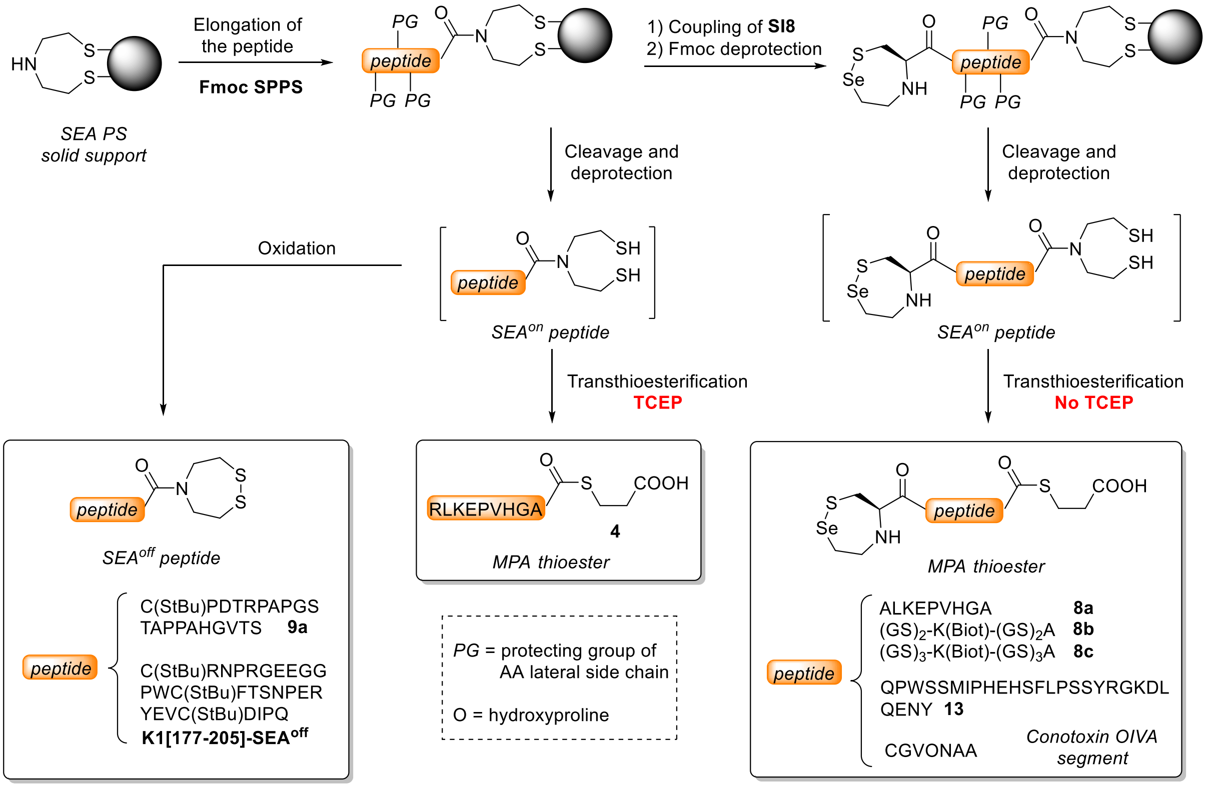

Supplementary Figure 4. General approach for the preparation of SEA^{off} peptides and MPA thioesters.

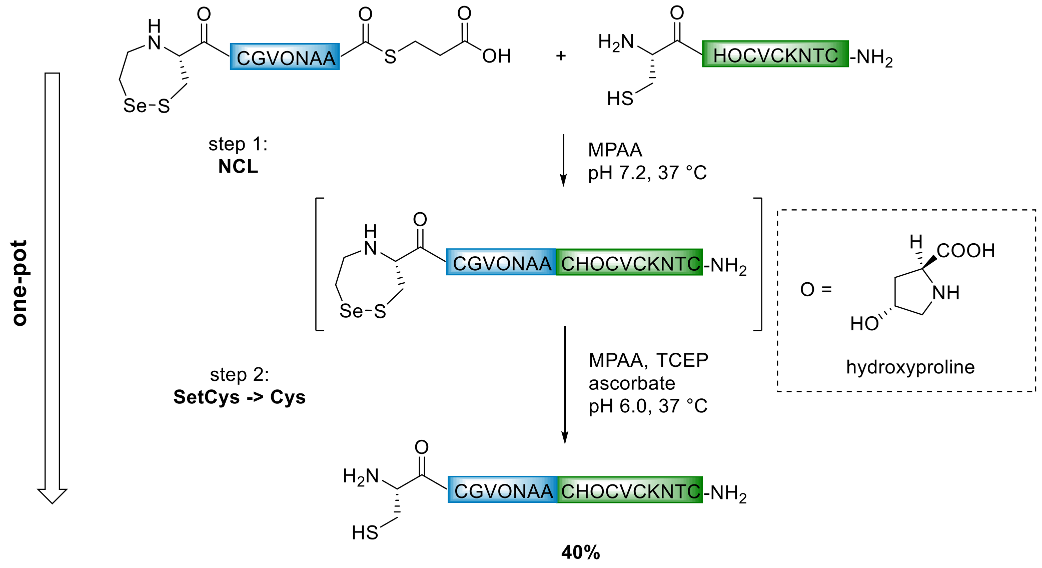

Supplementary Figure 5. Synthesis of conotoxin OIVA by ligation of two peptide segments.

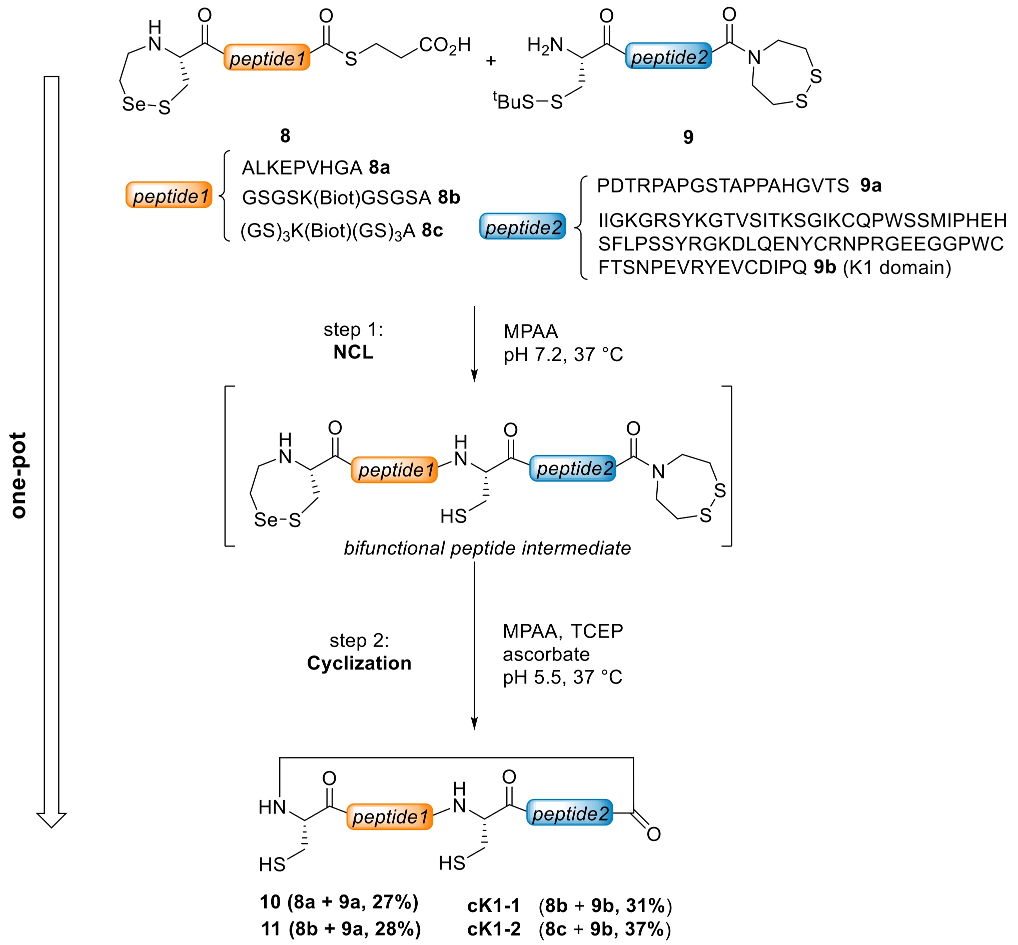

Supplementary Figure 6. One-pot synthesis of cyclic peptides **10, 11, cK1-1** and **cK1-2**.

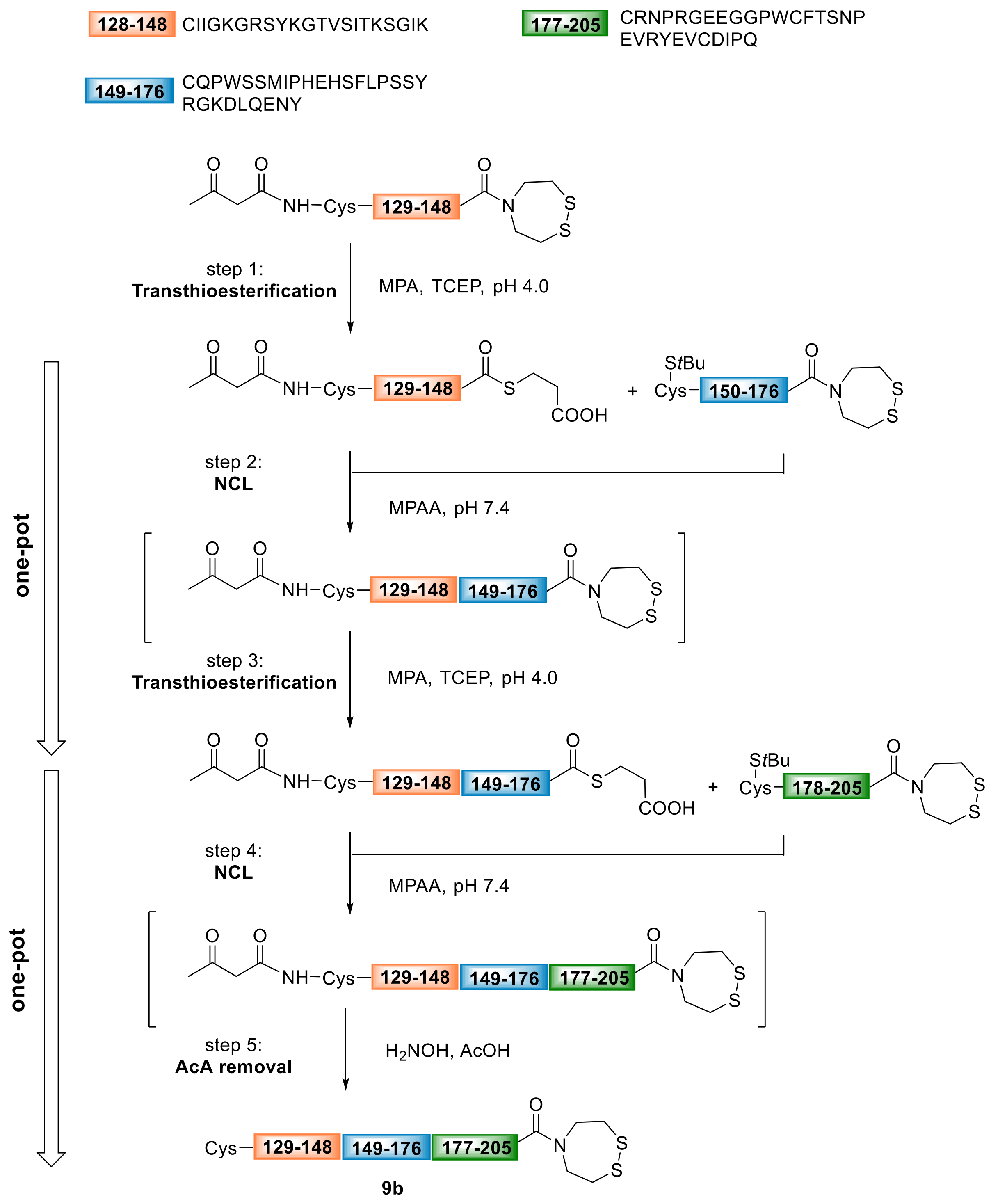

Supplementary Figure 7. Assembly of linear K1 precursor **9b**.

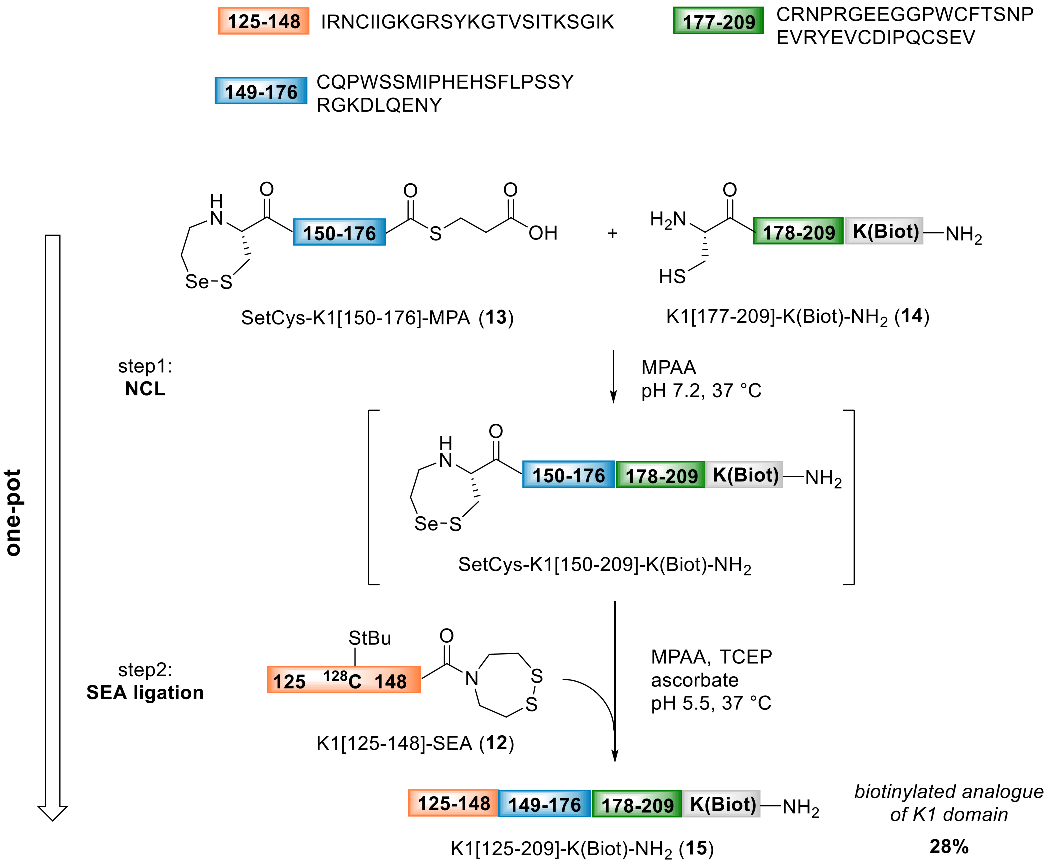

Supplementary Figure 8. One-pot three peptide segment assembly of linear K1 polypeptide **15** (Method A).

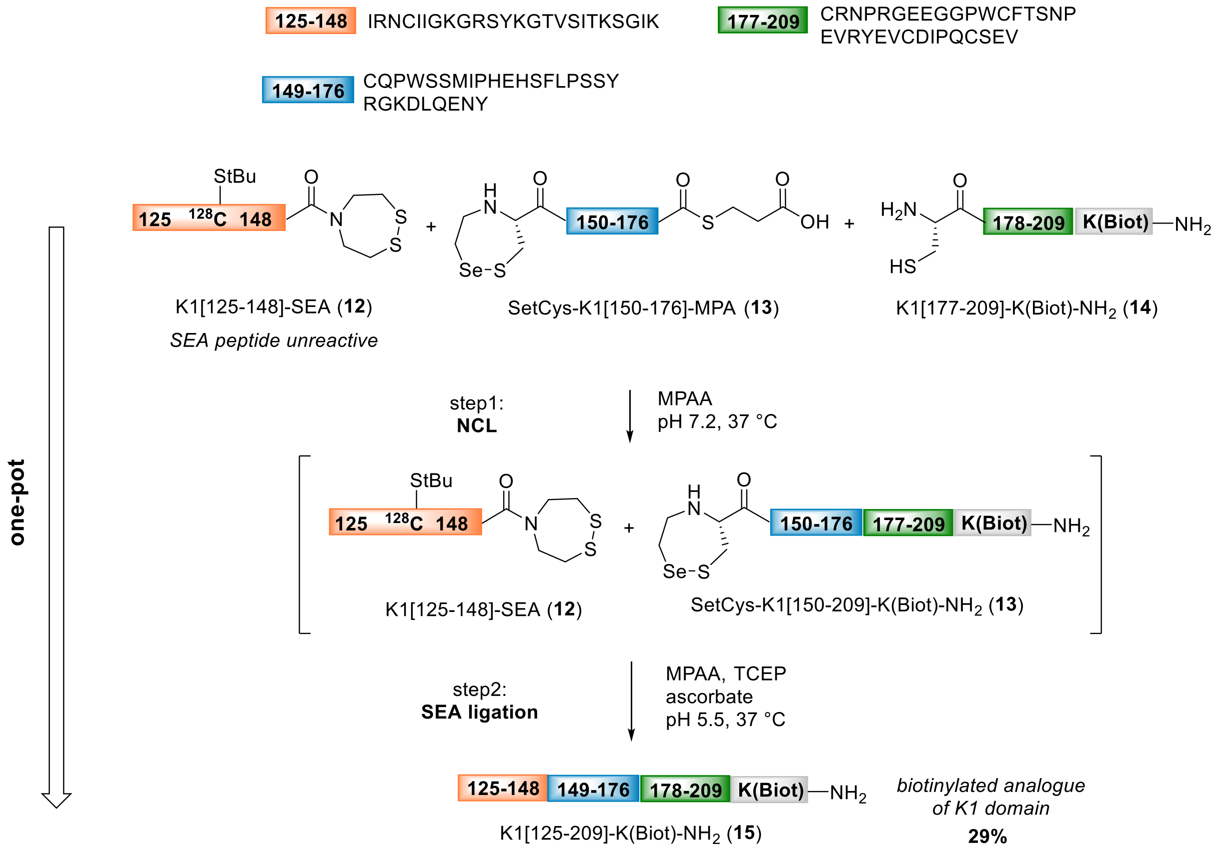

Supplementary Figure 9. One-pot three peptide segment assembly of linear K1 polypeptide **15** (Method B).

Synthesis of amino acids

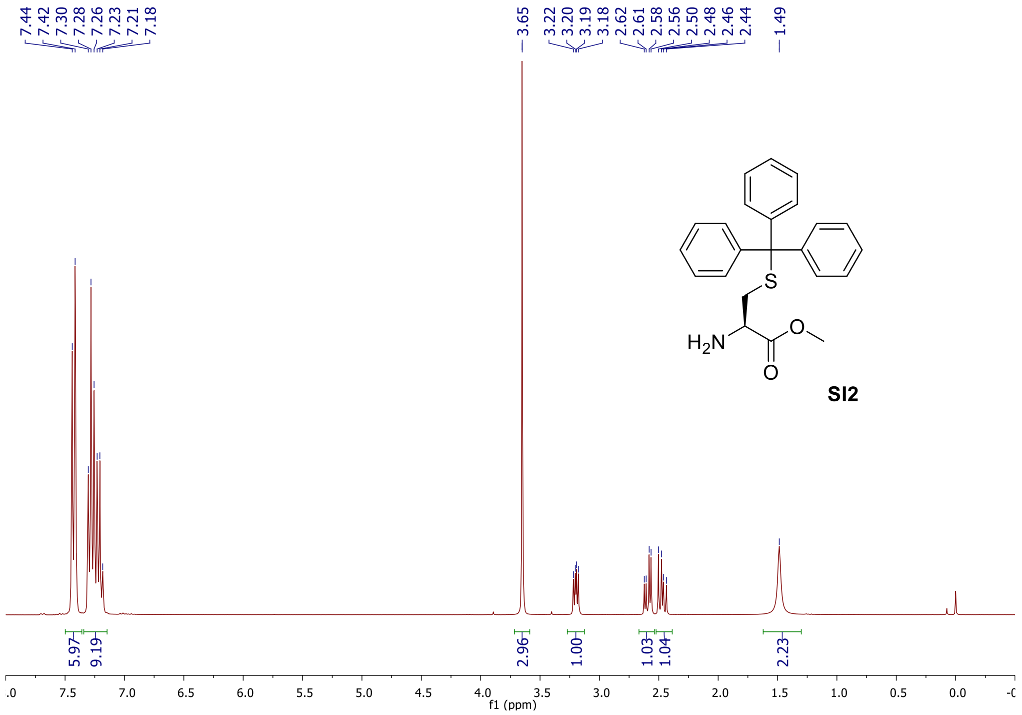

Supplementary Figure 10. ^{1}H NMR (300 MHz) spectrum of compound **SI2** ($CDCl_3$, 293 K).

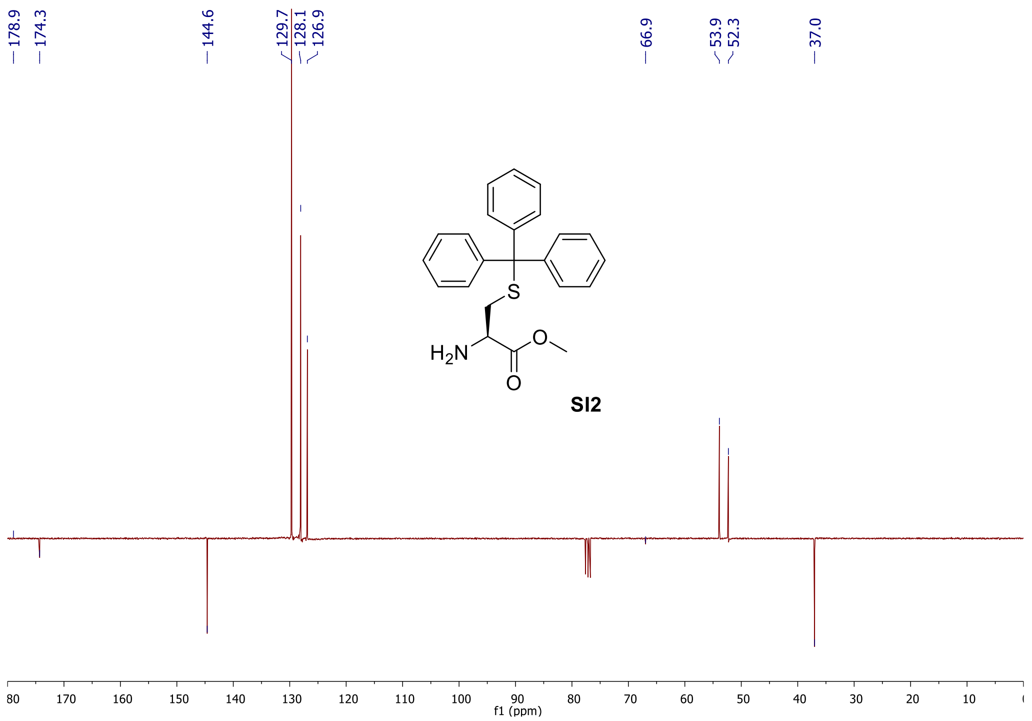

Supplementary Figure 11. ^{13}C JMOD NMR (75 MHz) spectrum of compound **SI2** ($CDCl_3$, 293 K).

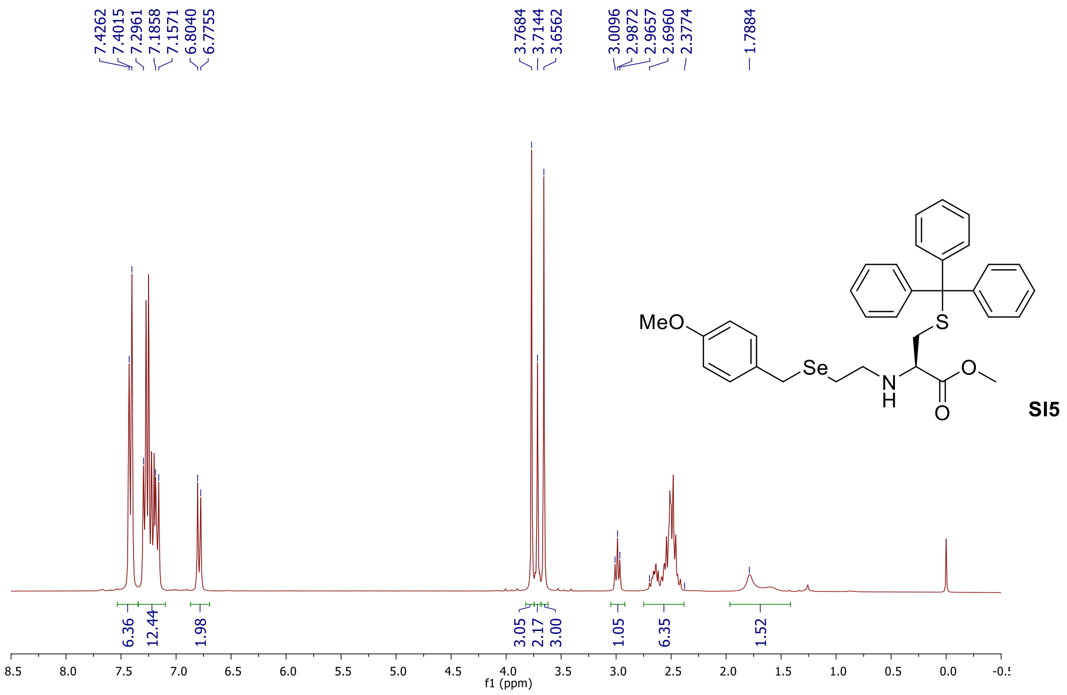

Supplementary Figure 12. ^{1}H NMR (300 MHz) spectrum of compound **SI5** ($CDCl_3$, 293 K).

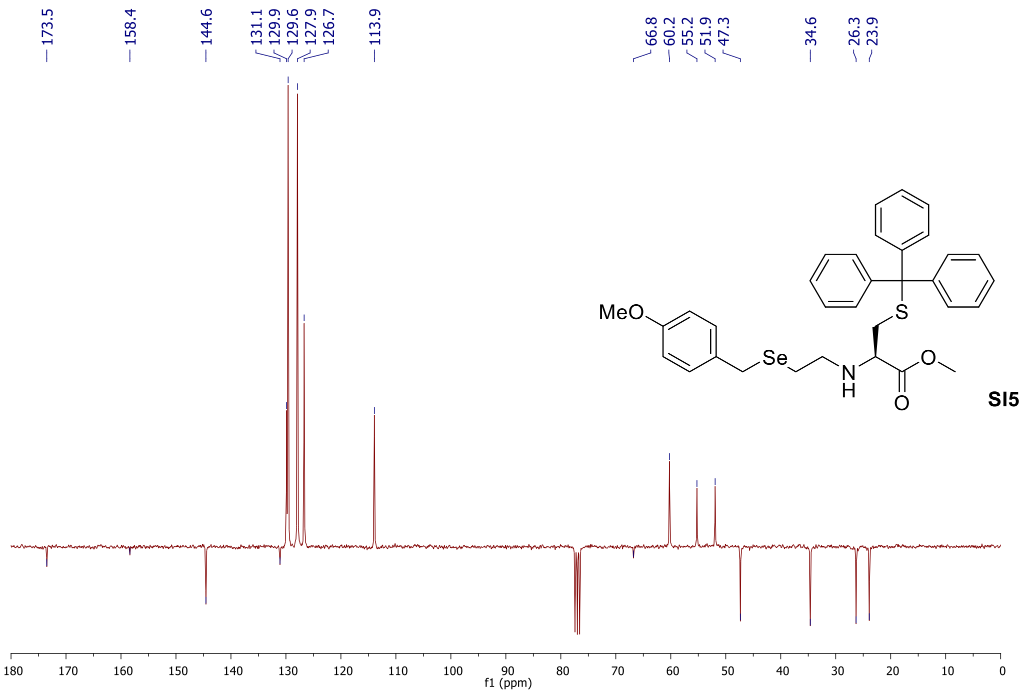

Supplementary Figure 13. ^{13}C JMOD NMR (75 MHz) spectrum of compound **SI5** ($CDCl_3$, 293 K).

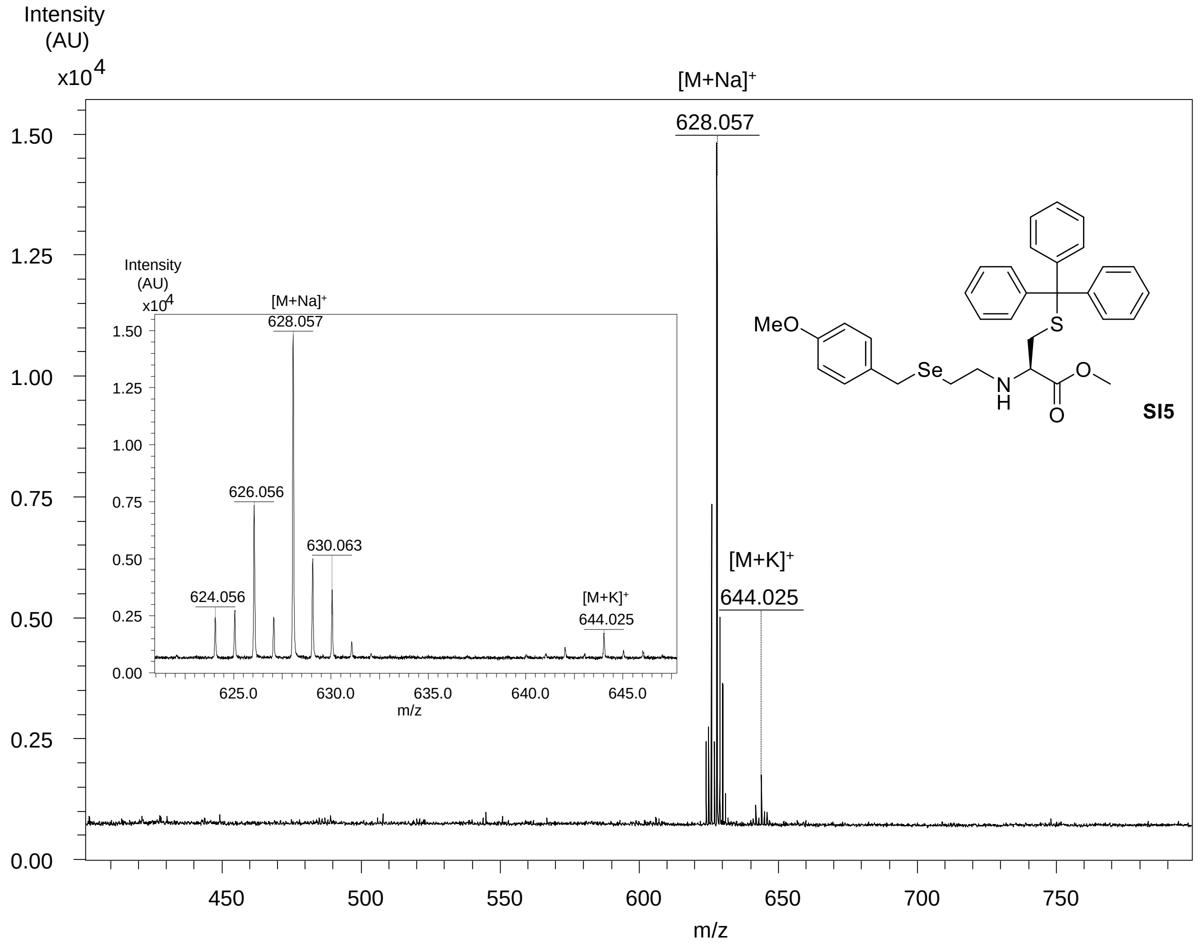

Supplementary Figure 14. MALDI-TOF analysis of compound **SI5**. Matrix 2,5-dihydroxybenzoic acid, positive detection mode, $[M+Na]^+$ calcd. (monoisotopic) 628.14, found 628.06.

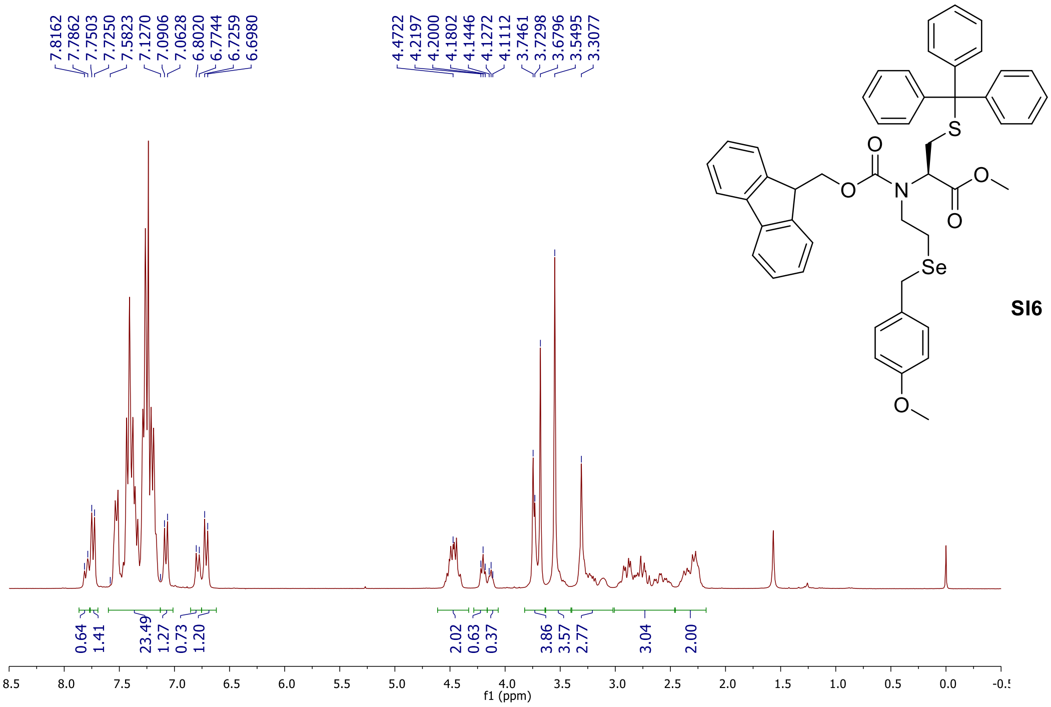

Supplementary Figure 15. ^{1}H NMR (300 MHz) spectrum of compound **SI6** ($CDCl_3$, 293 K).

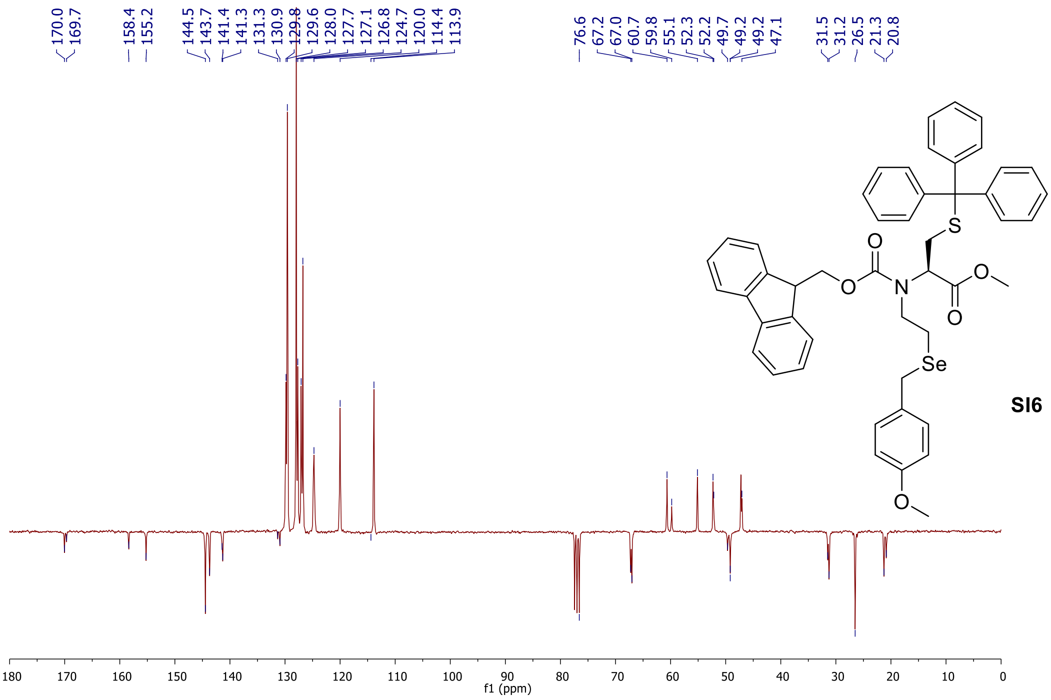

Supplementary Figure 16. ^{13}C JMOD NMR (75 MHz) spectrum of compound **SI6** ($CDCl_3$, 293 K).

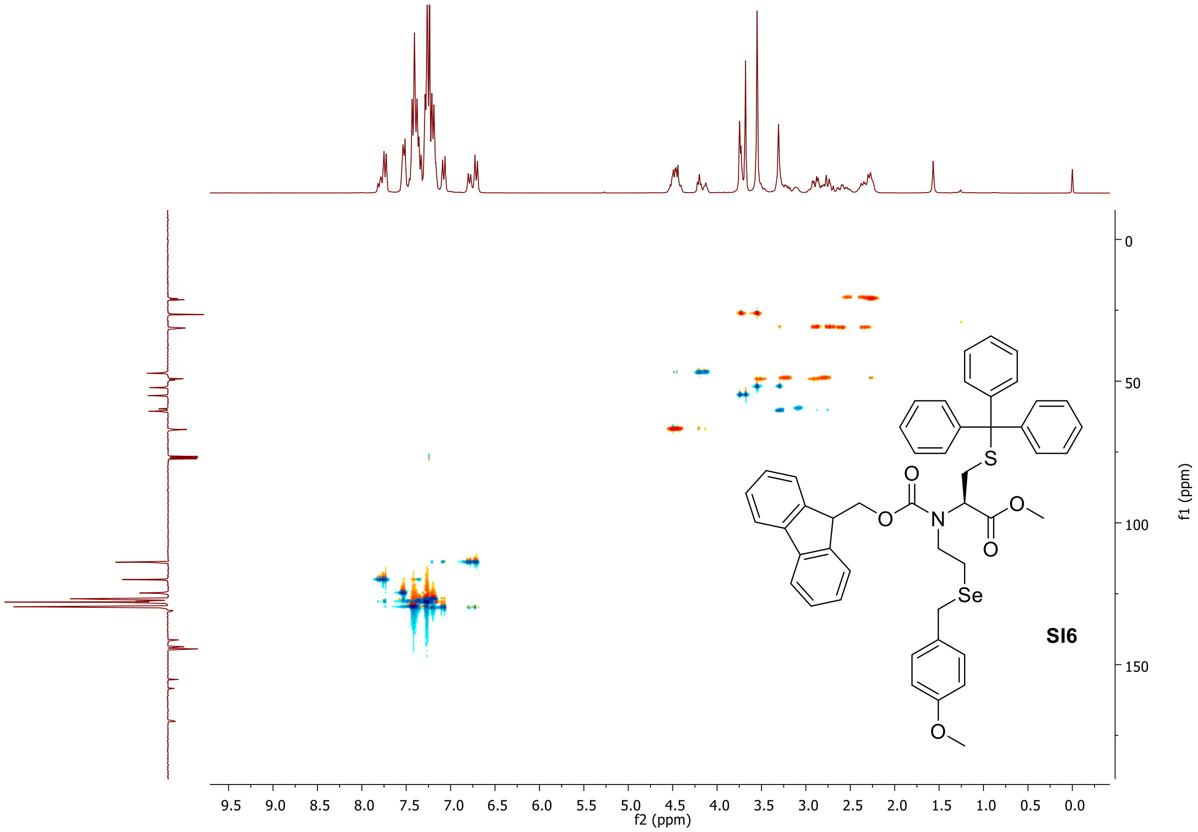

Supplementary Figure 17. ^{1}H-^{13}C HSQC spectrum of compound **SI6** ($CDCl_3$, 293 K).

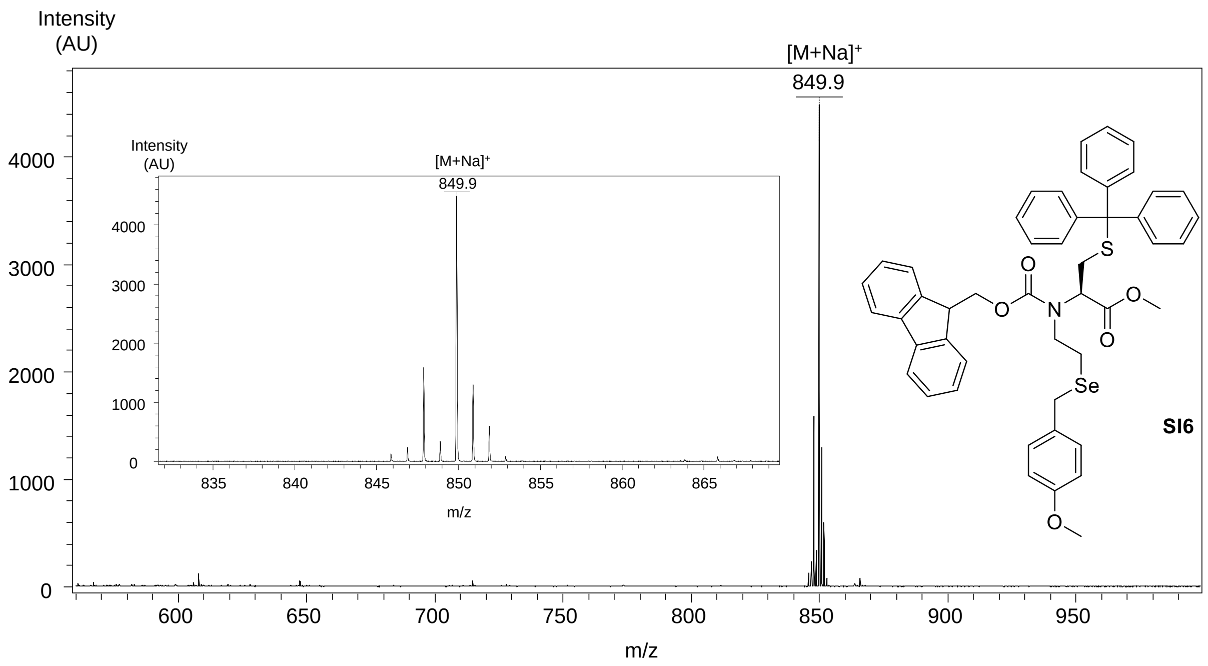

Supplementary Figure 18. MALDI-TOF analysis of compound **SI6**. Matrix 2,5-dihydroxybenzoic acid (DHB), positive detection mode, $[M+Na]^{+}$ calcd. (monoisotopic) 850.2, found 849.9.

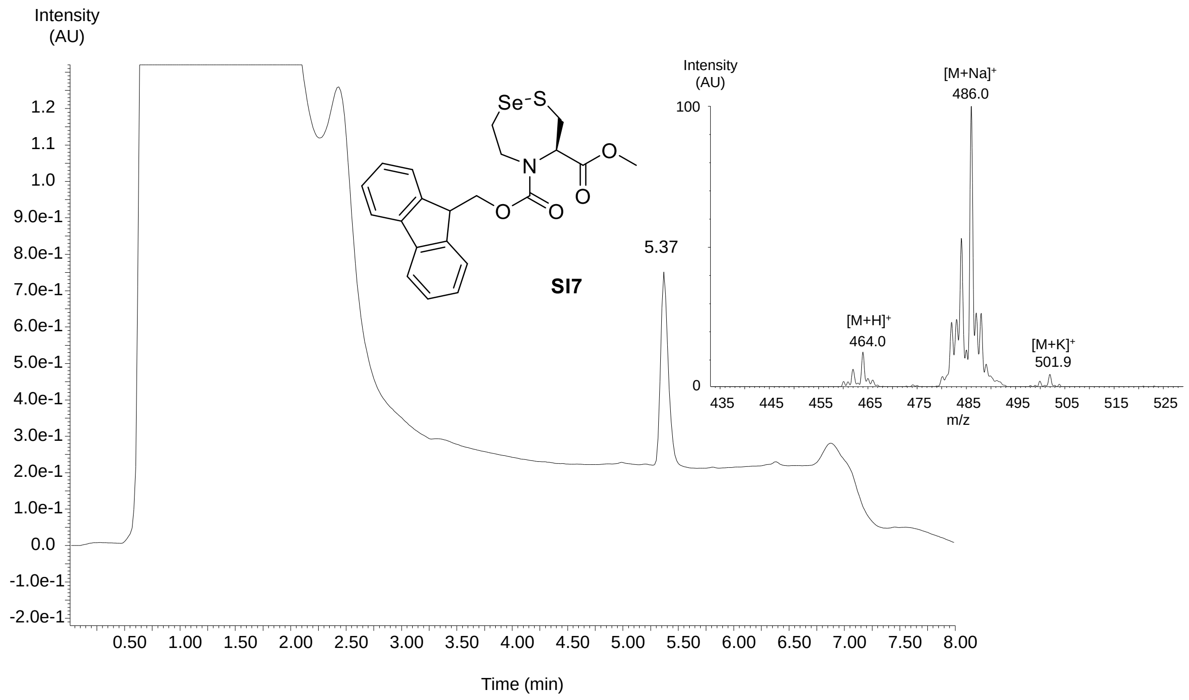

Supplementary Figure 19. LC-MS analysis of compound **SI7**. LC trace: eluent C 0.1% formic acid in water, eluent D 0.1% formic acid in CH_3CN/water: 4/1 v/v, C18 column, gradient 0-100% D in 4 min, 50 °C, 0.5 mL min^{-1}, UV detection. MS trace: $[M+H]^+$ m/z calcd. (monoisotopic mass) 464.0, found 464.0; $[M+Na]^+$ m/z calcd. (monoisotopic mass) 486.0, found 486.0.

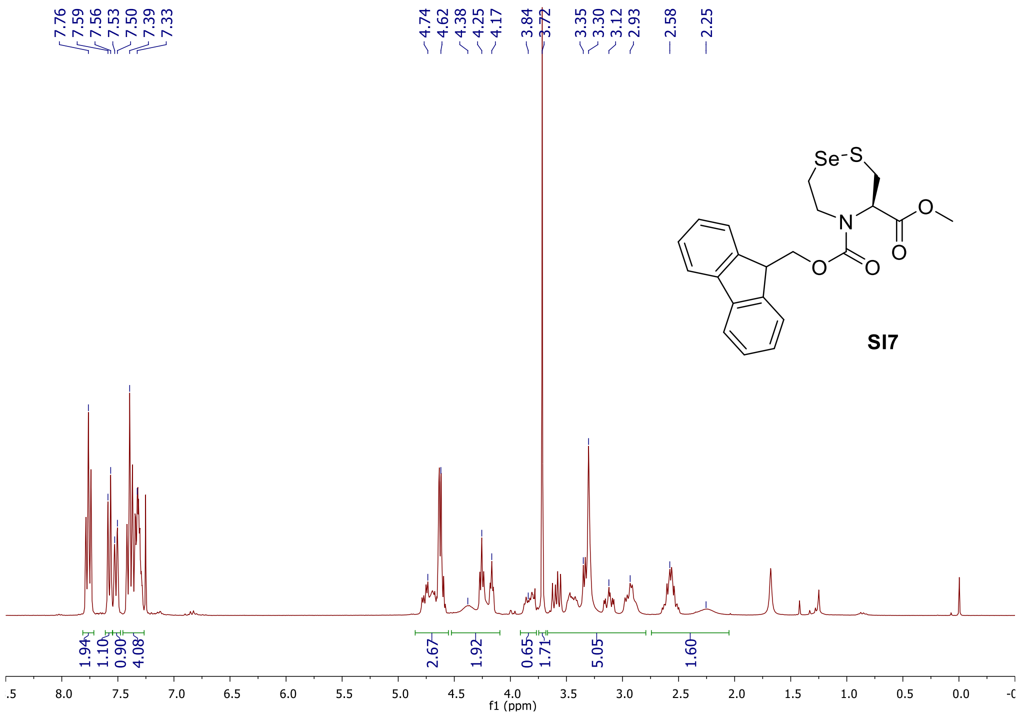

Supplementary Figure 20. ^1H NMR (300 MHz) spectrum of compound **SI7** ($CDCl_3$, 293 K).

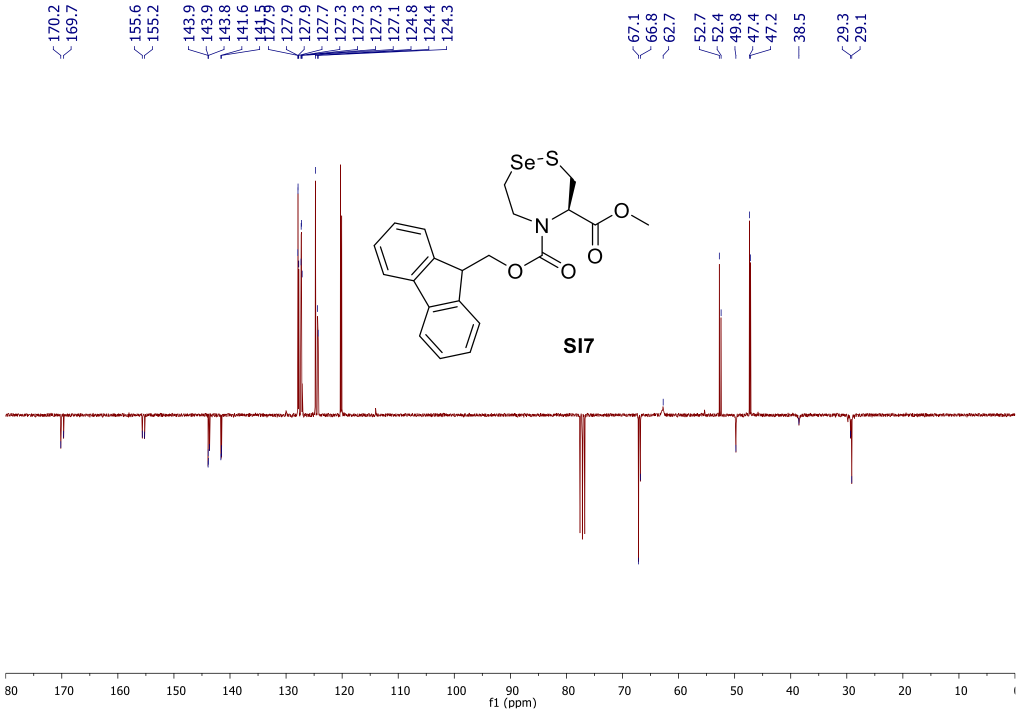

Supplementary Figure 21.^{13}C JMOD NMR (75 MHz) spectrum of compound **SI7** ($CDCl_3$, 293 K).

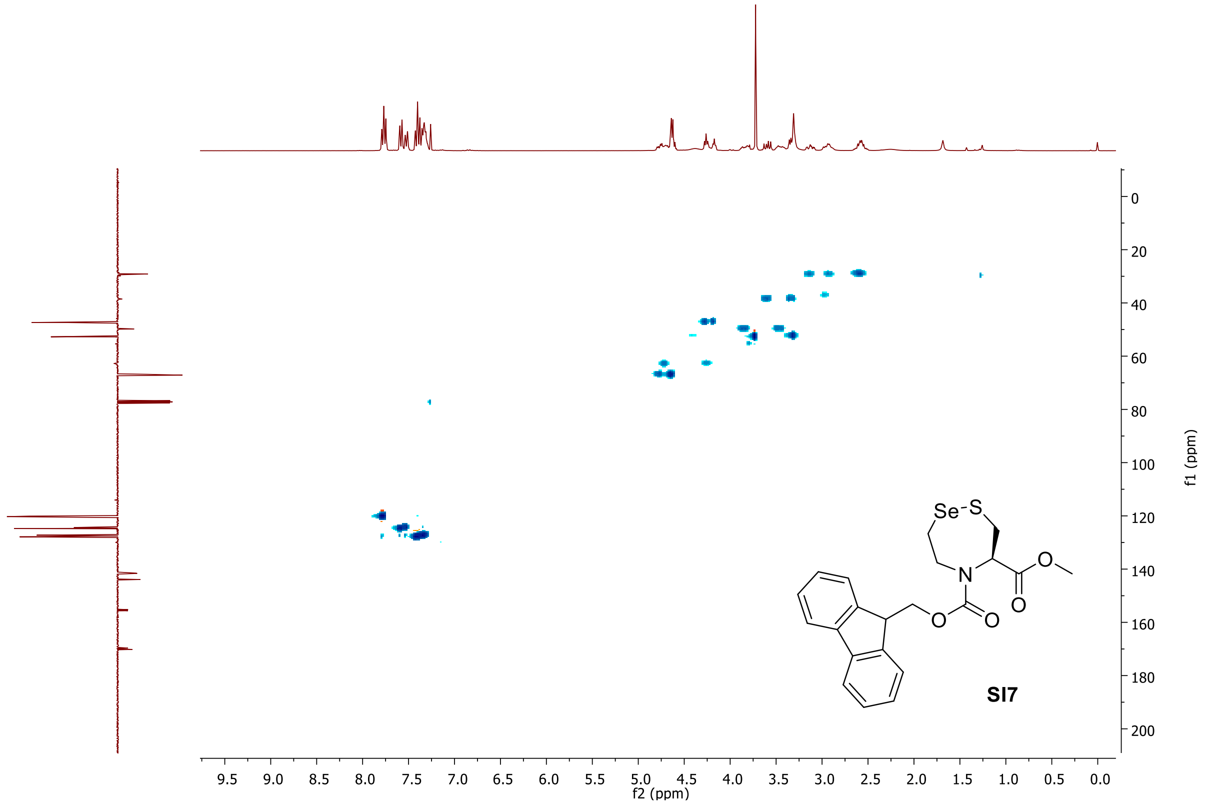

Supplementary Figure 22. ^{1}H-^{13}C HSQC spectrum of compound **SI7** ($CDCl_3$, 293 K).

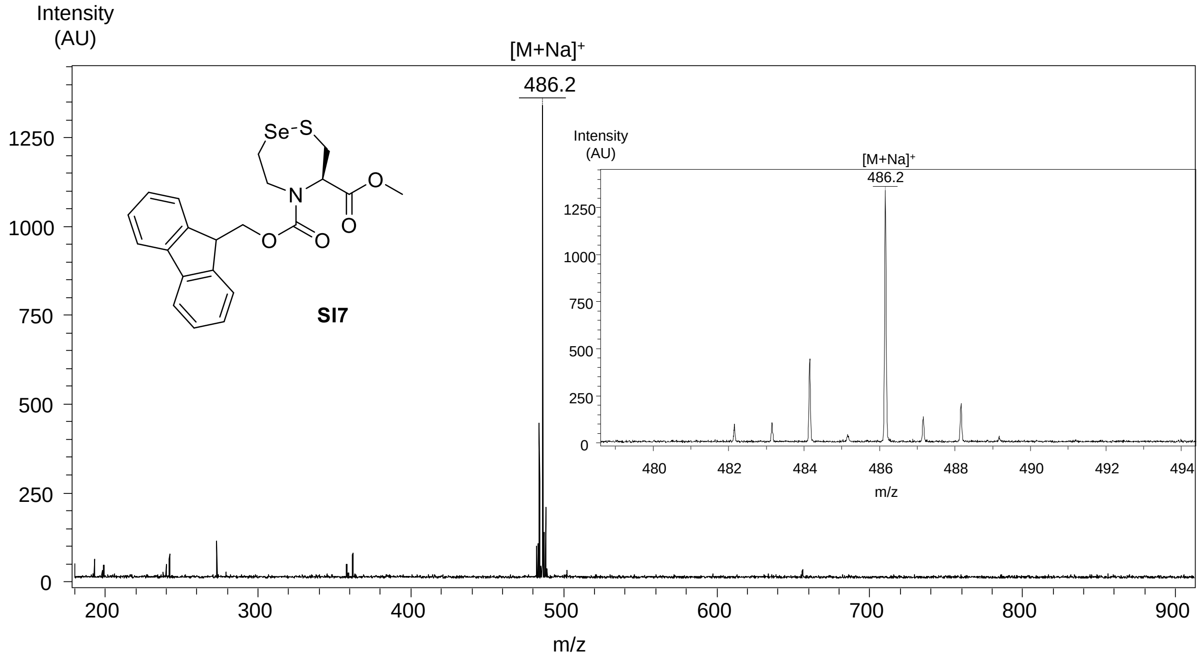

Supplementary Figure 23. MALDI-TOF analysis of compound **SI7**. Matrix 2,5-dihydroxybenzoic acid, positive detection mode, $[M+Na]^+$ calcd. (monoisotopic) 486.0, found 486.2.

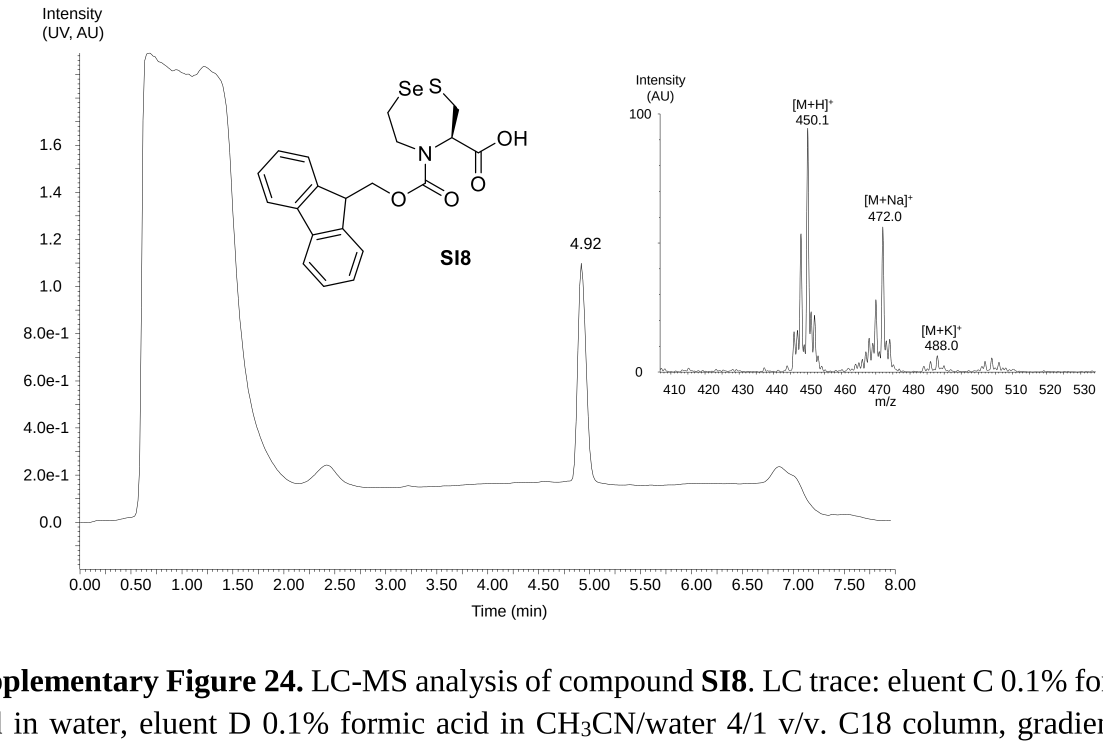

Supplementary Figure 24. LC-MS analysis of compound **SI8**. LC trace: eluent C 0.1% formic acid in water, eluent D 0.1% formic acid in CH_3CN/water 4/1 v/v. C18 column, gradient 0-100% D in 4 min, 50 °C, 0.5 mL min^{-1}, UV detection. MS trace: $[M+H]^+$ m/z calcd. (monoisotopic mass) 450.0, found 450.1; $[M+Na]^+$ m/z calcd. (monoisotopic mass) 472.0, found 472.0.

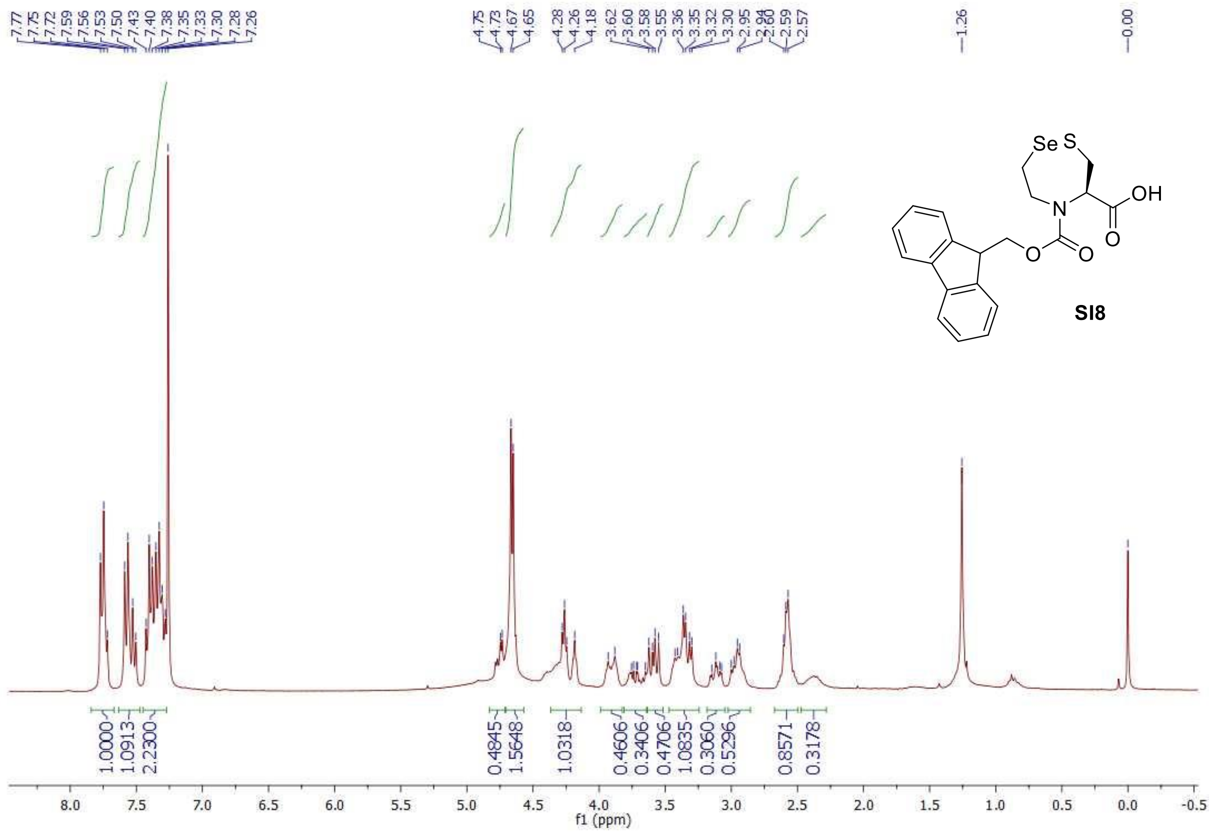

Supplementary Figure 25. ^{1}H NMR (300 MHz) spectrum of compound **SI8** ($CDCl_3$, 293 K).

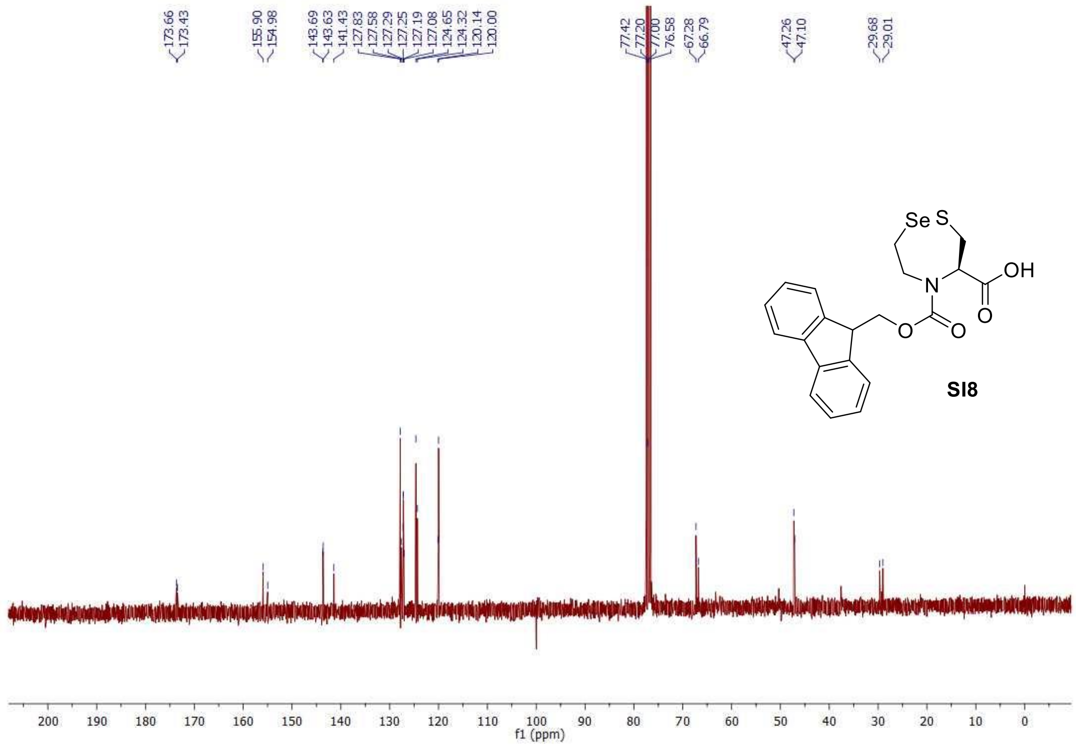

Supplementary Figure 26. ^{13}C NMR (75 MHz) spectrum of compound **SI8** ($CDCl_3$, 293 K).

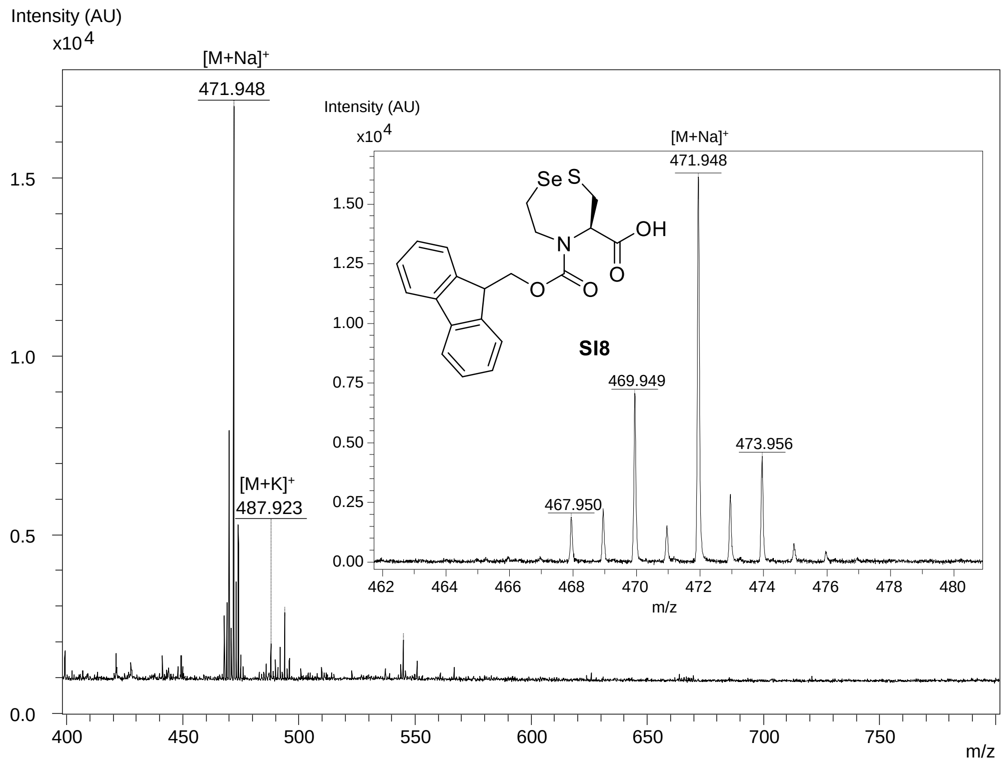

Supplementary Figure 27. MALDI-TOF analysis of compound **SI8**. Matrix 2,5-dihydroxybenzoic acid, positive detection mode, $[M+Na]^+$ calcd. (monoisotopic) 472.01, found 471.95.

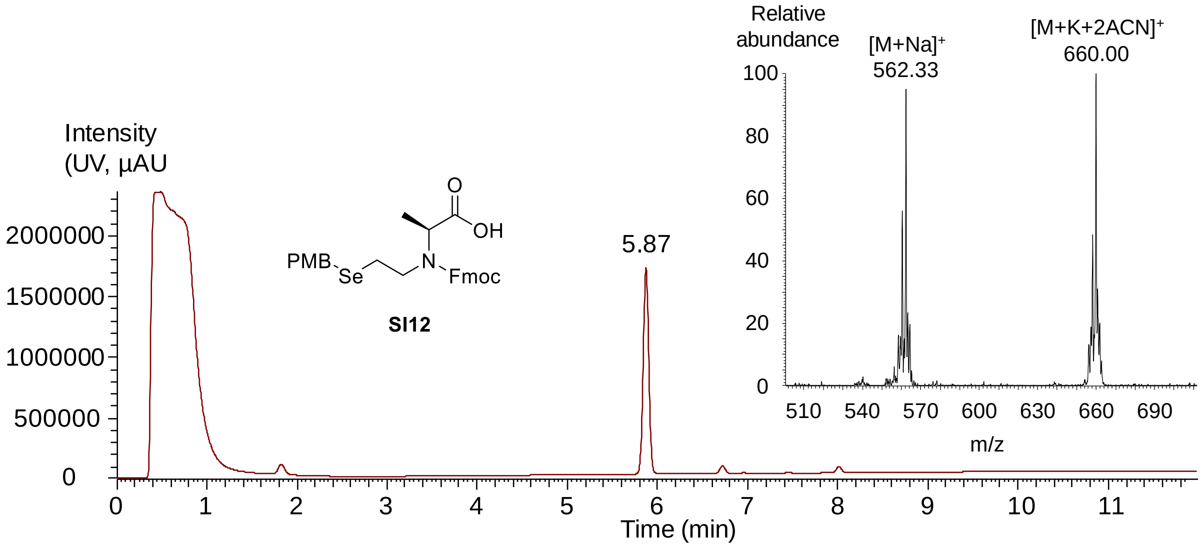

Supplementary Figure 28. LC-MS analysis of PMB-protected Fmoc-SetAla-OH **SI12**. LC trace: eluent C 0.1% formic acid in water, eluent D 0.1% formic acid in CH_3CN, gradient 40-100% D in 10 min, 0.4 mL min^{-1}, Atlantis T3 3 µm (2.1 × 50 mm) column, 50 °C, UV detection. MS trace: $[M+Na]^+$ m/z calcd. (monoisotopic mass) 562.11, found 562.33.

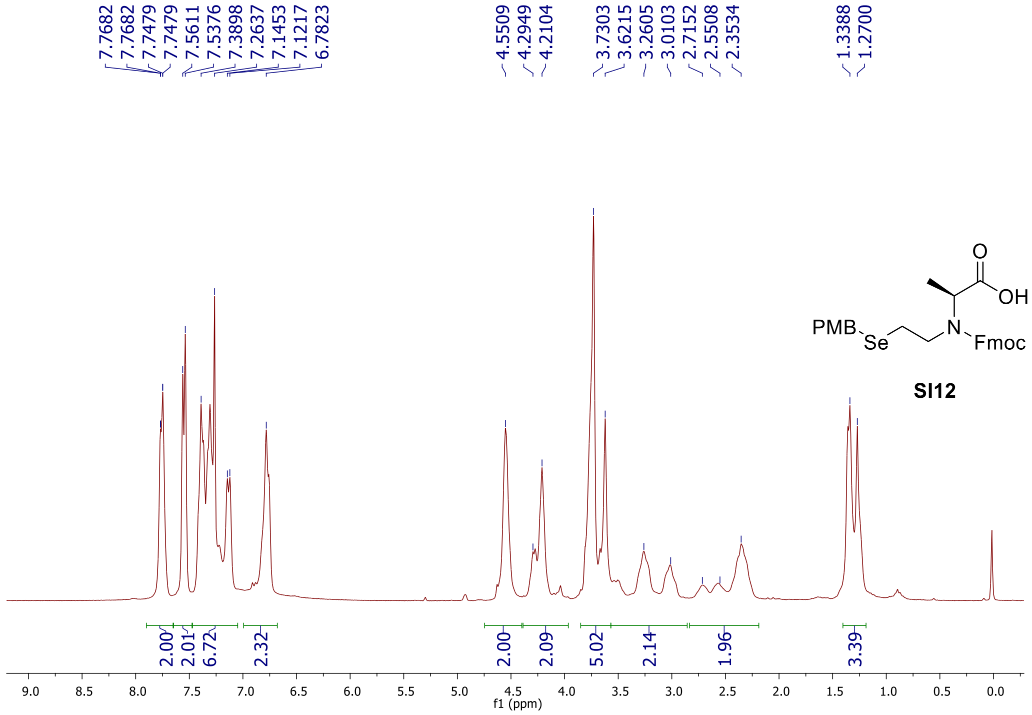

Supplementary Figure 29. ^1H NMR (300 MHz) spectrum of PMB-protected Fmoc-SetAla-OH **SI12** ($CDCl_3$, 293 K).

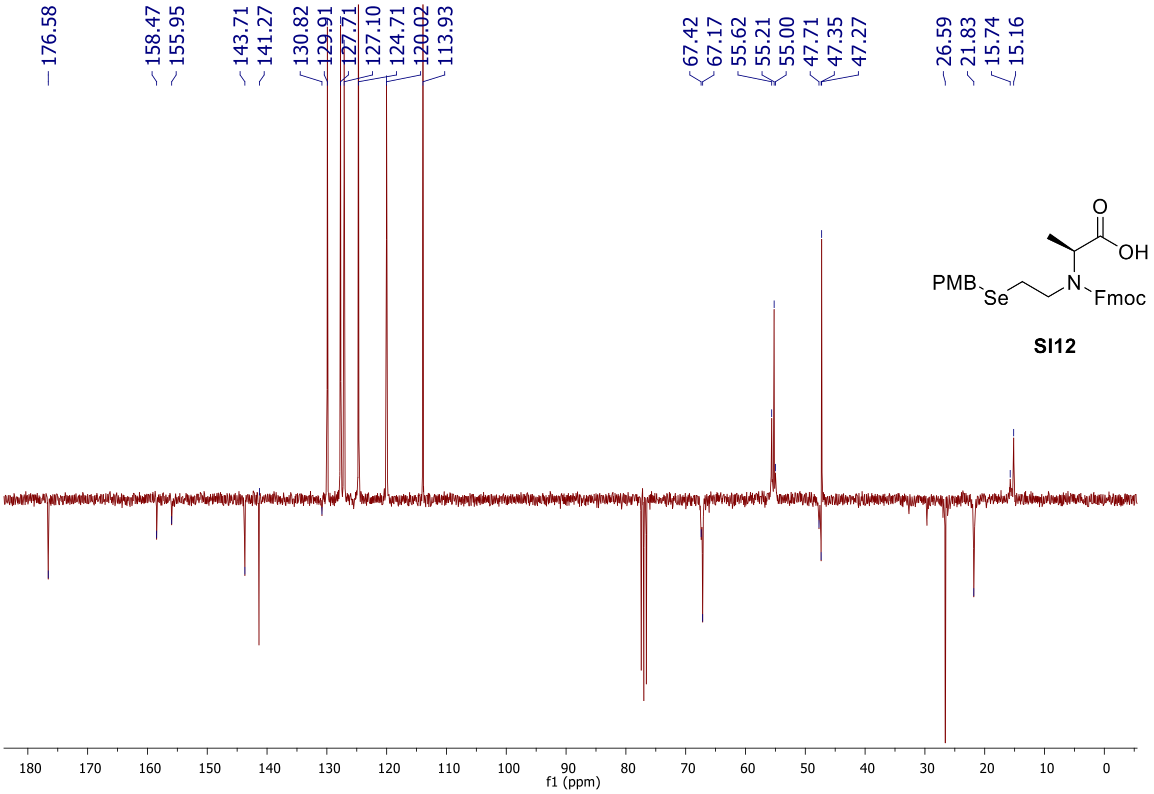

Supplementary Figure 30. ^{13}C NMR (75 MHz) spectrum of PMB-protected Fmoc-SetAla-OH **SI12** ($CDCl_3$, 293 K).

Peptide amides

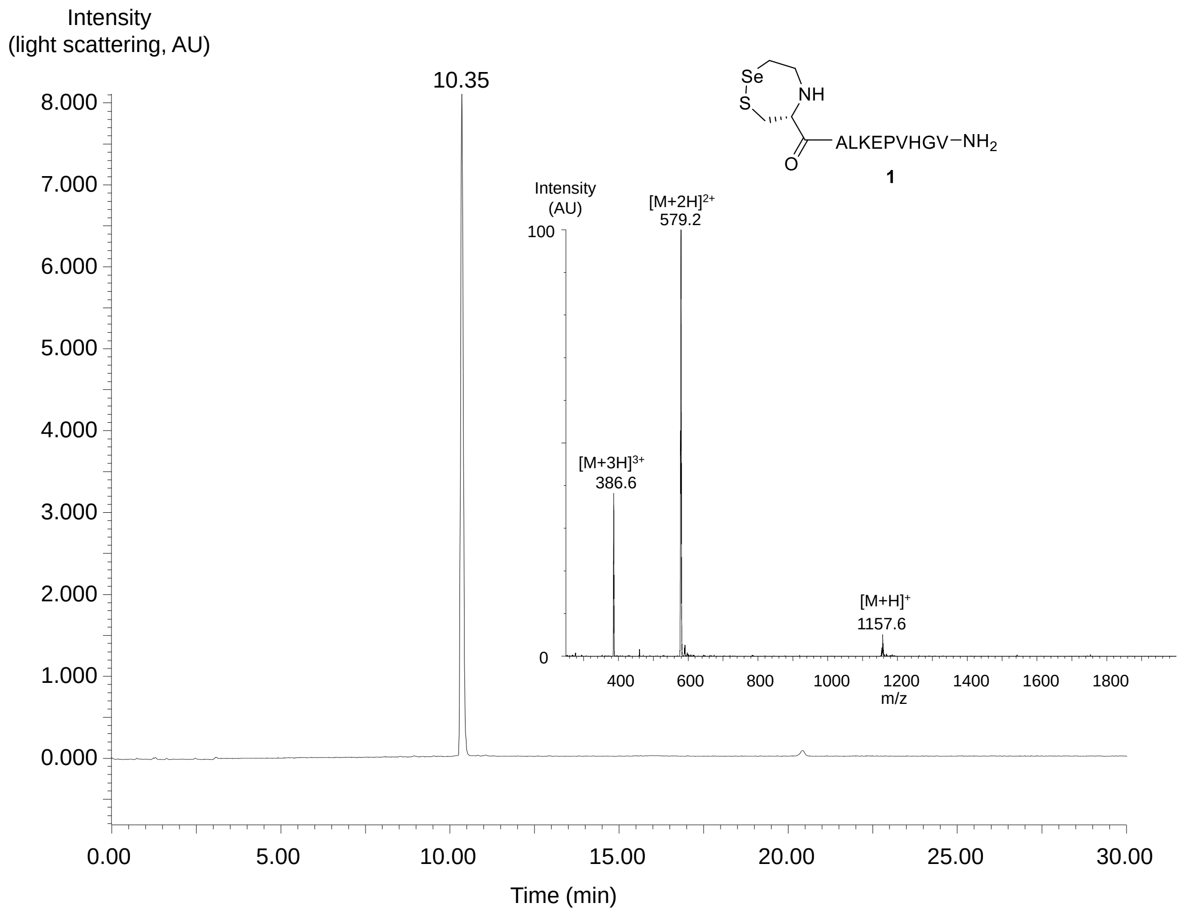

Supplementary Figure 31. LC-MS analysis of SetCys-ALKEPVHGV-NH_2 peptide **1**. LC trace: eluent A 0.10% TFA in water, eluent B 0.10% TFA in CH_3CN/water 4/1 v/v. C18 column, gradient 0-100% B in 30 min, 30 °C, 1 mL min^{-1}, light scattering detection. MS trace: m/z calcd. for $[M+H]^+$ (monoisotopic mass): 1157.5, found: 1157.6.

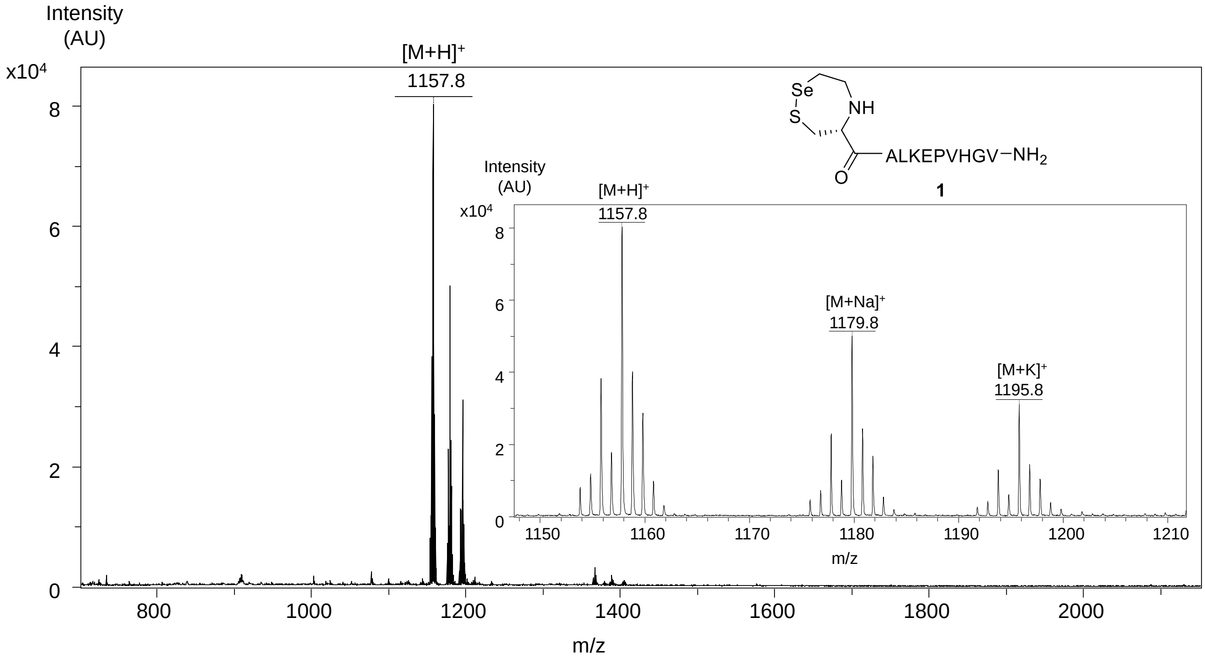

Supplementary Figure 32. MALDI-TOF analysis of SetCys-ALKEPVHGV-NH_2 peptide **1**. Matrix α-cyano-4-hydroxycinnamic acid, positive detection mode, calcd. for $[M+H]^+$ (monoisotopic): 1157.5, found: 1157.8.

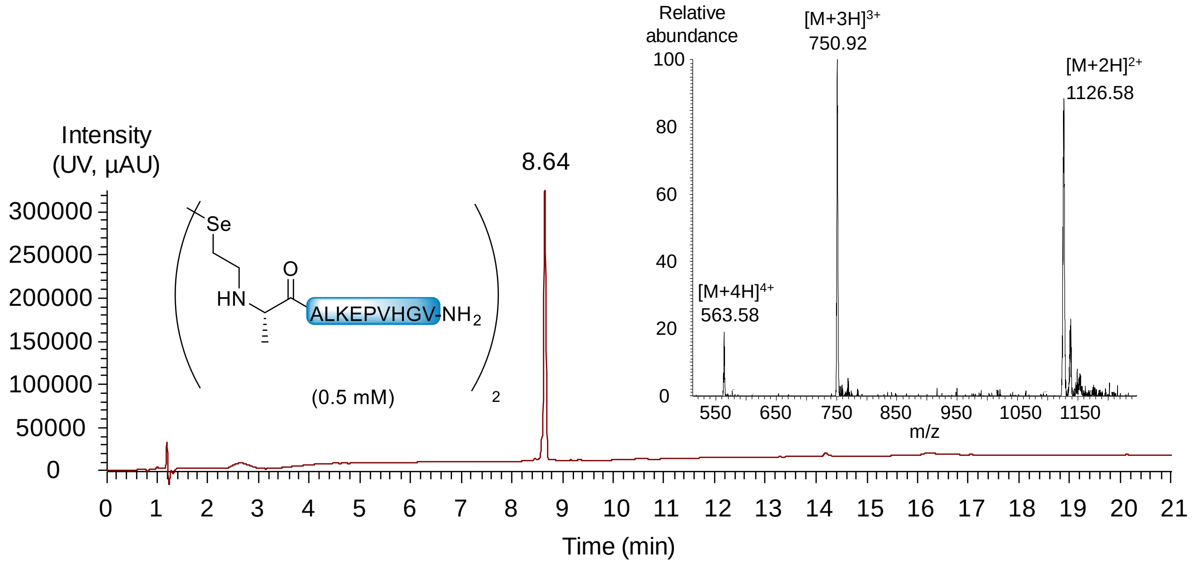

Supplementary Figure 33. LC-MS analysis of the diselenide (SetAla-ALKEPVHGV-NH_2)$_2$. LC trace: eluent A: 0.1% TFA in water, eluent B: 0.1% TFA in CH_3CN, gradient: 0-70% B in 20 min, 0.4 mL min^{-1}; column: AQUITY UPLC ® peptide BHE C18, 300 Å, 1.7 µm, 2.1 × 150 mm; 50 °C; UV detection. MS trace: m/z = 1126.6 ($[M+2H]^{2+}$), 750.9 ($[M+3H]^{3+}$), 563.58 ($[M+4H]^{4+}$); calcd. for M (average): 2250.4, found 2250.4.

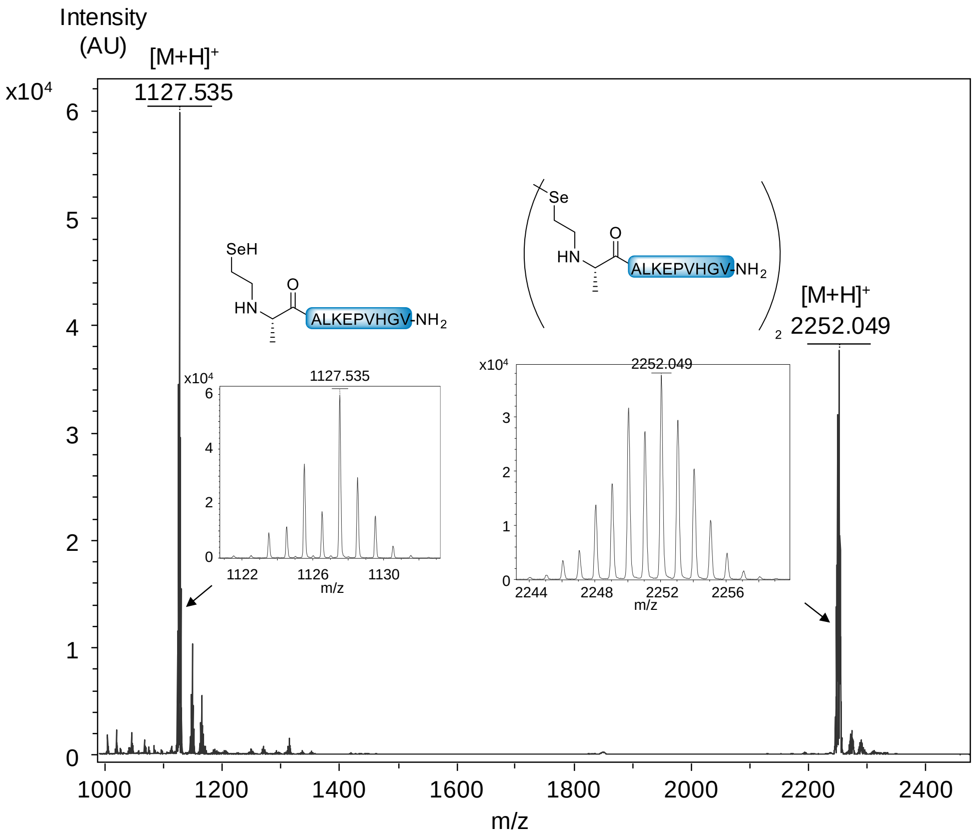

Supplementary Figure 34. MALDI-TOF analysis of the diselenide (SetAla-ALKEPVHGV-$NH_2)_2$. Matrix α-cyano-4-hydroxycinnamic acid, positive detection mode. The diselenide as well as the selenol are detected by MALDI-TOF analysis. Calcd. for $[M+H]^+$ (selenol): 1127.55, found: 1127.53; calcd for $[M+H]^+$ (diselenide): 2252.07, found: 2252.05.

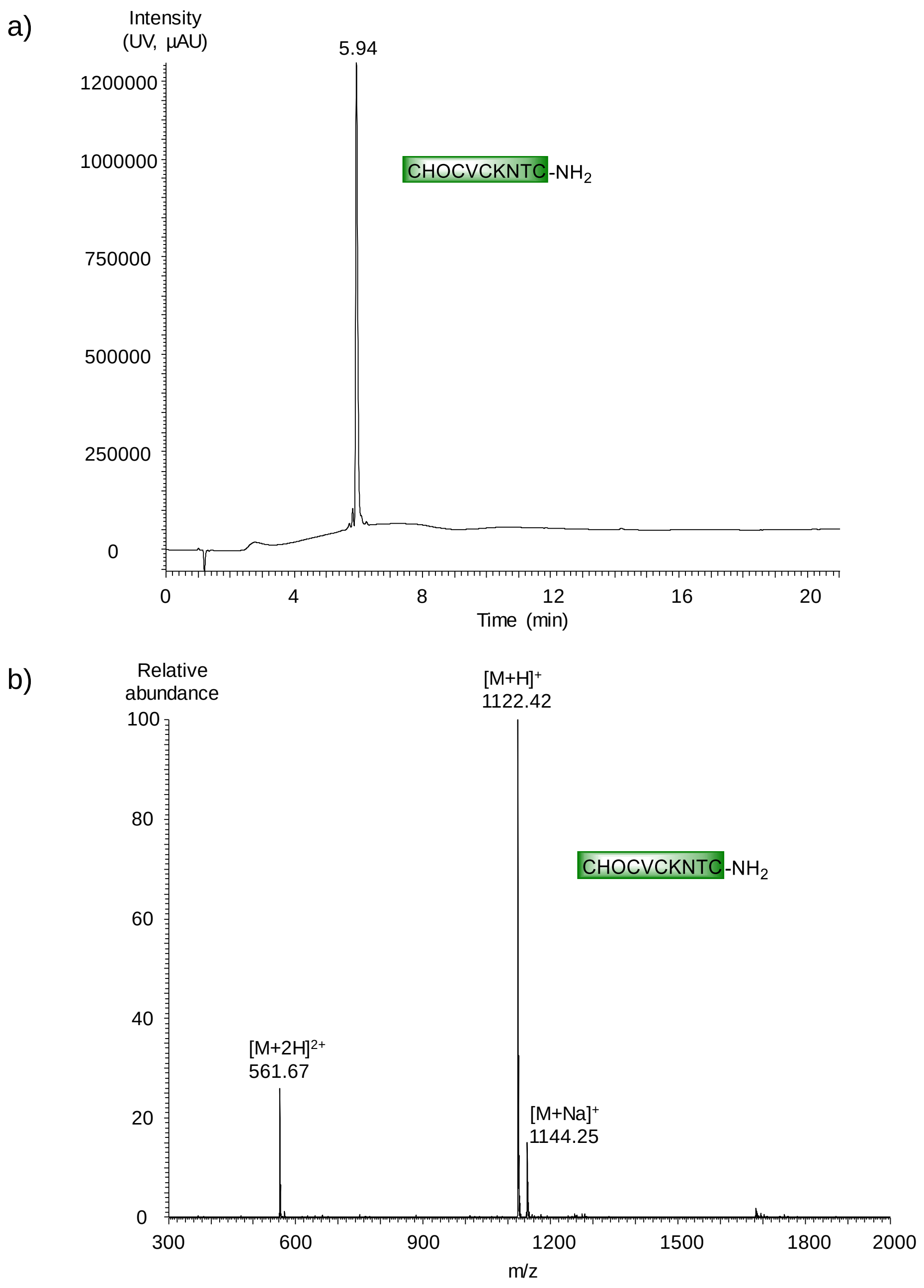

Supplementary Figure 35. UPLC-MS analysis of CHOCVCKNTC-NH_2. a) LC trace. Eluent A 0.1% TFA in water, eluent B 0.1% TFA in CH_3CN, ACQUITY UPLC Peptide BEH 300 Å 1.7 µm (2.1 × 150 mm) column, 50 °C, gradient 0-70% B in 20 min, 0.4 mL min^{-1}, UV detection. b) MS trace: calcd. for $[M+H]^+$ (monoisotopic): 1122.42, found: 1122.42.

SEA peptides

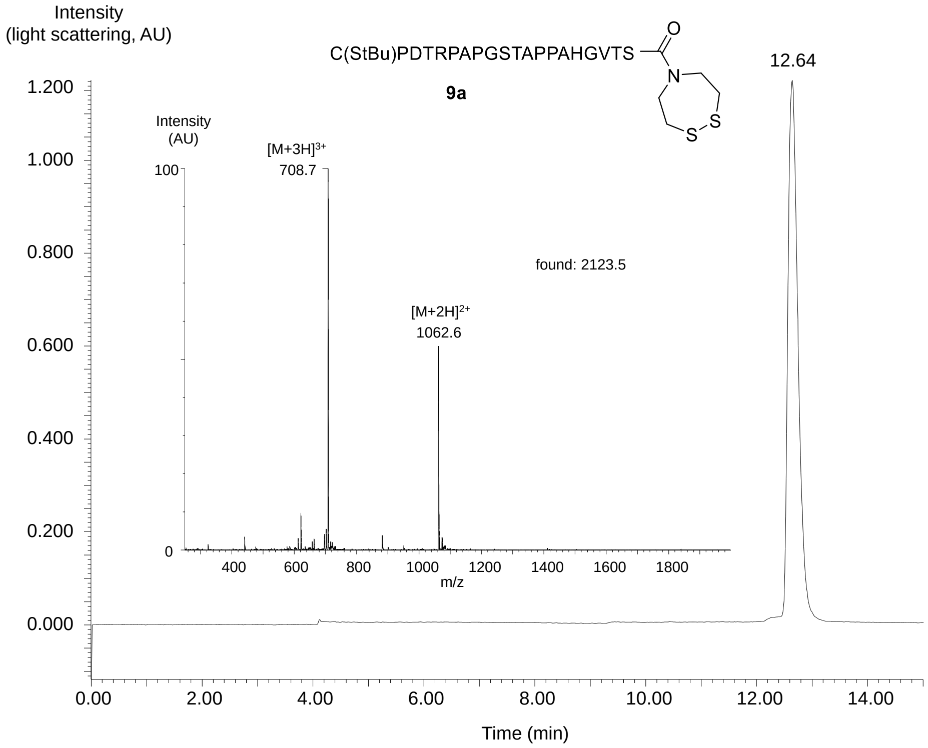

Supplementary Figure 36. LC-MS analysis of peptide **9a**. LC trace: eluent A 0.1% TFA in water, eluent B 0.1% TFA in CH_3CN/water 4/1 v/v, C18 column, gradient 0-50% B in 15 min, 30 °C, 1 mL min^{-1}, light scattering detection. MS trace: m/z = 1062.5 ($[M+2H]^{2+}$), 708.7 ($[M+3H]^{3+}$). Calcd. for M (average): 2124.5, found: 2123.5.

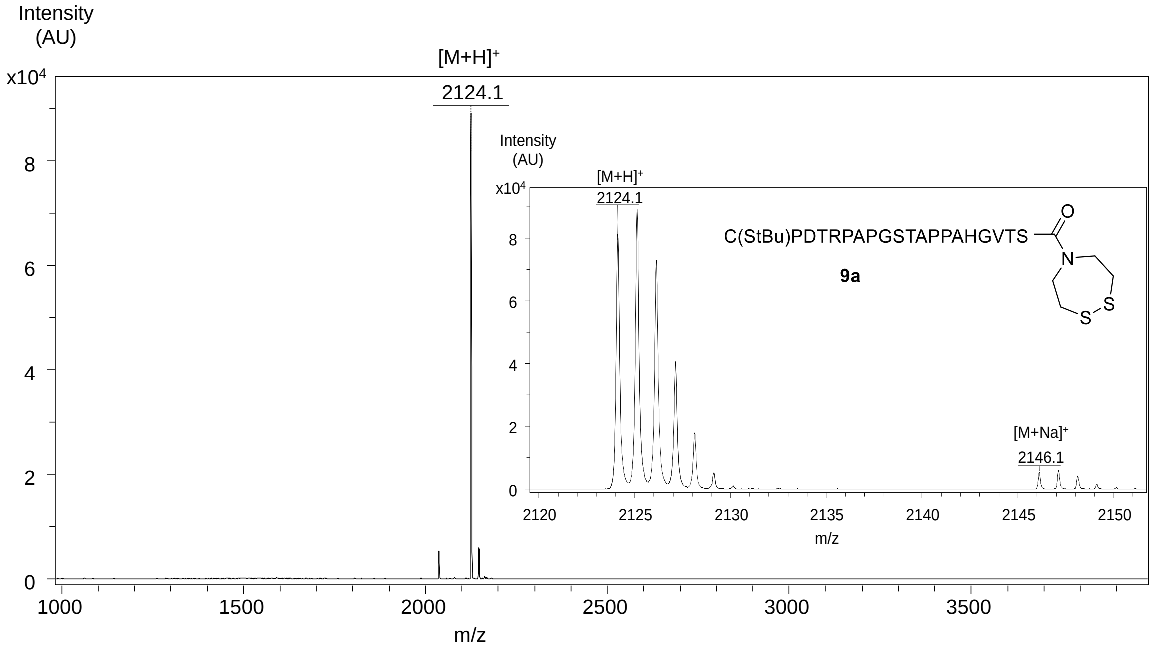

Supplementary Figure 37. MALDI-TOF analysis of peptide **9a**. Matrix α-cyano-4-hydroxycinnamic acid, positive detection mode, calcd. for $[M+H]^+$ (monoisotopic): 2123.9, found: 2124.1.

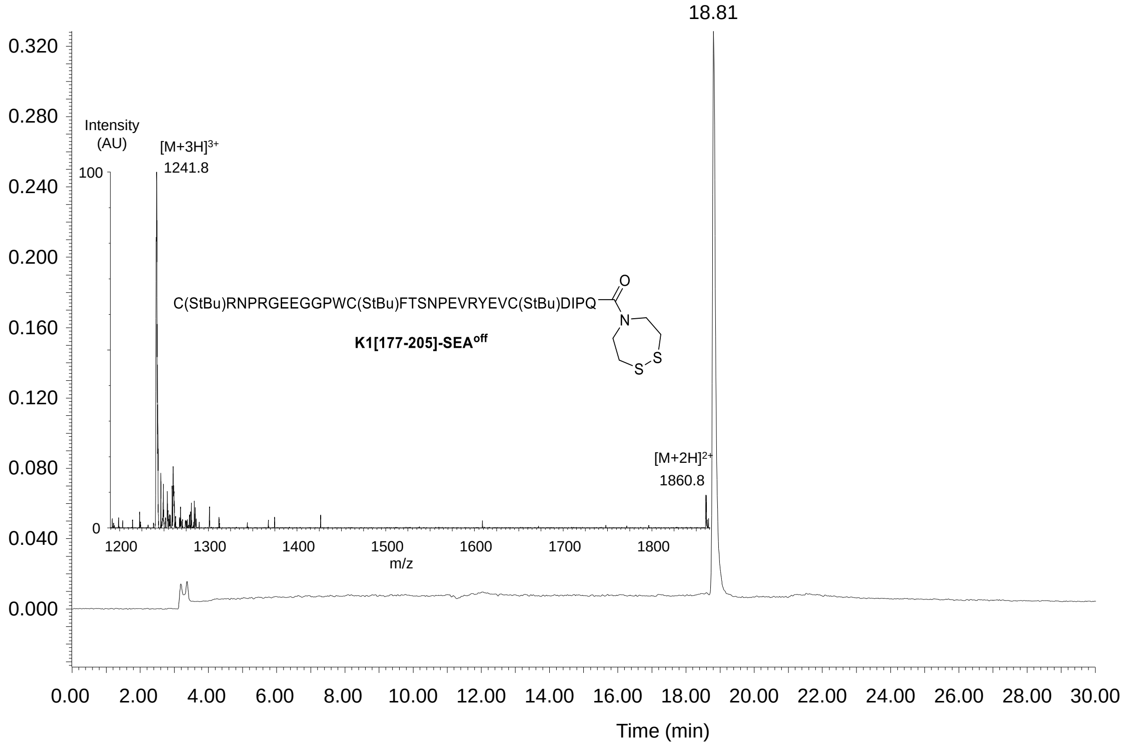

Supplementary Figure 38. LC-MS analysis of K1[177-205]-SEA^{off}. LC trace: eluent A 0.1% TFA in water, eluent B 0.1% TFA in CH_3CN/water 4/1 v/v, C18 column, gradient 0-100% B in 30 min, 30 °C, 1 mL min^{-1}, light scattering detection. MS trace: m/z = 1860.8 ($[M+2H]^{2+}$), 1241.8 ($[M+3H]^{3+}$); calcd. for M: 3721.4, found: 3721.0.

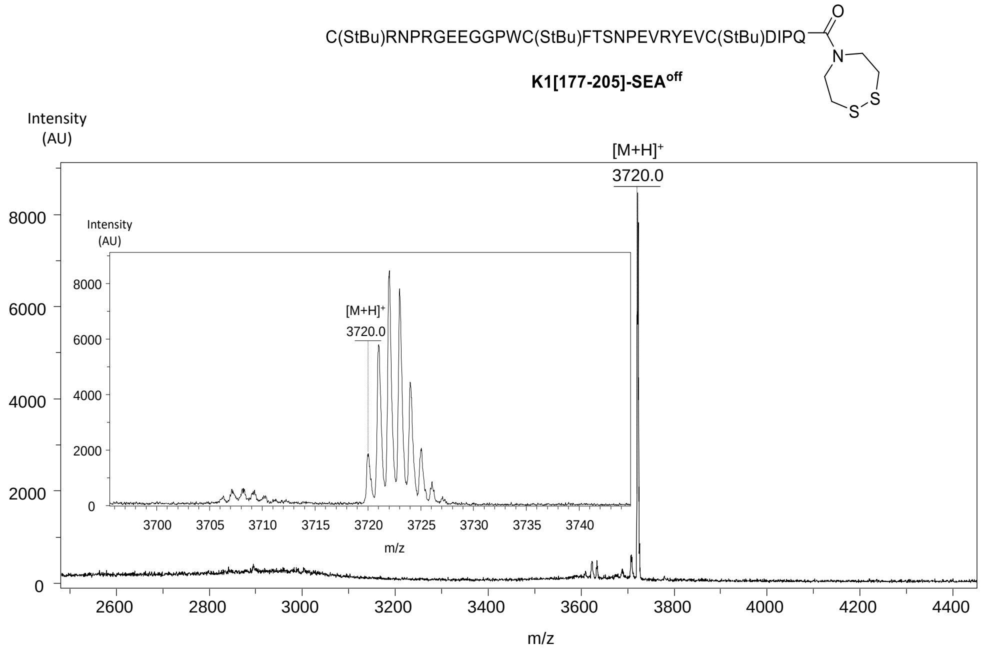

Supplementary Figure 39. MALDI-TOF analysis of K1[177-205]-SEA^{off}. Matrix α-cyano-4-hydroxycinnamic acid, positive detection mode, calcd. for $[M+H]^+$ (monoisotopic) 3719.6, found 3720.0.

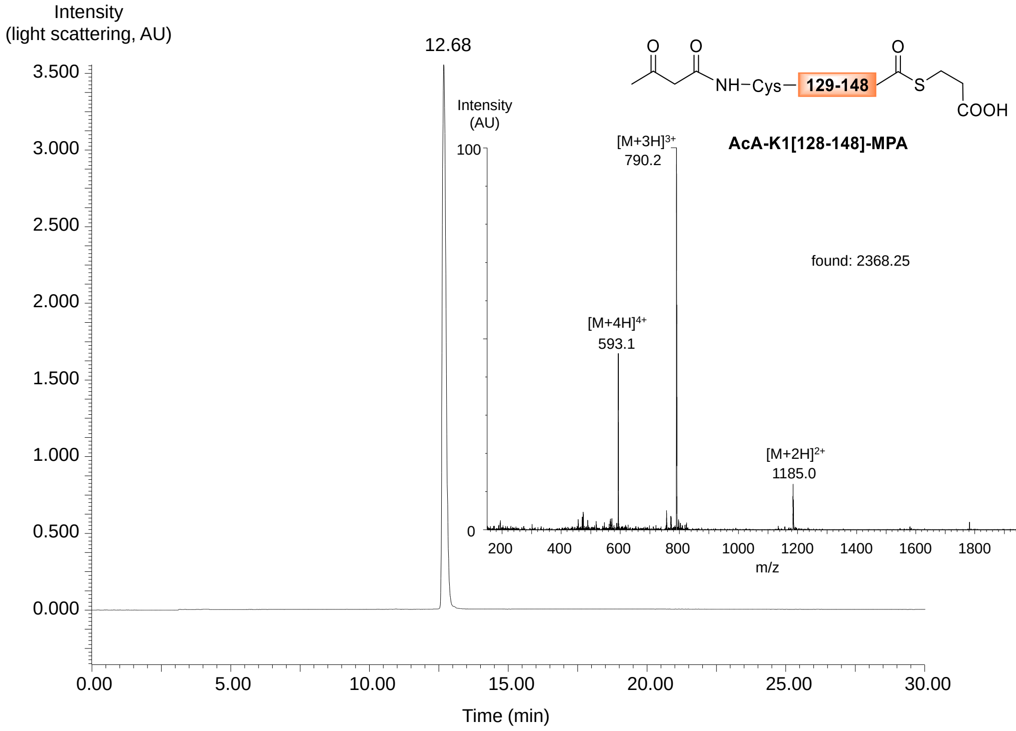

Supplementary Figure 40. LC-MS analysis of AcA-K1[128-148]-MPA. LC trace: eluent A 0.1% TFA in water, eluent B 0.1% TFA in CH_3CN/water 4/1 v/v, C18 Xbridge BEH 300 Å 5 µm (4.6 × 250 mm) column, 30 °C, gradient 0-100% B in 30 min, 1 mL min^{-1}, light scattering detection. MS trace: m/z = 1185.0 ($[M+2H]^{2+}$), 790.2 ($[M+3H]^{3+}$), 593.1 ($[M+4H]^{4+}$); calcd. for M (average): 2368.8, found: 2368.2.

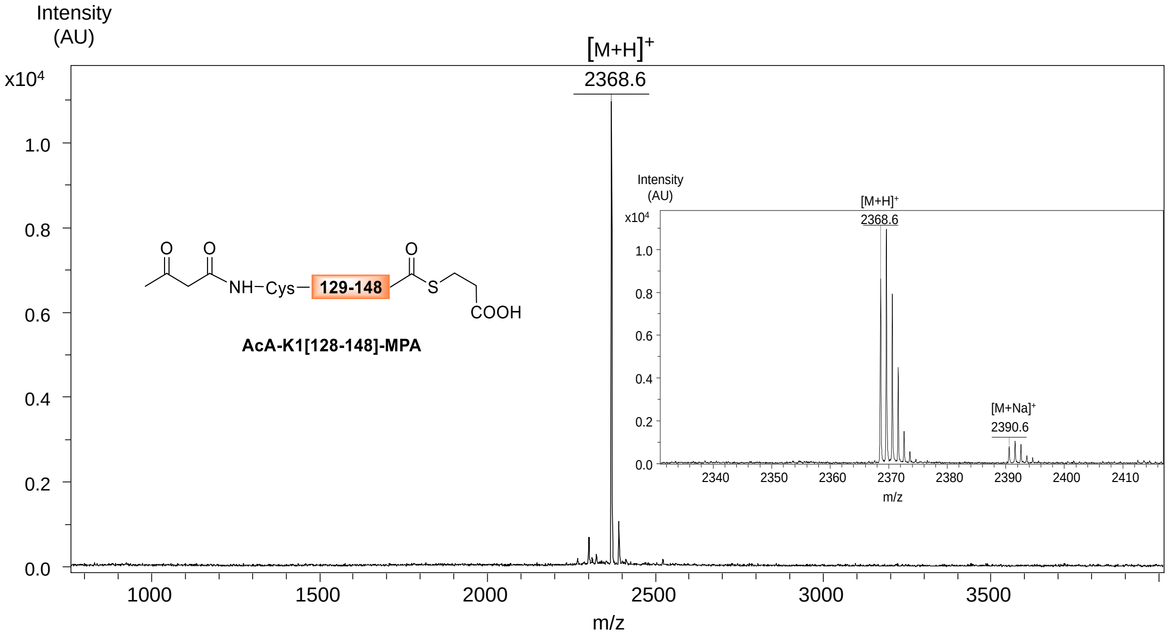

Supplementary Figure 41. MALDI-TOF analysis of AcA-K1[128-148]-MPA. Matrix 2,5-dihydroxybenzoic acid, positive detection mode, calcd. for $[M+H]^+$ (monoisotopic) 2368.3, found 2368.6.

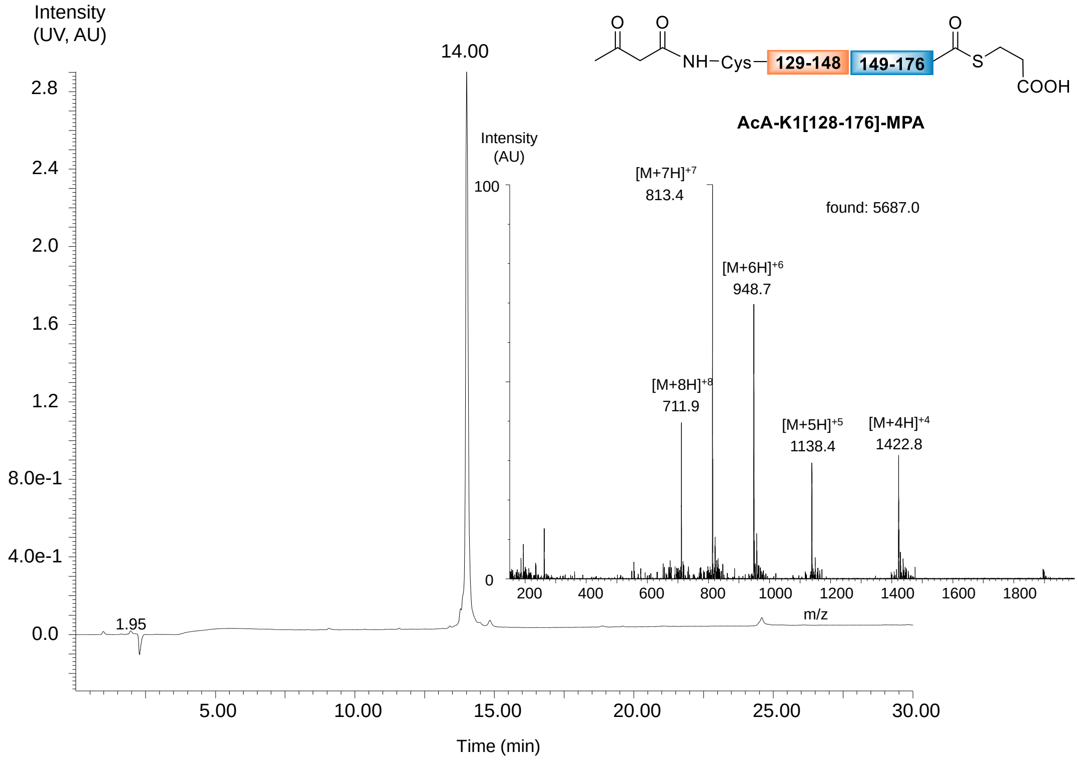

Supplementary Figure 42. LC-MS analysis of AcA-K1[128-176]-MPA. LC trace: eluent A 0.10% TFA in water, eluent B 0.10% TFA in CH_3CN/water 4/1 by vol. C18 Xbridge BEH 300 Å 5 µm (4.6 × 250 mm) column, 50 °C, gradient 0-100% B in 30 min, 1 mL min^{-1}, UV detection. MS trace: m/z = 1422.8 ($[M+4H]^{4+}$), 1138.4 ($[M+5H]^{5+}$), 948.7 ($[M+6H]^{6+}$), 813.4 ($[M+7H]^{7+}$), 711.9 ($[M+8H]^{8+}$); calcd. for M: 5688.5, found: 5687.0.

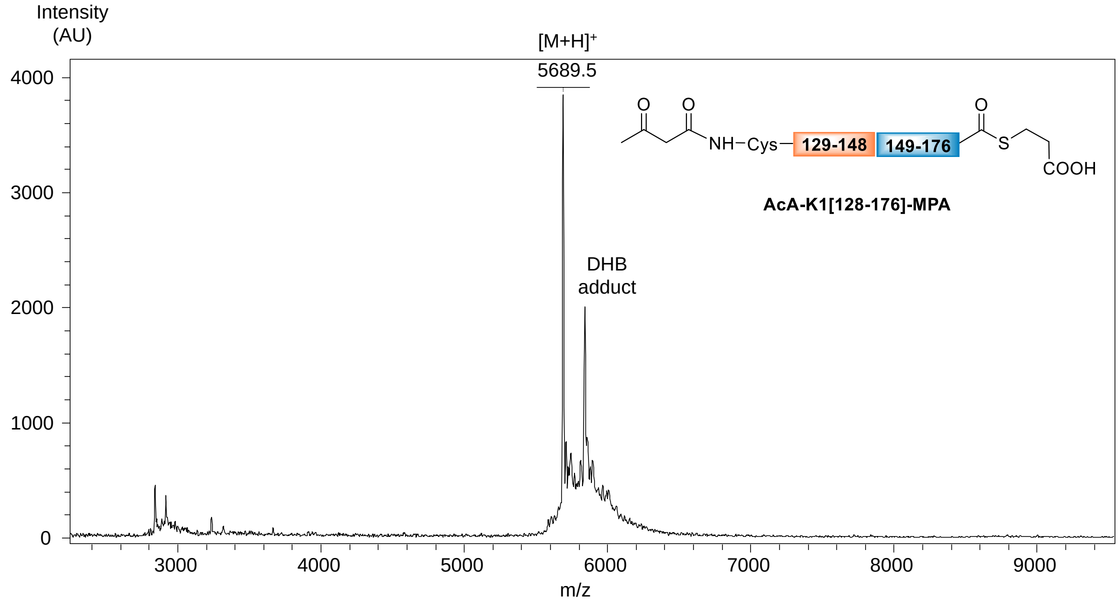

Supplementary Figure 43. MALDI-TOF analysis of AcA-K1[128-176]-MPA. Matrix 2,5-dihydroxybenzoic acid, positive detection mode, calcd. for $[M+H]^+$ (average) 5689.5, found 5689.5.

a)

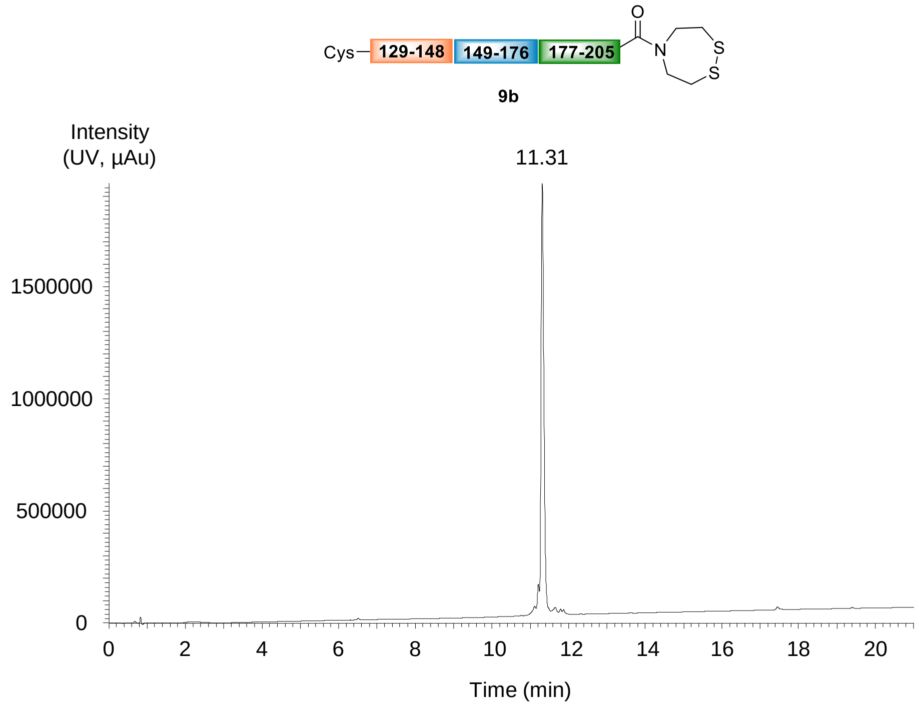

b)

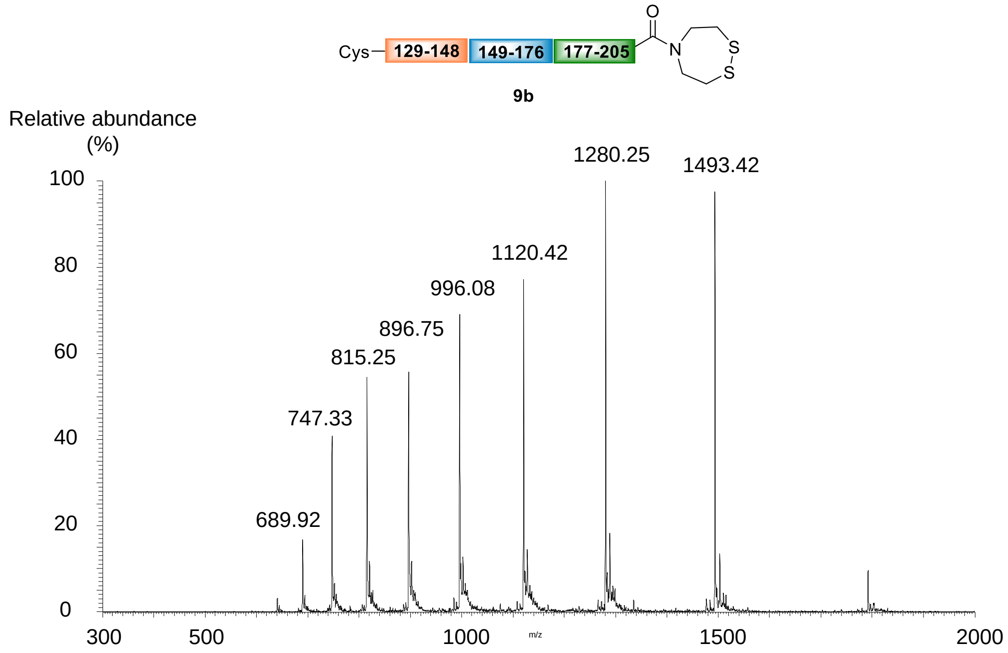

Supplementary Figure 44. LC-MS analysis of the K1 analogue **9b**. a) LC trace. Eluent A 0.1% TFA in water, eluent B 0.1% TFA in CH_3CN, ACQUITY UPLC Peptide BEH 300Å 1.7 µm (2.1 × 150 mm) column, 50 °C, gradient 0-70% B in 20 min, 0.4 mL min^{-1}, UV detection. b) MS trace: m/z = 1493.42 ($[M+6H]^{6+}$), 1280.25 ($[M+7H]^{7+}$), 1120.42 ($[M+8H]^{8+}$), 996.08 ($[M+9H]^{9+}$), 896.75 ($[M+10H]^{10+}$), 815.25 ($[M+11H]^{11+}$), 747.33 ($[M+12H]^{12+}$), 689.92 ($[M+13H]^{13+}$); calcd. for M (average): 8955.26, found: 8955.81.

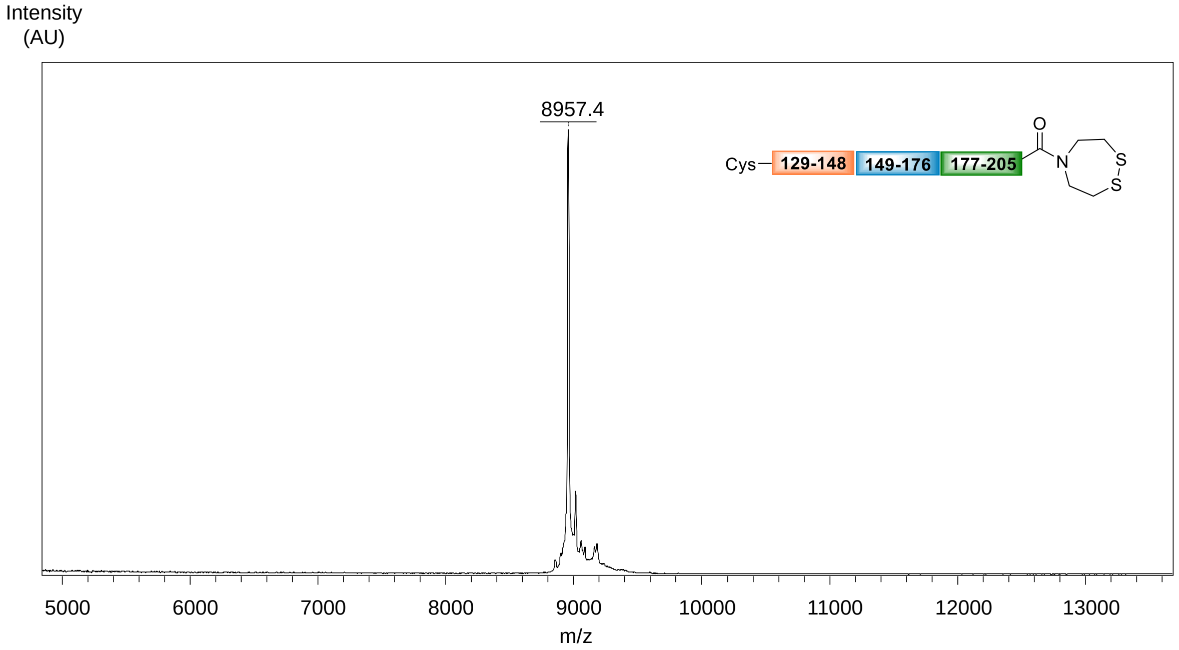

Supplementary Figure 45. MALDI-TOF analysis of the K1 analogue **9b**. Matrix 2,5-dihydroxybenzoic acid, positive detection mode, calcd. for $[M+H]^+$ (average) 8956.2, found: 8957.4.

Peptide thioesters

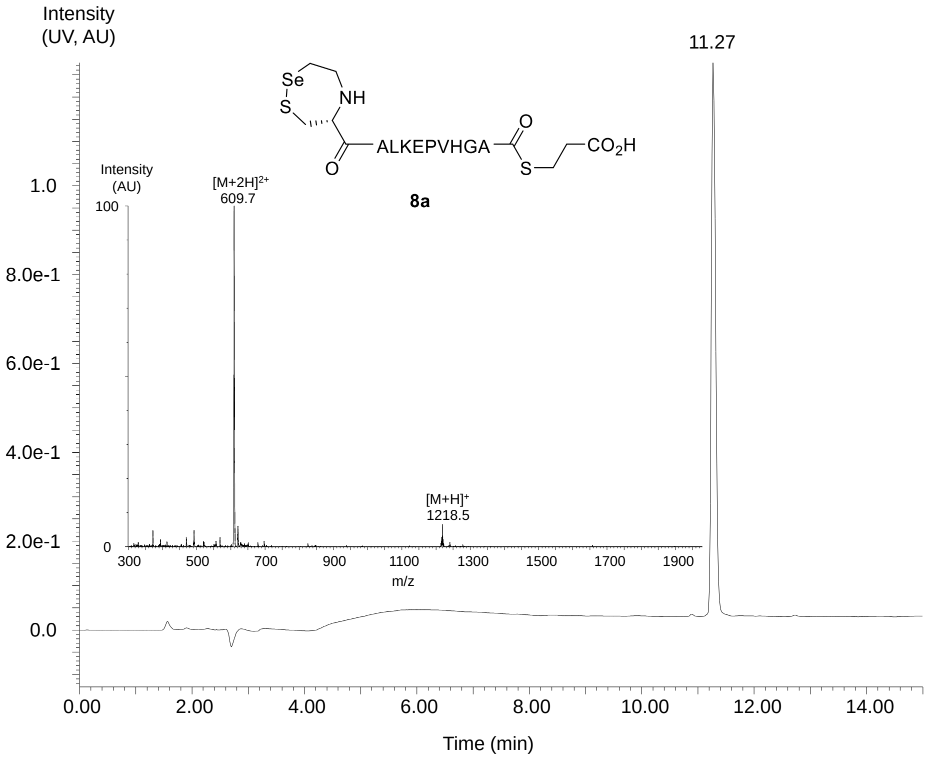

Supplementary Figure 46. LC-MS analysis of SetCys-ALKEPVHGA-MPA peptide **8a**. LC trace: eluent A 0.1% TFA in water, eluent B 0.1% TFA in CH_3CN/water 4/1 v/v, C18 column, gradient 0-50% B in 15 min, 30 °C, 1 mL min^{-1}, UV detection. MS trace: m/z calcd. for $[M+H]^+$ (monoisotopic mass): 1218.4, found: 1218.5.

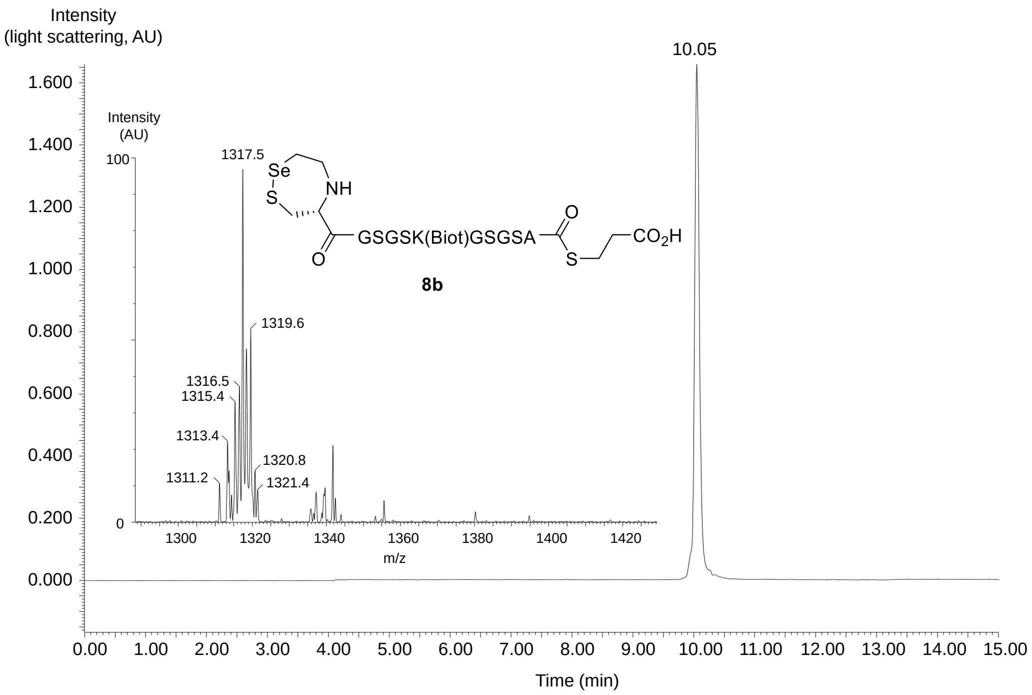

Supplementary Figure 47. LC-MS analysis of SetCys-$(GS)_2$-K(Biot)-$(GS)_2$A-MPA peptide **8b**. LC trace: eluent A 0.1% TFA in water, eluent B 0.1% TFA in CH_3CN/water 4/1 v/v. C18 column, gradient 0-50% B in 15 min, 30 °C, 1 mL min^{-1}, light scattering detection. MS trace: m/z calcd. for $[M+H]^+$ (monoisotopic mass): 1317.4, found: 1317.5.

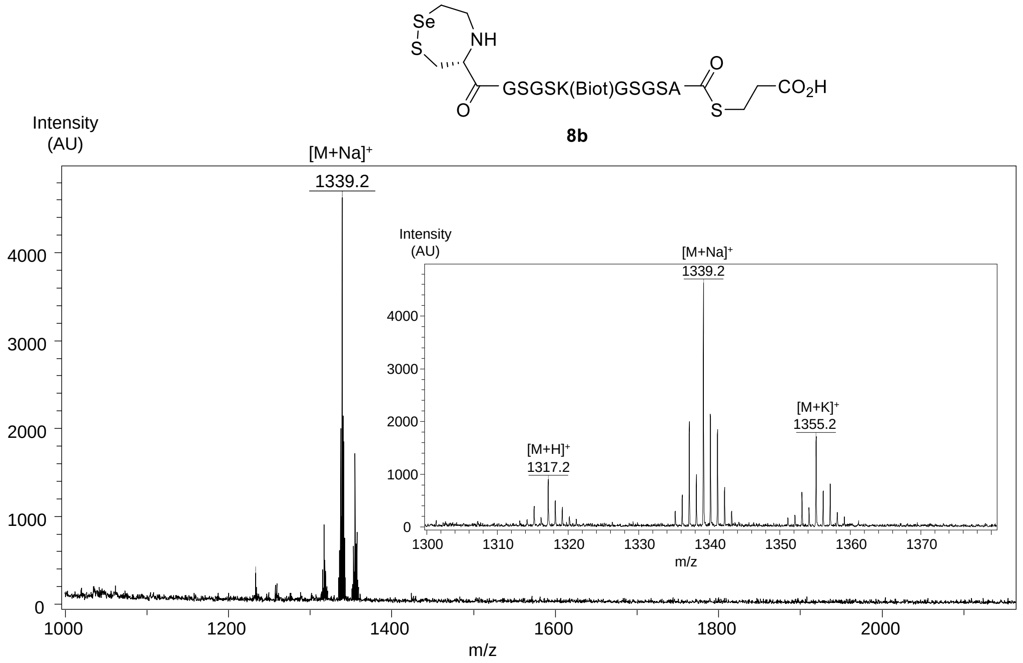

Supplementary Figure 48. MALDI-TOF analysis of SetCys-$(GS)_2$-K(Biot)-$(GS)_2$A-MPA peptide **8b**. Matrix α-cyano-4-hydroxycinnamic acid, positive detection mode, calcd. for $[M+Na]^+$ (monoisotopic): 1339.4, found: 1339.2.

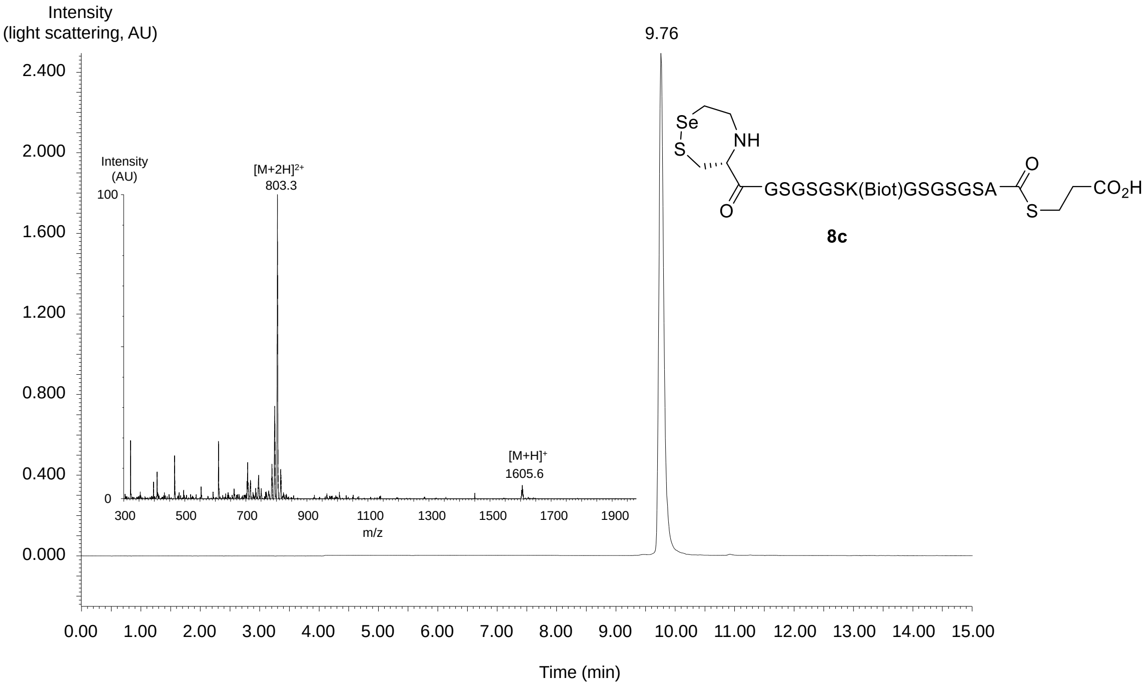

Supplementary Figure 49. LC-MS analysis of SetCys-(GS)3-K(Biot)-(GS)3A-MPA peptide **8c**. LC trace: eluent A 0.1% TFA in water, eluent B 0.1% TFA in CH_3CN/water 4/1 v/v, C18 column, gradient 0-50% B in 15 min, 30 °C, 1 mL min^{-1}, light scattering detection. MS trace: m/z calcd. for $[M+H]^+$ (monoisotopic mass): 1605.5, found: 1605.6.

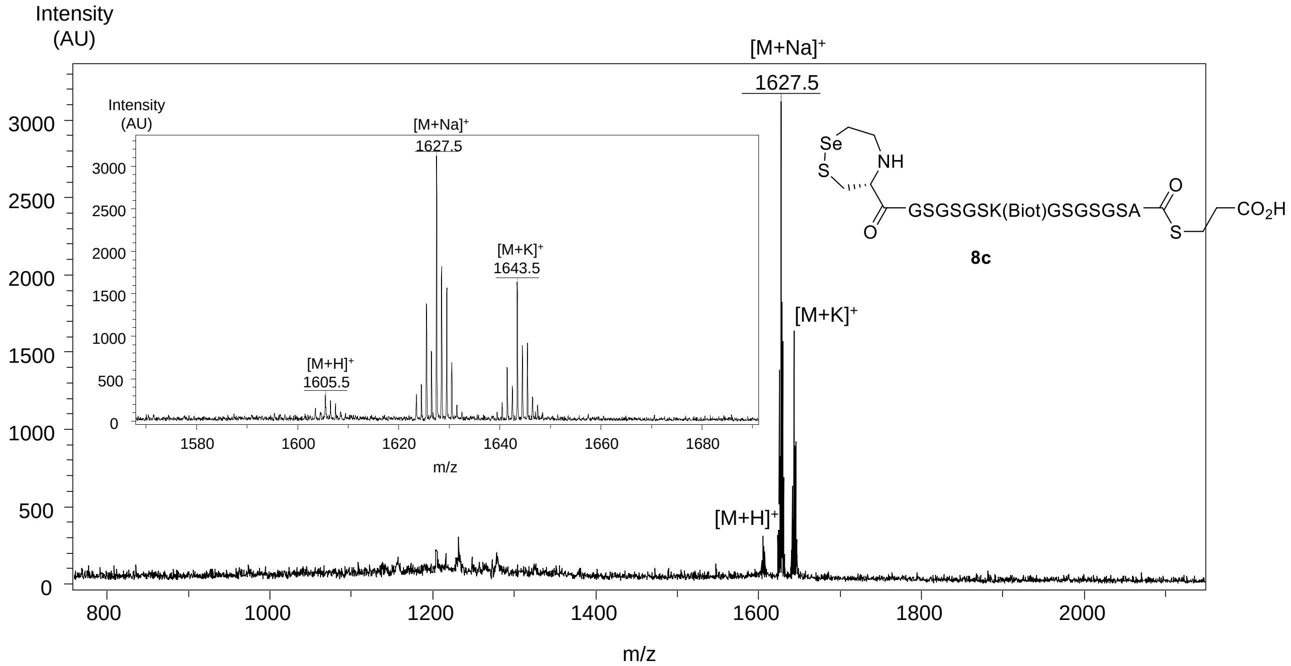

Supplementary Figure 50. MALDI-TOF analysis of SetCys-(GS)3-K(Biot)-(GS)3A-MPA peptide **8c**. Matrix α-cyano-4-hydroxycinnamic acid, positive detection mode, calcd. for $[M+Na]^+$ (monoisotopic): 1627.5, found: 1627.5.

a)

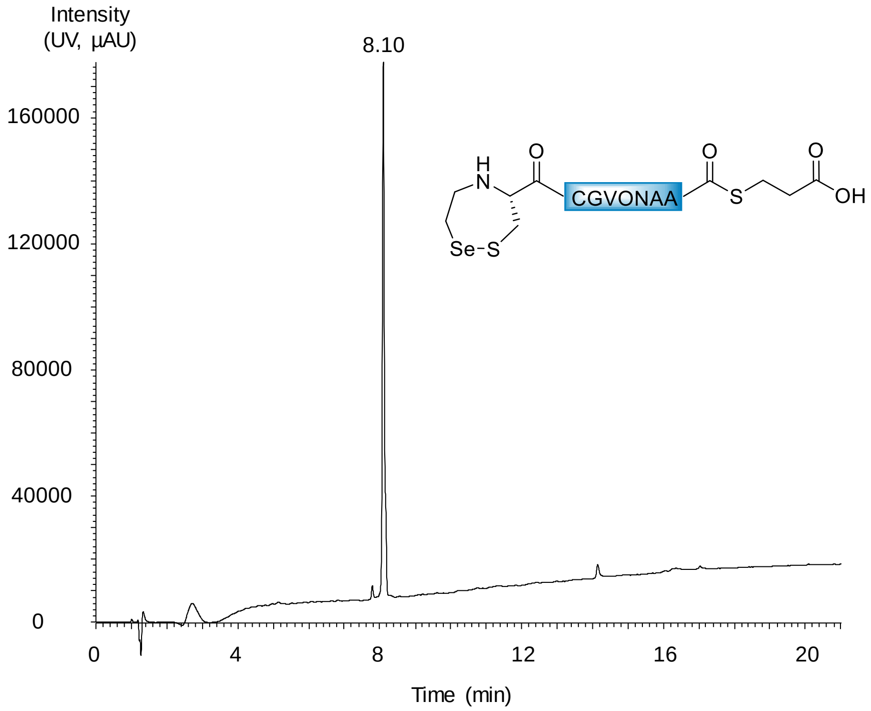

b)

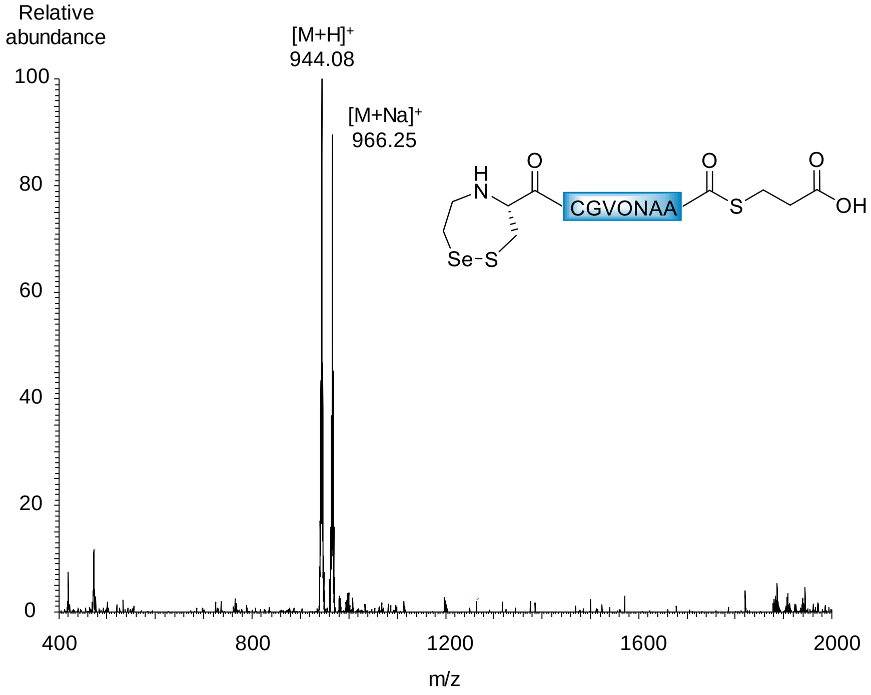

Supplementary Figure 51. UPLC-MS analysis of SetCys-CGVONAA-MPA. a) LC trace. Eluent A 0.1% TFA in water, eluent B 0.1% TFA in CH_3CN, ACQUITY UPLC Peptide BEH 300 Å 1.7 µm (2.1 × 150 mm) column, 50 °C, gradient 0-70% B in 20 min, 0.4 mL min^{-1}, UV detection. b) MS trace: Calcd. for $[M+H]^+$ (monoisotopic): 944.21, found: 944.08.

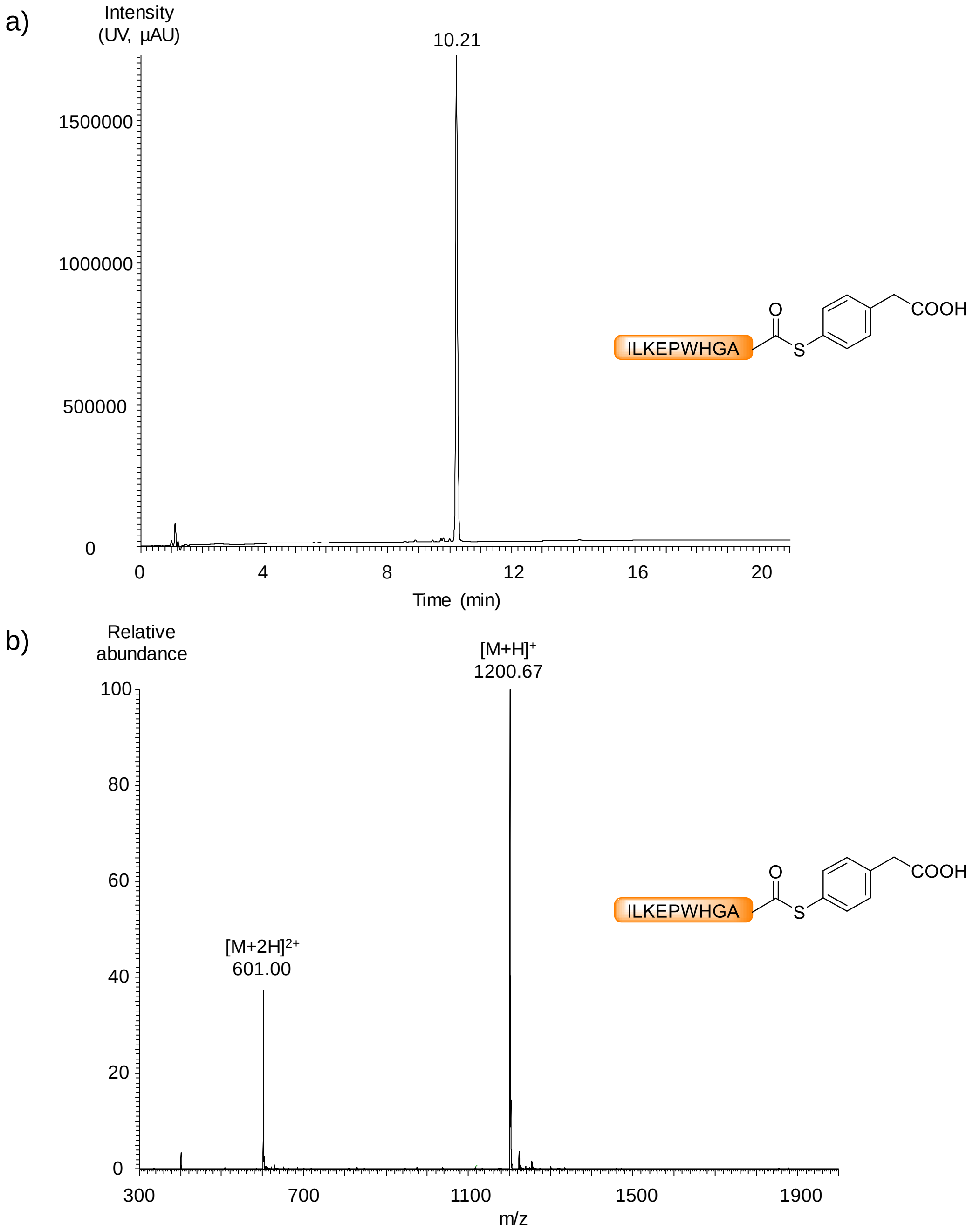

Supplementary Figure 52. LC-MS analysis of ILKEPWHGA-MPAA. a) LC trace. Eluent A 0.1% TFA in water, eluent B 0.1% TFA in CH_3CN, ACQUITY UPLC Peptide BEH 300 Å 1.7 µm (2.1 × 150 mm) column, 50 °C, gradient 0-70% B in 20 min, 0.4 mL min^{-1}, UV detection. b) MS trace: calcd. for $[M+H]^+$ (monoisotopic): 1200.58, found: 1200.67.

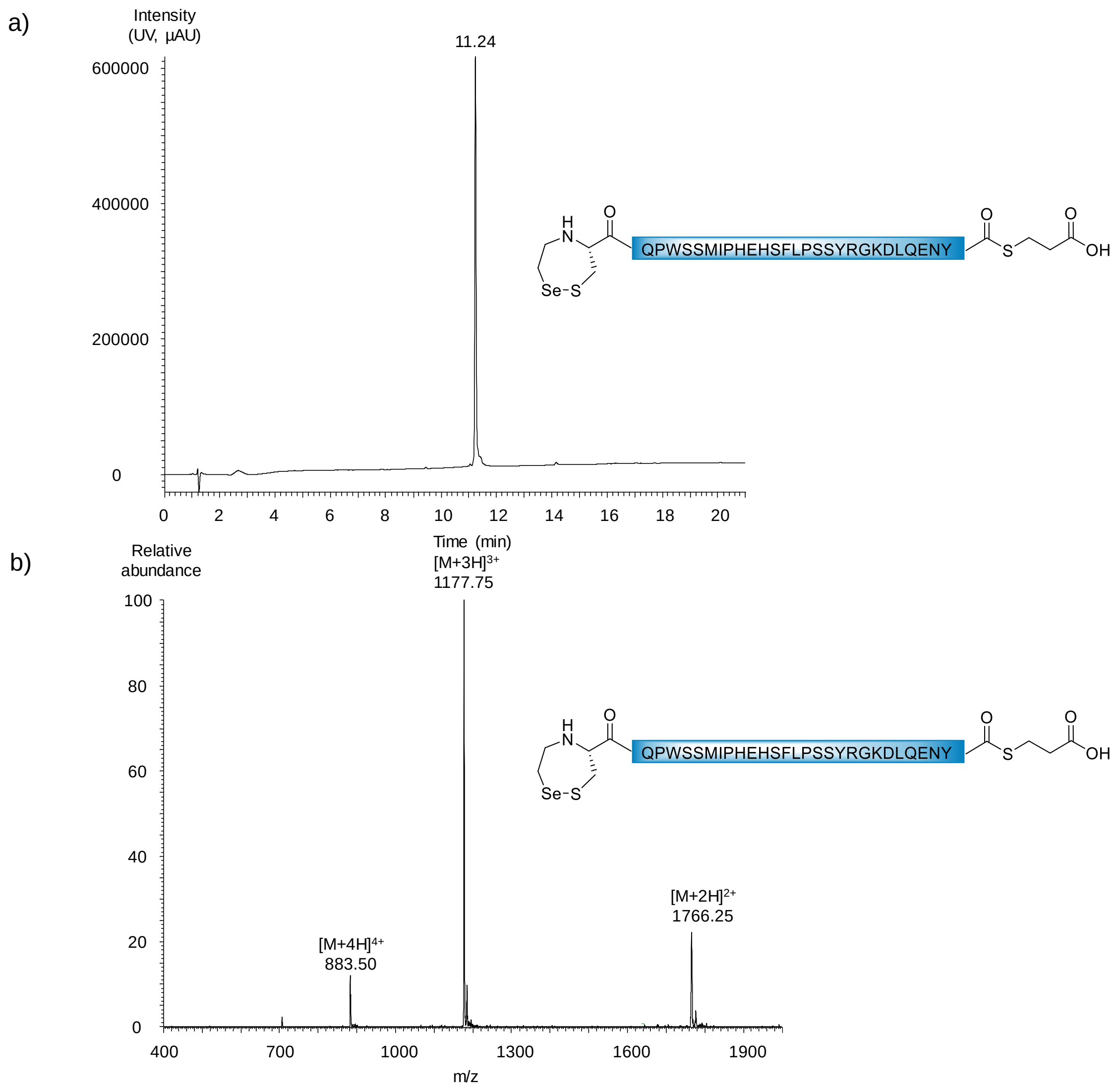

Supplementary Figure 53. UPLC-MS analysis of SetCys-K1[150-176]-MPA peptide **13**. a) LC trace. Eluent A: 0.1% TFA in water, eluent B: 0.1% TFA in CH_3CN, ACQUITY UPLC Peptide BEH 300 Å 1.7 μm (2.1 × 150 mm) column, 50 °C, gradient 0-70% B in 20 min, 0.4 mL min^{-1}, UV detection (215 nm). b) MS trace. m/z = 1766.25 ([M+2H]$^{2+}$), 1177.75 ([M+3H]$^{3+}$), 883.50 ([M+4H]$^{4+}$); calcd. for M (average): 3530.25, found: 3530.82.

Reactivity studies (Figure 2a)

Property 1

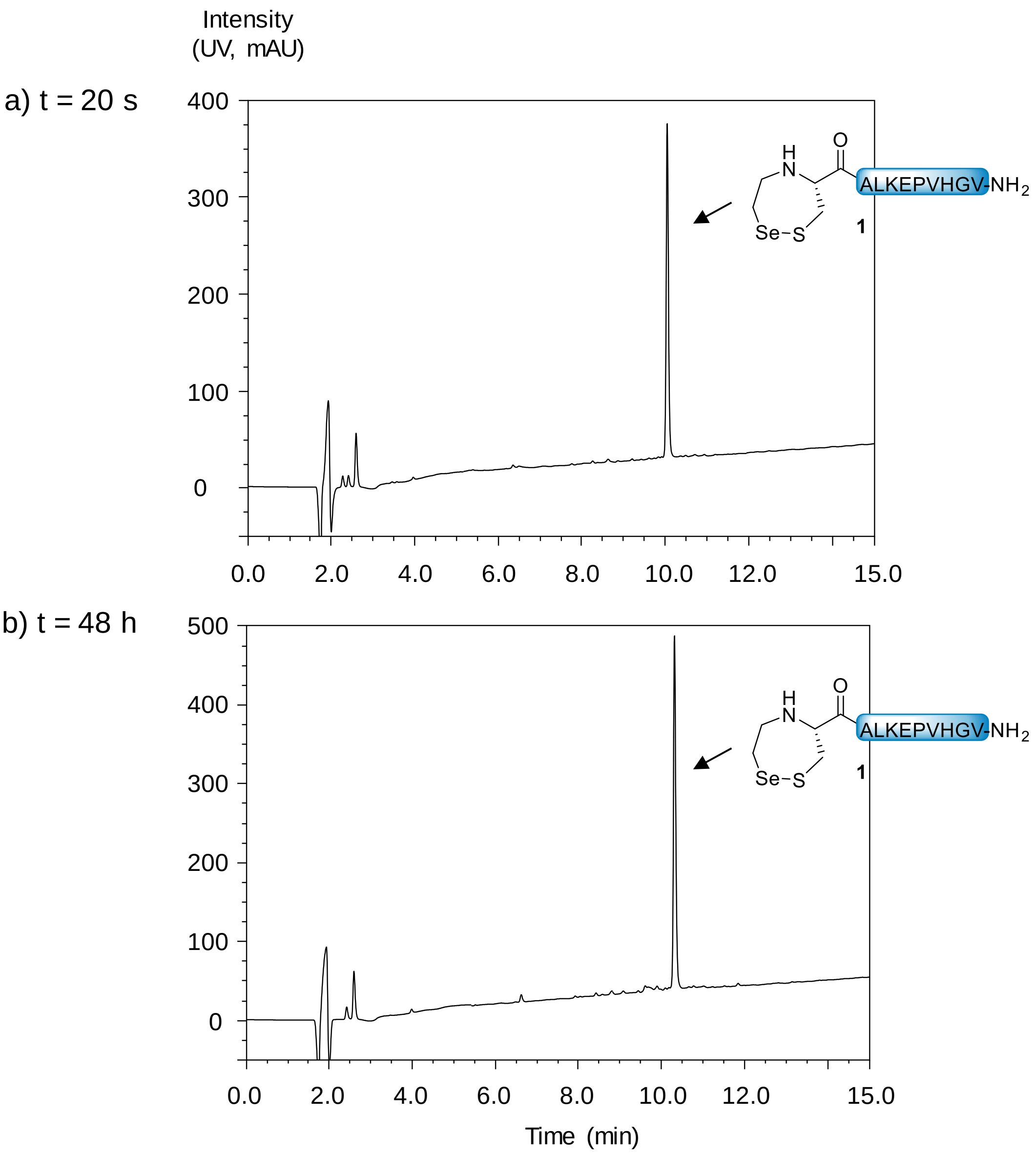

Supplementary Figure 54. Property 1. Stability of the SetCys peptide **1** in the presence of MPAA. a) LC trace after 20 s, b) LC trace after 48 h. Eluent A 0.1% TFA in water, eluent B 0.1% TFA in CH_3CN/water 4/1 v/v, C18 column, gradient 0-50% B in 15 min, 30 °C, 1 mL min^{-1}, UV detection at 215 nm.

Property 2

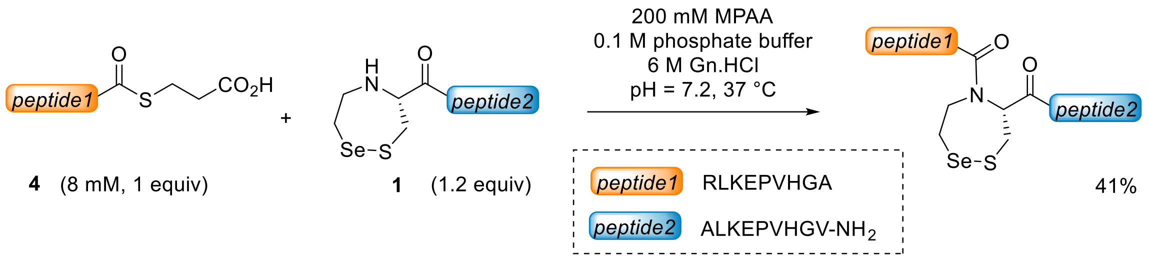

Supplementary Figure 55. Property 2. Ligation reaction of SetCys peptide **1** with peptidyl MPA-thioester **4**.

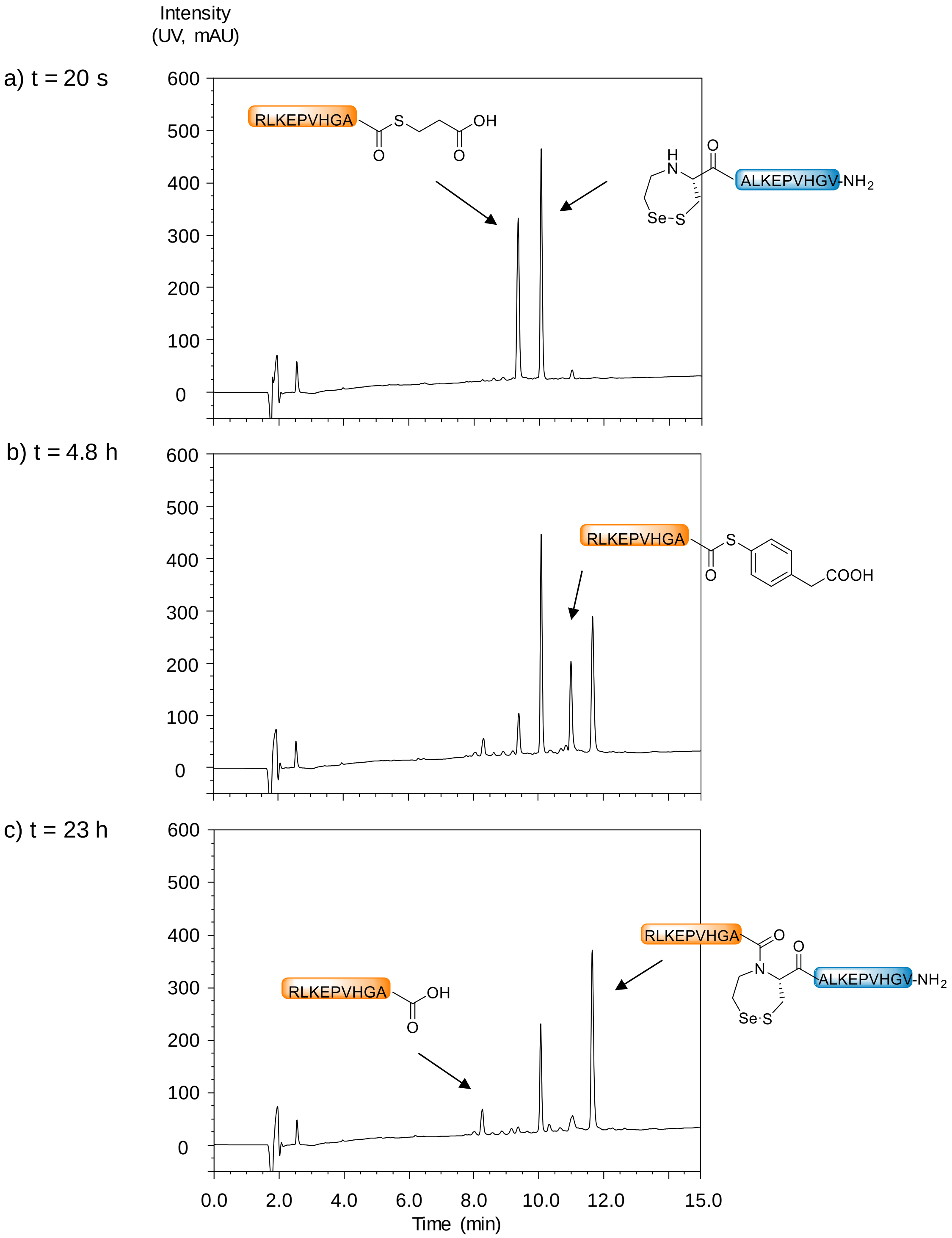

Supplementary Figure 56. Property 2. Monitoring of the ligation between SetCys peptide **1** and peptide thioester RLKEPVHGA-MPA **4**. a) LC trace after 20 s. b) LC trace after 4.8 h. c) LC trace after 23 h. For each point, a 2 µL aliquot was taken from the reaction mixture and quenched by adding 10% AcOH in water (100 µL). The sample was then extracted with Et_2O to remove MPAA prior to HPLC analysis. LC trace: eluent A 0.1% TFA in water, eluent B 0.1% TFA in CH_3CN/water 4/1 v/v, C18 column, gradient 0-50% B in 15 min, 30 °C, 1 mL min^{-1}, UV detection at 215 nm.

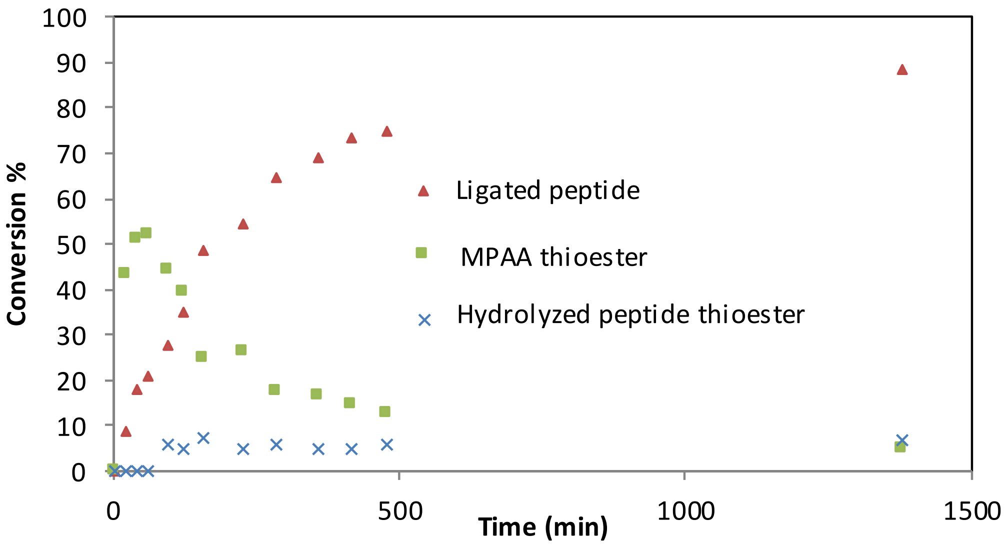

Supplementary Figure 57. Property 2. Ligation between SetCys peptide **1** and peptide thioester **4** performed under low reductive conditions (MPAA): evolution of the reaction mixture composition. The proportion of each species in the mixture was determined by HPLC using UV detection at 215 nm as shown in Supplementary Figure 56. The data show that the MPAA peptide thioester produced from RLKEPVHGA-MPA **4** by transthioesterification with MPAA quickly accumulates in the reaction mixture. This intermediate is then slowly consumed with concomitant formation of the ligated product.

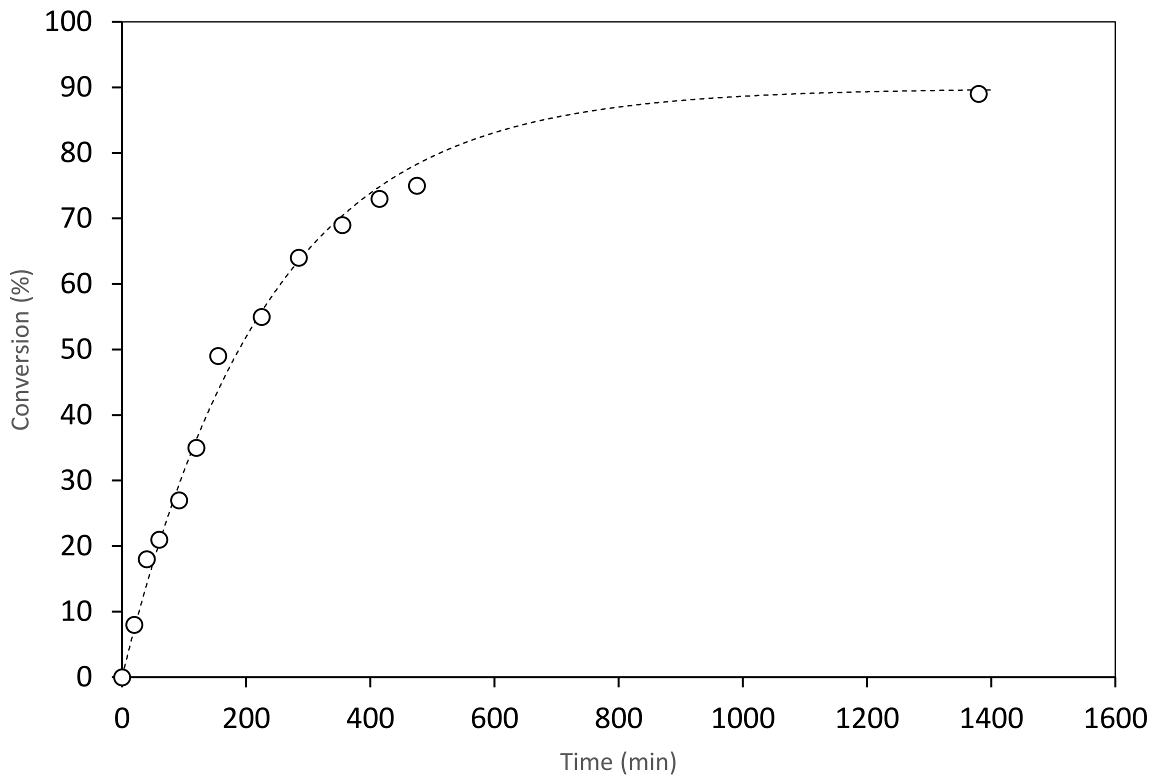

Supplementary Figure 58. Property 2. Fitting of the reaction between SetCys peptide **1** and peptide thioester **4** using Kintek Global Explorer Software (4.31 ± 1.4) × 10^{-3} min^{-1}; $t_{1/2}$ = 2.57 h). Circles correspond to experimental values.

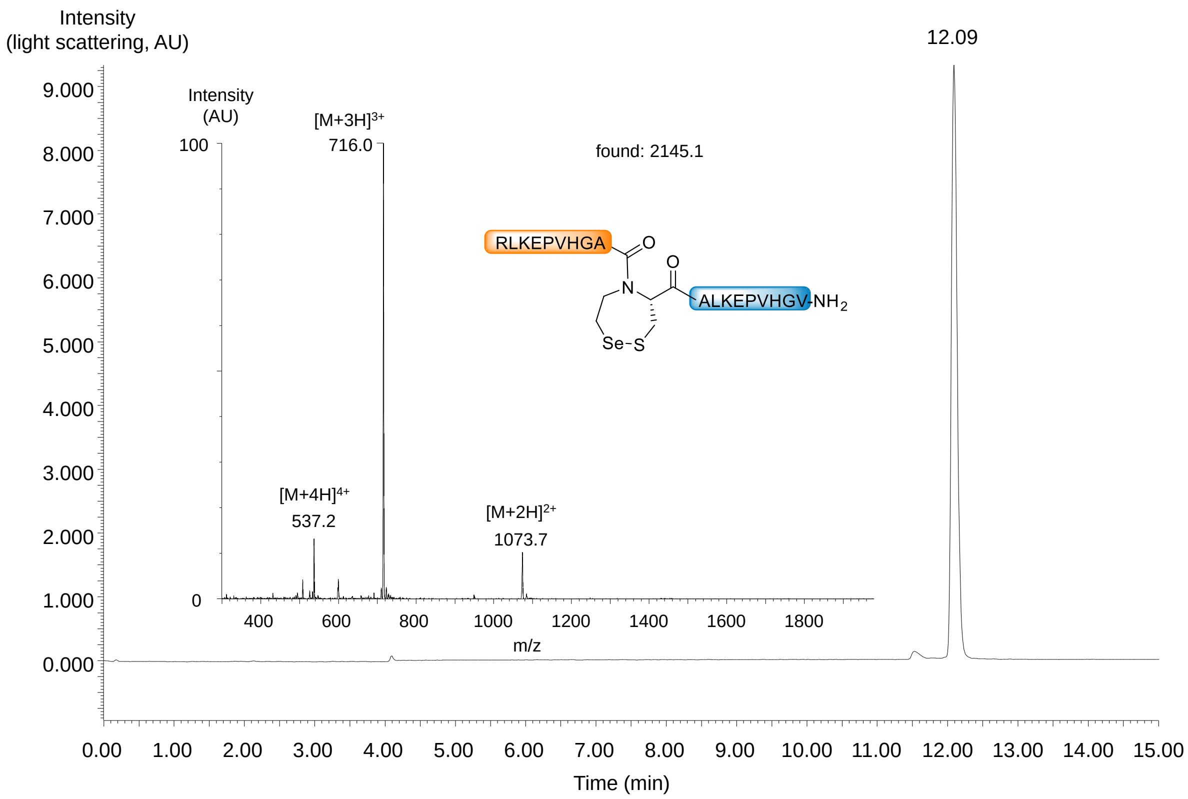

Supplementary Figure 59. LC-MS analysis of RLKEPVHGA-SetCys-ALKEPVHGV-NH_2 peptide. LC trace: eluent A 0.1% TFA in water, eluent B 0.1% TFA in CH3CN/water 4/1 v/v. C18 Xbridge BEH 300 Å 5 µm (4.6 × 250 mm) column, gradient 0-50% B in 15 min, 30 °C, 1 mL min^{-1}, light scattering detection. MS trace: 1073.7 ([M+2H]2+), 716.0 ([M+3H]3+), 537.2 ([M+4H]4+); calcd. for M (average): 2144.4, found: 2145.1.

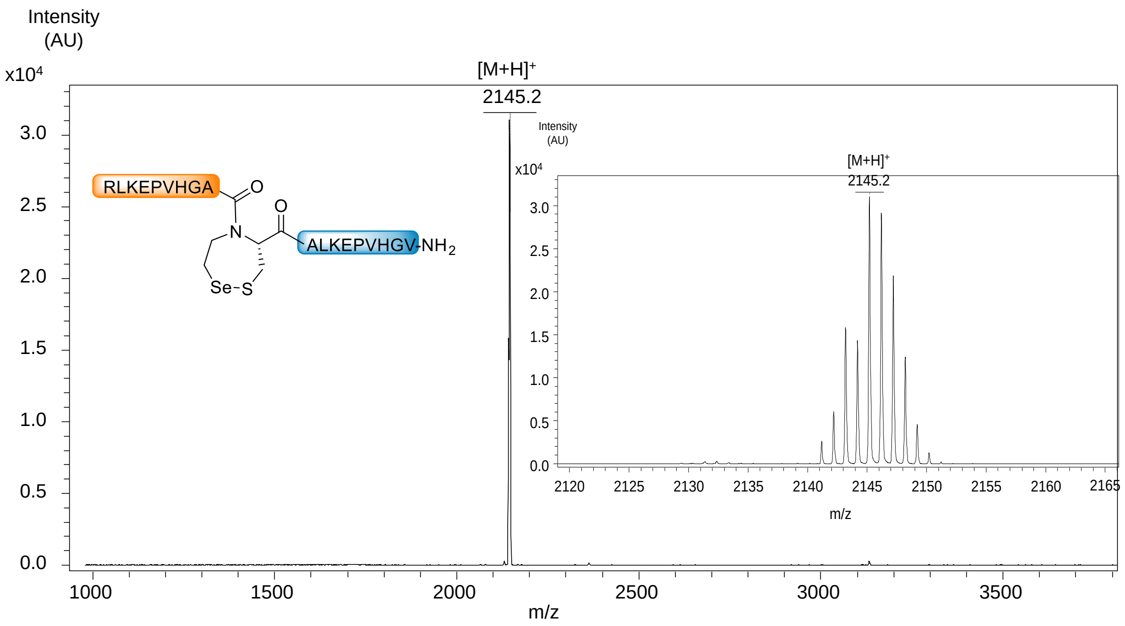

Supplementary Figure 60. MALDI-TOF analysis of RLKEPVHGA-SetCys-ALKEPVHGV-NH_2 peptide. Matrix α-cyano-4-hydroxycinnamic acid, positive detection mode, calcd. for $[M+H]^+$ (monoisotopic): 2145.1, found: 2145.2.

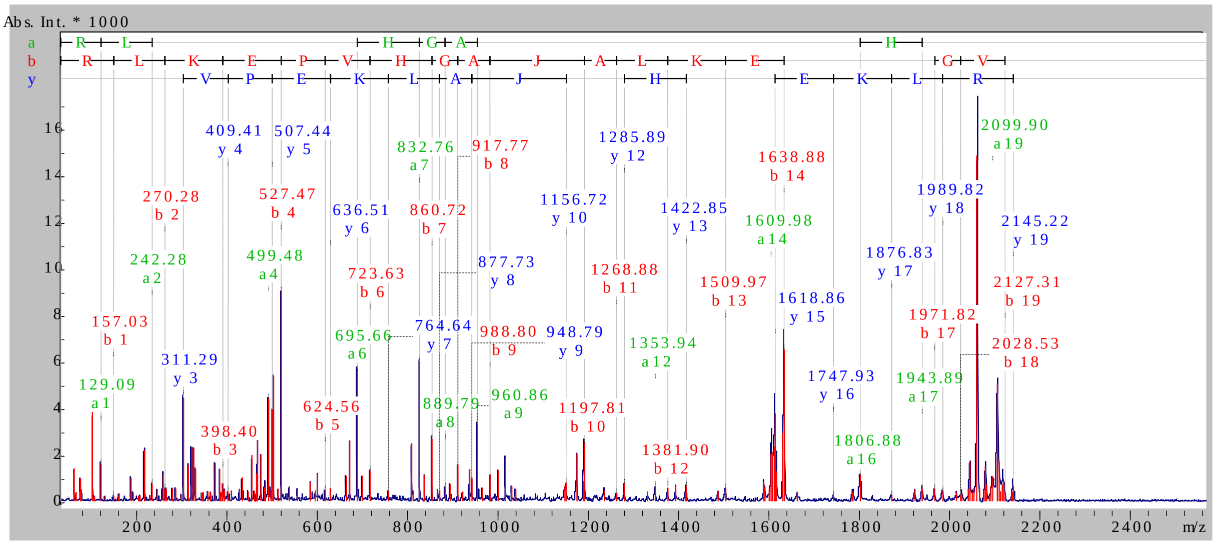

Supplementary Figure 61. MALDI-TOF-TOF sequencing of RLKEPVHGA-SetCys-ALKEPVHGV-NH_2 peptide. Matrix α-cyano-4-hydroxycinnamic acid, positive detection mode. The MS-MS analysis confirms the presence of SetCys (J on the figure) residue in the sequence of the peptide.

Property 3

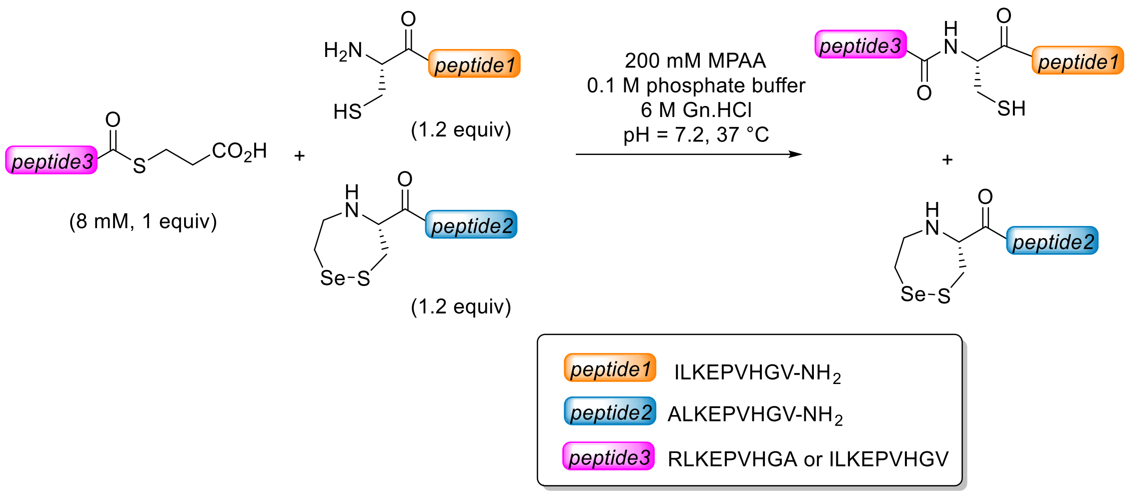

Supplementary Figure 62. Property 3. SetCys is silent during NCL under low reducing conditions.

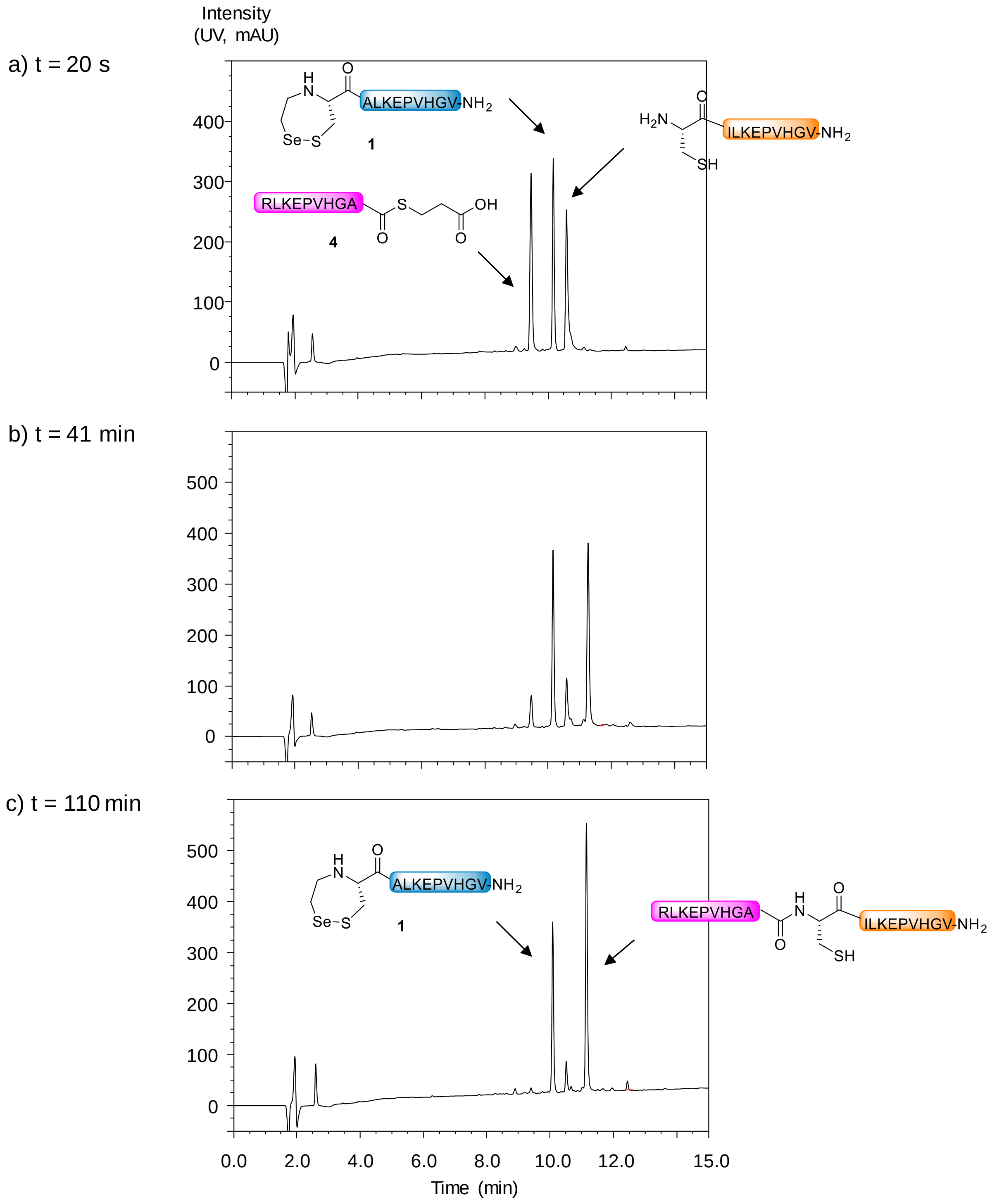

Supplementary Figure 63. Property 3. Monitoring of the competitive reaction between SetCys peptide **1** and cysteinyl peptide CILKEPVHGV-NH_2 with peptidyl MPA-thioester **4**. a) LC trace after 20 s. b) LC trace after 41 min. c) LC trace after 110 min. For each point, a 2 µL aliquot was taken from the reaction mixture and quenched by adding 10% AcOH in water (100 µL). The sample was then extracted with Et_2O to remove MPAA prior to HPLC analysis. Eluent A 0.1% TFA in water, eluent B 0.1% TFA in CH_3CN/water 4/1 v/v, C18 column, gradient 0-50% B in 15 min, 30 °C, 1 mL min^{-1}, UV detection at 215 nm.

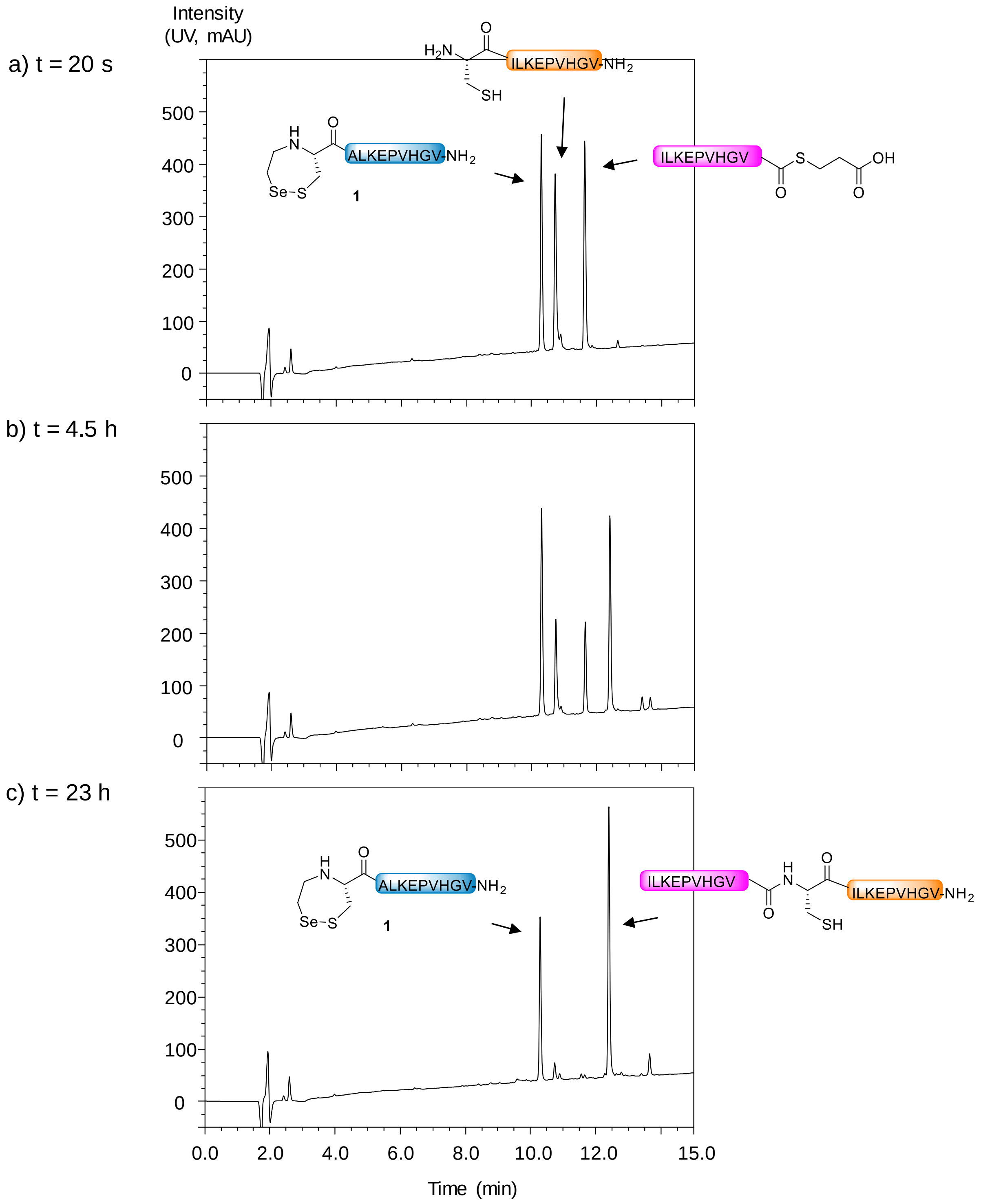

Supplementary Figure 64. Property 3. Monitoring of the competitive reaction between SetCys peptide **1** and cysteinyl peptide CILKEPVHGV-NH_2 with peptidyl MPA-thioester ILKEPVHGV-MPA. a) LC trace after 20 s. b) LC trace after 4.5 h. c) LC trace after 23 h. For each point, a 2 µL aliquot was taken from the reaction mixture and quenched by adding 10% AcOH in water (100 µL). The sample was then extracted with Et_2O to remove MPAA prior to HPLC analysis. Eluent A 0.1% TFA in water, eluent B 0.1% TFA in CH_3CN/water 4/1 v/v, C18 column, gradient 0-50% B in 15 min, 30 °C, 1 mL min^{-1}, UV detection at 215 nm.

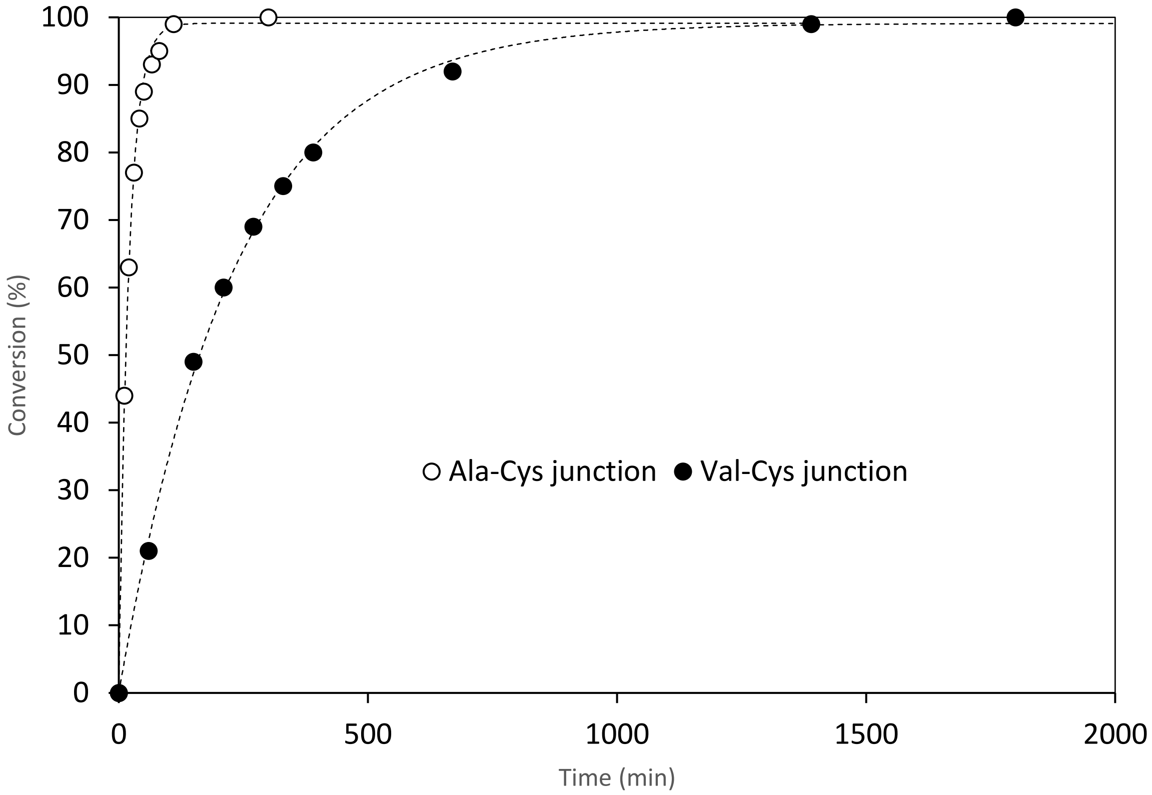

Supplementary Figure 65. Property 3. Competitive ligation between SetCys and Cys peptides using RLKEPVHGA-MPA **4** or ILKEPVHGV-MPA as acyl donors. The continuous curves correspond to the fitting to a pseudo first order kinetic law from which t½ and k were extracted.

Property 4

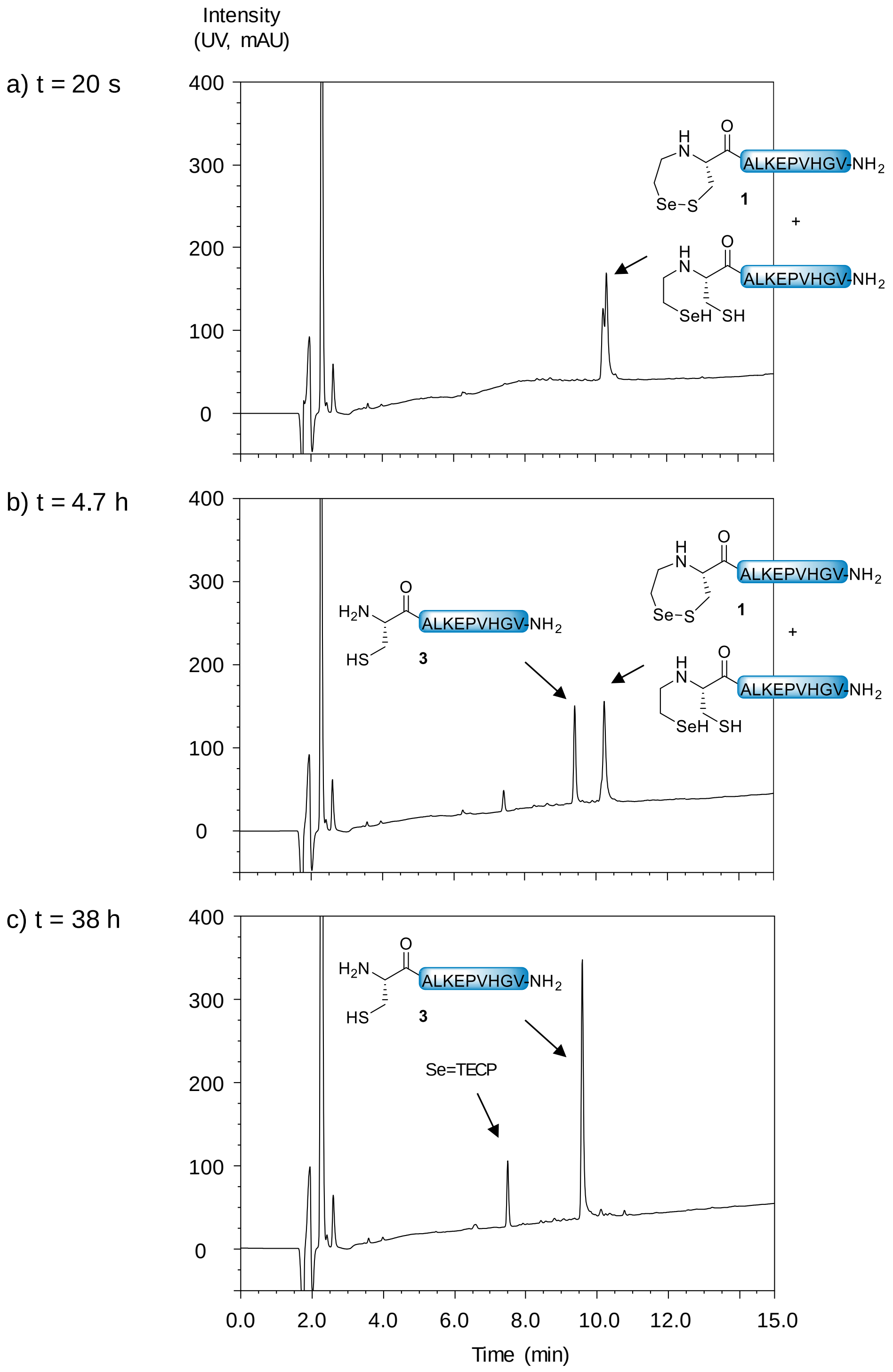

Supplementary Figure 66. Property 4. Monitoring of the conversion of SetCys peptide **1** into cysteinyl peptide **3** using MPAA + TCEP. a) LC trace after 20 s. b) LC trace after 4.7 h. c) LC trace after 38 h. Eluent A 0.1% TFA in water, eluent B 0.1% TFA in CH_3CN/water 4/1 v/v, C18 column, gradient 0-50% B in 15 min, 30 °C, 1 mL min^{-1}, UV detection at 215 nm.

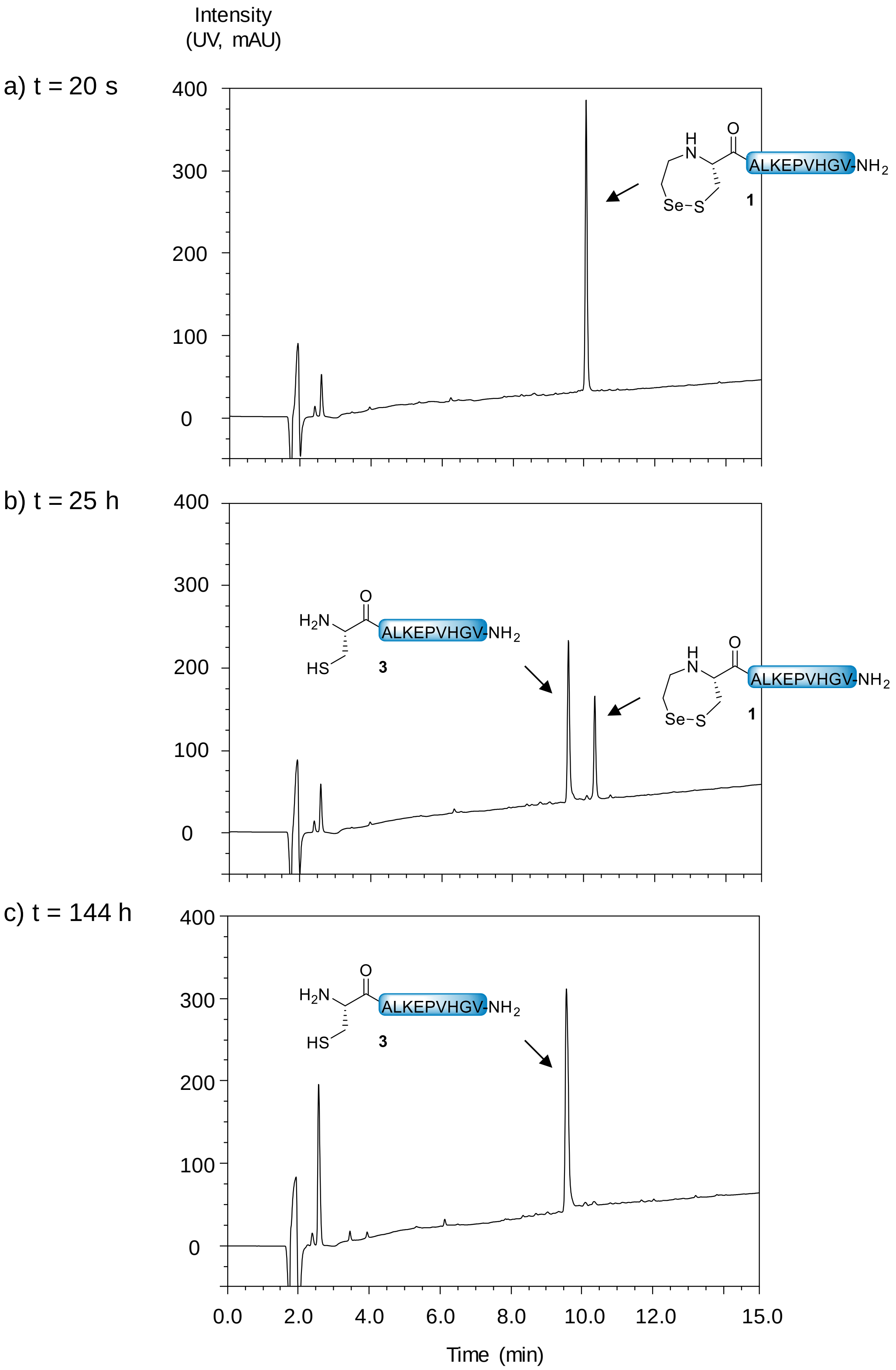

Supplementary Figure 67. Property 4. Monitoring of the conversion of SetCys peptide **1** into cysteinyl peptide **3** using DTT. a) LC trace after 20 s. b) LC trace after 25 h. c) LC trace after 144 h. Eluent A 0.1% TFA in water, eluent B 0.1% TFA in CH_3CN/water 4/1 v/v, C18 column, gradient 0-50% B in 15 min, 30 °C, 1 mL min^{-1}, UV detection at 215 nm.

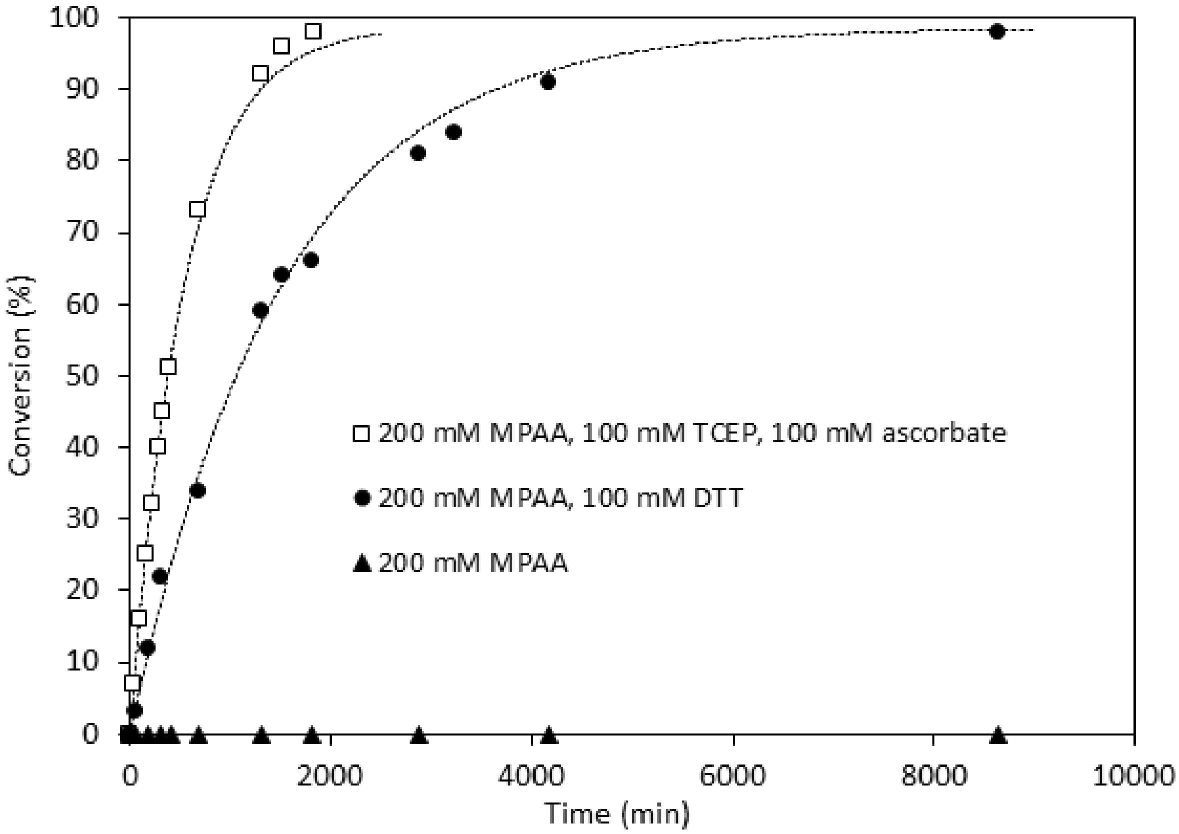

Supplementary Figure 68. Property 4. Conversion of SetCys peptide **1** into Cys peptide **3** in presence of TCEP or DTT. The continuous curves correspond to the fitting to a pseudo first order kinetic law from which t½ and k_1 were extracted.

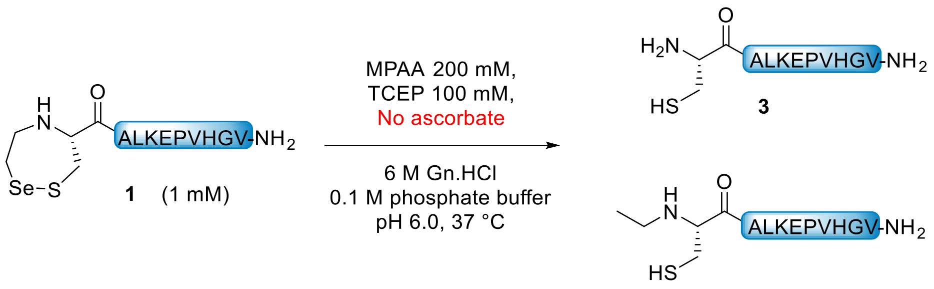

Supplementary Figure 69. Property 4. Treatment of SetCys by TCEP in absence of ascorbate.

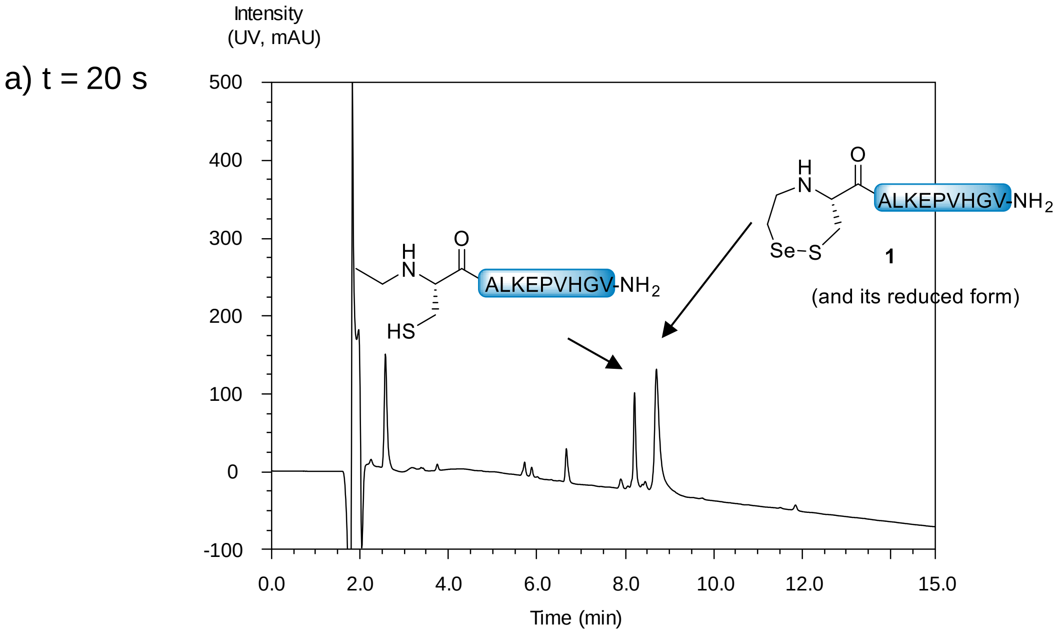

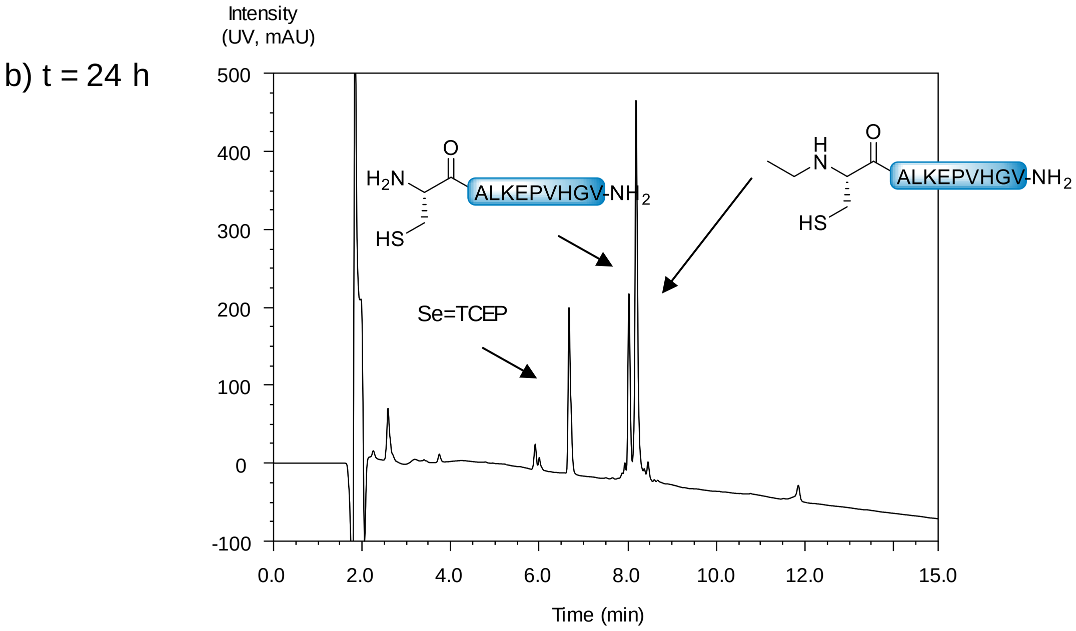

Supplementary Figure 70. Property 4. HPLC monitoring of the treatment of SetCys peptide **1** by TCEP in the absence of ascorbate performed at pH 6.0. a) LC trace after 20 s. b) LC trace after 24 h. Eluent A: 0.1% TFA in water, eluent B: 0.1% TFA in CH_3CN, gradient: 0-50% B in 15 min, 30 °C, 1 mL min^{-1}, C18 column, UV detection (215 nm).

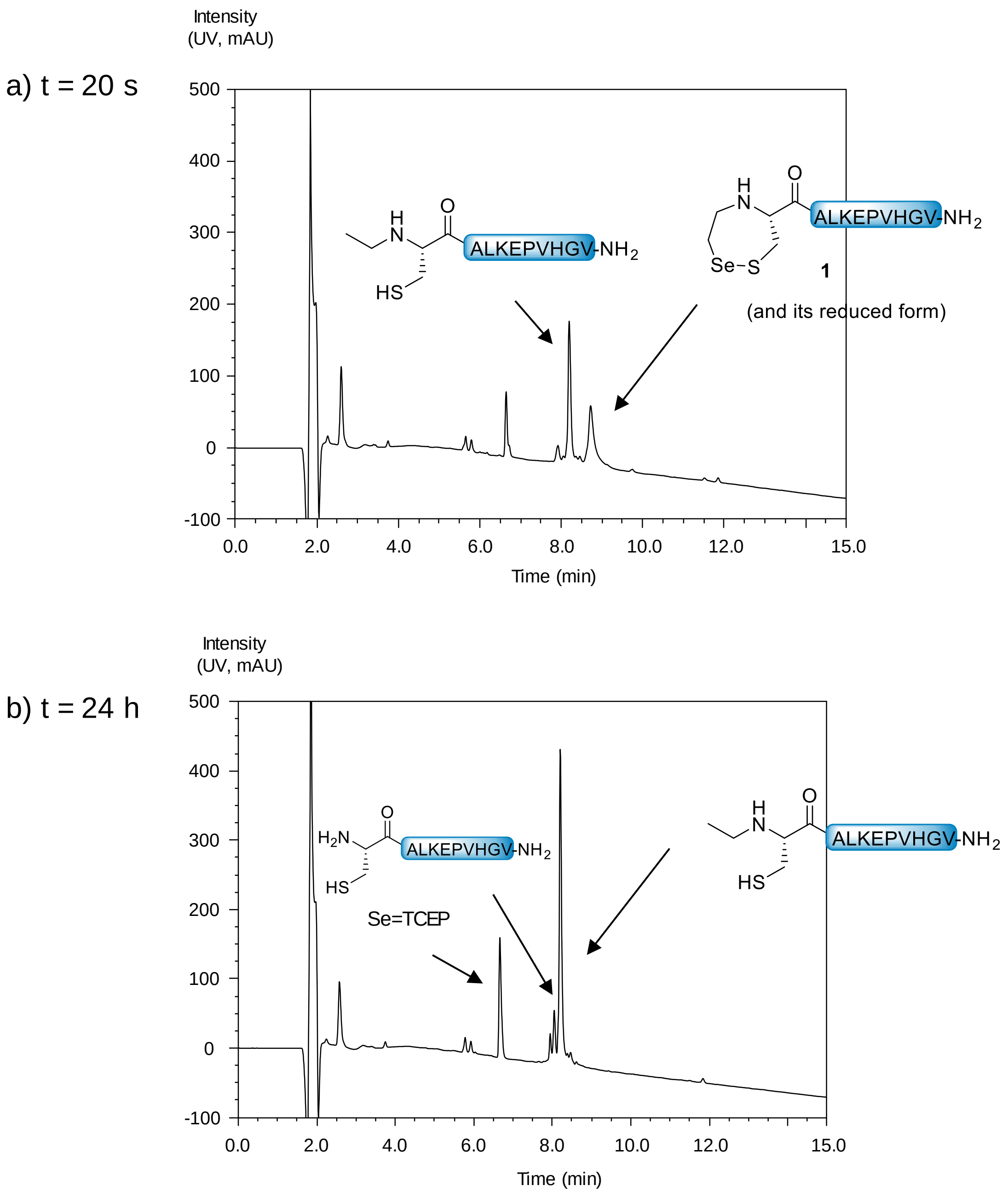

Supplementary Figure 71. Property 4. HPLC monitoring of the treatment of SetCys peptide **1** by TCEP in the absence of ascorbate performed at pH 7.2. a) LC trace after 20 s. b) LC trace after 24 h. Eluent A: 0.1% TFA in water, eluent B: 0.1% TFA in CH_3CN, gradient: 0-50% B in 15 min, 30 °C, 1 mL min^{-1}, C18 column, UV detection (215 nm).

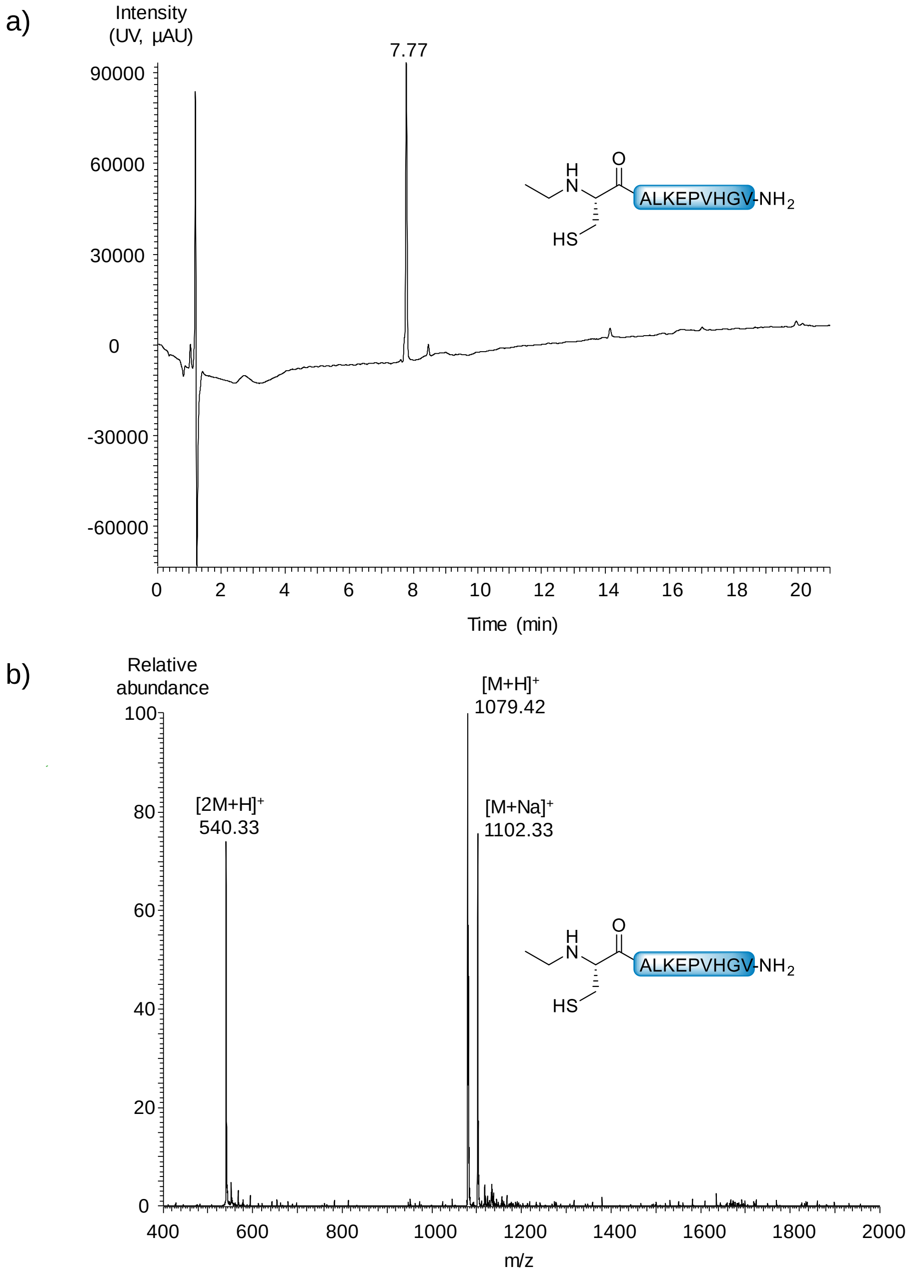

Supplementary Figure 72. UPLC-MS analysis of Et-CALKEPVHGV-NH_2. a) LC trace. Eluent A: 0.1% TFA in water, eluent B: 0.1% TFA in CH_3CN, ACQUITY UPLC Peptide BEH 300 Å 1.7 µm (2.1 × 150 mm) column, 50 °C, gradient 0-70% B in 20 min, 0.4 mL min^{-1}, UV detection at 215 nm. b) MS trace. Calcd. for $[M+H]^+$ (monoisotopic): 1079.60, found: 1079.42.

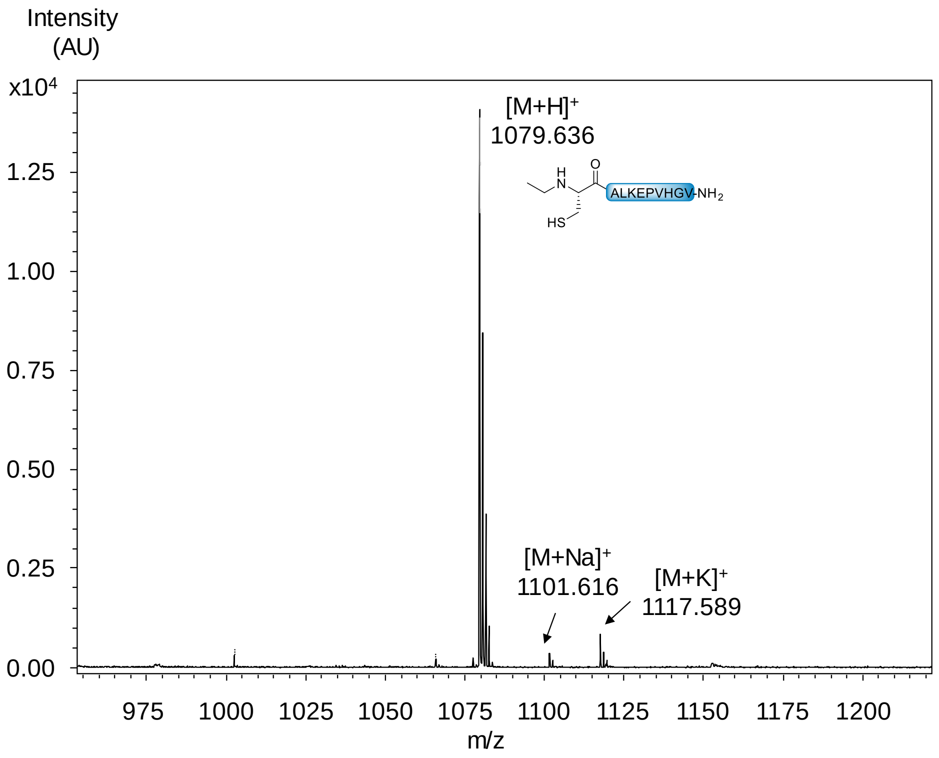

Supplementary Figure 73. MALDI-TOF analysis of Et-CALKEPVHGV-NH_2 peptide. Matrix α-cyano-4-hydroxycinnamic acid, positive detection mode, calcd. for $[M+H]^+$ (monoisotopic): 1079.60, found: 1079.64.

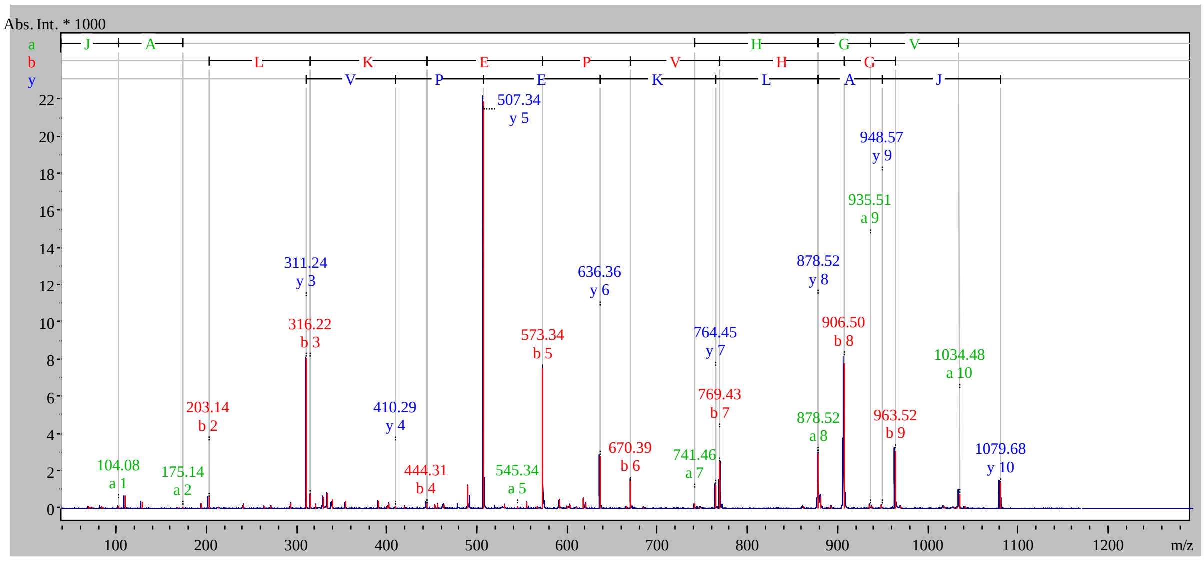

Supplementary Figure 74. MALDI-TOF-TOF sequencing of Et-CALKEPVHGV-NH_2 peptide. Matrix α-cyano-4-hydroxycinnamic acid, positive detection mode. The MS-MS analysis confirms that the SetCys residue is converted into a *N*-ethyl cysteine residue (J in the figure) in the peptide sequence.

Property 5

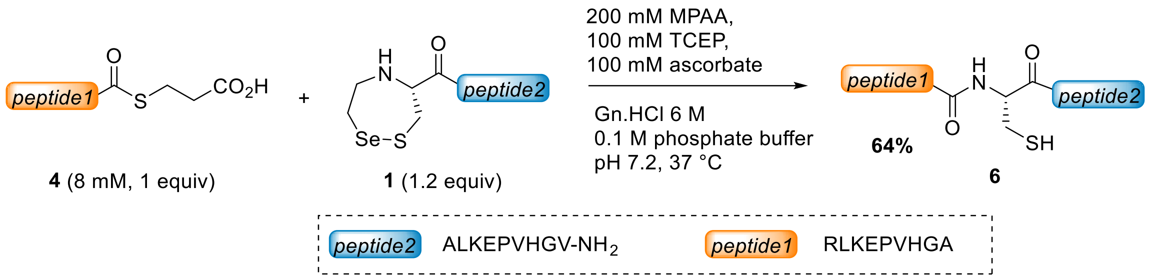

Supplementary Figure 75. Property 5. Model ligation of SetCys peptide **1** and peptidyl MPA-thioester **4** in the presence of TCEP.

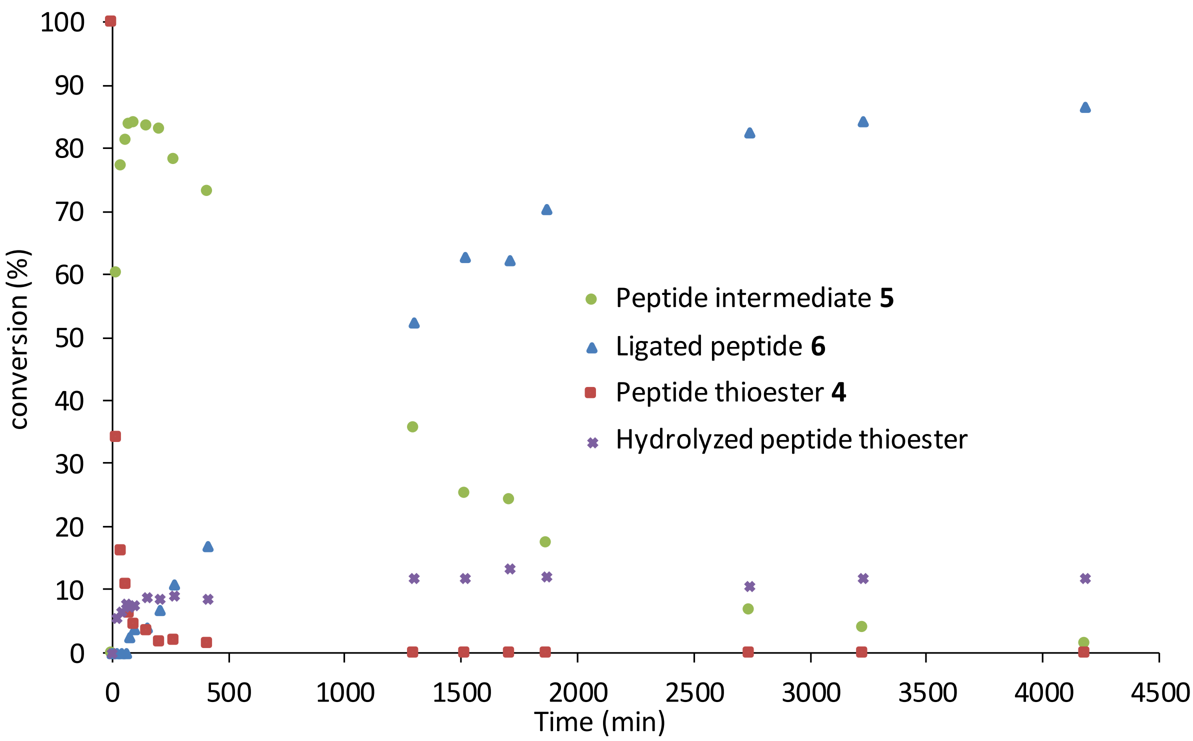

Supplementary Figure 76. HPLC monitoring of the reaction between SetCys peptide **1** and peptide thioester **4** performed in the presence of TCEP. For each point, a 2 µL aliquot was taken from the reaction mixture and quenched by adding 10% AcOH in water (100 µL). The sample was then extracted with Et_2O to remove MPAA prior to HPLC analysis. The proportion of each species in the mixture was determined by HPLC using UV detection at 215 nm.

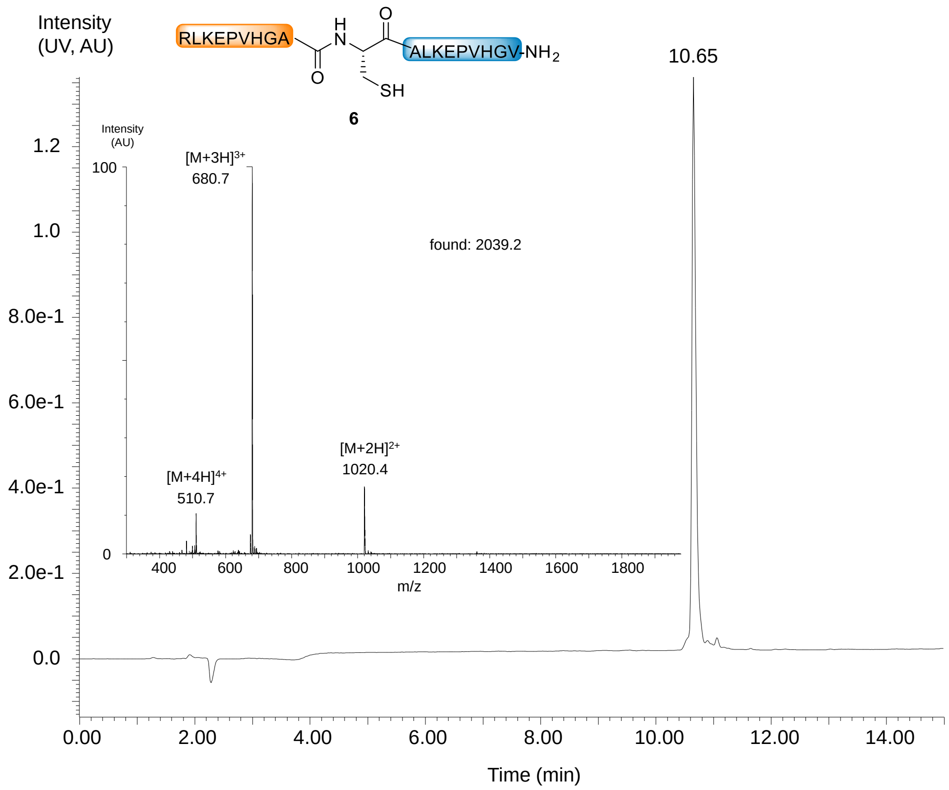

Supplementary Figure 77. LC-MS analysis of RLKEPVHGA-CALKEPVHGV-NH_2 peptide **6**. LC trace: eluent A 0.1% TFA in water, eluent B 0.1% TFA in CH_3CN/water 4/1 v/v, C18 Xbridge BEH 300 Å 5 µm (4.6 × 250 mm) column, gradient 0-50% B in 15 min, 30 °C, 1 mL min^{-1}, UV detection at 215 nm. MS trace: m/z = 1020.4 ($[M+2H]^{2+}$), 680.7 ($[M+3H]^{3+}$), 510.7 ($[M+4H]^{4+}$); calcd. for M (average): 2039.4, found 2039.2.

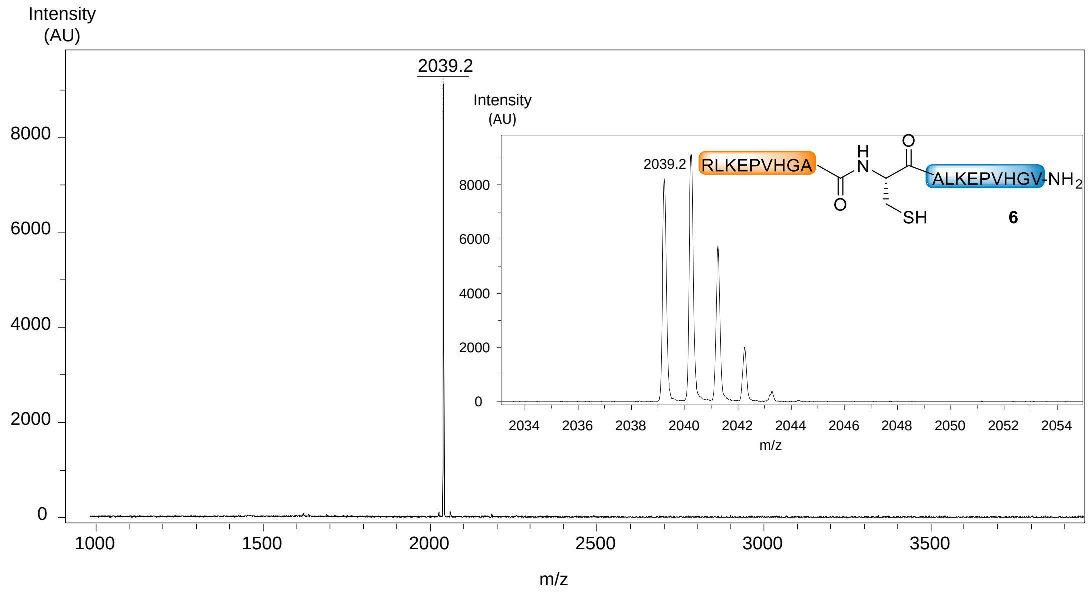

Supplementary Figure 78. MALDI-TOF analysis of RLKEPVHGA-CALKEPVHGV-NH_2 peptide **6**. Matrix α-cyano-4-hydroxycinnamic acid, positive detection mode, calcd. for $[M+H]^+$ (monoisotopic): 2039.1, found: 2039.2.

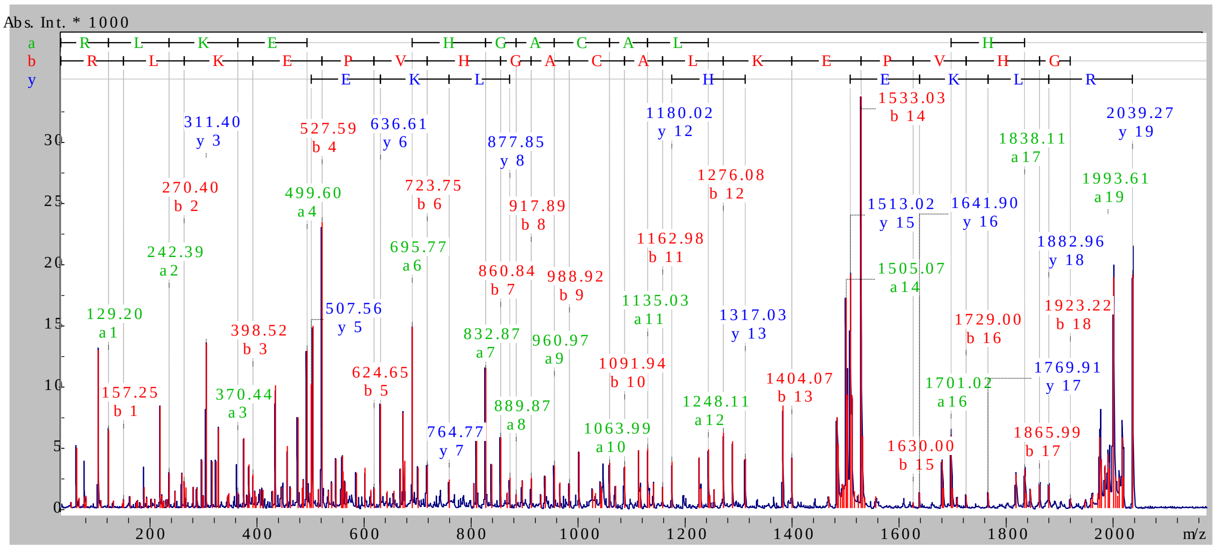

Supplementary Figure 79. MALDI-TOF-TOF sequencing of RLKEPVHGA-CALKEPVHGV-NH_2 peptide **6**. Matrix α-cyano-4-hydroxycinnamic acid, positive detection mode. The MS-MS analysis confirms the presence of Cys residue in the sequence of the peptide (in place of the SetCys amino acid).

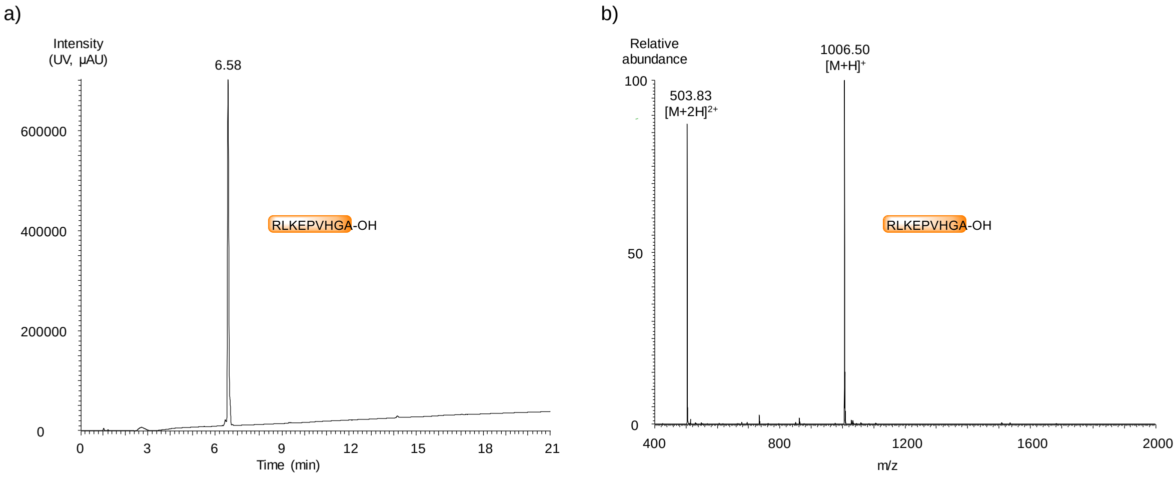

Supplementary Figure 80. LC-MS analysis of RLKEPVHGA-OH. a) LC trace. Eluent A 0.1% TFA in water, eluent B 0.1% TFA in CH_3CN, ACQUITY UPLC Peptide BEH 300Å 1.7 µm (2.1 × 150 mm) column, 50 °C, gradient 0-70% B in 20 min, 0.4 mL min^{-1}, UV detection at 215 nm. b) MS trace: calcd. for $[M+H]^+$ (monoisotopic): 1006.57, found: 1006.50.

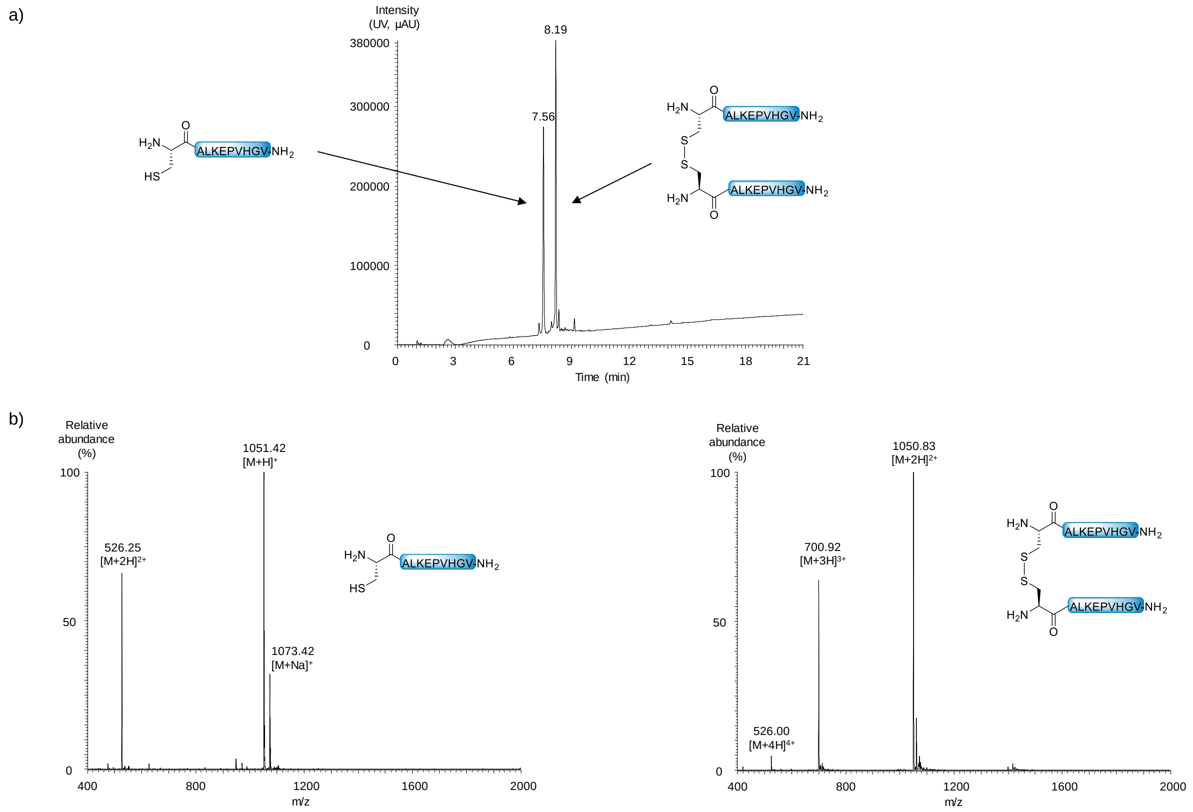

Supplementary Figure 81. LC-MS analysis of CALKEPVHGV-NH_2. The peptide was isolated as a mixture of monomer and oxidazed dimer. a) LC trace. Eluent A 0.1% TFA in water, eluent B 0.1% TFA in CH_3CN, ACQUITY UPLC Peptide BEH 300 Å 1.7 µm (2.1 × 150 mm) column, 50 °C, gradient 0-70% B in 20 min, 0.4 mL min^{-1}, UV detection at 215 nm. b) MS trace. For the monomer, calcd. for $[M+H]^+$ (monoisotopic): 1051.56, found: 1051.42. For the dimer, m/z = 1050.83 ($[M+2H]^{2+}$), 700.92 ($[M+3H]^{3+}$), 526.00 ($[M+4H]^{4+}$); calcd. for M (average): 2100.54, found: 2099.81.

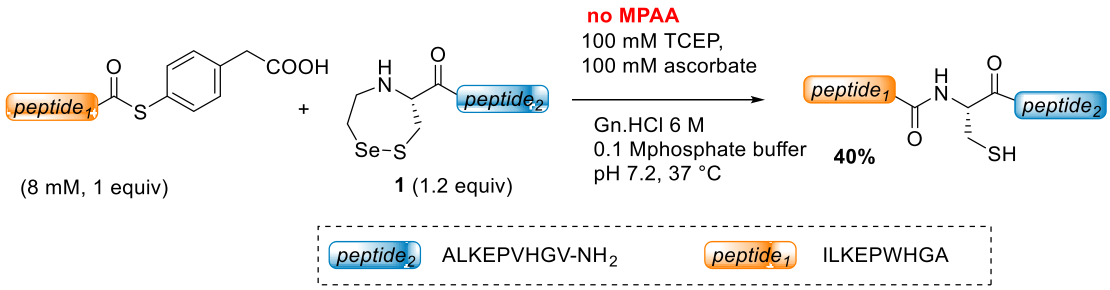

Supplementary Figure 82. Property 5. MPAA-free model ligation of SetCys peptide **1** and peptidyl MPAA-thioester ILKEPWHGA-MPAA in the presence of TCEP.

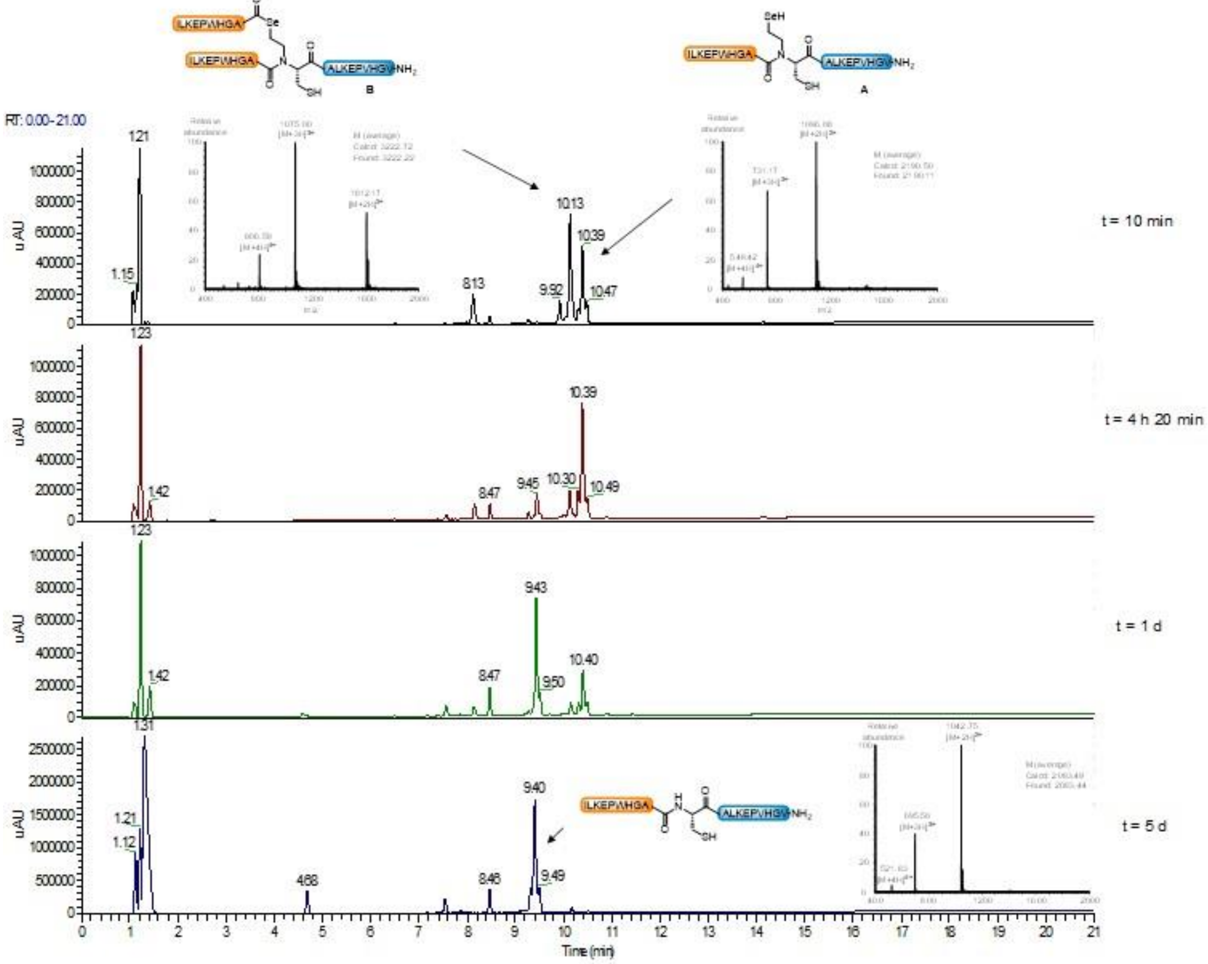

Supplementary Figure 83. LC-MS monitoring of the reaction between SetCys peptide **1** and peptidyl MPAA-thioester ILKEPWHGA-MPAA performed in the presence of TCEP.

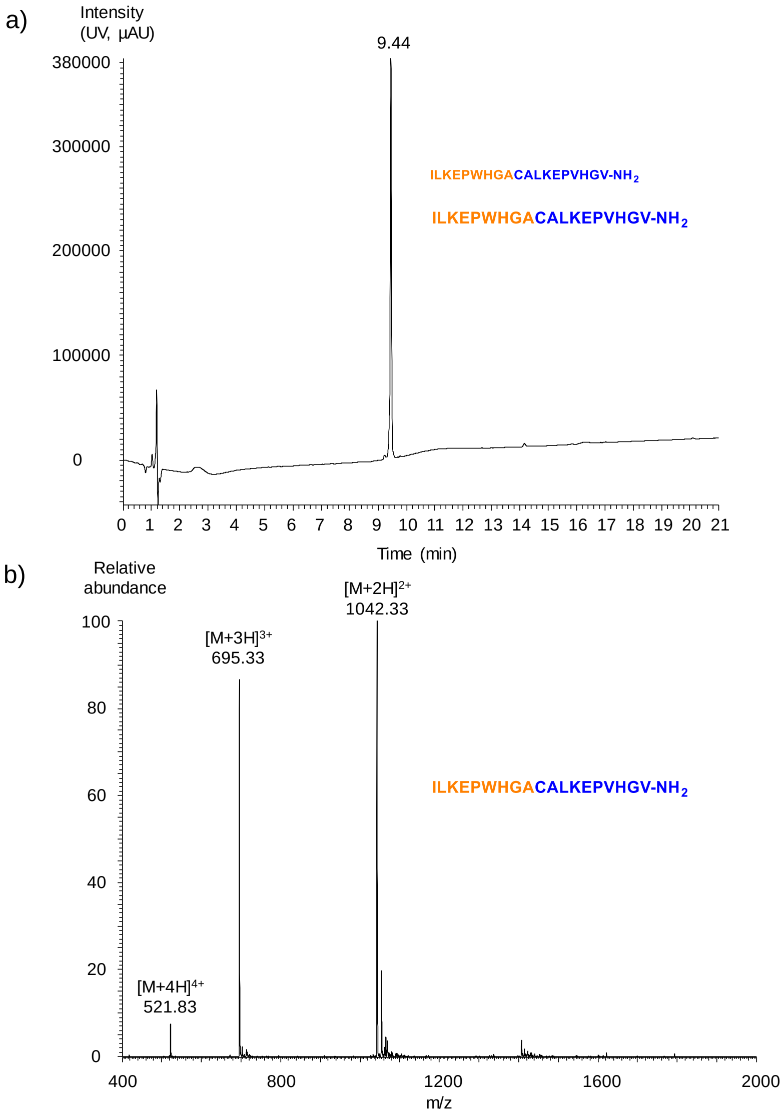

Supplementary Figure 84. LC-MS analysis of ILKEPWHGACALKEPVHGV-NH_2. a) LC trace. Eluent A 0.1% TFA in water, eluent B 0.1% TFA in CH_3CN, ACQUITY UPLC Peptide BEH 300 Å 1.7 µm (2.1 × 150 mm) column, 50 °C, gradient 0-70% B in 20 min, 0.4 mL min^{-1}, UV detection at 215 nm. b) MS trace: m/z = 1853.42 ($[M+2H]^{2+}$), 927.58 ($[M+3H]^{3+}$), 618.83 ($[M+4H]^{4+}$). Calcd. for M (average): 2083.49, found: 2082.99.

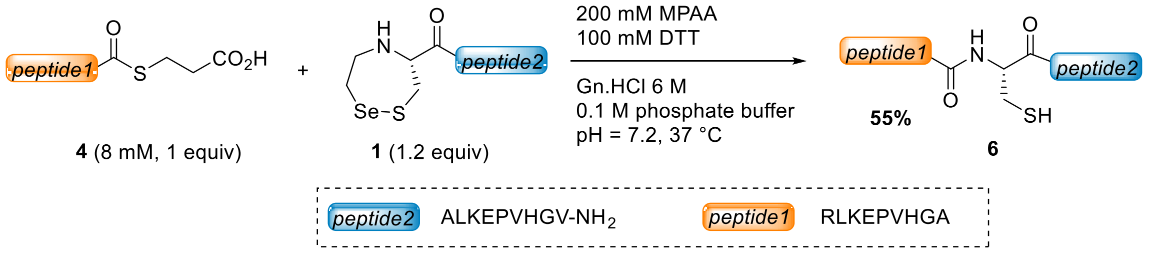

Supplementary Figure 85. Ligation of SetCys peptide **1** and peptide thioester **4** in the presence of DTT.

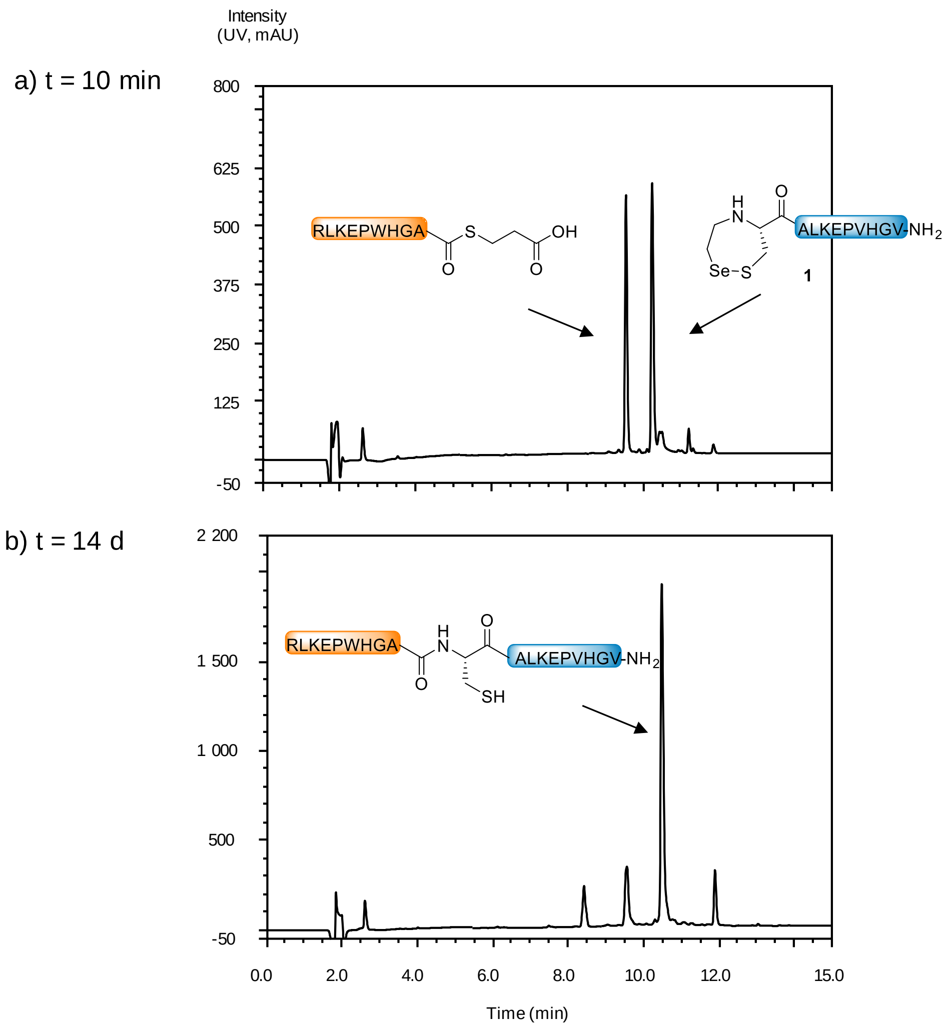

Supplementary Figure 86. LC-MS monitoring of the reaction between SetCys peptide **1** and peptide thioester **4** performed in the presence of DTT. a) LC trace after 10 min. b) LC trace after 14 days. Eluent A 0.1% TFA in water, eluent B 0.1% TFA in CH_3CN/water 4/1 v/v. C18 Xbridge BEH 300 Å 5 µm (4.6 × 250 mm) column, gradient 0-50% B in 15 min, 30 °C, 1 mL min^{-1}, UV detection at 215 nm.

Reactivity studies (Figure 2b)

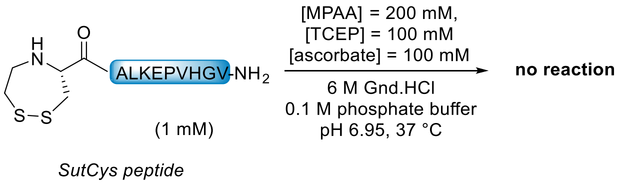

Supplementary Figure 87. Stability of the **SutCys-ALKEPVHGV-NH2** peptide under strong reductive conditions.

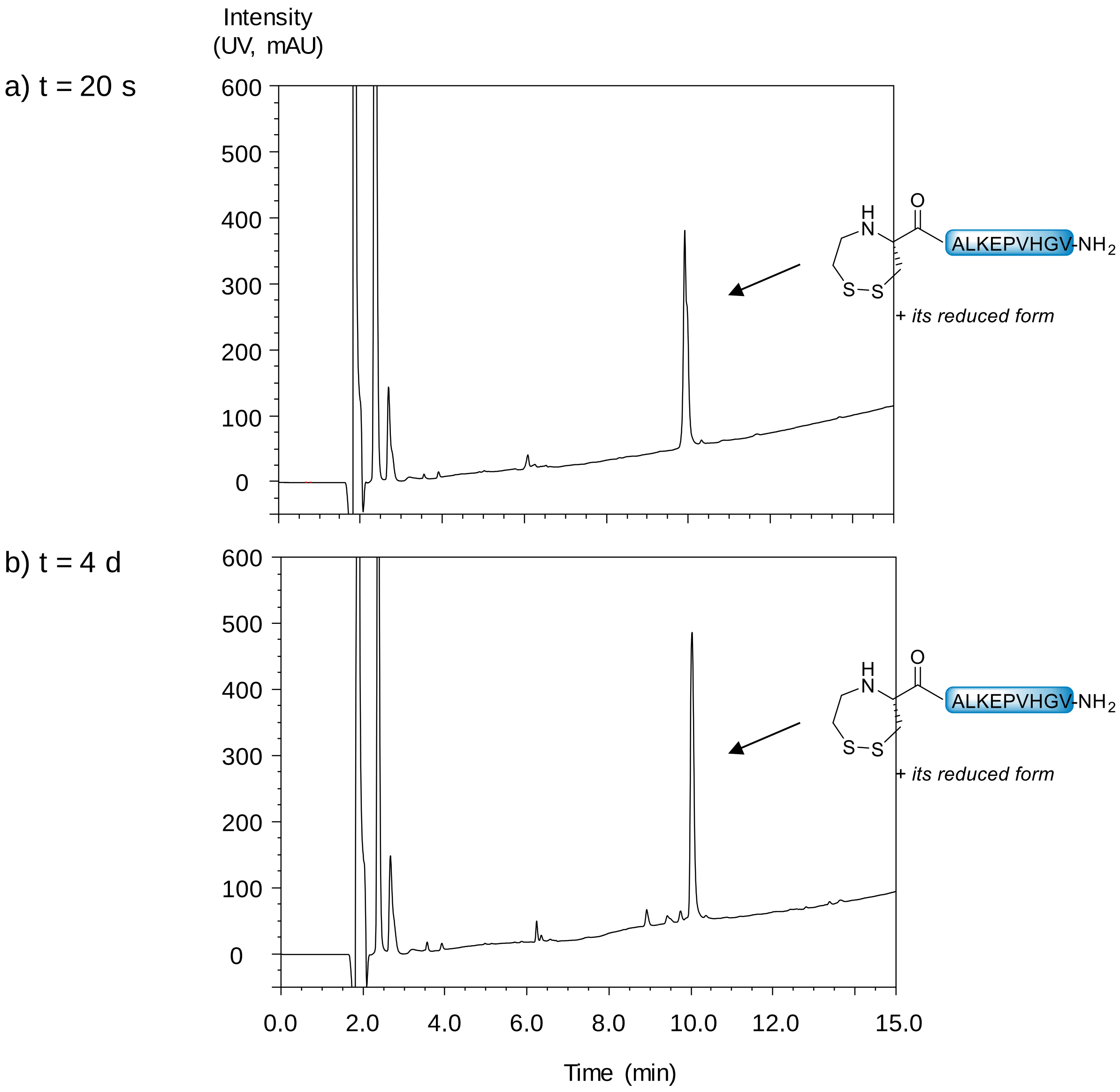

Supplementary Figure 88. Stability of the SutCys peptide in the presence of MPAA + TCEP (pH = 6.06). a) LC trace after 20 s. b) LC trace after 4 days. Eluent A 0.1% TFA in water, eluent B 0.1% TFA in CH_3CN/water 4/1 v/v, C18 column, gradient 0-50% B in 15 min, 30 °C, 1 mL min^{-1}, UV detection at 215 nm.

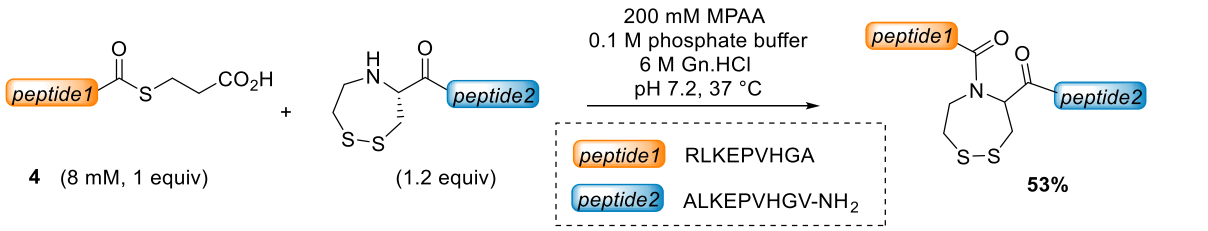

Supplementary Figure 89. Ligation reaction of SutCys-ALKEPVHGV-NH_2 peptide and peptidyl MPA-thioester **4** under low reducing conditions.

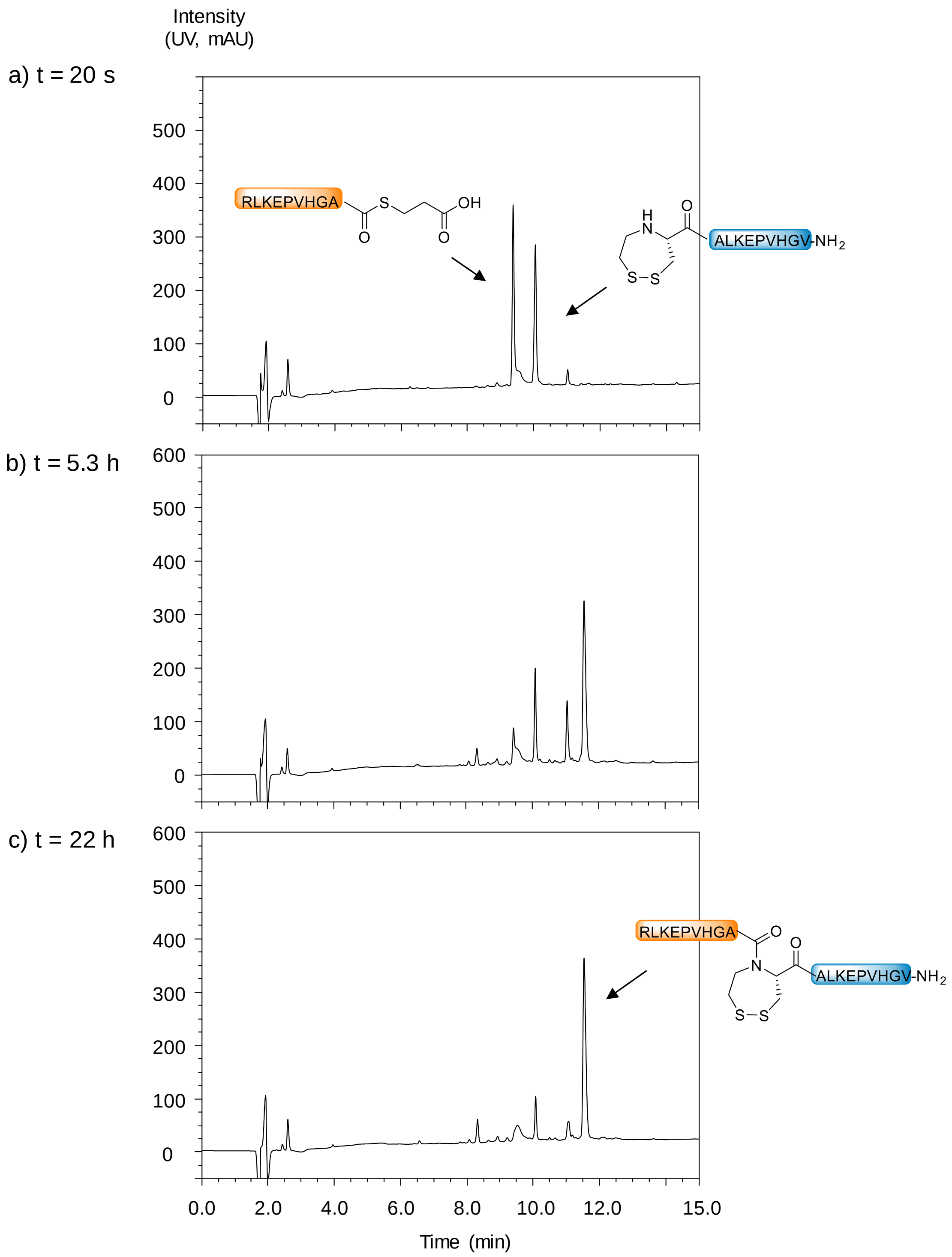

Supplementary Figure 90. Monitoring of the ligation between SutCys peptide SutCysALKEPVHGV-NH_2 and peptidyl MPA thioester **4** under low reducing conditions. a) LC trace after 20 s. b) LC trace after 5.3 h. c) LC trace after 22 h. Eluent A 0.1% TFA in water, eluent B 0.1% TFA in CH_3CN/water 4/1 v/v, C18 column, gradient 0-50% B in 15 min, 30 °C, 1 mL min^{-1}, UV detection at 215 nm.

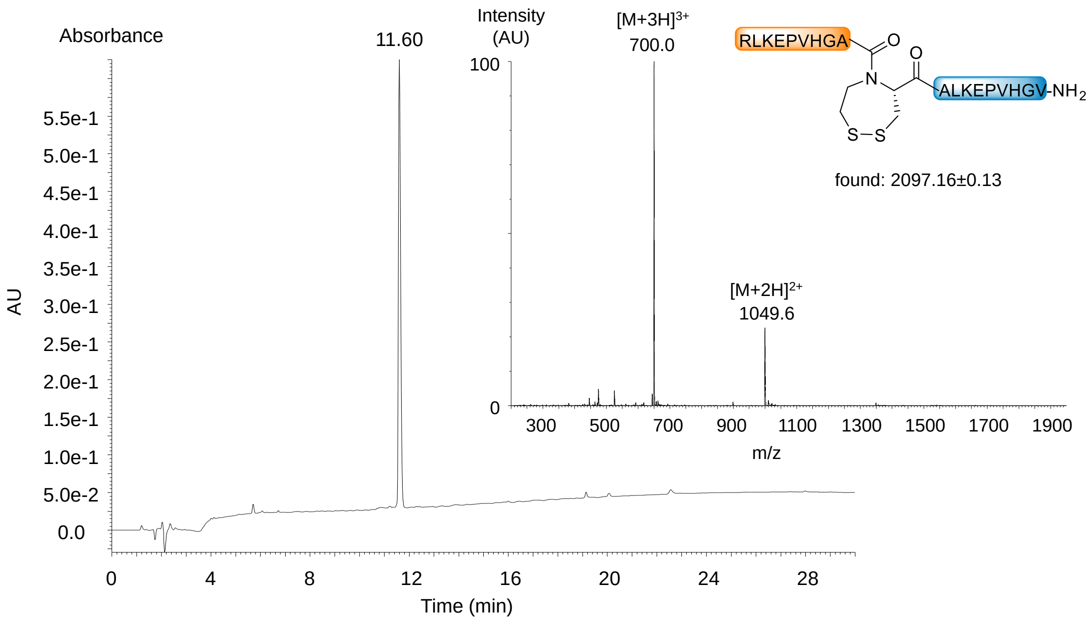

Supplementary Figure 91. LC-MS analysis of RLKEPVHGA-SutCys-ALKEPVHGV-NH_2 peptide. LC trace: eluent A 0.1% TFA in water, eluent B 0.1% TFA in CH_3CN/water 4/1 v/v, C18 Xbridge BEH 300 Å 5 μm (4.6 × 250 mm) column, gradient 0-100% B in 30 min, 50 °C, 1 mL min^{-1}, UV detection at 215 nm. MS trace: 1049.6 ($[M+2H]^{2+}$), 700.0 ($[M+3H]^{3+}$); calcd. for M (average): 2097.5, found: 2097.2.

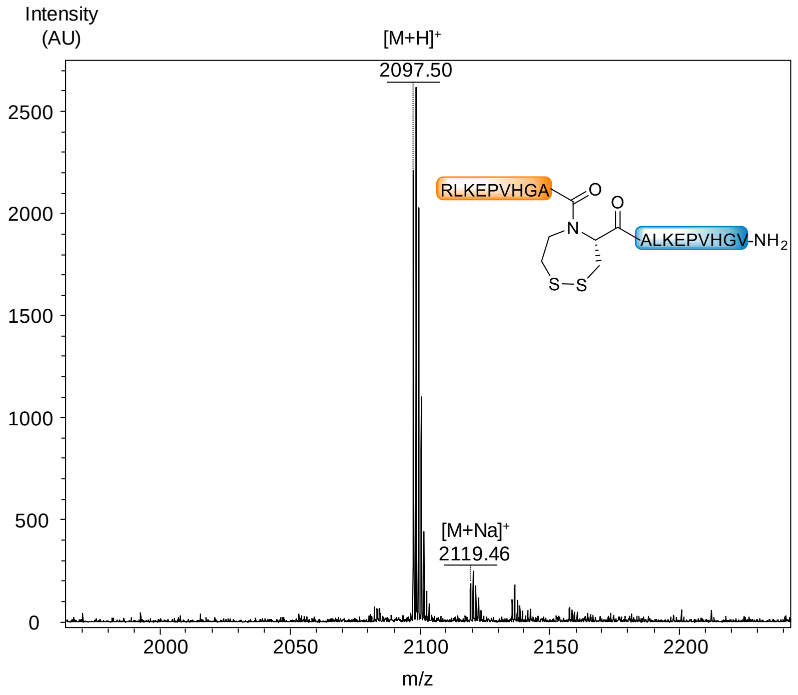

Supplementary Figure 92. MALDI-TOF analysis of RLKEPVHGA-SutCys-ALKEPVHGV-NH_2 peptide. Matrix α-cyano-4-hydroxycinnamic acid, positive detection mode, calcd. for $[M+H]^+$ (monoisotopic): 2097.1, found: 2097.5.

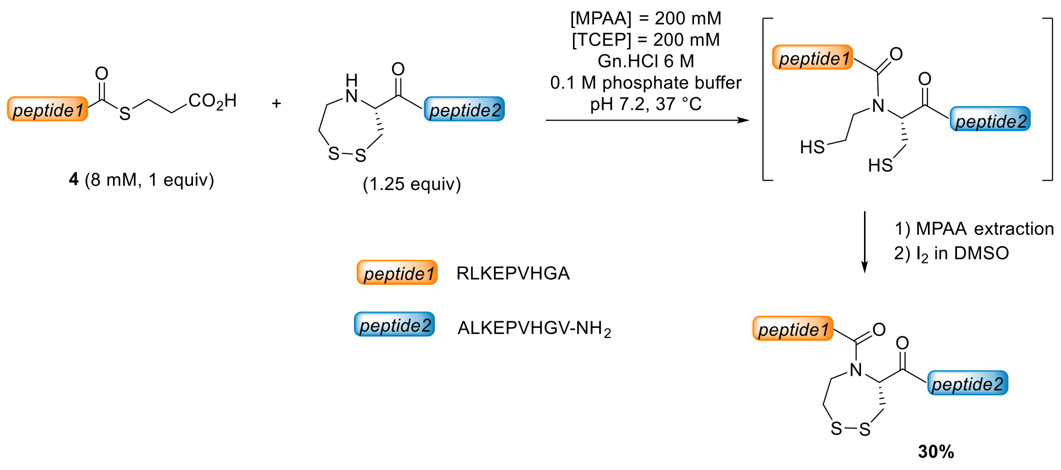

Supplementary Figure 93: Ligation reaction of SutCys-ALKEPVHGV-NH_2 peptide with peptidyl MPA-thioester **4** under strong reducing conditions.

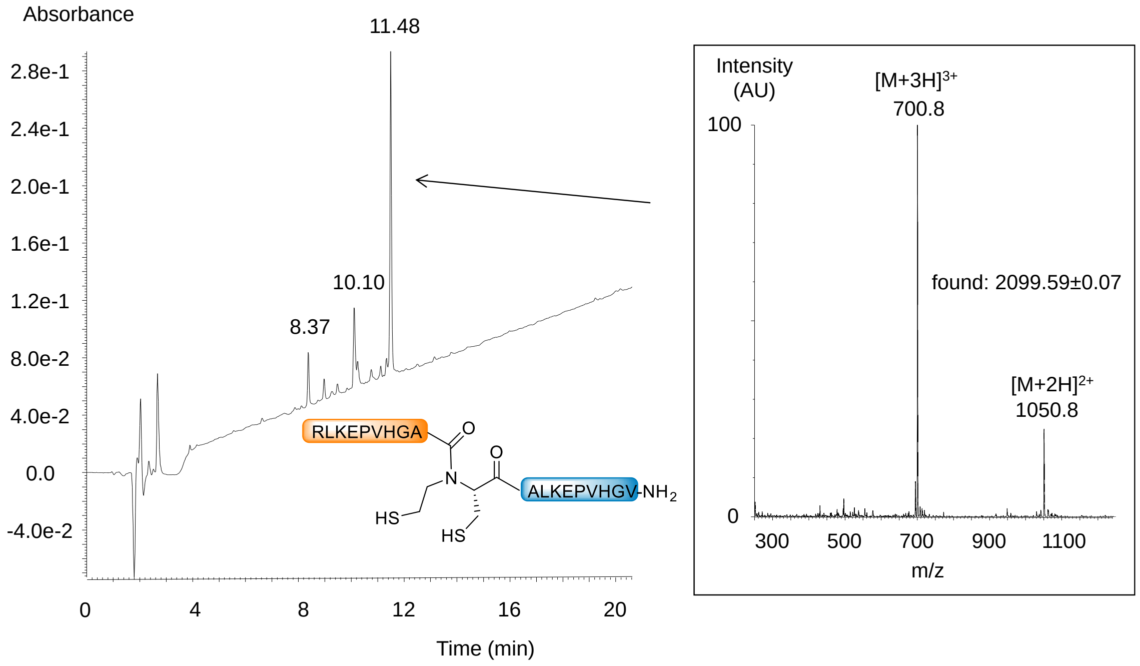

Supplementary Figure 94. LC-MS monitoring of the ligation between SutCys-ALKEPVHGV-NH_2 peptide and peptidyl MPA thioester **4** performed under strong reducing conditions. LC trace: eluent A 0.1% TFA in water, eluent B 0.1% TFA in CH_3CN/water 4/1 v/v, C18 Xbridge BEH 300 Å 5 µm (4.6 × 250 mm) column, gradient 0-100% B in 30 min, 50 °C, 1 mL min^{-1}, UV detection at 215 nm. MS trace for SutCys ligated peptide (t_R = 11.48 min): 1050.8 ($[M+2H]^{2+}$), 700.8 ($[M+3H]^{3+}$); calcd. for M (average): 2099.5, found: 2099.6.

Mechanistic studies

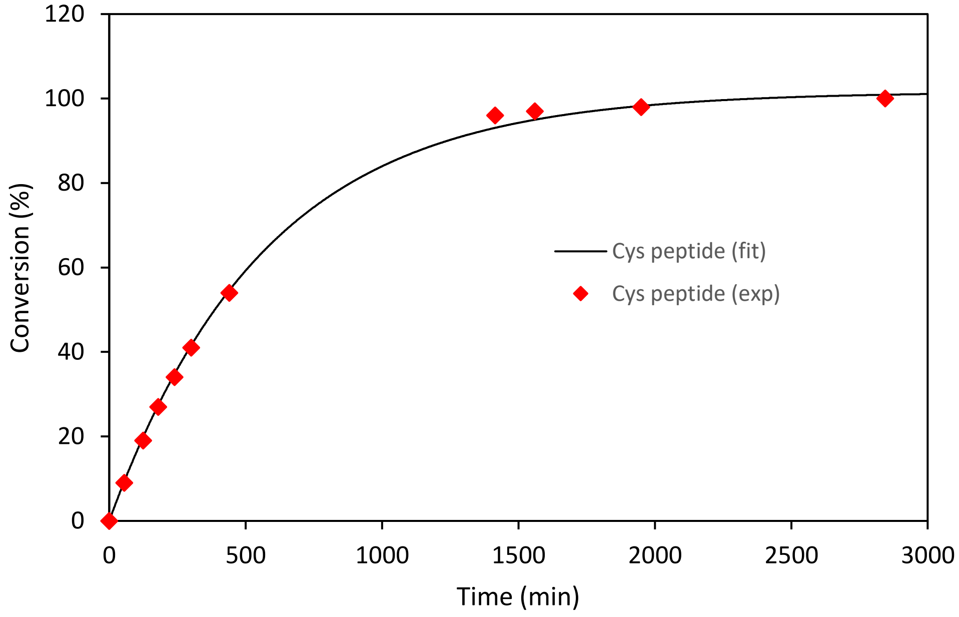

Supplementary Figure 95. Fitting of the reaction leading to a loss of the *N*-selenoethyl appendage on SetCys peptides at pH 6.90 using KinteK Explorer Software*TM* (Version 8.0.190823., Kintek Corporation - https://kintekcorp.com/software/). $k_{SetCys\ 6.90}$ = 0.00172 ± 0.00066 min^{-1}. Red diamonds correspond to experimental values / Gray line corresponds to fitted data.

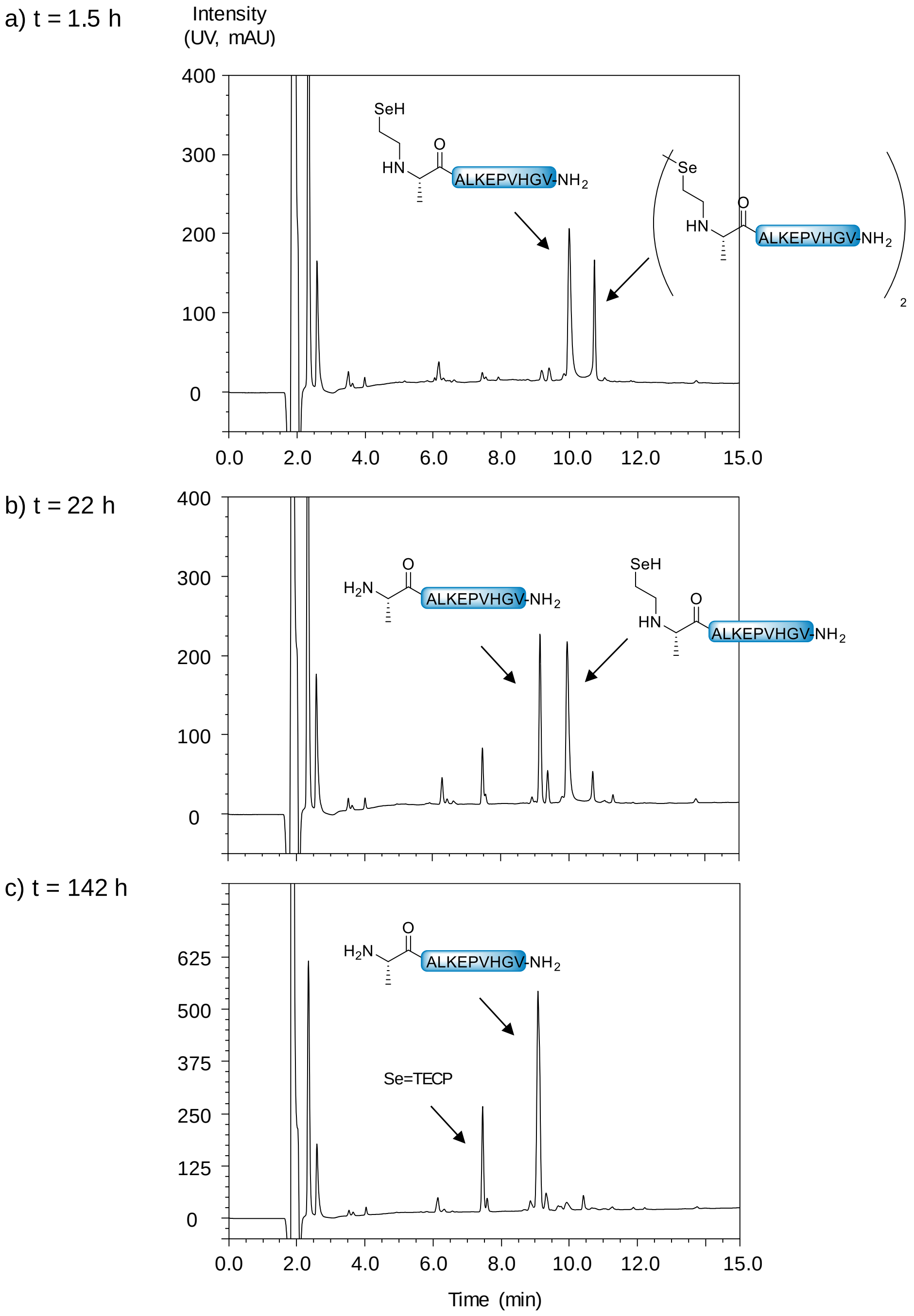

Supplementary Figure 96. Monitoring of the conversion of SetAla peptide **1** into Ala peptide using MPAA + TCEP. a) LC trace after 1.5 h. b) LC trace after 22 h. c) LC trace after 142 h. LC trace: eluent A 0.1% TFA in water, eluent B 0.1% TFA in CH_3CN, C18 column, gradient 0-50% B in 15 min, 30 °C, 1 mL min^{-1}, UV detection at 215 nm.

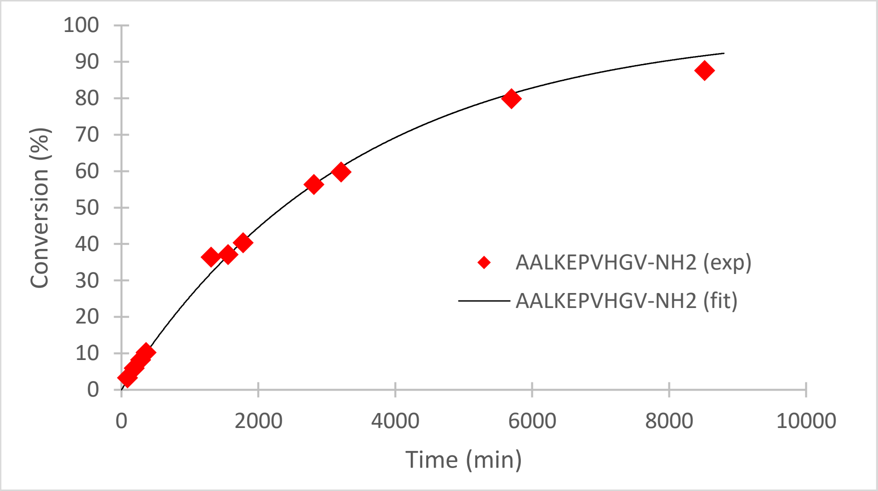

Supplementary Figure 97. Fitting of the reaction leading to a loss of the *N*-selenoethyl appendage on SetAla peptides at pH 5.97 using KinteK Explorer Software™ (Version 8.0.190823., Kintek Corporation - https://kintekcorp.com/software/). k_{SetAla} = 0.000296 ± 0.000091 min^{-1}. Red diamonds correspond to experimental values / Black line corresponds to fitted data.

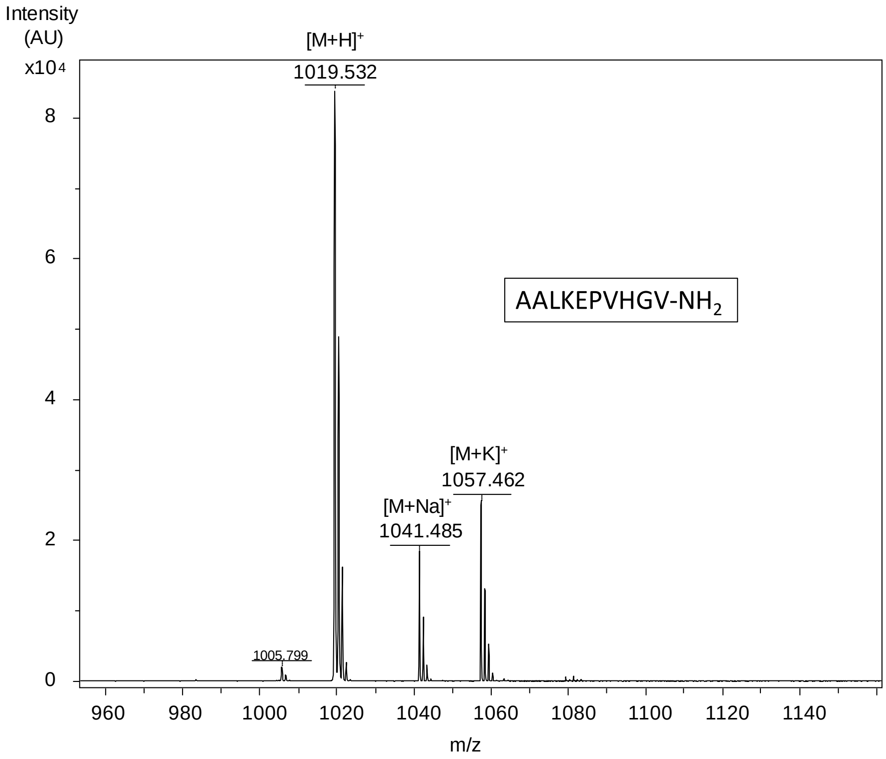

Supplementary Figure 98. MALDI-TOF analysis of AALKEPVHGV-NH_2 peptide. Matrix α-cyano-4-hydroxycinnamic acid, positive detection mode, calcd. for $[M+H]^+$ (monoisotopic): 1019.60, found: 1019.53.

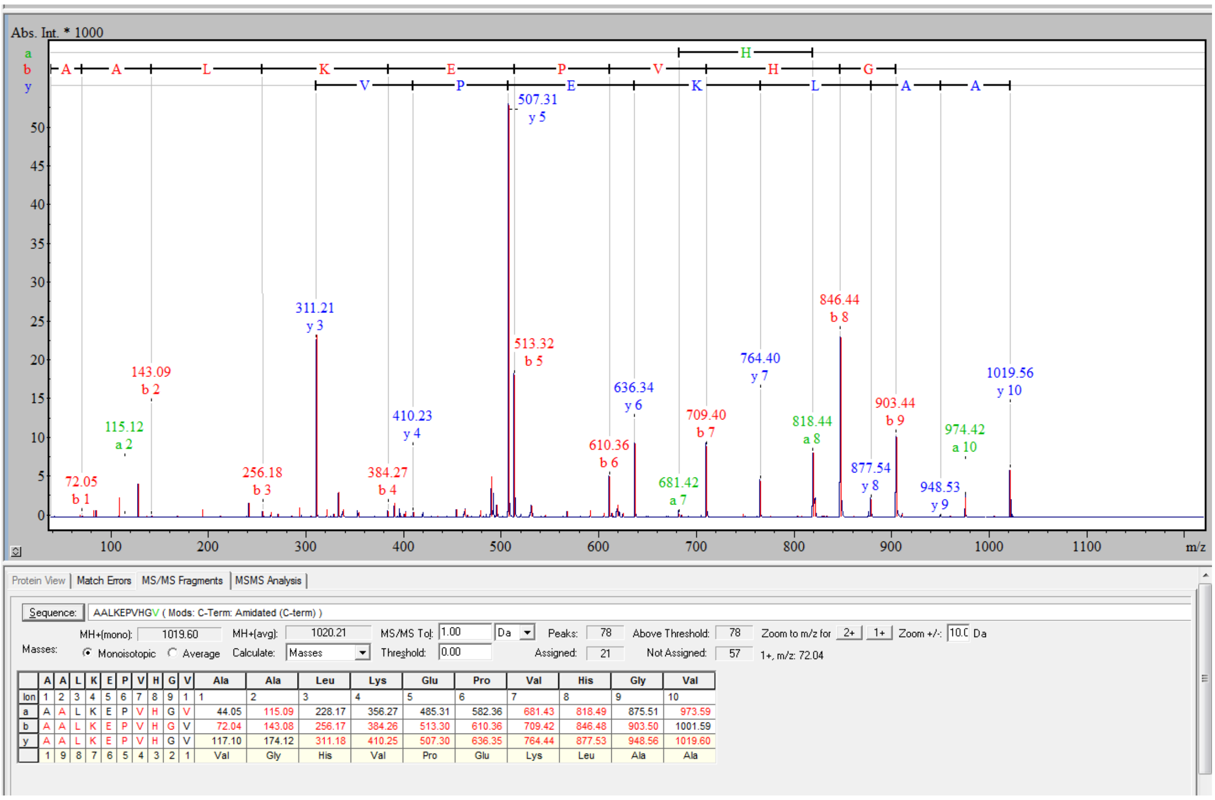

Supplementary Figure 99. MALDI-TOF-TOF sequencing of AALKEPVHGV-NH_2 peptide. Matrix α-cyano-4-hydroxycinnamic acid, positive detection mode. The MS-MS analysis confirms that the SetAla residue is converted into the Ala amino acid in the peptide.

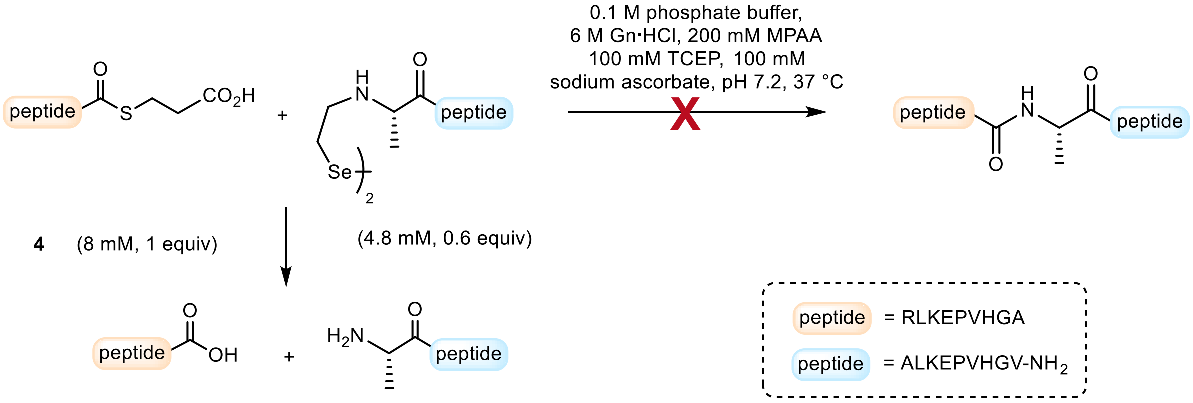

Supplementary Figure 100. Ligation reaction of SetAla peptide with peptidyl MPA thioester **4**.

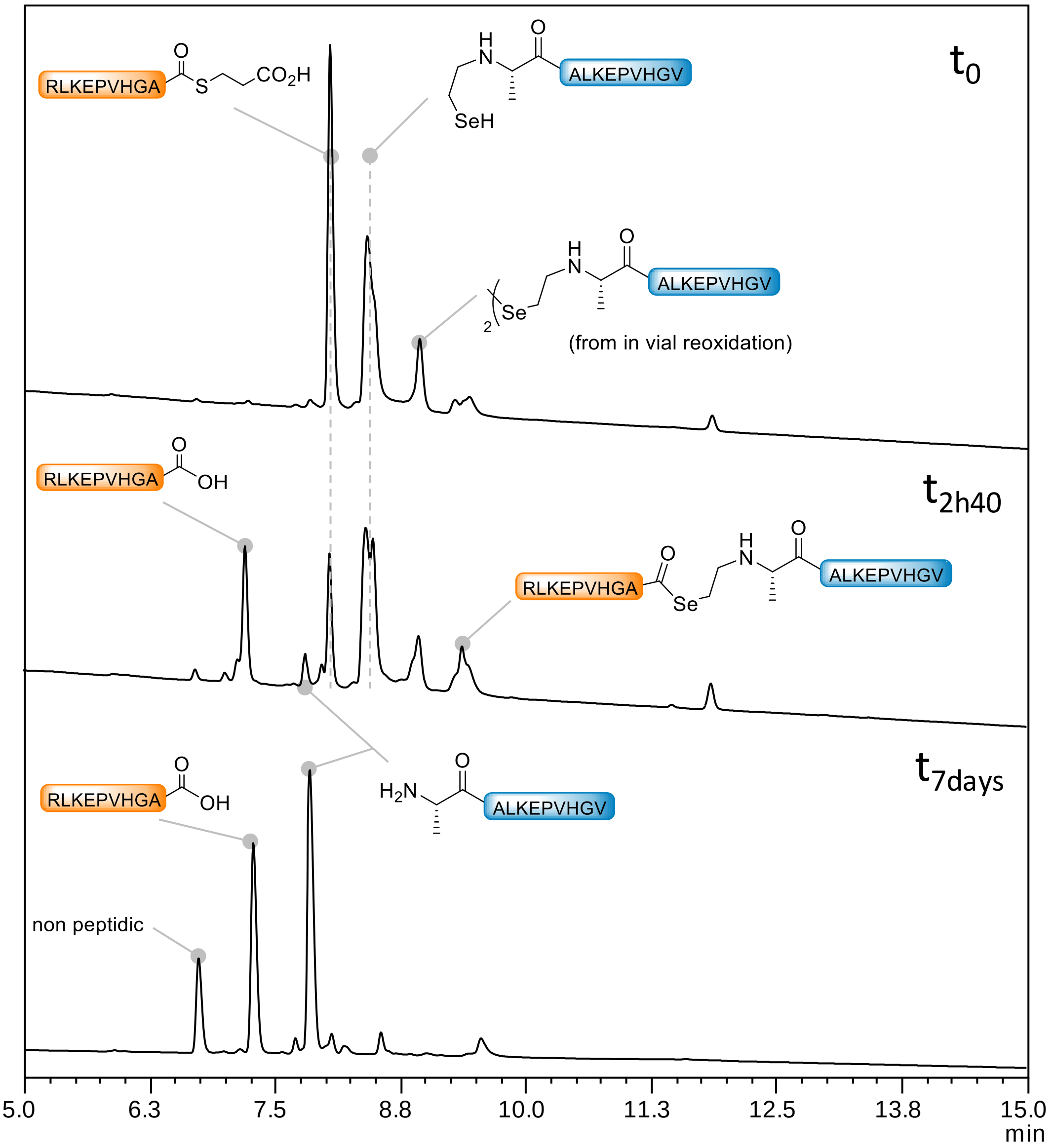

Supplementary Figure 101. Monitoring of the reaction between SetAla peptide and peptidyl MPA thioester **4** in the presence of MPAA, TCEP and sodium ascorbate. LC trace: eluent A 0.1% TFA in water, eluent B 0.1% TFA in CH_3CN, C18 column, gradient 0-50% B in 15 min, 30 °C, 1 mL min^{-1}, UV detection at 215 nm.

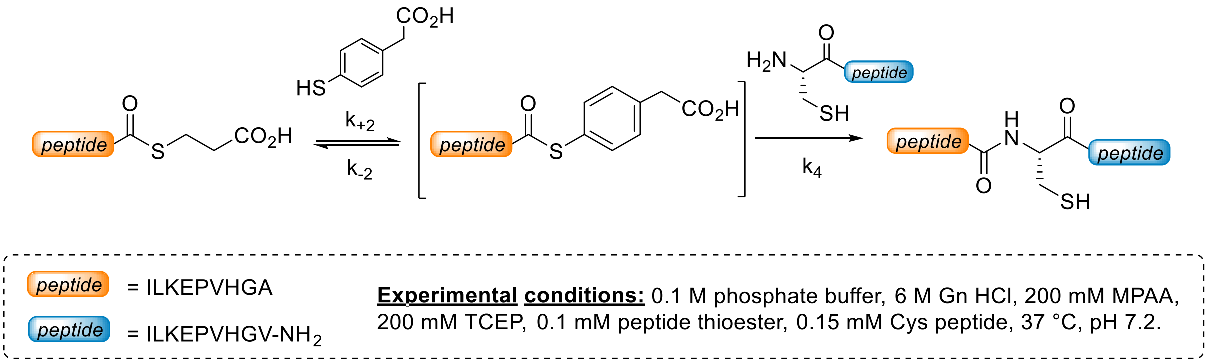

Supplementary Figure 102. Kinetic study of the model NCL reaction at submillimolar peptide concentration.

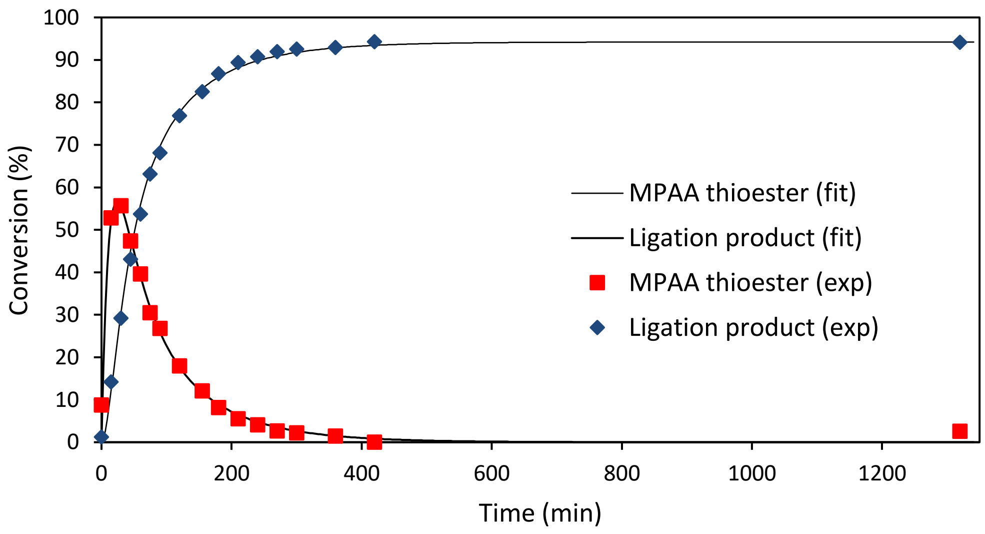

Supplementary Figure 103. Fitting of the model NCL reaction at submillimolar peptide concentration using KinteK Explorer Software^{TM} (Version 8.0.190823., Kintek Corporation - https://kintekcorp.com/software/). https://kintekcorp.com/software/). Experimental conditions: 0.1 M phosphate buffer, 6 M Gn·HCl, 200 mM MPAA, 200 mM TCEP, 0.1 mM peptide thioester, 0.15 mM Cys peptide, 37 °C, pH 7.2. Red squares / Blue diamonds correspond to experimental values.

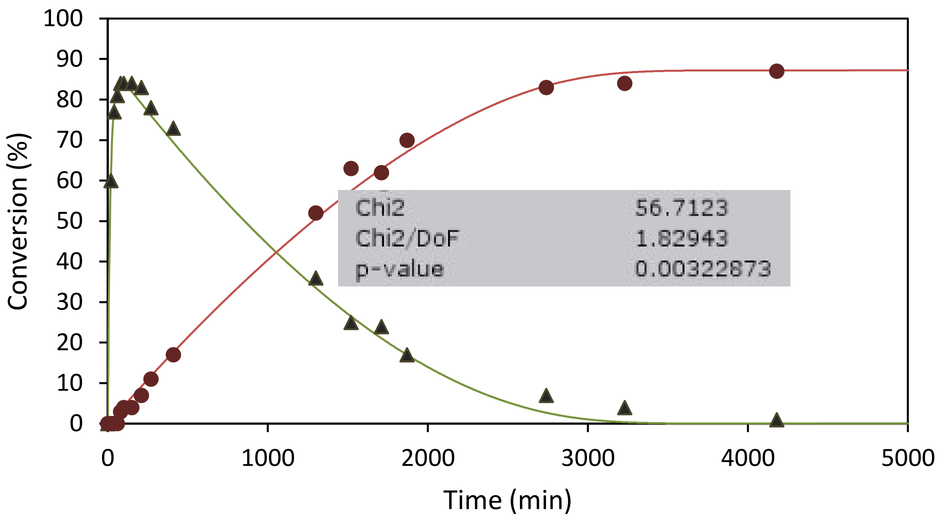

Supplementary Figure 104. Ligation of SetCys peptide **1** with MPA thioester **4** in NCL standard conditions. RP-HPLC monitoring of the formation of peptides **5** (black triangles) and **6** (brown circles) throughout the course of the reaction. The continuous lines correspond to the fitting of experimental data using KinteK Explorer Software*TM* (Version 8.0.190823., Kintek Corporation - https://kintekcorp.com/software/). *p*-value was obtained from a Pearson's chi-squared test (one-sided).

Peptide and protein assemblies

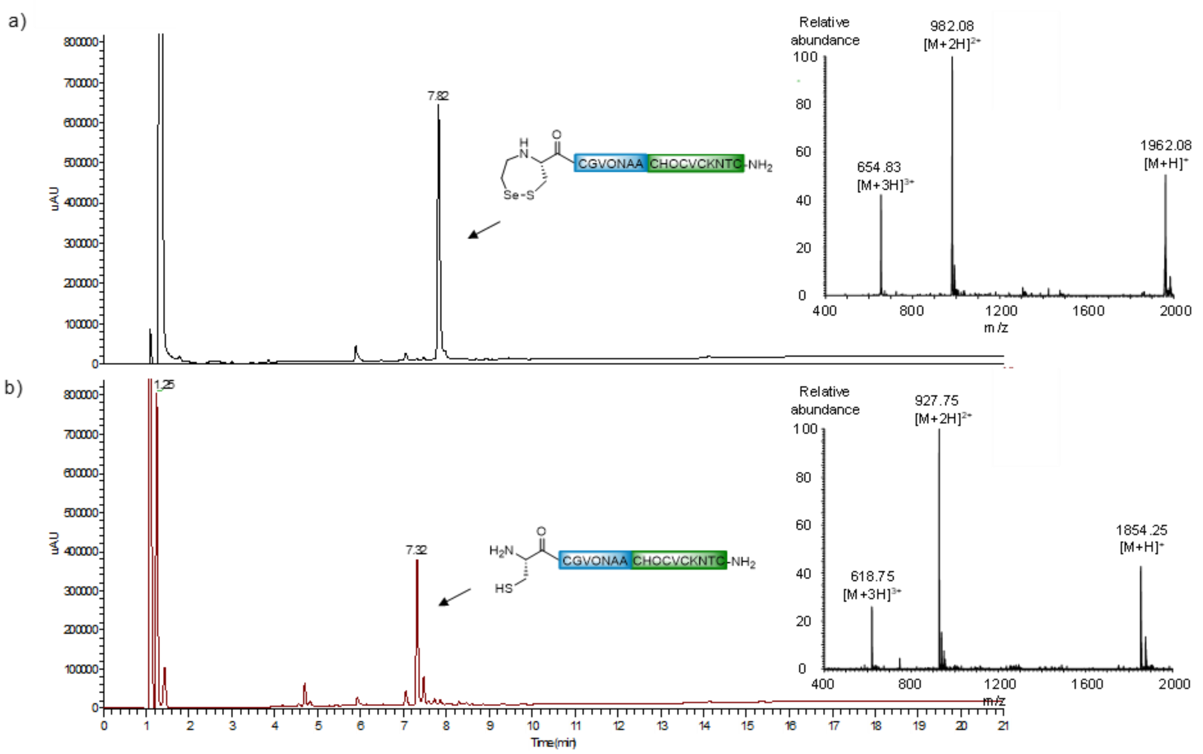

Supplementary Figure 105. Conotoxin OIVA assembly monitored by UPLC-MS. a) Analysis of the reaction mixture 2 h after mixing the two partners of the ligation reaction. The elongated SetCys peptide is detected in mixture with its reduced form, due to the addition of TCEP in the analyzed sample (to reduce MPAA adducts). b) Analysis of the reaction mixture 24 h after the addition of TCEP. LC trace: eluent A 0.1% TFA in water, eluent B 0.1% TFA in CH_3CN, ACQUITY UPLC Peptide BEH 300 Å 1.7 µm (2.1 × 150 mm) column, 50 °C, gradient 0-70% B in 20 min, 0.4 mL min^{-1}, UV detection.

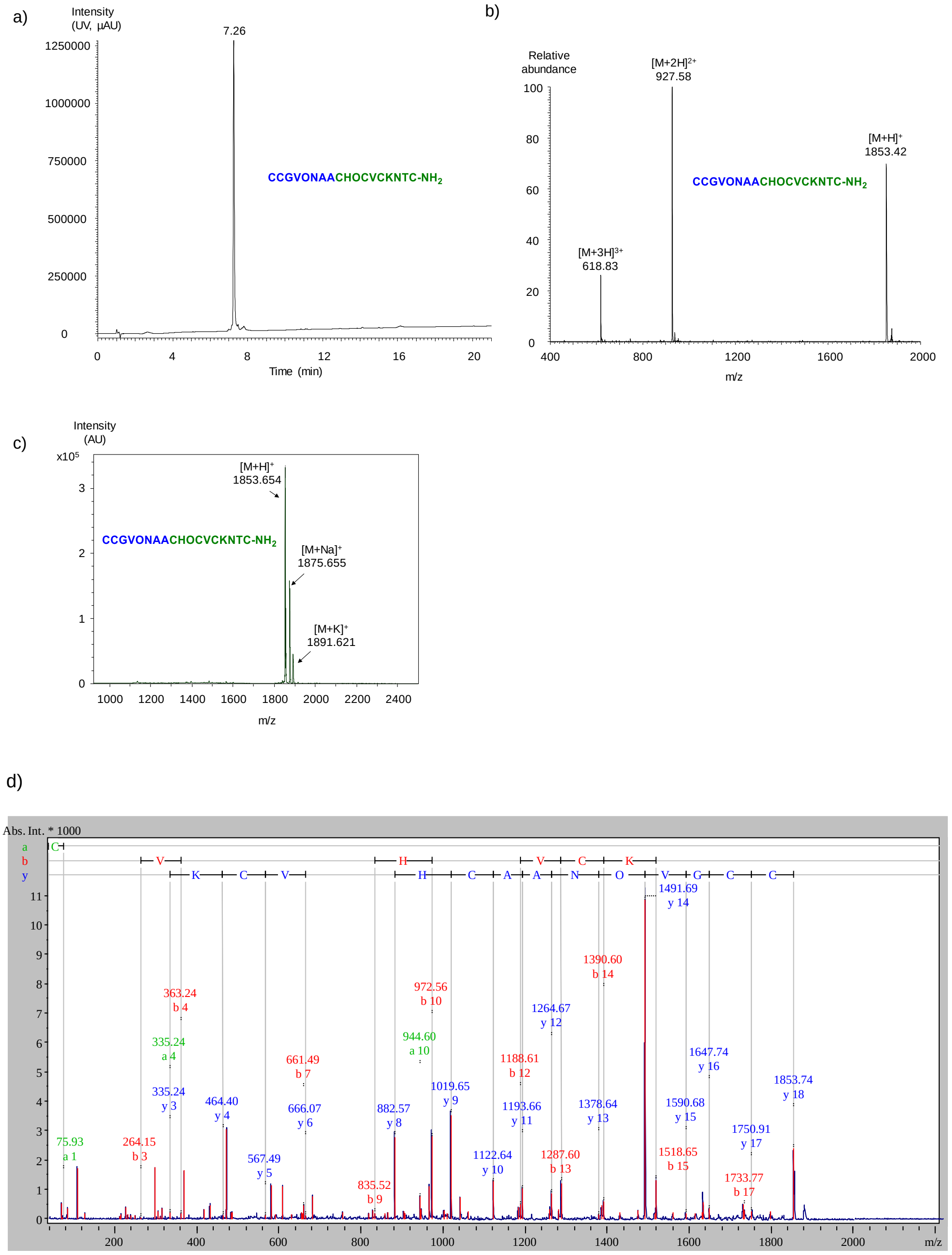

Supplementary Figure 106. Analysis of linear conotoxin OIVA. a) LC trace. Eluent A 0.1% TFA in water, eluent B 0.1% TFA in CH_3CN, ACQUITY UPLC Peptide BEH 300 Å 1.7 µm (2.1 × 150 mm) column, 50 °C, gradient 0-70% B in 20 min, 0.4 mL min^{-1}, UV detection. b) MS trace: Calcd. for $[M+H]^+$ (monoisotopic): 1853.70, found: 1853.42. c) MALDI-TOF analysis of conotoxin OIVA linear peptide. Matrix α-cyano-4-hydroxycinnamic acid, positive detection mode, calcd. for $[M+H]^+$ (monoisotopic): 1853.70, found: 1853.65. d) MALDI-TOF-TOF sequencing of conotoxin OIVA linear peptide. Matrix α-cyano-4-hydroxycinnamic acid, positive detection mode.

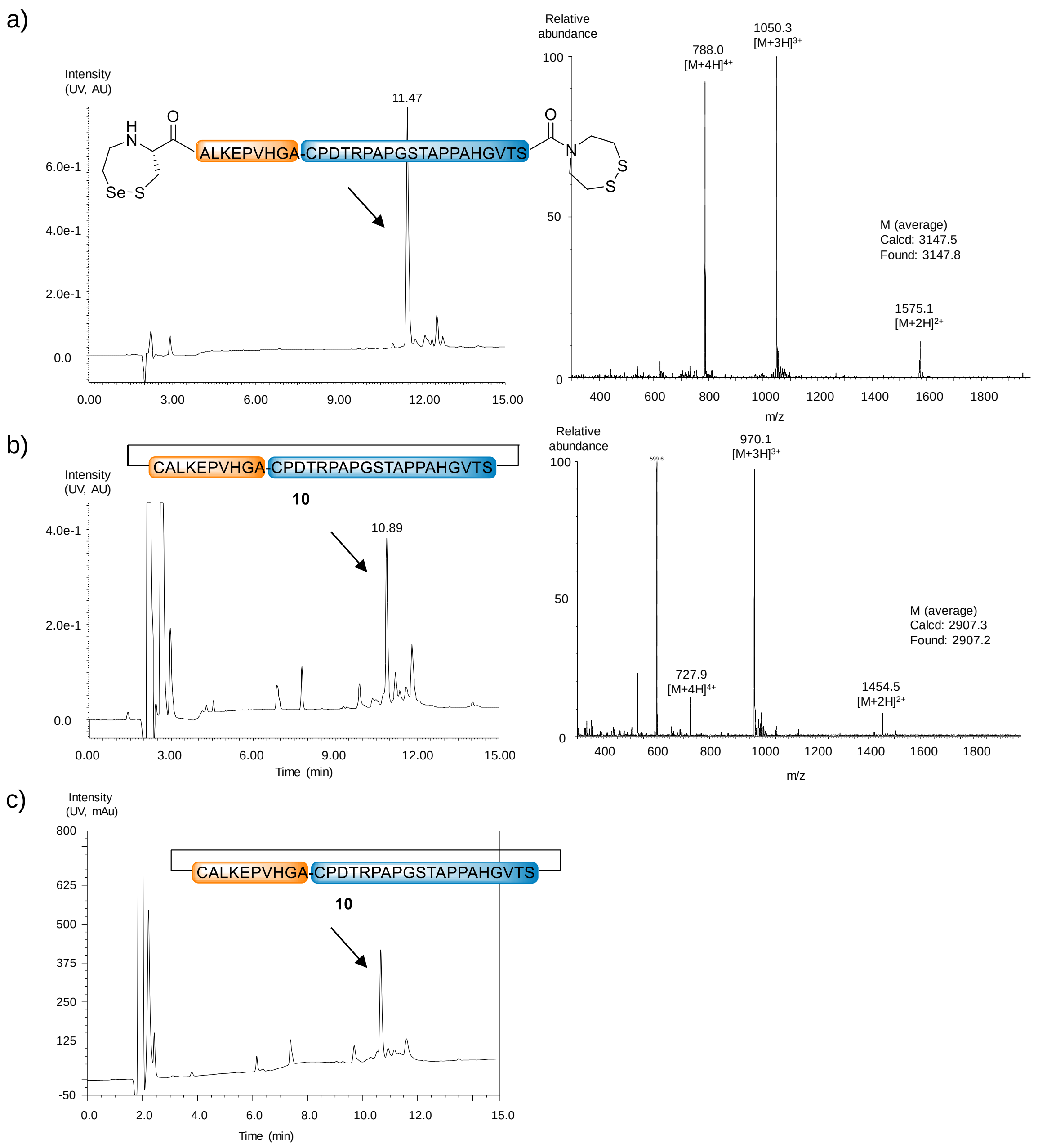

Supplementary Figure 107. Assembly of the cyclic peptide **10** monitored by LC-MS (for a and b) and HPLC (for c). a) Analysis of the reaction mixture 3 h after mixing the two partners of the ligation reaction. b) Analysis of the reaction mixture 16 h after the addition of TCEP. c) Analysis of the reaction mixture 22 h after the addition of TCEP. LC trace: eluent A 0.1% TFA in water, eluent B 0.1% TFA in CH_3CN/water 4/1 v/v, C18 column, gradient 0-50% B in 15 min, 50 °C (for a and b) or 30 °C (for c), 1 mL min^{-1}, UV detection at 215 nm.

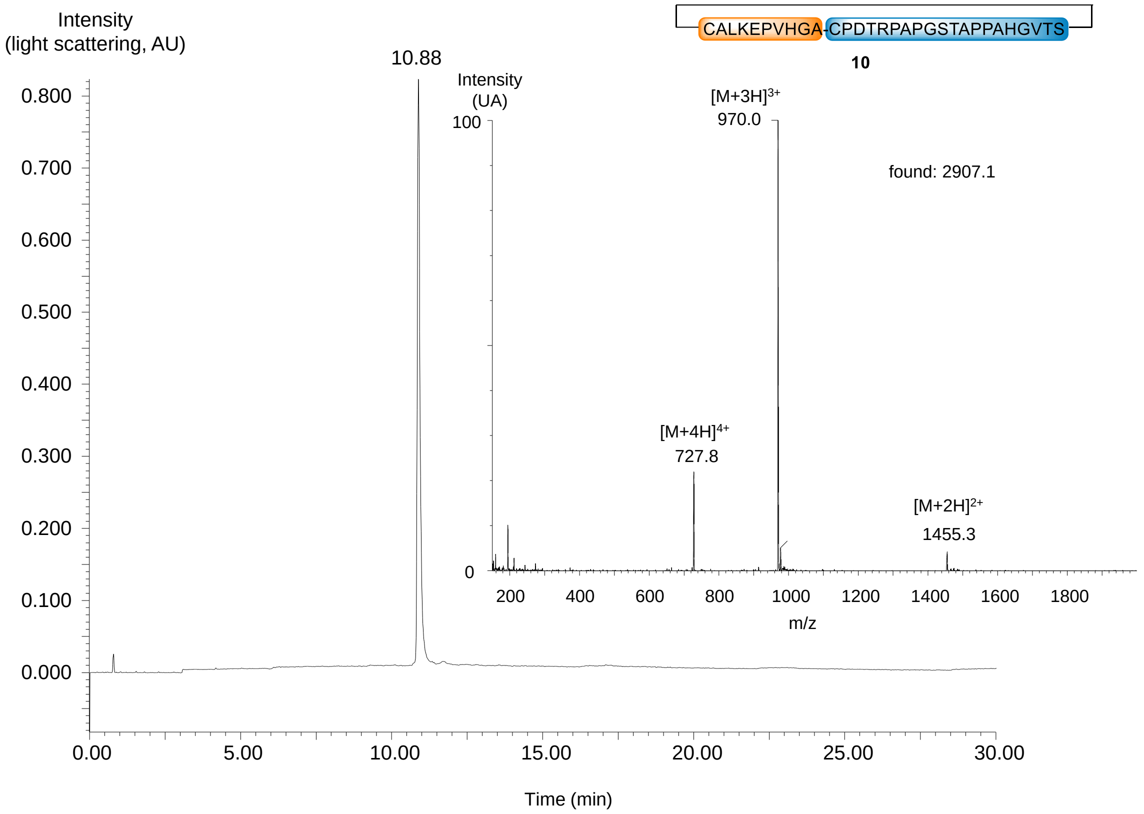

Supplementary Figure 108. LC-MS analysis of purified cyclic peptide **10**. LC trace: eluent A 0.1% TFA in water, eluent B 0.1% TFA in CH_3CN/water 4/1 v/v, C18 column, gradient 0-100% B in 30 min, 50 °C, 1 mL min^{-1}, light scattering detection. MS trace: m/z 1455.3 ($[M+2H]^{2+}$), 970.0 ($[M+3H]^{3+}$), 727.8 ($[M+4H]^{+}$); calcd. for M (average): 2907.3, found: 2907.1.

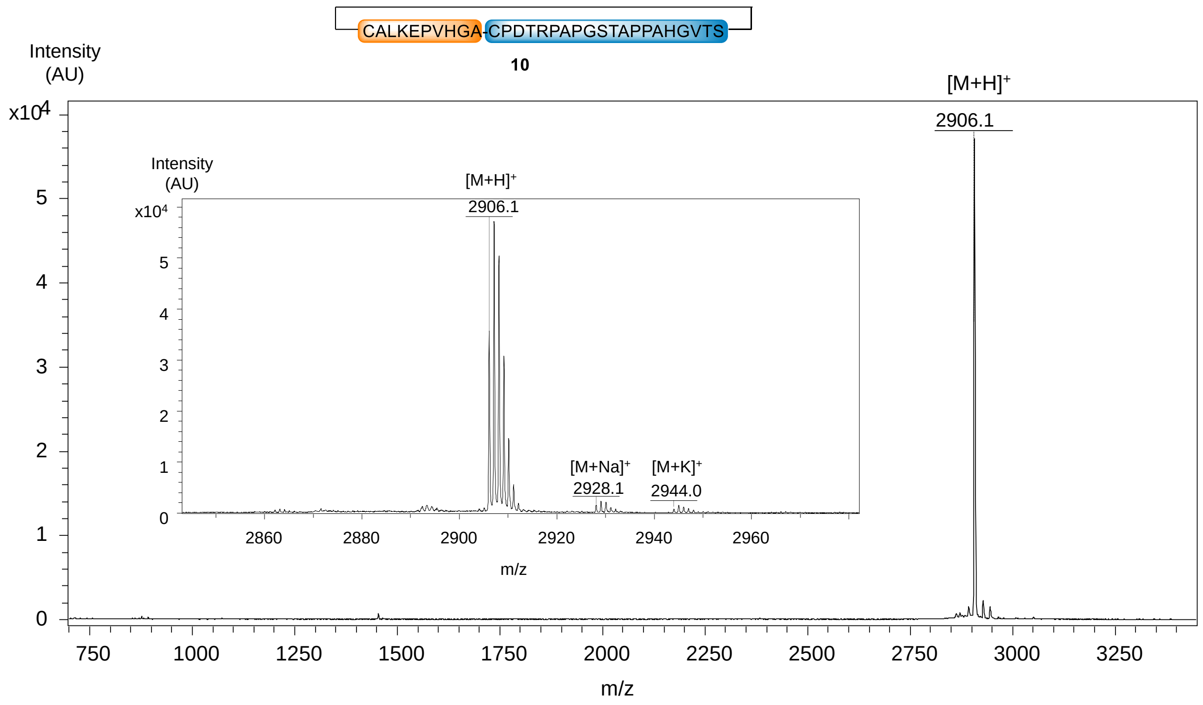

Supplementary Figure 109. MALDI-TOF analysis of cyclic peptide **10**. Matrix α-cyano-4-hydroxycinnamic acid, positive detection mode, calcd. for $[M+H]^+$ (monoisotopic): 2906.4, found: 2906.1.

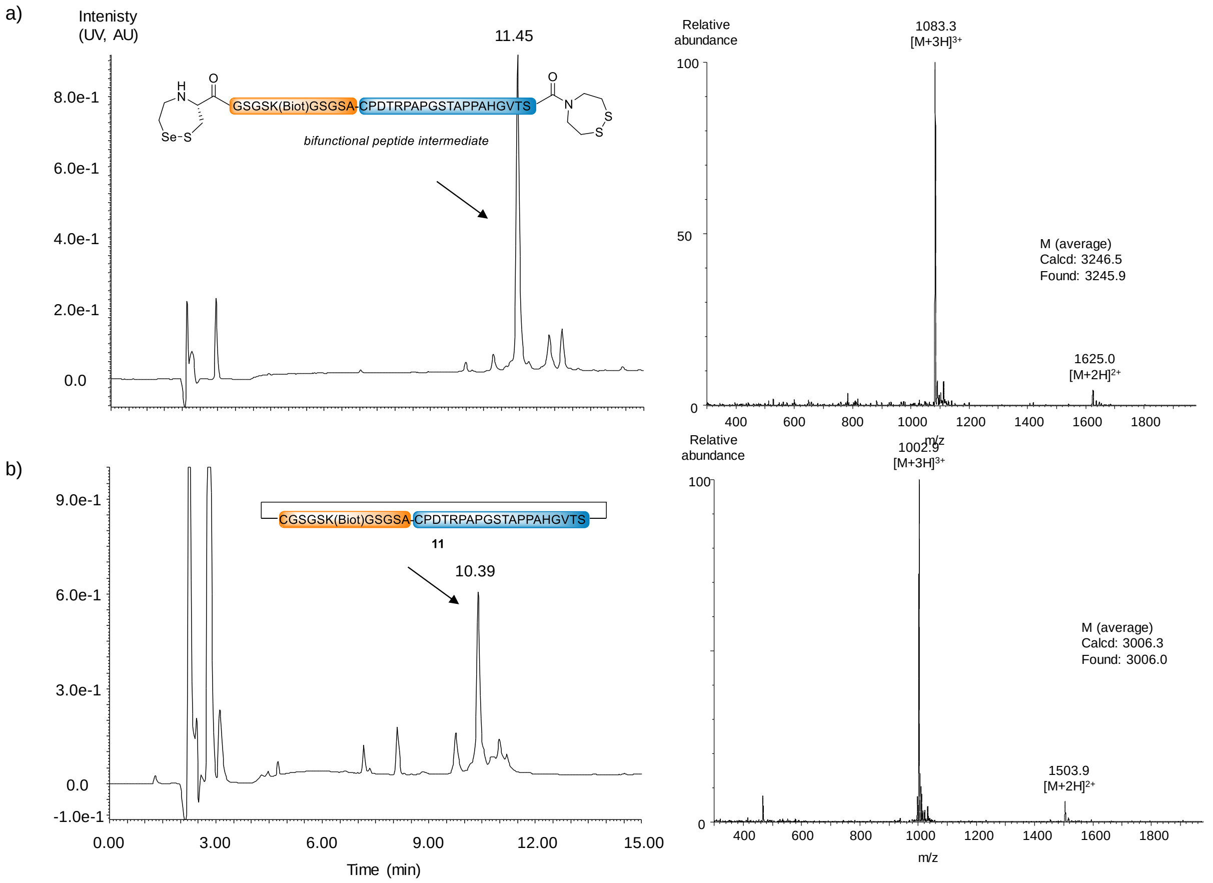

Supplementary Figure 110. Assembly of the cyclic peptide **11** monitored by LC-MS. a) Analysis of the reaction mixture 3.5 h after mixing the two partners of the ligation reaction. b) Analysis of the reaction mixture 4 d after the dilution with TCEP solution. LC trace: eluent A 0.1% TFA in water, eluent B 0.1% TFA in CH_3CN/water 4/1 v/v, C18 column, gradient 0-50% B in 15 min, 50 °C, 1 mL min^{-1}, UV detection at 215 nm.

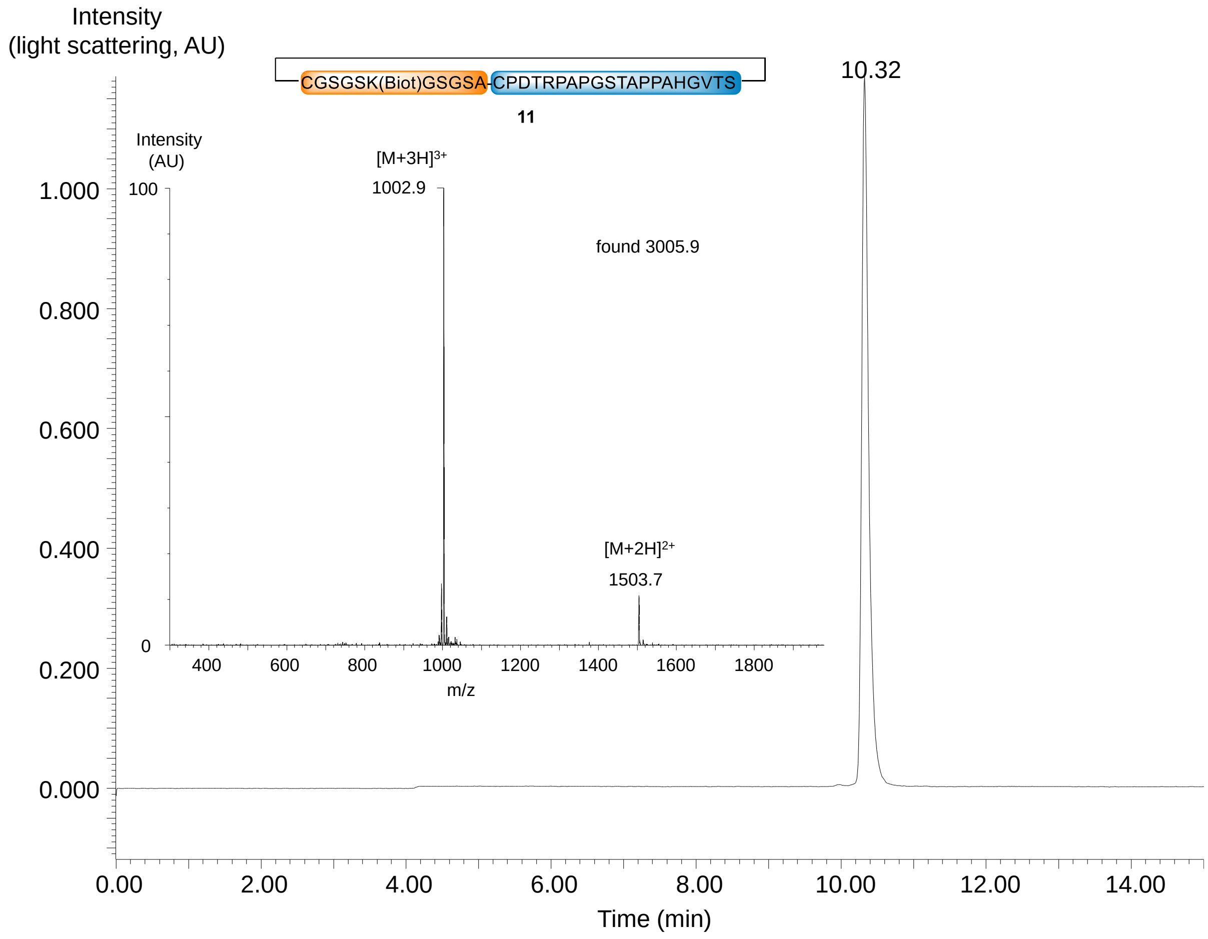

Supplementary Figure 111. LC-MS analysis of purified cyclic peptide **11**. LC trace: eluent A 0.1% TFA in water, eluent B 0.1% TFA in CH_3CN/water 4/1 v/v, C18 Xbridge BEH 300 Å 5 µm (4.6 × 250 mm) column, 30 °C, gradient 0-50% B in 15 min, 1 mL min^{-1}, light scattering detection. MS trace: m/z = 1503.7 ($[M+2H]^{2+}$), 1002.9 ($[M+3H]^{3+}$); calcd for M: 3006.3, found: 3005.9.

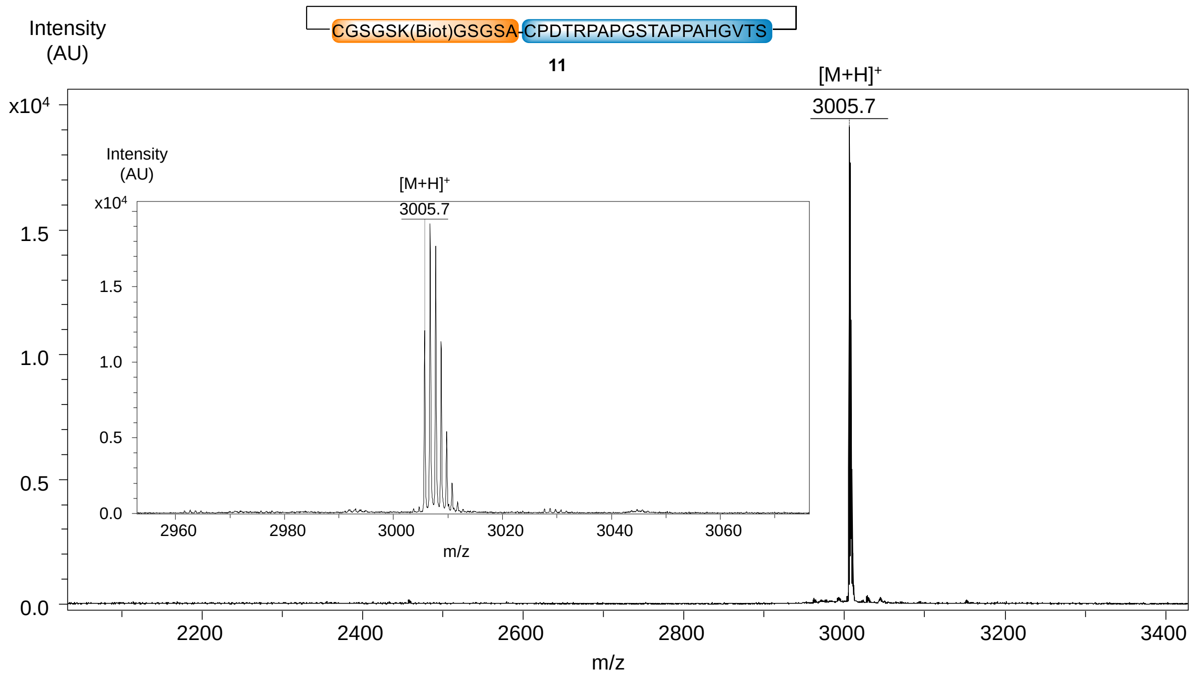

Supplementary Figure 112. MALDI-TOF analysis of cyclic peptide **11**. Matrix α-cyano-4-hydroxycinnamic acid, positive detection mode, calcd. for $[M+H]^+$ (monoisotopic): 3005.3, found: 3005.7.

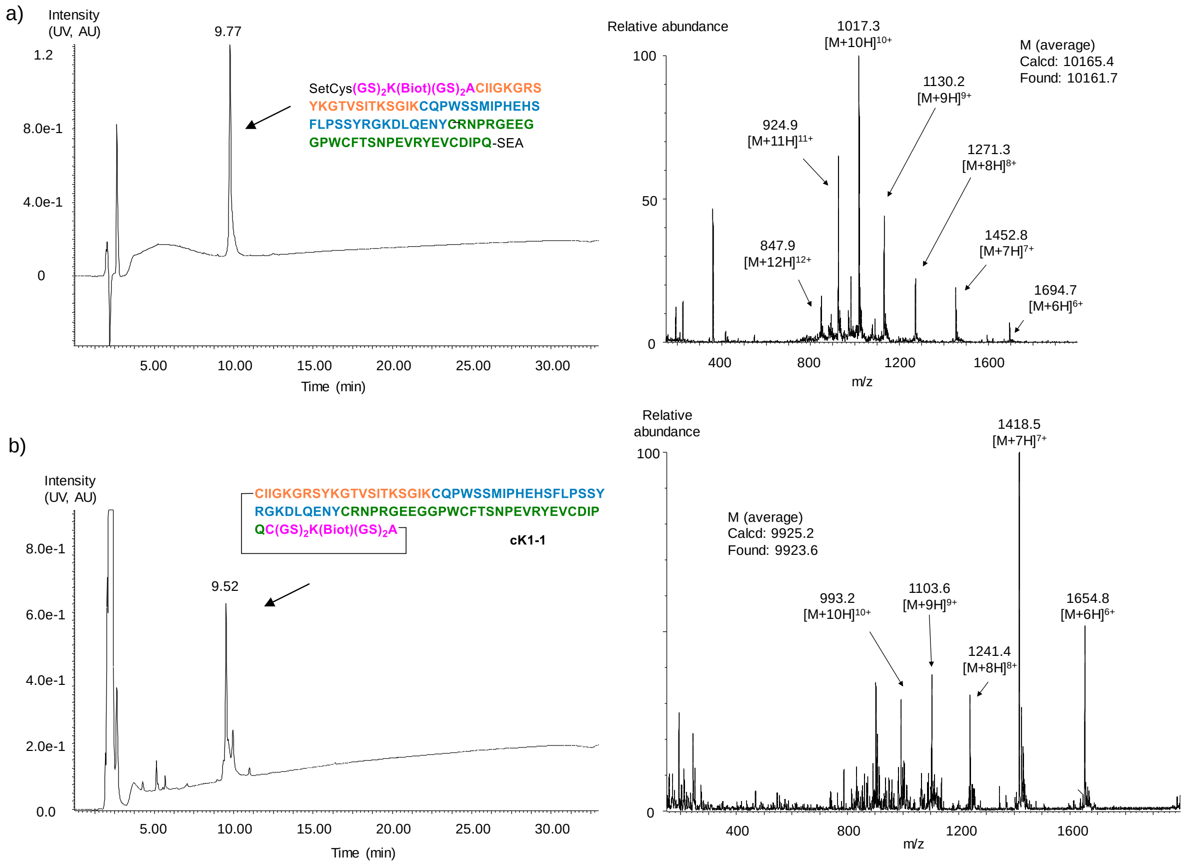

Supplementary Figure 113. Assembly of **cK1-1** monitored by LC-MS. a) Analysis of the reaction mixture 2 h after mixing the two partners of the ligation reaction. b) Analysis of the reaction mixture 40 h after the addition of TCEP. LC trace: eluent A 0.1% TFA in water, eluent B 0.1% TFA in CH_3CN/water 4/1 v/v, C18 column, gradient 0-100% B in 30 min, 50 °C, 1 mL min^{-1}, UV detection at 215 nm.

a)

b)

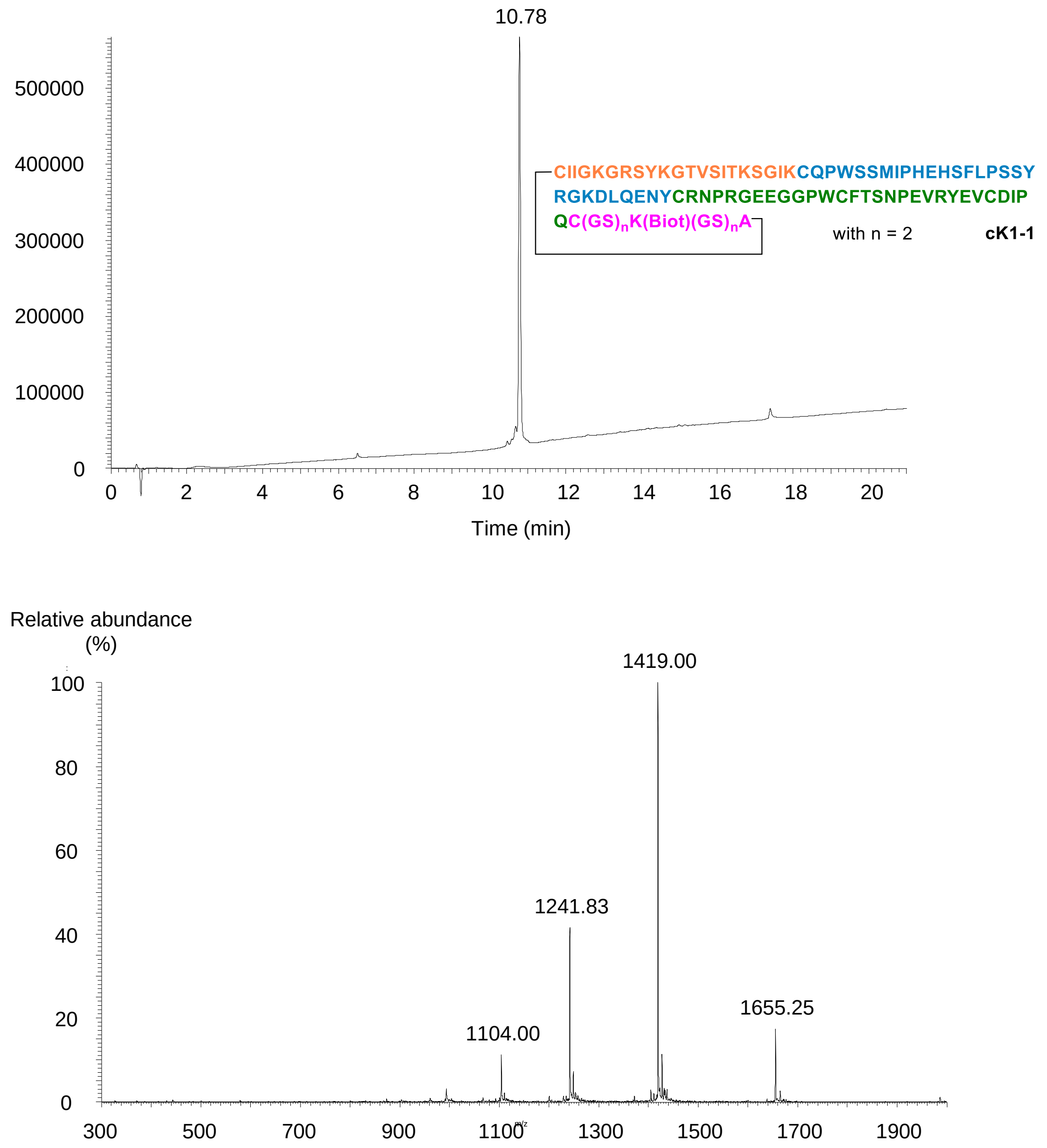

Supplementary Figure 114. LC-MS analysis of **cK1-1**. a) LC trace. Eluent A 0.1% TFA in water, eluent B 0.1% TFA in CH_3CN, ACQUITY UPLC Peptide BEH 300Å 1.7 µm (2.1 × 150 mm) column, 50 °C, gradient 0-70% B in 20 min, 0.4 mL min^{-1}, UV detection. b) MS trace: m/z = 1655.25 ($[M+6H]^{6+}$), 1419.00 ($[M+7H]^{7+}$), 1241.83 ($[M+8H]^{8+}$), 1104.00 ($[M+10H]^{10+}$); calcd. for M (average): 9925.19, found: 9926.28.

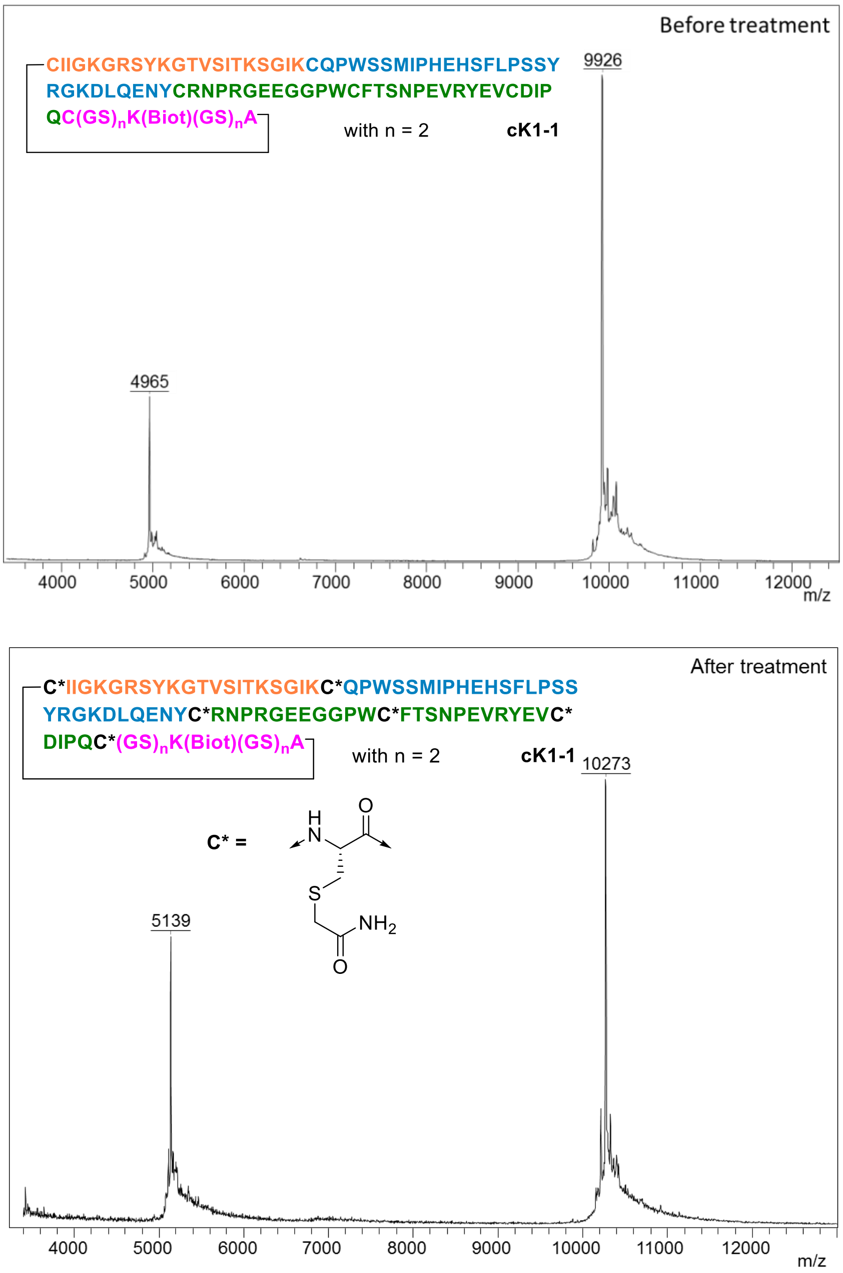

Supplementary Figure 115. Monitoring of the **cK1-1** alkylation step by MALDI-TOF MS (matrix: 2,5-dihydroxybenzoic acid).

a)

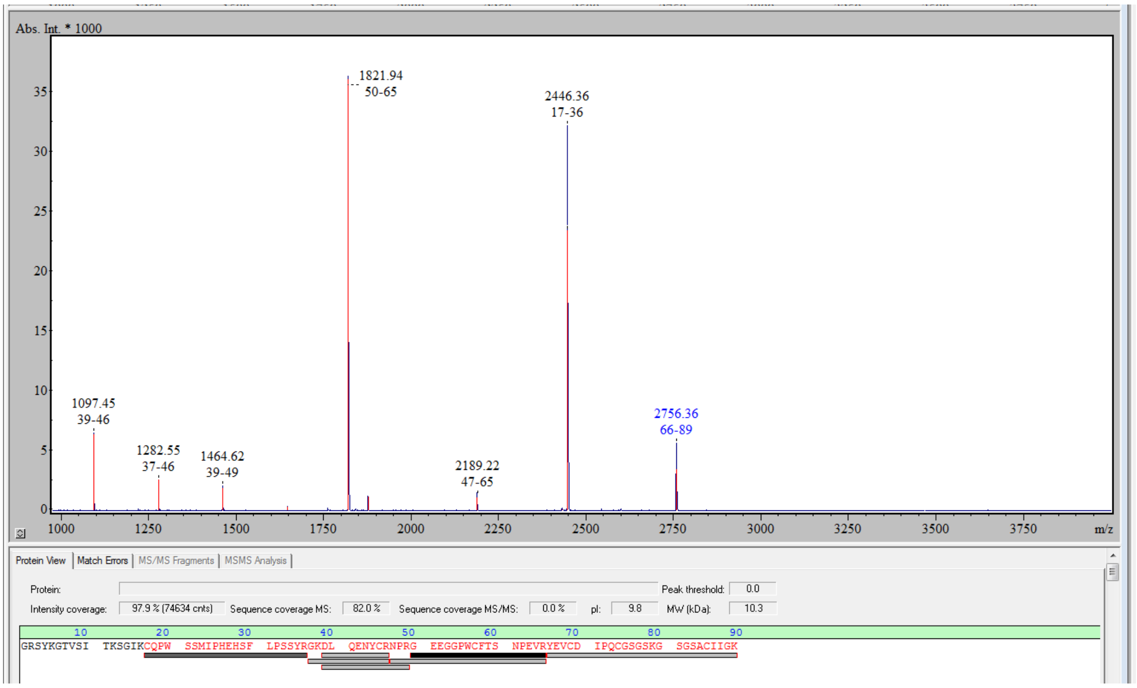

b)

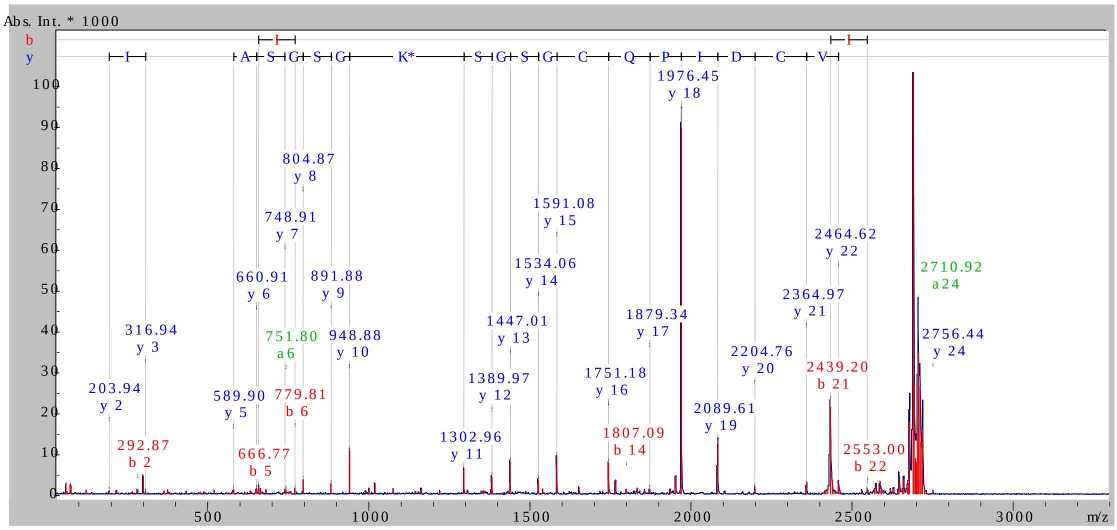

Supplementary Figure 116. MALDI-TOF TOF sequencing of cyclic peptide **cK1-1** after alkylation and trypsin cleavage using 2,5-dihydroxybenzoic acid as matrix (positive reflector mode). a) MS analysis of the enzymatic lysate (after 90 min). b) MS-MS sequencing of the ion at m/z 2756.36.

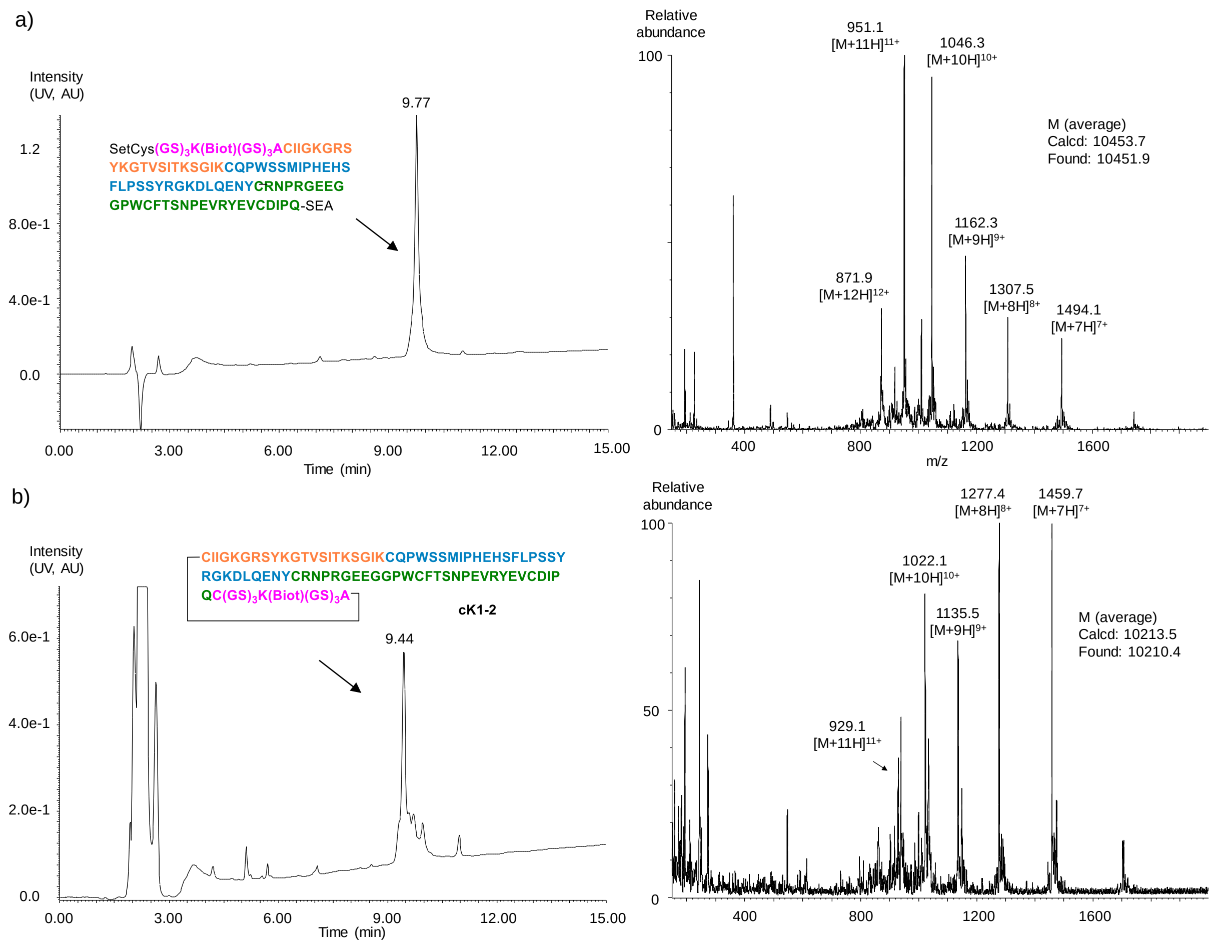

Supplementary Figure 117. Assembly of **cK1-2** monitored by LC-MS. a) Analysis of the reaction mixture 2 h after mixing the two partners of the ligation reaction. b) Analysis of the reaction mixture 16 h after the addition of TCEP. LC trace: eluent A 0.1% TFA in water, eluent B 0.1% TFA in CH_3CN/water 4/1 v/v, C18 column, gradient 0-100% B in 30 min, 50 °C, 1 mL min^{-1}, UV detection at 215 nm.

a)

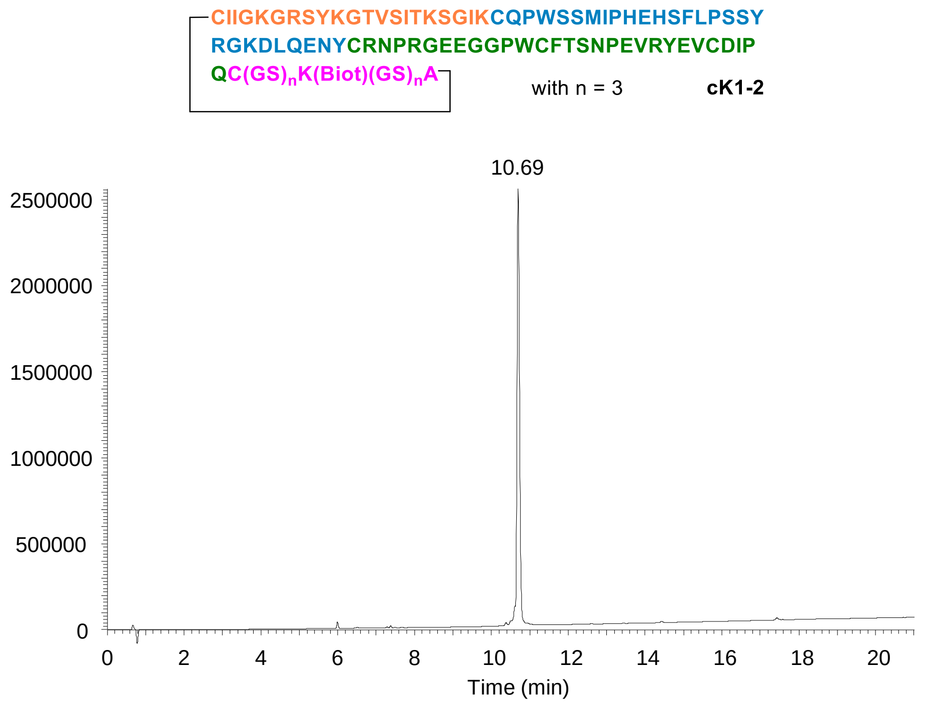

b)

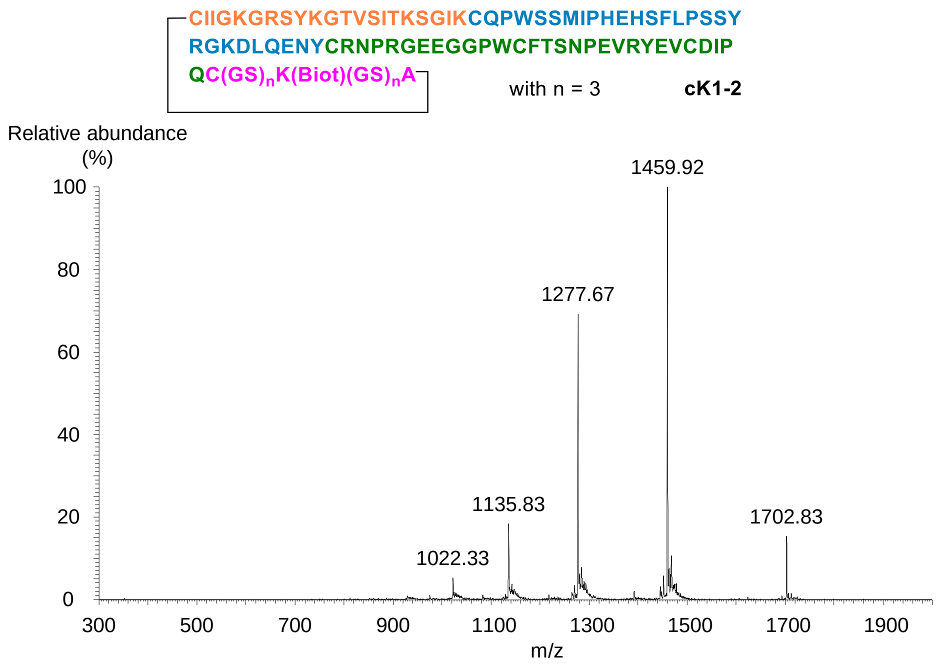

Supplementary Figure 118. LC-MS analysis of **cK1-2**. a) LC trace. Eluent A 0.1% TFA in water, eluent B 0.1% TFA in CH_3CN, ACQUITY UPLC Peptide BEH 300Å 1.7 µm (2.1 × 150 mm) column, 50 °C, gradient 0-70% B in 20 min, 0.4 mL min^{-1}, UV detection at 215 nm. b) MS trace: m/z = 1702.83 ($[M+6H]^{6+}$), 1459.92 ($[M+7H]^{7+}$), 1277.67 ($[M+8H]^{8+}$), 1135.83 ($[M+9H]^{9+}$), 1022.33 ($[M+10H]^{10+}$); calcd. for M: 10213.45, found: 10212.71.

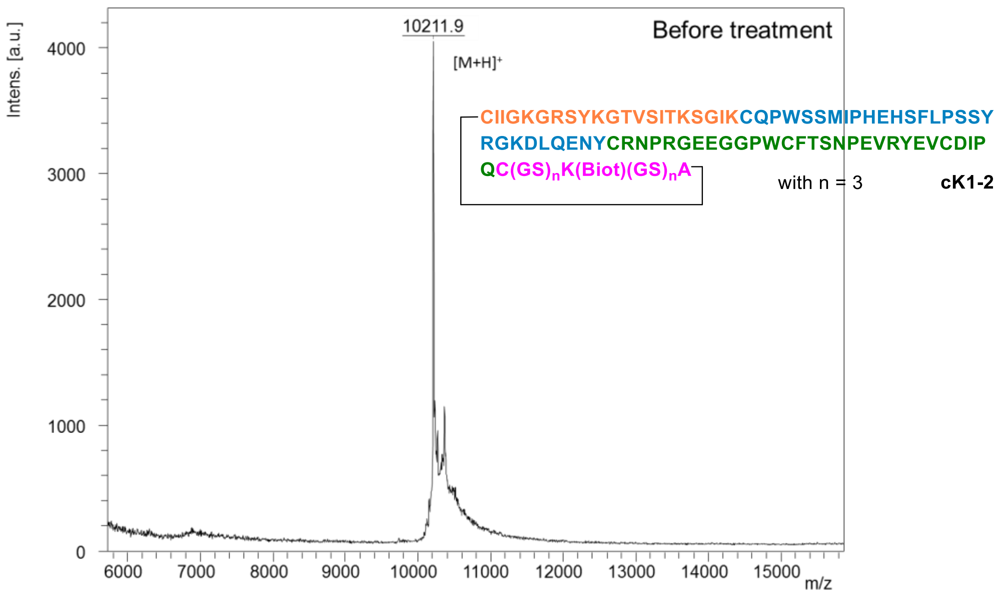

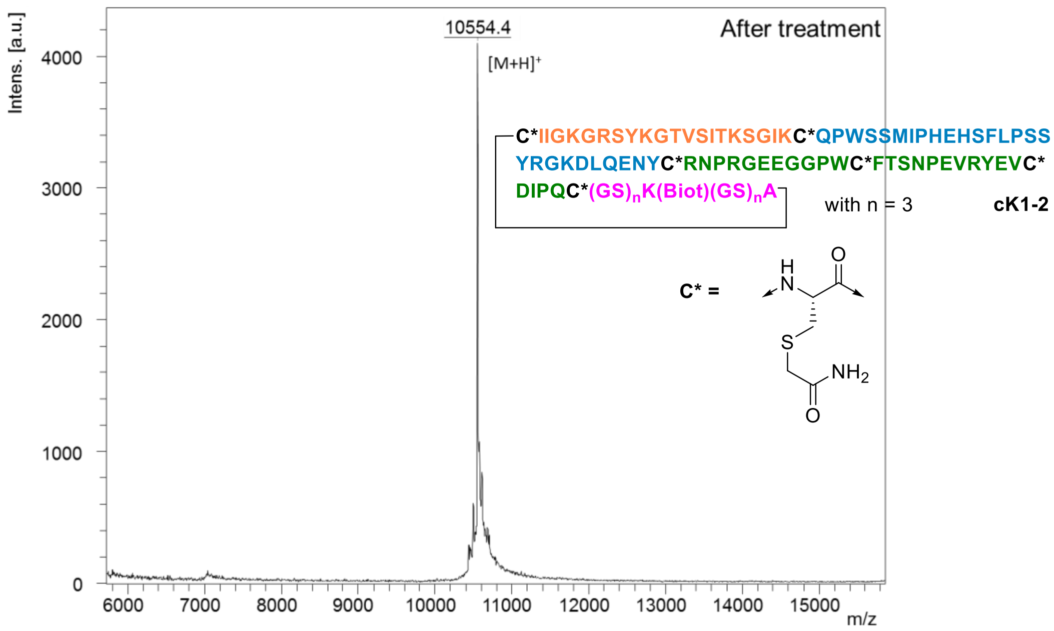

Supplementary Figure 119. Monitoring of **cK1-2** alkylation step by MALDI-TOF MS (matrix: 2,5-dihydroxybenzoic acid).

a)

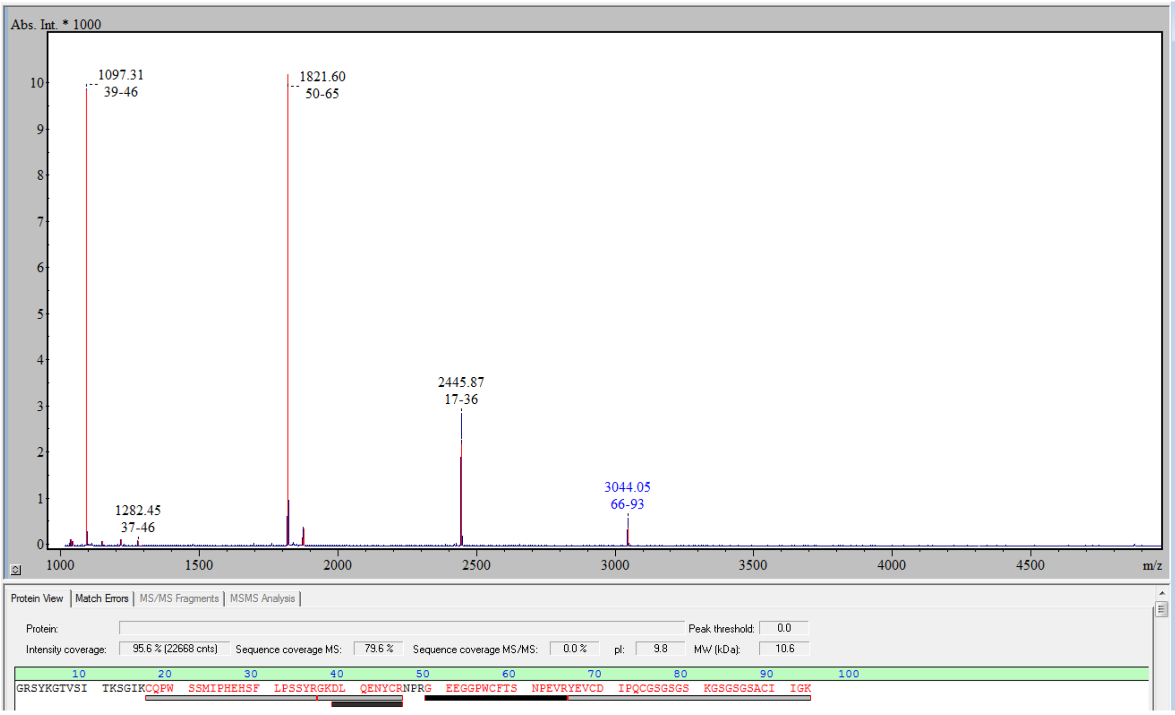

b)

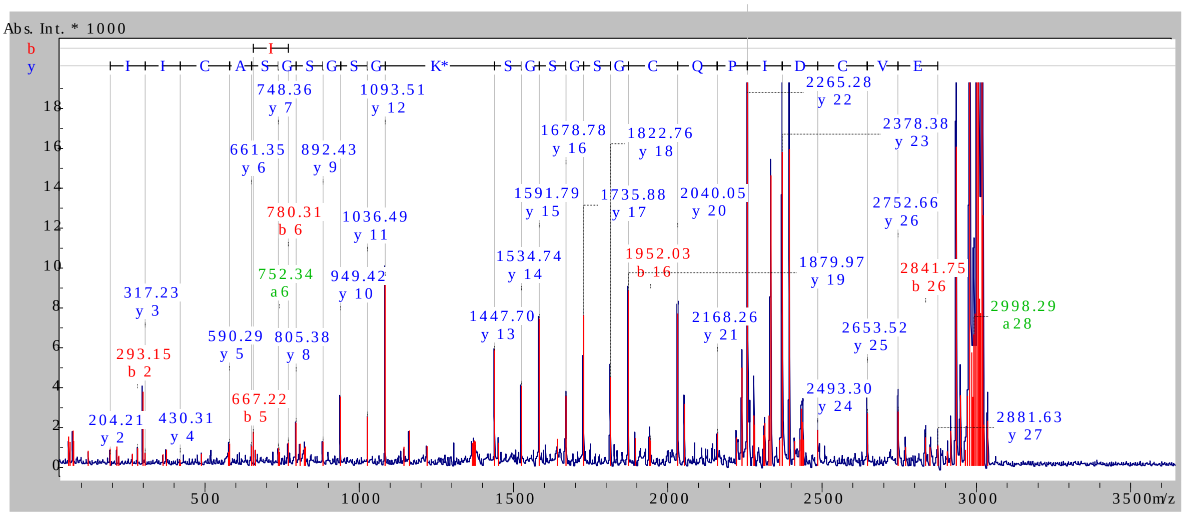

Supplementary Figure 120. MALDI-TOF TOF sequencing of cyclic peptide **cK1-2** after alkylation and trypsin cleavage using 2,5-dihydroxybenzoic acid as matrix (positive reflector mode). a) MS-analysis of the enzymatic lysate. b) MS-MS sequencing of the ion at m/z 3044.05.

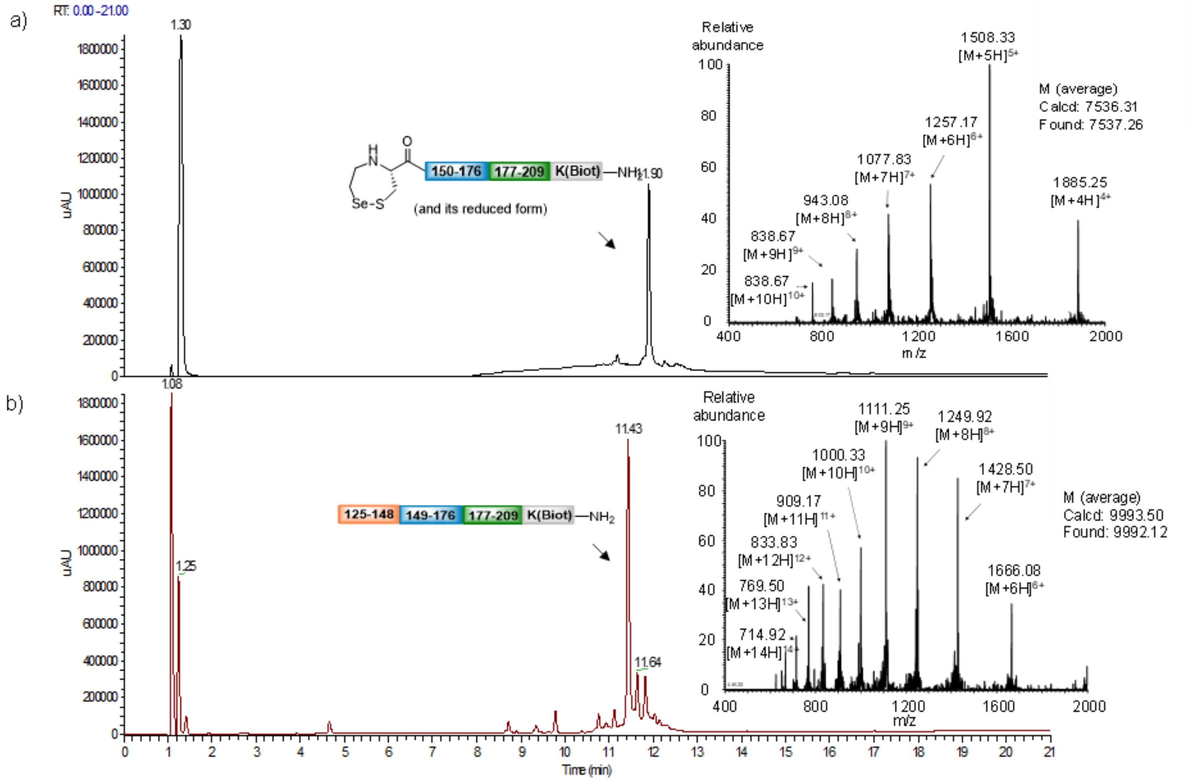

Supplementary Figure 121. Assembly of linear K1 polypeptide **15** (Method A) monitored by UPLC-MS. a) Analysis of the reaction mixture 2 h after mixing peptides SetCys-K1[150-176]-MPA (**13**) and K1[177-209]-K(Biot)-NH_2 (**14**). The elongated SetCys peptide was detected in mixture with its reduced form, due to the addition of TCEP in the analyzed sample (to reverse MPAA adducts). b) Analysis of the reaction mixture 48 h after the addition of the SEA peptide K1[125-148]-SEA (**12**) and TCEP. LC trace: eluent A 0.1% TFA in water, eluent B 0.1% TFA in CH_3CN, ACQUITY UPLC Peptide BEH 300 Å 1.7 µm (2.1 × 150 mm) column, 50 °C, gradient 0-70% B in 20 min, 0.4 mL min^{-1}, UV detection.

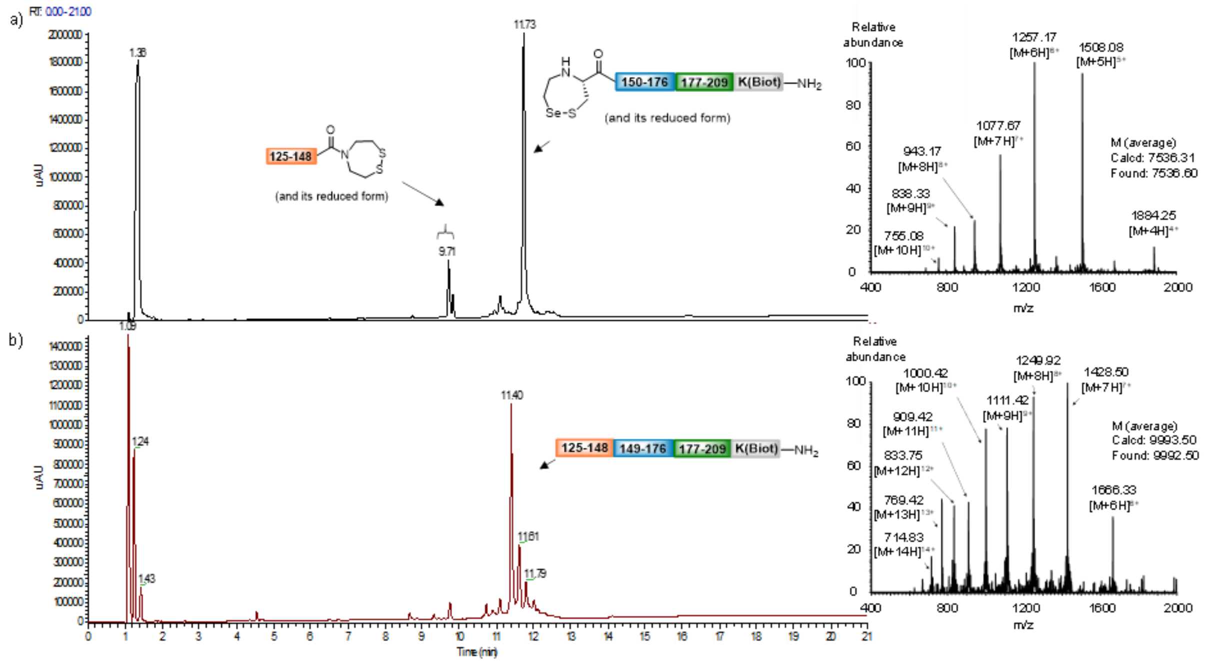

Supplementary Figure 122. Assembly of linear K1 polypeptide **15** (Method B) monitored by UPLC-MS. a) Analysis of the reaction mixture 4.5 h after mixing peptides K1[125-148]-SEA (**12**), SetCys-[150-176]K1-MPA (**13**) and K1[177-209]-K(Biot)-NH_2 (**14**). The elongated SetCys peptide and the remaining SEA peptide segments are detected in mixture with their reduced form, due to the addition of TCEP in the analyzed sample (to reverse MPAA and S*t*Bu adducts). b) Analysis of the reaction mixture 40 h after the addition of TCEP. LC trace: eluent A 0.1% TFA in water, eluent B 0.1% TFA in CH_3CN, ACQUITY UPLC Peptide BEH 300 Å 1.7 µm (2.1 × 150 mm) column, 50 °C, gradient 0-70% B in 20 min, 0.4 mL min^{-1}, UV detection at 215 nm.

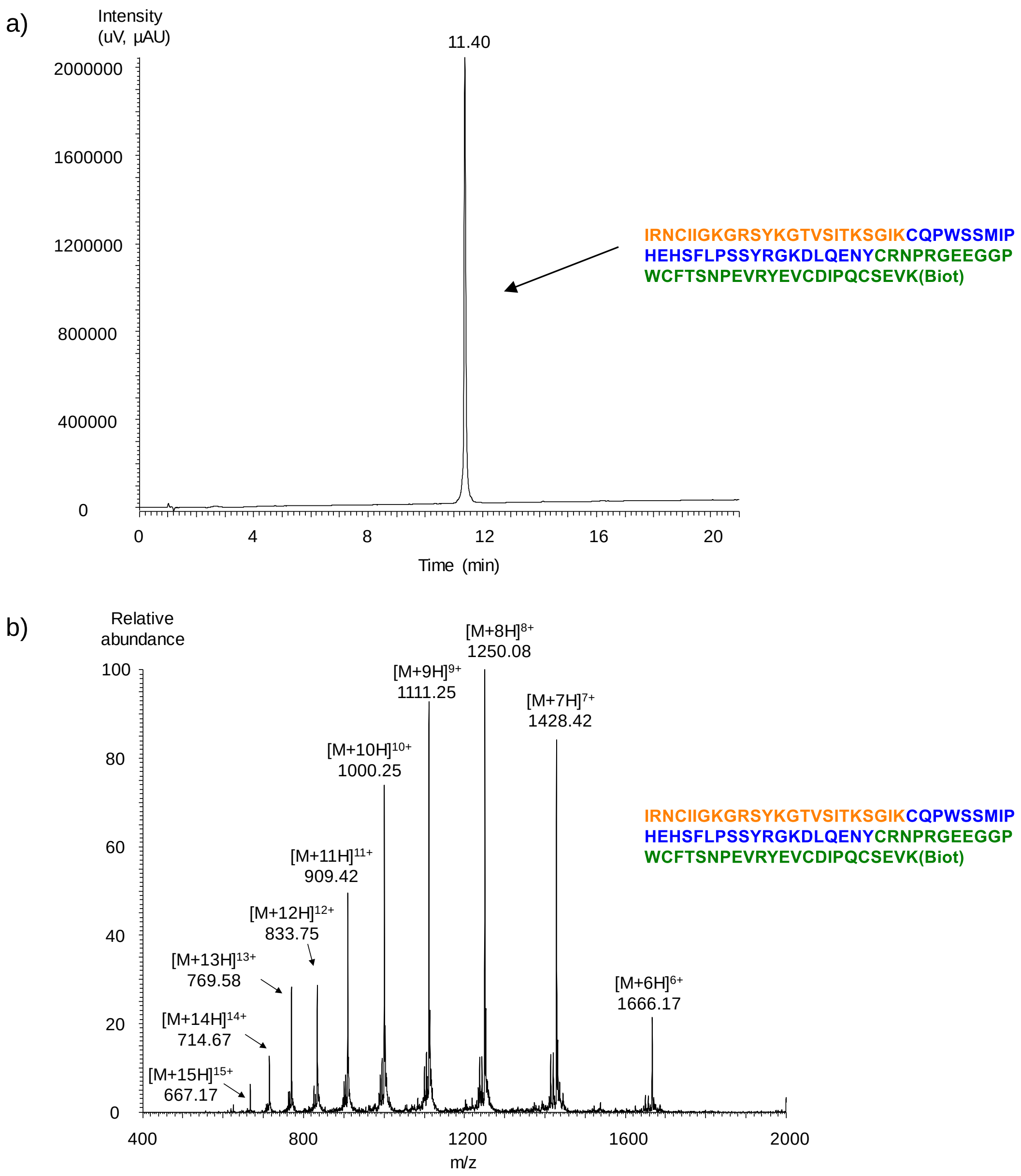

Supplementary Figure 123. LC-MS analysis of linear K1 polypeptide **15**. a) LC trace. Eluent A 0.1% TFA in water, eluent B 0.1% TFA in CH_3CN, ACQUITY UPLC Peptide BEH 300 Å 1.7 µm (2.1 × 150 mm) column, 50 °C, gradient 0-70% B in 20 min, 0.4 mL min^{-1}, UV detection. b) MS trace: m/z = 1666.17 ([M+6H]$^{6+}$), 1428.42 ([M+7H]$^{7+}$), 1250.08 ([M+8H]$^{8+}$), 1111.25 ([M+9H]$^{9+}$), 1000.25 ([M+10H]$^{10+}$), 909.42 ([M+11H]$^{11+}$), 833.75 ([M+12H]$^{12+}$), 769.58 ([M+13H]$^{13+}$), 714.67 ([M+14H]$^{14+}$), 667.17 ([M+15H]$^{15+}$). Calcd. for M (average): 9993.40, found: 9992.14.

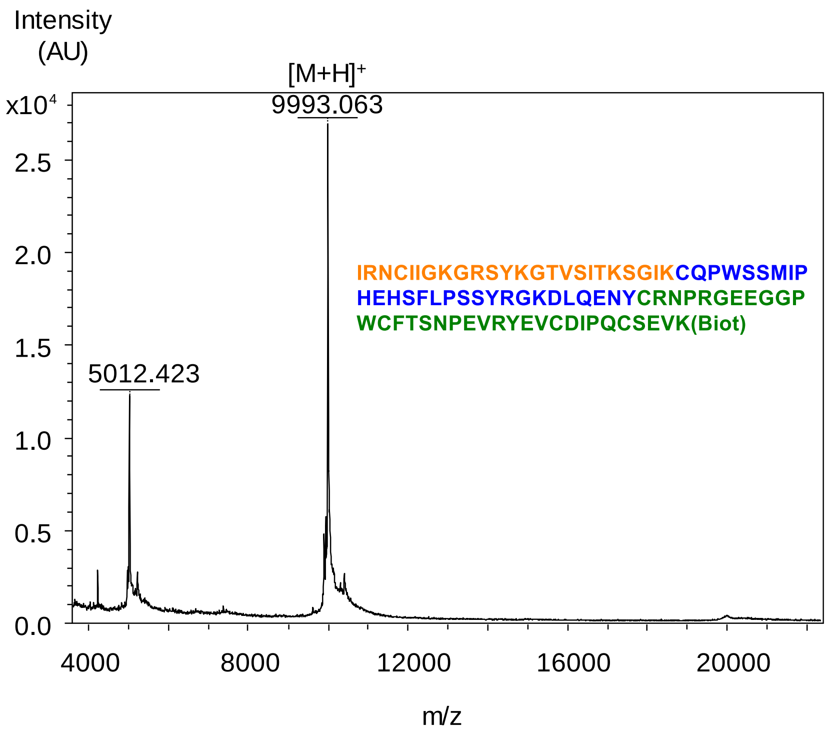

Supplementary Figure 124. MALDI-TOF analysis of linear K1 polypeptide **15**. Matrix: 2,5-dihydroxybenzoic acid, positive detection mode, calcd. for $[M+H]^+$ (average): 9994.4, found: 9993.1.

Before treatment:

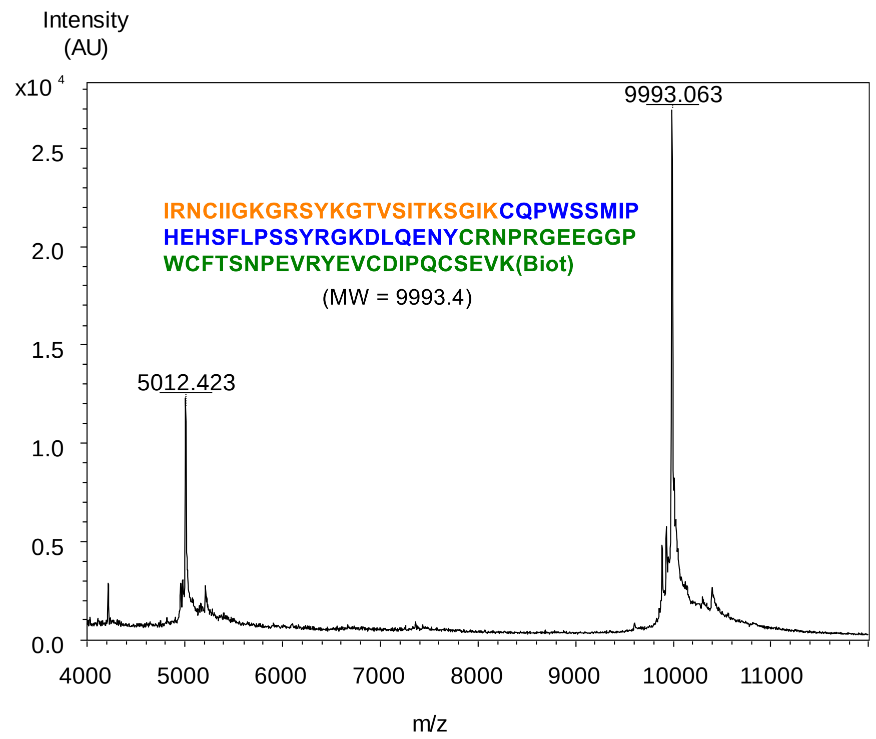

After treatment:

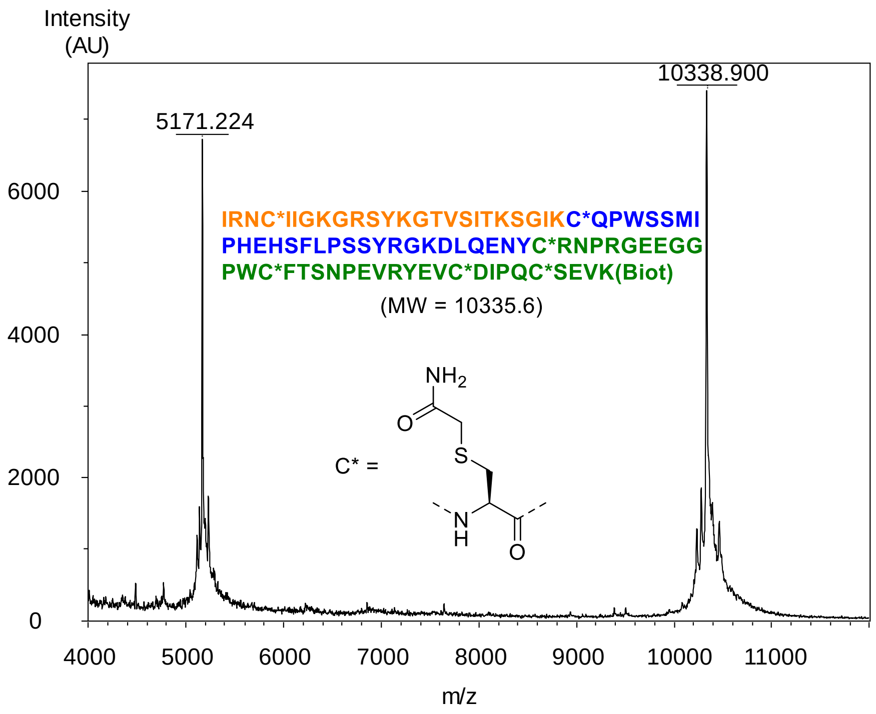

Supplementary Figure 125. Monitoring of polypeptide **15** alkylation by MALDI-TOF MS (matrix: 2,5-dihydroxybenzoic acid).

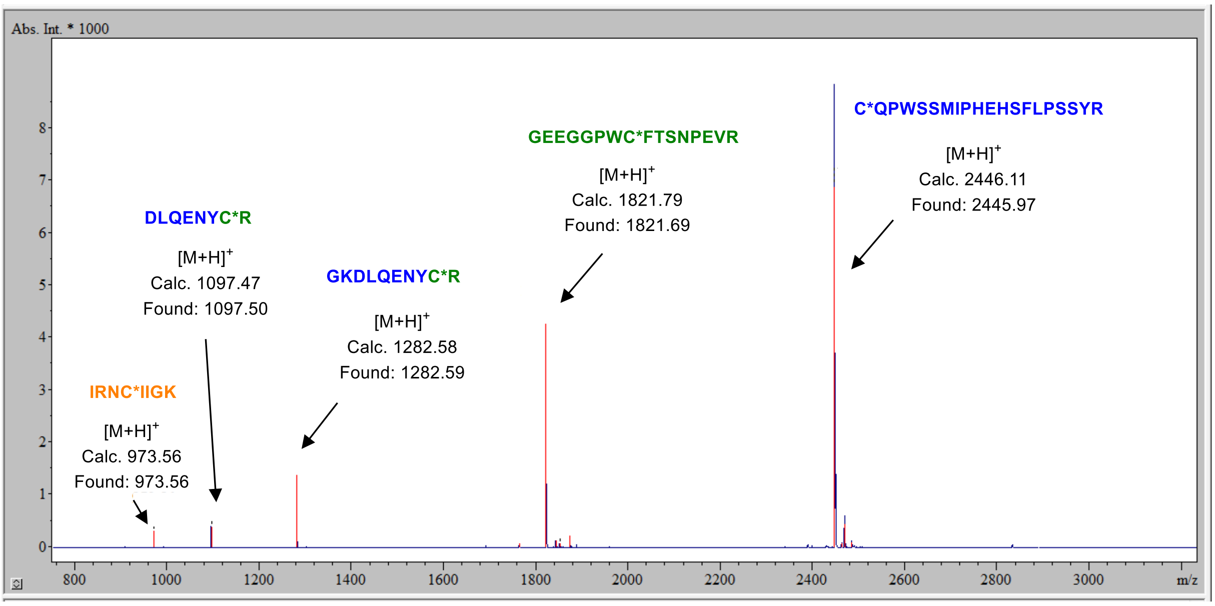

Supplementary Figure 126. MALDI-TOF analysis of the fragments obtained by enzymatic digestion of polypeptide **15**. Matrix: 2,5-dihydroxybenzoic acid, positive detection mode. Analysis of the fragments produced by enzymatic cleavage of alkylated polypeptide **15** confirms the Tyr-Cys junction formed by NCL and the conversion of the SetCys residue into Cys residue during the second ligation step.

Protein folding

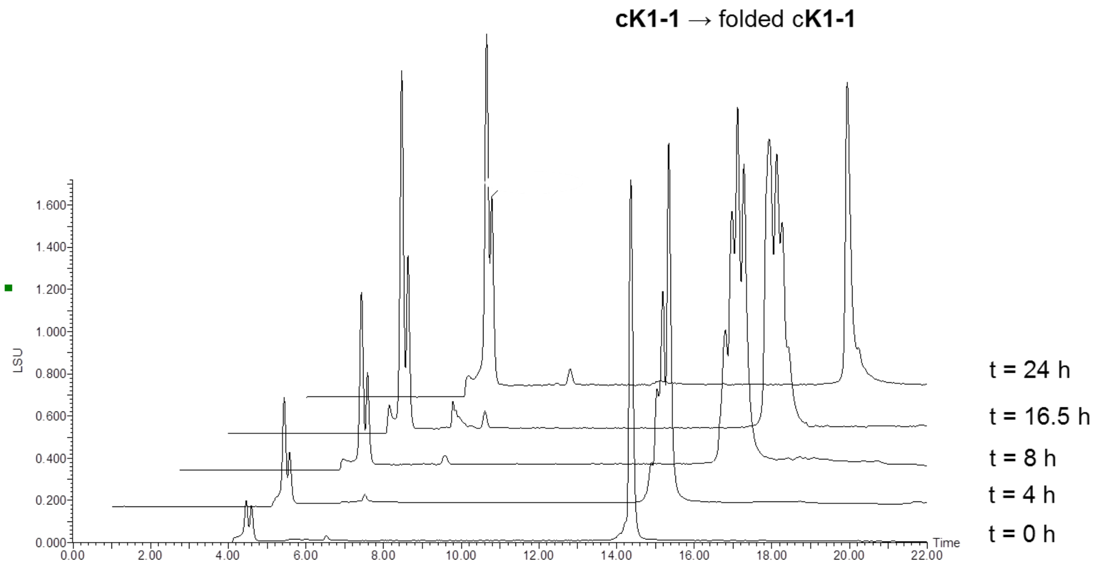

Supplementary Figure 127. Kinetics of **cK1-1** folding monitored by LC-MS. Elution conditions: eluent A 0.1% TFA in water, eluent B 0.1% TFA in CH_3CN/water 4/1 v/v, XBridge Peptide BHE C18 3.5 µm (4.6×150 mm) column, gradient 0-50% B in 15 min, 50 °C, 1 mL min^{-1}, light scattering detection.

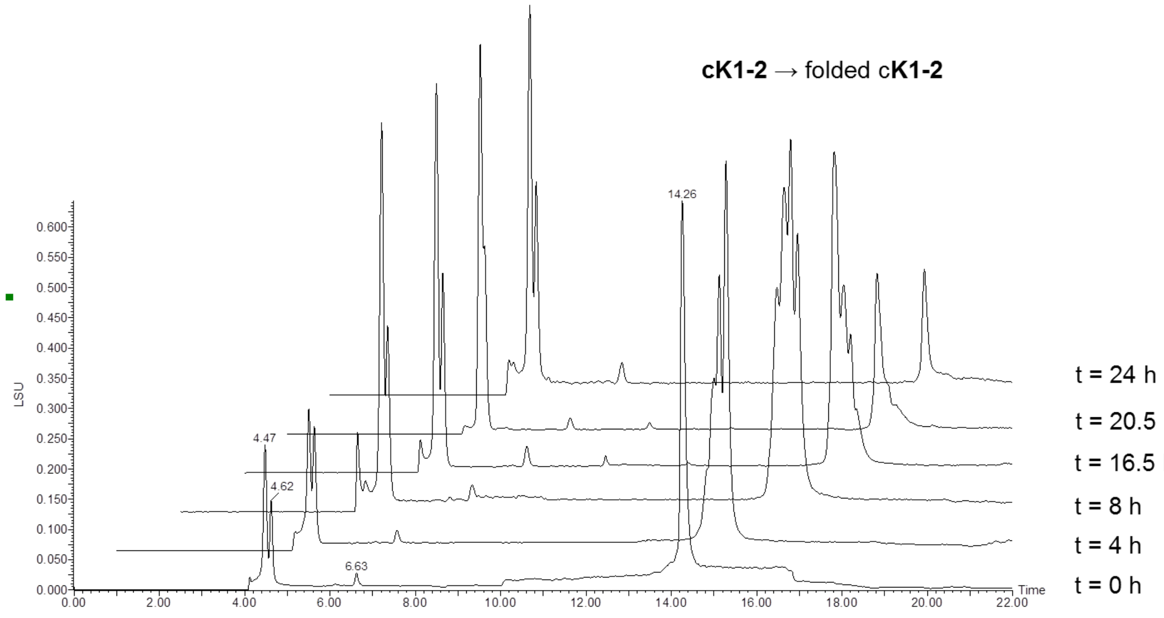

Supplementary Figure 128. Kinetics of **cK1-2** folding studied by LC-MS. Elution conditions: eluent A 0.1% TFA in water, eluent B 0.1% TFA in CH_3CN/water 4/1 v/v, XBridge Peptide BHE C18 3.5 µm (4.6×150 mm) column, gradient 0-50% B in 15 min, 50 °C, 1 mL min^{-1}, light scattering detection.

a)

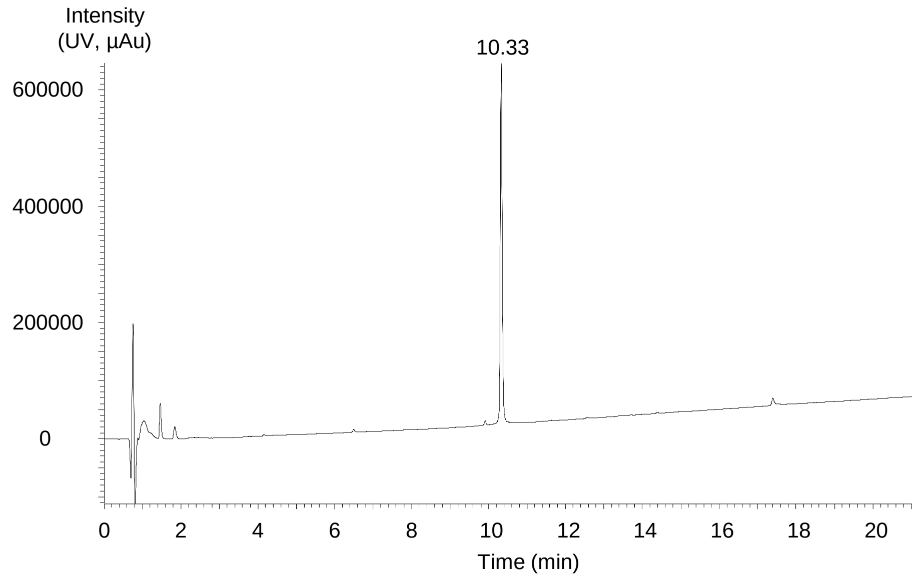

b)

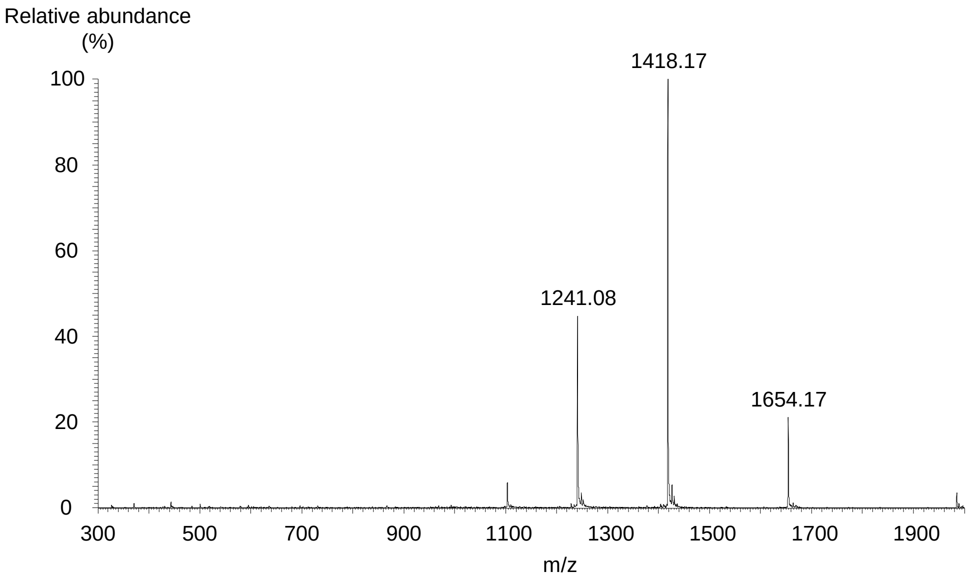

Supplementary Figure 129. LC-MS analysis of **cK1-1f**. a) LC trace. Eluent A 0.1% TFA in water, eluent B 0.1% TFA in CH_3CN, ACQUITY UPLC Peptide BEH 300Å 1.7 µm (2.1 × 150 mm) column, 50 °C, gradient 0-70% B in 20 min, 0.4 mL min^{-1}, UV detection at 215 nm. b) MS trace. m/z = 1654.17 ($[M+6H]^{6+}$), 1418.17 ($[M+7H]^{7+}$), 1241.08 ($[M+8H]^{8+}$), calcd for M (average): 9919.13, found: 9919.95.

a)

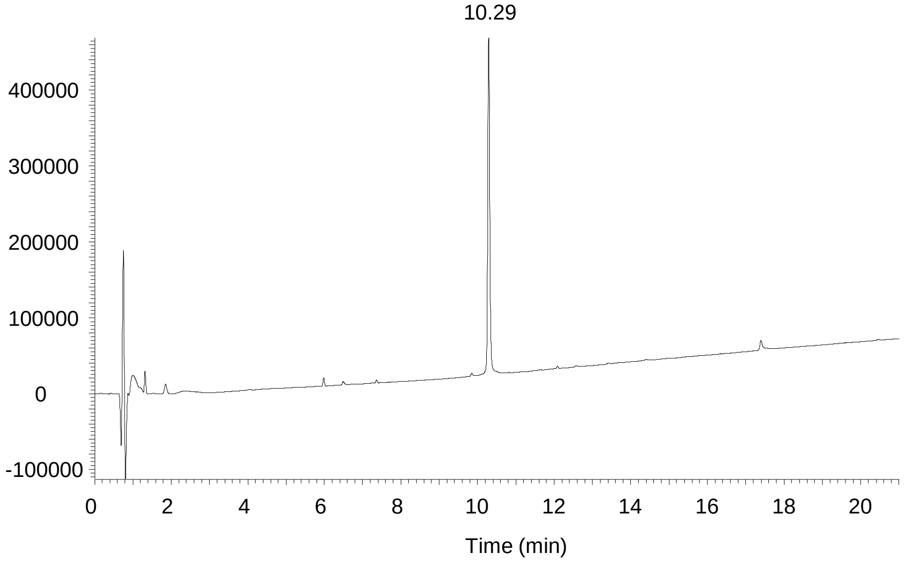

b)

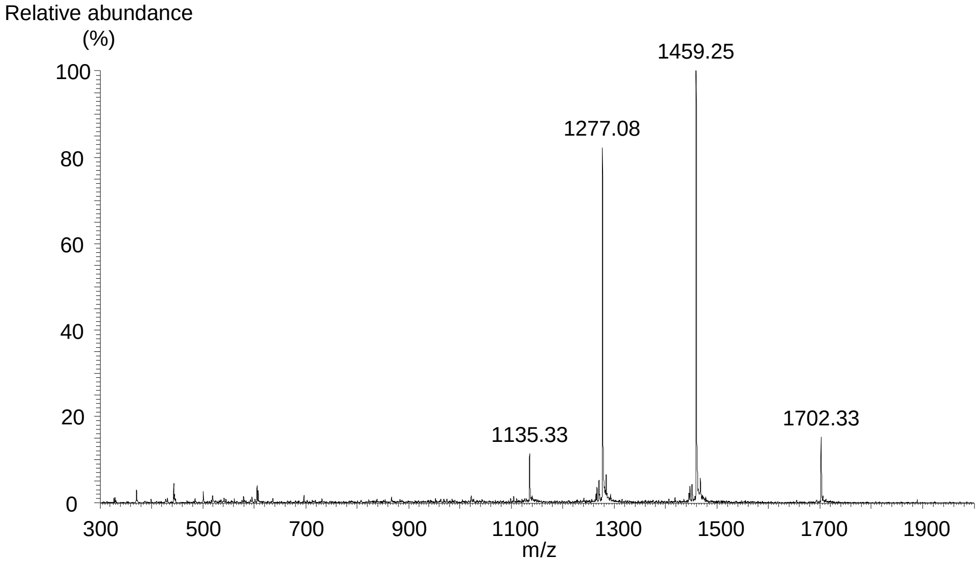

Supplementary Figure 130. LC-MS analysis of **cK1-2f**. a) LC trace. Eluent A 0.1% TFA in water, eluent B 0.1% TFA in CH_3CN, ACQUITY UPLC Peptide BEH 300Å 1.7 µm (2.1 × 150 mm) column, 50 °C, gradient 0-70% B in 20 min, 0.4 mL min^{-1}, UV detection. b) MS trace: m/z = 1702.33 ($[M+6H]^{6+}$), 1459.25 ($[M+7H]^{7+}$), 1277.08 ($[M+8H]^{8+}$), 1135.33 ($[M+9H]^{9+}$); calcd for M (average): 10207.39, found 10208.34.

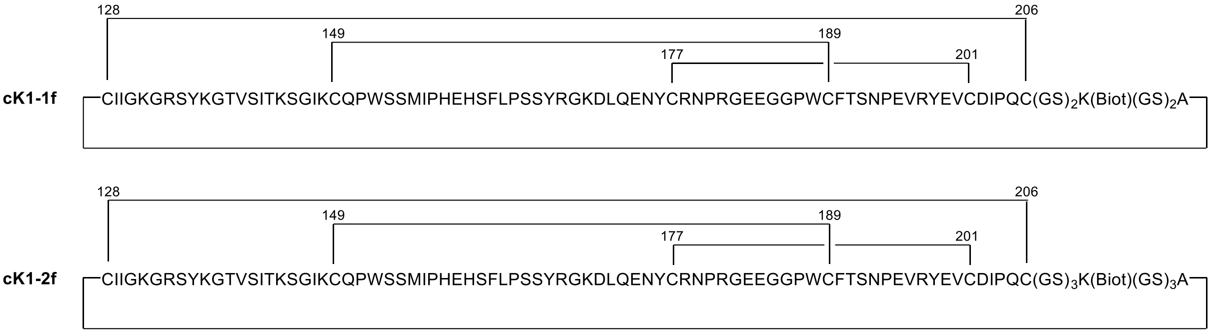

Supplementary Figure 131. Disulfide bridge patterns of proteins **cK1-1f** and **cK1-2f**.

a)

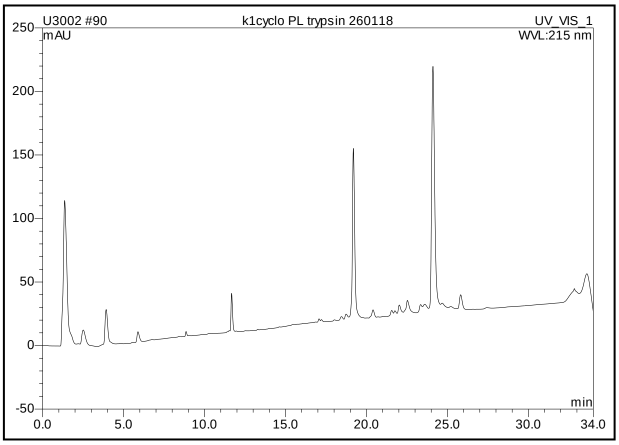

b)

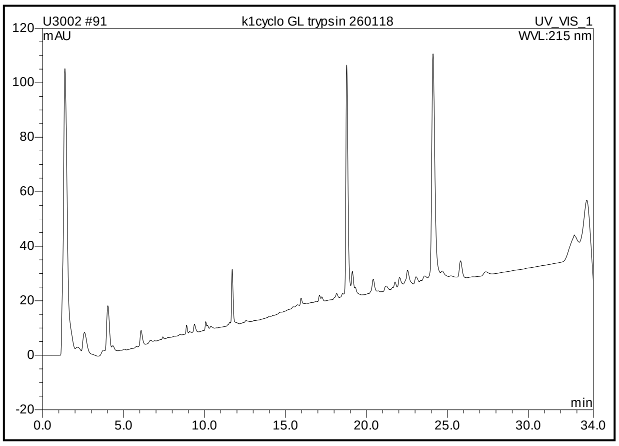

Supplementary Figure 132. HPLC analysis of the trypsic digest. Chromatogram obtained for **cK1-1f** (a) and **cK1-2f** (b). HPLC conditions: eluent A 0.1% TFA in water, eluent B 0.1% TFA in CH_3CN/water 4/1 v/v, C18 XBridge BEH 300 Å 3.5 μm 2.1×150 mm column, 50 °C, gradient 0-40% B in 40 min, 0.4 mL min^{-1}, detection at 215 nm.

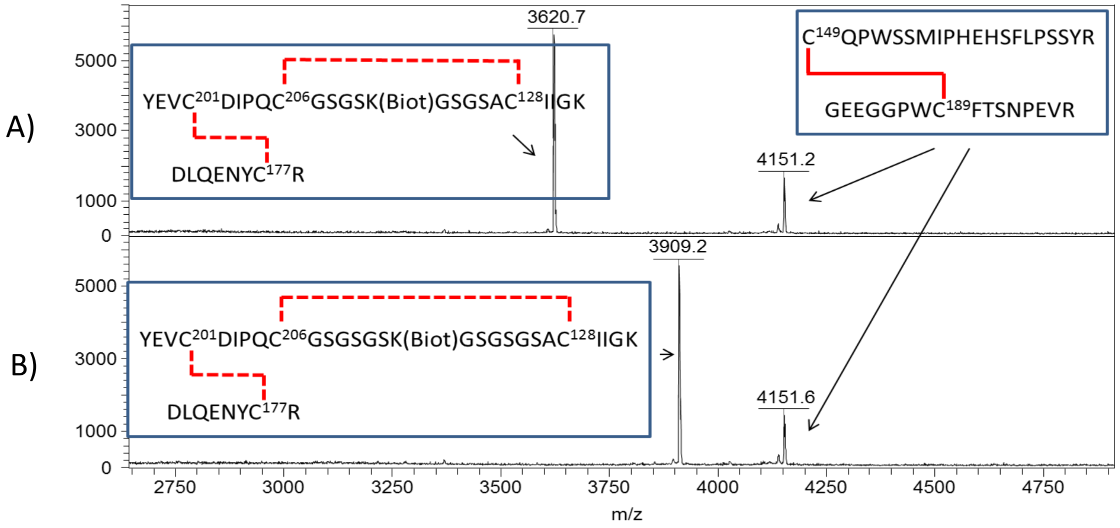

Supplementary Figure 133. MALDI-TOF analysis of the trypsic digest: results obtained with **cK1-1f** (A) and **cK1-2f** (B). Matrix 2,5-dihydroxybenzoic acid, positive detection mode.

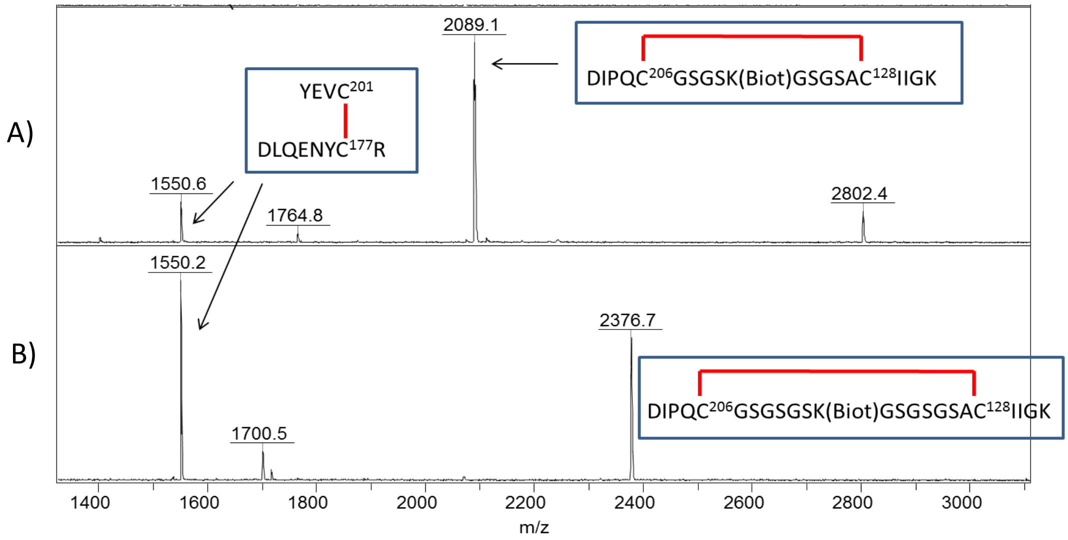

Supplementary Figure 134. MALDI-TOF analysis of the endoproteinase *N*-Asp digest: results obtained with **cK1-1f** (A) and **cK1-2f** (B). Matrix 2,5-dihydroxybenzoic acid, positive detection mode.

AlphaScreen and cell-based assays

$$\% \, Max \; Alpha \; signal = \frac{100}{1 + 10^{LogC - LogEC50}}$$

Supplementary Figure 135. Sigmoidal equation used for the non-linear regression analysis and determination of EC50. *C* is for NK1 concentration.

Supplementary Tables

Supplementary Table 1. Kinetic data extracted from curve fitting in Supplementary Figure 68**.**

[MPAA] (mM)	[DTT] (mM)	[TCEP] (mM)	[ascorbate] (mM)	k_1 (min^{-1})	$t_{1/2}$ (h)
**200**	0	0	0	0	0
200	**100**	0	0	$(6.7 \pm 1.6) \times 10^{-4}$	16.5
200	0	**100**	100	$(1.8 \pm 0.8) \times 10^{-3}$	6.4

Rate constants were extracted using KinteK Explorer Software*TM*. A standard deviation estimate for experimental measurements was obtained from fitting with a multi-exponential function. Rate constants and associated standard errors were produced by chi² minimization fitting. Standard errors were estimated based upon the covariance matrix using Kintek software.

Supplementary Table 2. Rate constants and half-reaction times determined from data in Supplementary Figure 65.

Product of ligation	k (min^{-1})	$t_{1/2}$ (h)
RLKEPVHG**A-C**ILKEPVHGV-NH_2	$(4.95 \pm 0.28) \times 10^{-2}$	0.22
ILKEPVHG**V-C**ILKEPVHGV-NH_2	$(4.32 \pm 0.10) \times 10^{-3}$	2.62

Rate constants were extracted using KinteK Explorer Software*TM*. A standard deviation estimate for experimental measurements was obtained from fitting with a multi-exponential function. Rate constants and associated standard errors were produced by chi² minimization fitting. Standard errors were estimated based upon the covariance matrix using Kintek software.

Supplementary Table 3. Effect of the pH on the conversion of SetCys-ALKEPVHGV-NH_2 **1** into CALKEPVHGV-NH_2 **3**.

pH	k_1 (min^{-1})	standard error
4.99	0.00135	0.00086
5.25	0.00184	0.00066
5.54	0.002	0.00075
5.98	0.00242	0.00042
6.52	0.002	0.00117
6.90	0.00172	0.00066
7.22	0.00118	0.00085
7.55	0.00087	0.00004
8.03	0.00046	0.00007
8.49	0.00014	0.00005

Rate constants were extracted using KinteK Explorer Software*TM*. The reactions leading to the loss of the SetCys *N*-selenoethyl appendage were performed once at each pH value from 4.99 to 8.49. A standard deviation estimate for experimental measurements was obtained from fitting

with a multi-exponential function. Rate constants k_1 and associated standard errors were produced by chi² minimization fitting. Standard errors were estimated based upon the covariance matrix using Kintek software.

Supplementary Table 4. Second-order rate constants for the model NCL reaction.

	value (M^{-1} min^{-1})	Std error
k_{+2}	0.257	0.012
k_{-2}	129	34
k_4	243	6

The model NCL reaction was performed once (n = 1). Corresponding rate constants k_{+2}, k_{-2}, k_4 and associated standard errors were determined by fitting 35 experimental measures from two traces to the model (a standard deviation estimate was determined for each trace from fitting with a multi-exponential function). Standard errors produced by chi² minimization fitting were estimated based upon the covariance matrix using Kintek software.

Supplementary Table 5. Rate constants for the model NCL reaction of SetCys peptides.

	Value	Std err
k_1	0.0012 min^{-1}	0.0009
k_{+2}	0.257 M^{-1} min^{-1}	0.012
k_{-2}	129 M^{-1}. min^{-1}	34
k_{+3}	182 M^{-1}. min^{-1}	49
k_{-3}	0.0632 M^{-1} min^{-1}	0.0314
k_4	243 M^{-1} min^{-1}	6
k_5	0 min^{-1}	nd

Reaction was performed once (n = 1). Estimates of standard deviation for each experimental data point were obtained from fitting each trace with a multi-exponential function. Rate constants and associated standard errors were produced by chi² minimization fitting. Standard errors were estimated based upon the covariance matrix using Kintek software. A one-sided Pearson's chi²-test was made and gave a *p*-value of 0.00323. The process was completed by careful visual examination of the fit and critical evaluation of the model.

Supplementary Table 6. Folding results for **cK1-1** and **cK1-2**.

peptide	Initial mass of peptide (in mg)	Volume after dialys (in µL)	Concentration of peptide determined by BCA (in µmol)	Yield (in %)
**cK1-1**	1.056	312	30	12
**cK1-2**	1.084	230	26	7

Supplementary References

1. Miura C., Kiyama M., Iwano S., Ito K., Obata R., Hirano T. *et al.* Synthesis and luminescence properties of biphenyl-type firefly luciferin analogs with a new, near-infrared light-emitting bioluminophore. *Tetrahedron* **69**, 9726-9734 (2013).
2. Cargoët M., Diemer V., Snella B., Desmet R., Blanpain A., Drobecq H. *et al.* Catalysis of thiol-thioester exchange by water-soluble alkyldiselenols applied to the synthesis of peptide thioesters and SEA-mediated ligation. *J. Org. Chem.* **83**, 12584–12594 (2018).
3. Ollivier N., Dheur J., Mhidia R., Blanpain A. & Melnyk O. *Bis*(2-sulfanylethyl)amino native peptide ligation. *Org. Lett.* **12**, 5238-5241 (2010).
4. Simonneau C., Berenice L., Mougel A., Adriaenssens E., Paquet C., Raibaut L. *et al.* Semi-synthesis of a HGF/SF kringle one (K1) domain scaffold generates a potent in vivo MET receptor agonist. *Chem. Sci.* **6**, 2110-2121 (2015).
5. Ollivier N., Desmet R., Drobecq H., Blanpain A., Boll E., Leclercq B. *et al.* A simple and traceless solid phase method simplifies the assembly of large peptides and the access to challenging proteins. *Chem. Sci.* **8**, 5362-5370 (2017).
6. Ollivier N., Vicogne J., Vallin A., Drobecq H., Desmet R., El-Mahdi O. *et al.* A one-pot three-segment ligation strategy for protein chemical synthesis. *Angew. Chem. Int. Ed.* **51**, 209-213 (2012).
7. Fang G.-M., Li Y.-M., Shen F., Huang Y.-C., Li J.-B., Lin Y. *et al.* Protein chemical synthesis by ligation of peptide hydrazides. *Angew. Chem. Int. Ed.* **50**, 7645-7649 (2011).
8. Ruff Y., Garavini V. & Giuseppone N. Reversible native chemical ligation: a facile access to dynamic covalent peptides. *J. Am. Chem. Soc.* **136**, 6333-6339 (2014).
