# Supplementary material for: A cysteine selenosulfide redox switch for protein chemical synthesis
Source: Nat Commun. 2020 May 22;11:2558. doi: 10.1038/s41467-020-16359-6 (PMC7244499; doi:10.1038/s41467-020-16359-6)
Supplement: Supplementary file 3 — Reporting Summary [file 41467_2020_16359_MOESM3_ESM.pdf]

nature research

Corresponding author(s): Oleg Melnyk, Vangelis Agouridas

Last updated by author(s): April 10, 2020

Reporting Summary

Nature Research wishes to improve the reproducibility of the work that we publish. This form provides structure for consistency and transparency in reporting. For further information on Nature Research policies, see Authors & Referees and the Editorial Policy Checklist .

Statistics

For all statistical analyses, confirm that the following items are present in the figure legend, table legend, main text, or Methods section.

n/a	Confirmed	
☐	☒	The exact sample size (*n*) for each experimental group/condition, given as a discrete number and unit of measurement
☐	☒	A statement on whether measurements were taken from distinct samples or whether the same sample was measured repeatedly
☐	☒	The statistical test(s) used AND whether they are one- or two-sided<br>*Only common tests should be described solely by name; describe more complex techniques in the Methods section.*
☒	☐	A description of all covariates tested
☒	☐	A description of any assumptions or corrections, such as tests of normality and adjustment for multiple comparisons
☐	☒	A full description of the statistical parameters including central tendency (e.g. means) or other basic estimates (e.g. regression coefficient) AND variation (e.g. standard deviation) or associated estimates of uncertainty (e.g. confidence intervals)
☐	☒	For null hypothesis testing, the test statistic (e.g. *F*, *t*, *r*) with confidence intervals, effect sizes, degrees of freedom and *P* value noted<br>*Give P values as exact values whenever suitable.*
☒	☐	For Bayesian analysis, information on the choice of priors and Markov chain Monte Carlo settings
☒	☐	For hierarchical and complex designs, identification of the appropriate level for tests and full reporting of outcomes
☒	☐	Estimates of effect sizes (e.g. Cohen's *d*, Pearson's *r*), indicating how they were calculated

Our web collection on statistics for biologists contains articles on many of the points above.

Software and code

Policy information about availability of computer code

Data collection	No software was used in this study for data collection
Data analysis	SigmaPlot version 13 (pharmacokinetic module for fitting AlphaScreen data). Kintek Global Kinetic Explorer Version 8.0.190823 was used for kinetic modelization.

For manuscripts utilizing custom algorithms or software that are central to the research but not yet described in published literature, software must be made available to editors/reviewers. We strongly encourage code deposition in a community repository (e.g. GitHub). See the Nature Research guidelines for submitting code & software for further information.

Data

Policy information about availability of data

All manuscripts must include a data availability statement. This statement should provide the following information, where applicable:

- Accession codes, unique identifiers, or web links for publicly available datasets
- A list of figures that have associated raw data
- A description of any restrictions on data availability

The source data underlying Figs 3b, 4b, 6d-f, Supplementary Tables S 3, S 4, S 5 and Supplementary Figure S 104 are provided as a Source Data file

Field-specific reporting

Please select the one below that is the best fit for your research. If you are not sure, read the appropriate sections before making your selection.

☒ Life sciences ☐ Behavioural & social sciences ☐ Ecological, evolutionary & environmental sciences

Life sciences study design

All studies must disclose on these points even when the disclosure is negative.

Sample size	The work did not involve a study that required sample size determination
Data exclusions	No data were excluded from the study
Replication	Number of replicates are indicated in the supplementary data. Experiments were reproducible with no exception.
Randomization	The work did not involve a study that required randomization
Blinding	The work did not involve a study that required blinding

Reporting for specific materials, systems and methods

We require information from authors about some types of materials, experimental systems and methods used in many studies. Here, indicate whether each material, system or method listed is relevant to your study. If you are not sure if a list item applies to your research, read the appropriate section before selecting a response.

Materials & experimental systems

n/a	Involved in the study	
☐	☒	Antibodies
☐	☒	Eukaryotic cell lines
☒	☐	Palaeontology
☒	☐	Animals and other organisms
☒	☐	Human research participants
☒	☐	Clinical data

Methods

n/a	Involved in the study	
☒	☐	ChIP-seq
☒	☐	Flow cytometry
☒	☐	MRI-based neuroimaging

Antibodies

Antibodies used	Cell lysates were then analyzed by Western blot using specific total MET (#37-0100 Invitrogen), total ERK2 (#SC-154 Tebu-bio), total AKT (pan, clone C67E7, #4691 Cell Signaling), phospho-MET (Y1234/1235, clone CD26, #3077 Cell Signaling), phospho-Akt (S473, clone CD9E, #4060 Cell Signaling), phospho-ERK (T202/Y204, clone E10, #9106 Cell Signaling) and anti-mouse (#115-035-146) or anti-rabbit (#711-035-152) peroxidase-conjugated IgG secondary antibodies (Jackson ImmunoResearch).
Validation	All antibodies are validated by manufacturers and are used by us for more than 10 years

Eukaryotic cell lines

Policy information about cell lines

Cell line source(s)	HeLa and CAPAN1 were purchased from ATCC in 2008
Authentication	None of the cell lines used were authenticated since purchase in 2008
Mycoplasma contamination	All cells were cultered with ZellShield (Minerva Biolabs) that prevent mycoplasma contamination. Cells are tested annually by PCR (Minerva Biolabs kit) and show mycoplasma levels below detection levels.
Commonly misidentified lines (See ICLAC register)	No commonly misidentified cell lines were used in this study.
